# Rapid Access to Dispirocyclic Scaffolds Enabled by Diastereoselective Intramolecular Double Functionalization of Benzene Rings

**DOI:** 10.1002/asia.202001179

**Published:** 2020-10-23

**Authors:** Hiromasa Yokoe, Yuka Mizumura, Kana Sugiyama, Kejia Yan, Yuna Hashizume, Yuto Endo, Sae Yoshida, Akiko Kiriyama, Masayoshi Tsubuki, Naoki Kanoh

**Affiliations:** ^1^ Institute of Medicinal Chemistry Hoshi University 2-4-41 Ebara, Shinagawa-ku Tokyo 142-8501 Japan

**Keywords:** cyclization, domino reactions, cross coupling, dearomatization, natural products

## Abstract

Here we describe the diastereoselective synthesis of (5*r*,8*r*)‐1,9‐diazadispiro[4.2.4^8^.2^5^]tetradecatrienes via domino double spirocyclization of *N*‐arylamide derivatives. This reaction can serve as a fast way to synthesize diazadispirocycles, which are found in the core structures of bioactive natural products. Product diversification via Suzuki–Miyaura cross coupling and application to the synthesis of 1‐oxa‐9‐azadispiro[4.2.4^8^.2^5^]tetradecatrienes were also conducted.

## Graphical abstract

**Double Spirocyclization**: The present reaction is the first example of double spirocyclization of *N*‐arylamides via dearomatization, where 1,9‐diazadispiro[4.2.4^8^.2^5^]tetradecatrienes were synthesized diastereoselectively.

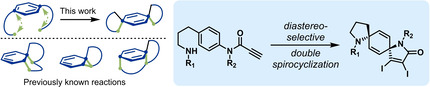

Dearomative transformations play a significant role in organic chemistry.^[1]^ In particular, a double cyclization reaction via dearomatization is attractive because it can facilitate a single‐step formation of two cycles (Figure 1a). However, to date, the application of this method has been limited to angularly fused[^2^, ^3^] and bridged^[4]^ cycles. To the best of our knowledge, dispirocycles have not been synthesized despite the attention they have received in the field of chemical sciences.^[5]^ Here, we report a diastereoselective domino double spirocyclization via dearomatization.

**Figure 1:**
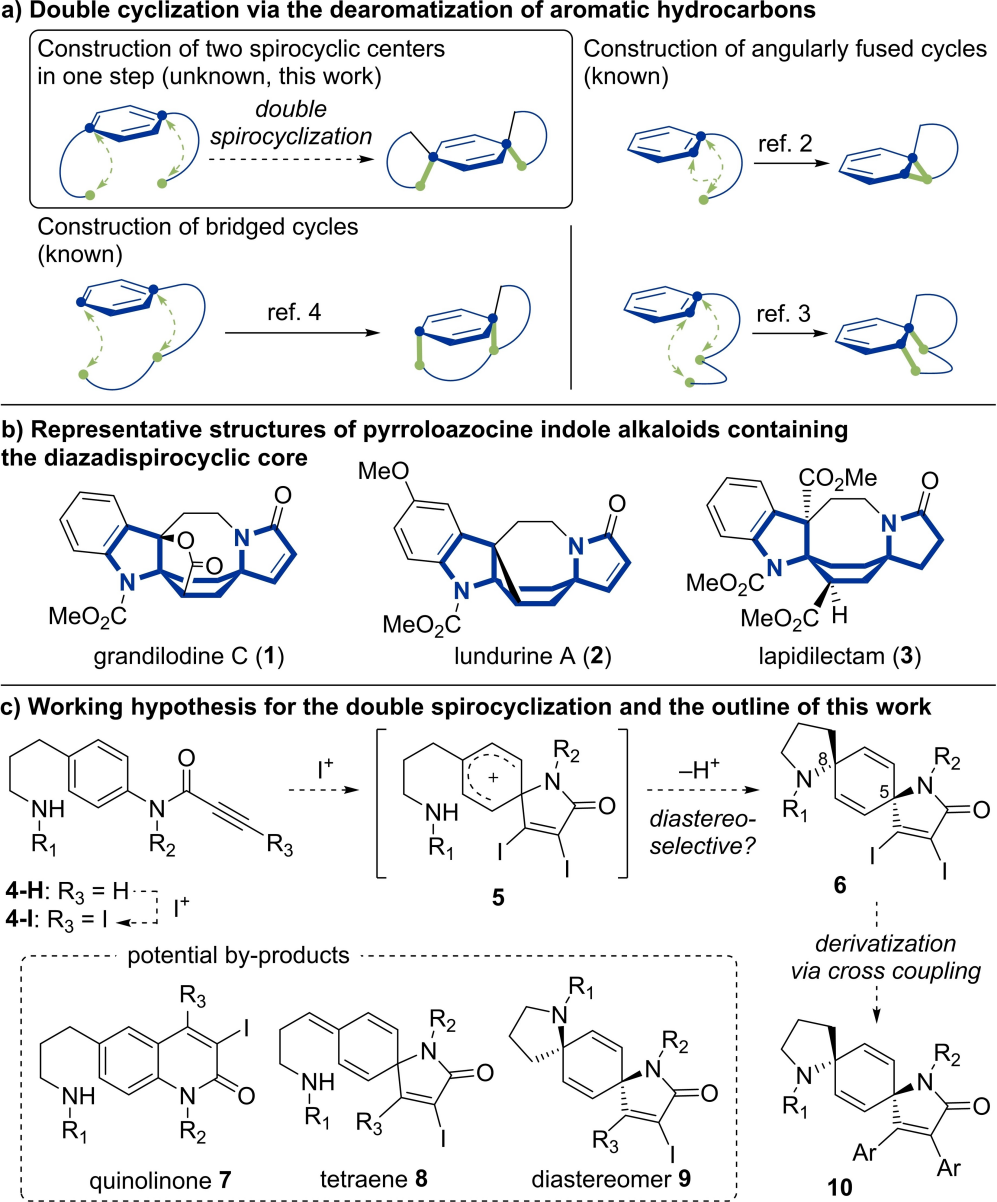
Background and working hypothesis of this work.

In this context, we planned to construct a (5*r*,8*r*)‐1,9‐diazadispiro[4.2.4^8^.2^5^]tetradecatriene scaffold, which is found in the core structures of grandilodine C (**1**),^[6c]^ lundurine A (**2**),^[6d]^ and lapidilectam (**3**)^[6e]^ (Figure 1b). These are known as pyrroloazocine indole alkaloids,^[6]^ which can reverse drug resistance^[7]^ in vincristine‐resistant cell lines. The characteristic diazadispirocyclic scaffold is considered a fascinating synthetic target because, in general, some of the drug seeds and molecular probes are inspired by low‐molecular‐weight fragments derived from natural product sources.^[8]^ However, the cyclic structures have rarely been synthesized,^[9]^ and their diastereoselective synthesis, to the best of our knowledge, has not been reported.

Based on advances in dearomative *ipso*‐cyclization chemistry,^[10]^ especially in the synthesis of cyclohexadienes,[^11^, ^12^] we hypothesized that the following process from **4‐H** toward **6** would occur in one step (Figure 1c). The reaction consists of three steps. Iodination of the terminal alkyne of **4‐H** would give **4‐I**, and the subsequent *ipso*‐iodocyclization^[11]^ would afford cyclohexadienyl cation **5**. Moreover, the resulting cation would be captured^[13]^ by the nitrogen atom of the side chain in a diastereoselective manner. Along with the desired cyclization reaction, quinolinone **7** ^[14]^ and tetraene **8** ^[15]^ might be produced. The diastereoselectivity between **6** and **9** would be varied by substituents R_1_ and R_2_. Furthermore, derivatives **10** would be obtained via a cross‐coupling reaction employing two iodine atoms as chemical handles incorporated in **6**.

As illustrated in Scheme 1, the synthesis of cyclization precursor **4‐Hb** was commenced with commercially available carboxylic acid **11. 11** was converted into carbamate **12** via a Curtius rearrangement in 95% yield, and the nitro group of **12** was reduced with ammonium chloride and iron powder to give aniline **13** quantitatively. Then **13** was condensed with propiolic acid to synthesize amide **4‐Ha** followed by *N*‐benzylation to afford cyclization precursor **4‐Hb**.

**Scheme 1:**
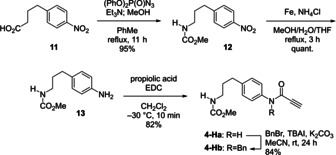
Preparation of precursor **4‐Hb**.

After synthesizing the cyclization precursor **4‐Hb**, we began our studies on the double cyclization (Table 1). As shown in entry 1, treatment of **4‐Hb** with 2.2 equivalent of NIS and AgNO_3_ in MeCN solvent gave (5*r*,8*r*)‐diazadispirocycle **6 b** in 21% ^1^H NMR yield, whose stereochemistry was established by the NOESY experiment. Next, the reaction was performed with NIS and AgNO_3_ in various solvents (entries 2–4). Acetone or CH_2_Cl_2_ was inefficient, while MeNO_2_ worked efficiently and gave relatively clean crude material. Two diastereomers, **6 b** and **9 b**, could be separated through conventional silica gel column chromatography, and **6 b** was isolated in 46% yield. The results of the solvent screening experiment indicated that polar and low‐nucleophilic solvents are suitable for the reaction. Therefore we turned to test fluorinated alcohols (i. e., TFE, TFP, and HFIP), which are known to enhance the halogenation of both aromatics and olefins.^[16]^ As shown in entries 5–7, both yields and dr were improved as the polarlity of the solvent increased, and HFIP gave the best result to give **6 b** in 54% yield with 73 : 27 dr. The amount of AgNO_3_ could be reduced to 0.1 equivalent without affecting the outcome (entry 8), and performing the reaction at 0 °C improved the yield by 68% (entry 9). As in entry 10, replacing the counter anion of silver reagent from nitrate to trifluoroacetate improved the yield by 75%, while the diastereoselectivity did not change. The importance of silver reagents was explored in entries 11 and 12. The reactions with catalytic nitric acid or without any catalysts did not reach completion, although retained diastereoselectivity with 75 : 25 dr. Lastly, as in entry 13, the reaction without silver catalyst in MeNO_2_ did not give **6 b** or even intermediate **4‐Ib**, resulting in the recovery of **4‐Hb**. Through the optimization of the reaction conditions, the product derived from the direct spirocyclization of **4‐Hb** before the formation of **4‐Ib** was not detected.

**Table 1:** Optimization of cyclization using **4‐Hb**.

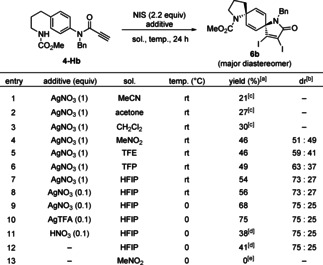

[a] isolated yield of **6 b**; [b] diastereomeric ratio determined by ^1^H NMR of crude material; [c] yield determined by ^1^H NMR of crude material; [d] the reaction did not complete; [e] no reaction: **4‐Hb** was recovered. NIS=*N*‐iodosuccinimide, TFE=2,2,2‐trifluoroethanol, TFP=2,2,3,3‐tetrafluoro‐1‐propanol, HFIP=1,1,1,3,3,3‐hexafluoro‐2‐propanol.

With the optimized conditions in hand, we evaluated the effects of the R_1_ and R_2_ groups on diastereoselectivity (Table 2). The precursors **4‐Hc**−**4‐Hk** were readily prepared via similar synthetic routes for **4‐Hb**. First, we examined the effect of R_1_ on diastereoselectivity, and the substrate **4‐Hc** bearing the *t*‐butoxycarbonyl group showed results comparable to those of **4‐Hb** (entry 1, 2). The reaction employing **4‐Hd** with the *p*‐toluenesulfonyl group for R_1_ proceeded smoothly, although dr decreased to 58 : 42 (entry 3). Next, we tested the effect of the electron density of R_2_ on diastereoselectivity. Thus we performed cyclization with substrates bearing an electron‐donating group (entries 4, 5) or an electron‐withdrawing group (entry 6). Only slight differences were observed among the yields and selectivities among **4‐He**−**4‐Hg**. Lastly, the size of the R_2_ was investigated (entries 7–10). While the methyl group showed low diastereoselectivity, the 2‐naphthylmethyl group gave high selectivity. The best result was obtained when the 1‐naphthylmethyl group was employed, providing (5*r*,8*r*)‐diazadispirocycle **6 k** with 91 : 9 dr.

**Table 2:** Scope of the spirocyclization.

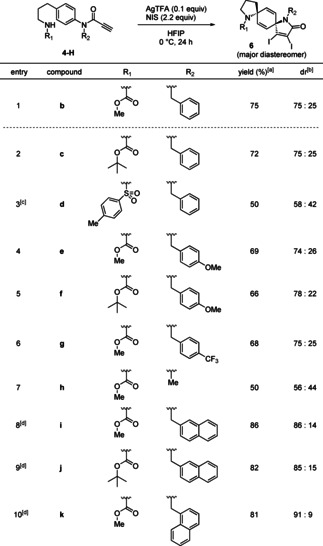

[a] Isolated yield of **6**; [b] diastereomeric ratio determined by ^1^H NMR of crude material; [c] 2.3 equiv. of NIS; [d] 2.5 equiv. of NIS.

The observed stereoselectivity can be explained by considering the conformation of the cationic intermediates produced by the first spirocyclization reaction (Figure 2a). In the second spirocyclization, C−N bond formation should occur to minimize the steric interaction between the R_1_ group of the side chain and the R_2_ or R_3_ group of the lactam ring. The intermediate **A**, leading to the desired isomer **6**, would be predominant if the R_2_ group is more sterically hindered than the R_3_ group, so that the substrate **4‐Hk** with the R_2_ group for a bulkier substituent (R_2_=1‐naphthylmethyl, R_3_=iodine) resulted in the high selective (91 : 9 dr) formation of the desired (5*r*,8*r*)‐diazadispirocycle **6**. In contrast, (5*s*,8*s*)‐isomer was predominant when the precursor with the R_3_ group for a bulkier substituent (R_2_=methyl, R_3_=phenyl) was employed. Thus, the treatment of **4‐Ph** under the same reaction conditions gave **9**–**Ph** as a major product with 60 : 40 dr, as shown in Figure 2b.

**Figure 2:**
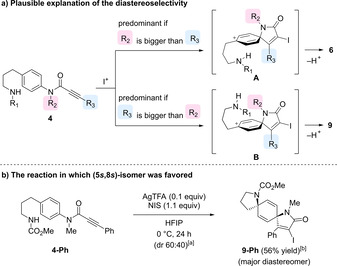
Plausible mechanism of dispirocyclization. [a] Diastereomeric ratio determined by ^1^H NMR of crude material; [b] isolated yield of **9**–**Ph**.

Next, derivatization was conducted by a modification of the diiodo moiety (Scheme 2). Thus, diarylethenes^[17]^ with electron‐rich and electron‐poor aromatics were synthesized via Suzuki–Miyaura cross coupling in the presence of Pd(PPh_3_)_4_ catalyst, Ag_2_O, and CsF. The reaction was completed at room temperature within 2 hours, and **6 e** was converted into diarylethene **10 e**–**1**, having two *p*‐nitrophenyl groups in 78% yield, while the use of an organic base such as K_3_PO_4_ instead of Ag_2_O resulted in partial decomposition. Next, under similar conditions, derivative **10 e**–**2** with two *p*‐methoxyphenyl groups was synthesized in 62% yield.

**Scheme 2:**
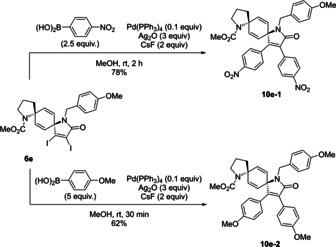
Derivatization of diazadispirocycle **6 e**.

Finally, we extended the above‐developed method to the synthesis of both (5*r*,8*r*)‐ and (5*s*,8*s*)‐diastereomers of 1‐oxa‐9‐azadispiro[4.2.4^8^.2^5^]tetradecatrienes (Scheme 3). First, readily available carboxylic acid **14** was treated with AgTFA and NIS in HFIP, providing (5*r*,8*r*)‐isomer of dispirocycle **15** as a major product in 59% isolated yield with 65 : 35 dr. Second, cyclization of phenylpropiolate **16** afforded (5*s*,8*s*)‐isomer of the dispirocycle **17** in 57% yield with 66 : 34 dr. The stereochemical outcome can be explained in the same way as the corresponding diazadispirocycles in Figure 2.

**Scheme 3:**
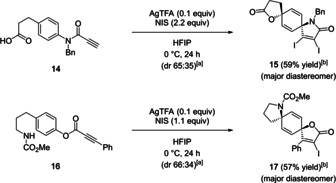
Extension of the cyclization to azaoxadispirocycles; [a] diastereomeric ratio determined by ^1^H NMR of crude material; [b] isolated yield of major diastereomer.

In conclusion, we have developed a diastereoselective double spirocyclization reaction, enabling the one‐step construction of two quaternary centers. The method allowed the synthesis of the diazadispirocyclic core found in the structures of the pyrroloazocine indole alkaloids family. Our discovery indicated that HFIP and silver catalyst could promote both the iodination and spirocyclization processes with NIS, and HFIP appeared to be essential for the high level of diastereoselectivity. The spirocyclization seemed to be triggered by the iodination of terminal alkynes, since no trace of direct cyclization was observed before the iodination. The stereoselectivity was affected by the steric sizes of the lactam ring's substituents but was not affected by the electronic properties. The cyclized product was modified to expand the product diversity. Furthermore, the method developed was applied to the synthesis of 1‐oxa‐9‐azadispiro[4.2.4^8^.2^5^]tetradecatrienes. More extensive applications of the reaction including the total synthesis of pyrroloazocine indole alkaloids are now under way in this laboratory.

## Conflict of interest

The authors declare no conflict of interest.

## Supporting information

As a service to our authors and readers, this journal provides supporting information supplied by the authors. Such materials are peer reviewed and may be re‐organized for online delivery, but are not copy‐edited or typeset. Technical support issues arising from supporting information (other than missing files) should be addressed to the authors.

Supplementary

## References

[1] For selected recent reviews, see:

[1a] C. J. Huck , D. Sarlah , Chem 2020, 6, 1589–1603; 32715154 10.1016/j.chempr.2020.06.015 PMC7380651

[1b] J. M. Saya , E. Ruijter , R. V. A. Orru , Chem. Eur. J. 2019, 25, 8916–8935; 30994212 10.1002/chem.201901130

[1c] T. Dohi , K. Kikushima , H. China , Heterocycles 2019, 98, 1489–1511;

[1d] W. C. Wertjes , E. H. Southgate , D. Sarlah , Chem. Soc. Rev. 2018, 47, 7996–8017; 30073226 10.1039/c8cs00389k

[1e] M. Okumura , D. Sarlah , Synlett 2018, 845–855;

[1f] C. Zheng , S.-L. You , Chem 2016, 1, 830–857;

[1g] *Asymmetric Dearomatization Reactions*, (Ed. S.-L. You), Wiley-VCH, Germany, **2016**;

[1h] L. Pantaine , X. Moreau , V. Coeffard , C. Greck , Tetrahedron Lett. 2016, 57, 2567–2574;

[1i] T. Nemoto , Y. Hamada , J. Syn. Org. Chem. 2015, 73, 977–986;

[1j] S. P. Roche , J. A. Porco Jr , Angew. Chem. Int. Ed. 2011, 50, 4068–4093; 10.1002/anie.201006017 PMC4136767 21506209

[2a] R. R. Nani , S. E. Reisman , J. Am. Chem. Soc. 2013, 135, 7304–7311; 23642221 10.1021/ja401610p

[2b] S. Levin , R. R. Nani , S. E. Reisman , J. Am. Chem. Soc. 2011, 133, 774–776; 21174417 10.1021/ja110192b

[2c] S. Levin , R. R. Nani , S. E. Reisman , Org. Lett. 2010, 12, 780–783; 20088588 10.1021/ol902848k

[2d] M. P. Doyle , D. G. Ene , D. C. Forbes , T. H. Pillow , Chem. Commun. 1999, 1691–1692;

[2e] G. R. King , L. N. Mander , N. J. Monck , J. C. Morris , H. A. Zhang , J. Am. Chem. Soc. 1997, 119, 3828–3829.

[3a] M. J. James , J. L. Schwarz , F. Strieth-Kalthoff , B. Wibbeling , F. Glorius , J. Am. Chem. Soc. 2018, 140, 8624–8628; 29961335 10.1021/jacs.8b03302

[3b] L.-J. Wang , H.-T. Zhu , Y.-F. Qiu , X.-Y. Liu , Y.-M. Liang , Org. Biomol. Chem. 2014, 12, 643; 24297089 10.1039/c3ob42020e

[3c] T. Shibuya , K. Noguchi , K. Tanaka , Angew. Chem. Int. Ed. 2012, 51, 6219–6222; 10.1002/anie.201202165 22566013

[4a] H. Takikawa , A. Nishii , H. Takiguchi , H. Yagishita , M. Tanaka , K. Hirano , M. Uchiyama , K. Ohmori , K. Suzuki , Angew. Chem. Int. Ed. 2020, 59, 12440–12444; 10.1002/anie.202003131 32270569

[4b] A. Nishii , H. Takikawa , K. Suzuki , Chem. Sci. 2019, 10, 3840–3845; 31015926 10.1039/c8sc05518a PMC6461022

[4c] J. Ling , S. Lam , K.-H. Low , P. Chiu , Angew. Chem. Int. Ed. 2017, 56, 8879–8882; 10.1002/anie.201704155 28558142

[4d] Y. Deng , C. Jing , P. Y. Zavalij , M. P. Doyle , Org. Lett. 2015, 17, 4312–4315; 26295847 10.1021/acs.orglett.5b02129 PMC5572213

[4e] D. Niu , T. Wang , B. P. Woods , T. R. Hoye , Org. Lett. 2014, 16, 254–257; 24329102 10.1021/ol403258c PMC3955733

[4f] Y. Schmidt , J. K. Lam , H. V. Pham , K. N. Houk , C. D. Vanderwal J. Am. Chem. Soc. 2013, 135, 7339–7348; 23634642 10.1021/ja4025963 PMC3727913

[4g] K. Kishikawa , S. Akimoto , S. Kohmoto , M. Yamamoto , K. Yamada , J. Chem. Soc. Perkin Trans. 1 1997, 77–84;

[4h] G. Himbert , L. Henn , Angew. Chem. Int. Ed. 1982, 21, 620–620;

[5a] Y.-J. Zheng , C. M. Tice , Expert Opin. Drug Discovery 2016, 11, 831–834; 10.1080/17460441.2016.1195367 27233084

[5b] Y. Zhen , C. M. Tice , S. B. Singh , Bioorg. Med. Chem. Lett. 2014, 24, 3673–3682; 25052427 10.1016/j.bmcl.2014.06.081

[5c] F. Lovering , J. Bikker , C. Humblet , J. Med. Chem. 2009, 52, 6752–6756. 19827778 10.1021/jm901241e

[6] For reviews on pyrroloazocine indole alkaloids, see:

[6a] M. S. Kirillova , F. M. Miloserdov , A. M. Echavarren , Org. Chem. Front. 2018, 5, 273–287;

[6b] S. Arai , M. Nakajima , A. Nishida , in The Alkaloids: Chemistry and Biology (Ed. Knölker H.-J.), Academic Press, 2017, pp. 167–204; 10.1016/bs.alkal.2017.01.001 28838428

[6c] W.-S. Yap , C.-Y. Gan , Y.-Y. Low , Y.-M. Choo , T. Etoh , M. Hayashi , K. Komiyama , T.-S. Kam , J. Nat. Prod. 2011, 74, 1309–1312; 21428274 10.1021/np200008g

[6d] T.-S. Kam , K.-H. Lim , K. Yoganathan , M. Hayashi , K. Komiyama Tetrahedron 2004, 60, 10739–10745;

[6e] K. Awang , T. Sévenet , M. Païs , A. H. A. Hadi , J. Nat. Prod. 1993, 56, 1134–1139.

[7] For selected reviews, see:

[7a] D. Waghray , Q. Zhang , J. Med. Chem. 2018, 61, 5108–5121; 29251920 10.1021/acs.jmedchem.7b01457 PMC6281405

[7b] J. R. Vargas , E. G. Stanzl , N. N. H. Teng , P. A. Wender , Mol. Pharm. 2014, 11, 2553–2565; 24798708 10.1021/mp500161z PMC4123947

[7c] J. Robert , C. Jarry , J. Med. Chem. 2003, 46, 4805–4817. 14584929 10.1021/jm030183a

[8] For selected recent reviews, see:

[8a] X. Chen , Y. Wang , N. Ma , J. Tian , Y. Shao , B. Zhu , Y. K. Wong , Z. Liang , C. Zou , J. Wang , Sig. Transduct. Target. Ther. 2020, 5, 72; 10.1038/s41392-020-0186-y PMC7239890 32435053

[8b] Wang , G. Dong , C. Sheng , Chem. Rev. 2019, 119, 4180–4220; 30730700 10.1021/acs.chemrev.8b00504

[8c] T. Rodriques , D. Reker , P. Schneider , G. Schneider , Nat. Chem. 2016, 8, 531–541; 27219696 10.1038/nchem.2479

[8d] D. J. Newman , G. M. Cragg , J. Nat. Prod. 2016, 79, 629–661. 26852623 10.1021/acs.jnatprod.5b01055

[9a] N. N. Majeed , A. H. Esaa , A. A. Turki Der. Pharm. Chemica 2014, 6, 288–293;

[9b] K. Suzuki , D. G. Mazhukin , H. Takahashi , Y. Uchida , R. Tamura , I. A. Grigor'ev , Heterocycles 2009, 78, 3091–3099.

[10a] C. R. Reddy , S. K. Prajapti , K. Warudikar , R. Ranjan , B. B. Rao , Org. Biomol. Chem. 2017, 15, 3130–3151; 28338704 10.1039/c7ob00405b

[10b] X.-W. Liang , C. Zheng , S.-L. You , Chem. Eur. J. 2016, 22, 11918–11933. 27377184 10.1002/chem.201600885

[11a] V. A. Fiore , M. Keim , R. Werz , G. Maas , Synthesis 2020, 1489–1497;

[11b] Z.-Q. Wang , B.-X. Tang , H.-P. Zhang , F. Wang , J.-H. Li Synthesis 2009, 891–902;

[11c] B.-X. Tang , D.-J. Tang , S. Tang , Q.-F. Yu , Y.-H. Zhang , Y. Liang , P. Zhong , J.-H. Li , Org. Lett. 2008, 10, 1063–1066. 18275209 10.1021/ol703050z

[12] Y. Kikugawa , A. Nagashima , T. Sakamoto , E. Miyazawa , M. Shiiya , J. Org. Chem. 2003, 68, 6739–6744. 12919042 10.1021/jo0347009

[13] For selected reports on the intramolecular O-addition reactions via dearomatization of benzene rings, see:

[13a] A. Fischer , D. R. A. Leonard , R. Röderer Can. J. Chem. 1979, 57, 2527–2532;

[13b] E. J. Corey , S. Barcza , G. Klotmann , J. Am. Chem. Soc. 1969, 91, 4782–4786.

[14a] D. S. Ryabukhin , A. V. Vasilyev , Tetrahedron Lett. 2015, 56, 2200–2202;

[14b] D. S. Ryabukhin , L. Y. Gurskaya , G. K. Fukin , A. V. Vasilyev , Tetrahedron 2014, 70, 6428–6443;

[14c] D. S. Ryabukhin , A. V. Vasil'ev , Russ. J. Org. Chem. 2008, 44, 1849–1851.

[15] Q.-F. Yu , Y.-H. Zhang , Q. Yin , B.-X. Tang , R.-Y. Tang , P. Zhong , J.-H. Li , J. Org. Chem. 2008, 73, 3658–3661. 18376861 10.1021/jo800328a

[16] For a review on HFIP, see:

[16a] I. Colomer , A. E. R. Chamberlain , M. B. Haughey , T. J. Donohoe , Nat. Rev. Chem. 2017, 1, 0088;

[16b] A. M. Arnold , A. Pçthig , M. Drees , T. Gulder , J. Am. Chem. Soc. 2018, 140, 4344–4353; 29412652 10.1021/jacs.8b00113

[16c] R. J. Tang , T. Milcent , B. Crousse , J. Org. Chem. 2018, 83, 930–938; 29256248 10.1021/acs.joc.7b02920

[16d] E. A. Ilardi , C. E. Stivala , A. Zakarian , Org. Lett. 2008, 10, 1727–1730. 18386904 10.1021/ol800341z

[17] For selected reviews on diarylethenes, see:

[17a] M. Irie , T. Fukaminato , K. Matsuda , S. Kobatake , Chem. Rev. 2014, 114, 12174–12277; 25514509 10.1021/cr500249p

[17b] M. Irie , Chem. Rev. 2000, 100, 1685–1716. 11777416 10.1021/cr980069d

