# Supplementary material for: Rapid Access to Dispirocyclic Scaffolds Enabled by Diastereoselective Intramolecular Double Functionalization of Benzene Rings
Source: Chem Asian J. 2020 Oct 23;15(24):4271–4. doi: 10.1002/asia.202001179 (PMC7756633; doi:10.1002/asia.202001179)
Supplement: Supplementary file 1 — Supplementary [file ASIA-15-4271-s001.pdf]

CHEMISTRY
AN ASIAN JOURNAL

Supporting Information

Rapid Access to Dispirocyclic Scaffolds Enabled by Diastereoselective Intramolecular Double Functionalization of Benzene Rings

Hiromasa Yokoe,* Yuka Mizumura, Kana Sugiyama, Kejia Yan, Yuna Hashizume, Yuto Endo, Sae Yoshida, Akiko Kiriyama, Masayoshi Tsubuki, and Naoki Kanoh*© 2020 The Authors. Chemistry - An Asian Journal published by Wiley-VCH GmbH. This is an open access article under the terms of the Creative Commons Attribution License, which permits use, distribution and reproduction in any medium, provided the original work is properly cited.

Table of contents

Material and methods 2

Experimental procedures 3

References 27

NMR spectra of synthetic compounds 28

Materials and methods

^{1}H NMR and ^{13}C NMR were measured in $CDCl_3$ solution at rt otherwise noted using JEOL ECA 600 II (600 MHz) or Bruker Avance III 400 (400 MHz) spectrometers, and the residual solvent peaks were used as internal standard (δ = 7.26 for ^{1}H NMR and δ = 77.0 for ^{13}C NMR). For peak multiplicities, the following abbreviations were used: s, singlet; d, doublet; t, triplet; q, quartet; quint; quintet; m, multiplet. IR spectra were measured on Shimadzu IR Prestige-21 or JASCO FT/IR-4100 spectrometer. High resolution mass spectra (HRMS) were recorded on JEOL JMS-T100LP or JMS-700. Flash column chromatography was performed on silica gel (Fuji Silisia Chemical spherical neutral 40–50 μm using indicated solvent. PTLC was performed with Merck silica gel plate 60 F_{254} 250 μm, and compounds were visualized with UV light. All non-aqueous reactions were performed in glassware dried with heat gun under positive pressure of Ar. Tetrahydrofuran (Kanto Kagaku, dehydrated stabilizer free) and dichloromethane (Fujifilm Wako Chemical, super dehydrated) were used as purchased. Acetonitrile, 2,2,2-trifluotoethanol, 2,2,3,3-tetrafluoro-1-propanol, and 1,1,1,3,3,3-hexafluoro-2-propanol were dried over activated 3A molecular sieves, and acetone and nitromethane were freshly distilled prior to use. NIS was purified by recrystallization prior to use, and other commercially available reagents were used without purification.

Experimental procedures

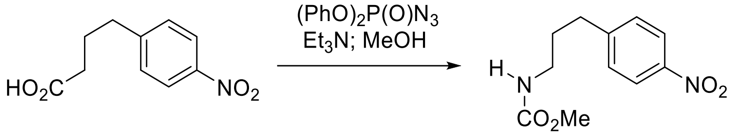

11 12

To a stirred solution of 2.09 g (10.0 mmol) of **11** and 2.79 mL (20.0 mmol, 2 equiv) of trimethylamine in 100 mL of toluene was added dropwise 2.16 mL (10.0 mmol, 1.0 equiv) of diphenylphosphoryl azide, and the resulting solution was stirred for 30 min at rt and refluxed for 1.5 h. To the mixture was added 11.3 mL of methanol at rt and refluxed for 11 h. After cooling to rt, the reaction mixture was concentrated in vacuo and the residue was diluted with $CHCl_3$ then washed with saturated aqueous $NaHCO_3$, water, and brine then dried over Na_2SO_4, and concentrated in vacuo. The crude product was chromatographed on silica gel with a gradient of 30–60% AcOEt/hexane to provide 2.38 g (9.5 mmol, 95% yield) of **12** as a colorless solid.

12: ^{1}H NMR (400 MHz, $CDCl_3$) δ 8.15 (2H, d, J = 8.5 Hz), 7.34 (2H, d, J = 8.5 Hz), 4.72 (1H, s), 3.67 (3H, s), 3.23 (2H, q, J = 6.4 Hz), 2.76 (2H, t, J = 7.8 Hz), 1.83–1.90 (2H, quint., J = 7.2 Hz); ^{13}C NMR (100 Hz, $CDCl_3$) δ 157.1, 149.3, 146.4 129.1 (×2), 123.7 (×2), 52.08, 40.37, 32.82, 31.22; IR (KBr plates) 3327, 2951, 1685, 1517 cm^{-1}; HRMS (ESI) m/z $[M+Na]^+$ calcd for $C_{11}H_{14}N_2NaO_4$ 261.0851, found 261.0881.

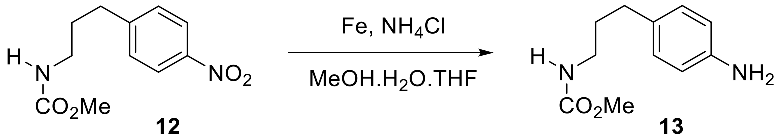

12 13

To a stirred solution of 2.26 g (9.49 mmol) of **12** in 50 mL of a mixed solvent composed of MeOH/H_2O/THF (40/20/40) were added 1.27 g (23.7 mmol, 2.5 equiv) of NH_4Cl and 2.65 g (47.5 mmol, 5 equiv) of iron powder at rt, then the resulting suspension was refluxed for 3.5 h. The mixture was diluted with AcOEt and then filtered through a pad of Celite and washed with AcOEt. The resulting organic layer was separated and the aqueous layer was extracted with AcOEt. The combined organic layers were dried over Na_2SO_4 and concentrated in vacuo. The crude product was chromatographed on silica gel eluting with a gradient of 1–6% MeOH/$CHCl_3$ to give 1.98 g (9.49 mmol, quant.) of **13** as a pale orange solid.

13: ^{1}H NMR (600 MHz, $CDCl_3$) δ 6.96 (2H, d, J = 7.2 Hz), 6.63 (2H, d, J = 7.9 Hz), 4.67 (1H, s), 3.65 (3H, s), 3.19 (2H, q, J = 6.4 Hz), 2.53 (2H, t, J = 7.7 Hz), 1.74–1.79 (2H, quint., J = 7.2 H); ^{13}C NMR (150 MHz, $CDCl_3$) δ 156.9, 144.2, 131.1, 128.8 (×2), 115.1 (×2), 51.74, 40.36, 31.87, 31.59; IR (KBr plates) 3412, 3284, 1718, 1551 cm^{-1}; HRMS (ESI) m/z $[M+Na]^+$ calcd for $C_{11}H_{16}N_2NaO_2$

231.1110, found 231.1090.

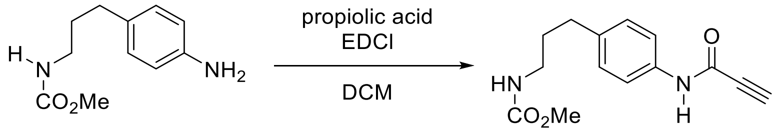

13 **4-Ha**

To a stirred solution of 1.05 g (5.05 mmol) of **13** in 10 mL of CH_2Cl_2 were added 0.47 mL (7.56 mmol) of propiolic acid and 1.45 g (7.56 mmol) of EDC at –30 °C, and the stirring was continued at the same temperature for 10 min. The reaction was diluted with water. After the organic layer separation, the aqueous layer was extracted with $CHCl_3$. The combined organic layers were dried over Na_2SO_4 and concentrated in vacuo. The crude product was chromatographed on silica gel with a gradient of 2–8% MeOH/$CHCl_3$ to give 1.08 g (4.14 mmol, 82% yield) of **4-Ha** as a pale yellow oil.

4-Ha: ^{1}H NMR (400 MHz, $CDCl_3$) δ 7.74 (1H, s), 7.43 (2H, d, *J* = 8.5 Hz), 7.13 (2H, d, *J* = 8.5 Hz), 4.72 (1H, s), 3.66 (3H, s), 3.13–3.23 (2H, m), 2.91 (1H, s), 2.61 (2H, t, *J* = 7.7 Hz), 1.76–1.83 (2H, m); ^{13}C NMR (100 MHz, $CDCl_3$) δ 157.2, 149.8, 138.2, 135.1, 128.8 (×2), 120.3 (×2), 77.61, 74.02, 52.06, 40.47, 32.31, 31.46; IR (neat) 3303, 2960, 2109, 1689, 1648, 1601, 1561, 1535, 1270, 1256, 812 cm^{-1}; HRMS (ESI) *m*/*z* [M+Na]$^+$ calcd for $C_{14}H_{16}N_2NaO_3$ 283.1059, found 283.1060.

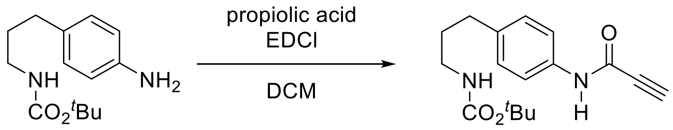

S01 **S02**

To a stirred solution of 3.00 g (12.0 mmol) of **S01**[1] in 30 mL of CH_2Cl_2 were added 10 mL (16.8 mmol, 1.4 equiv) of propiolic acid and 3.22 g (16.8 mmol, 1.4 equiv) of EDC at –30 °C, and the stirring was continued at the same temperature for 10 min. The reaction was diluted with water. After the organic layer separation, the aqueous layer was extracted with $CHCl_3$. The combined organic layers were dried over Na_2SO_4 and concentrated in vacuo. The crude product was chromatographed on silica gel with a gradient of 2–8% MeOH/$CHCl_3$ to give 2.2 g (7.3 mmol, 83% yield) of **S02** as a pale yellow oil.

S02: ^{1}H NMR (600 MHz, $CDCl_3$) δ 7.93 (1H, s), 7.43 (2H, d, *J* = 8.2 Hz), 7.12 (2H, d, *J* = 8.2 Hz), 4.59 (1H, s), 3.13 (2H, q, *J* = 6.5 Hz), 2.91 (1H, s), 2.59 (2H, t, *J* = 7.6 Hz), 1.74–1.79 (2H, m), 1.44 (9H, s); ^{13}C NMR (150 MHz, $CDCl_3$) δ 155.98, 149.64, 138.50, 134.94, 128.90 (×2), 120.20 (×2), 79.20, 77.64, 73.94, 40.07, 32.48, 31.64, 28.39 (×3); IR (neat) 3276, 2974, 2104, 1706, 1649, 1600, 1521, 1413, 1254, 1167, 884 cm^{-1}; HRMS (ESI) *m*/*z* [M+Na]$^+$ calcd for $C_{17}H_{22}N_2NaO_3$ 325.1528, found 325.1535.

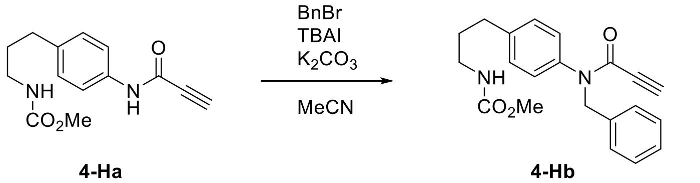

4-Ha **4-Hb**

To a stirred solution of 3.43 g (13.2 mmol) of **4-Ha** in 52.8 mL of MeCN were added 5.50 g (39.6 mmol, 3 equiv) of K_2CO_3, 6.3 mL (52.7 mmol, 4 equiv) of benzyl bromide, and 0.49 g (1.52 mmol, 0.1 equiv) of TBAI at rt, and the stirring was continued at the same temperature for 24 h. The reaction mixture was quenched with saturated aqueous NH_4Cl and diluted with AcOEt. After the organic layer separation, the aqueous layer was extracted with AcOEt. The combined organic layers were washed with brine, dried over Na_2SO_4 and concentrated in vacuo. The crude product was chromatographed on silica gel with a gradient of 20–60% AcOEt/hexane to give 3.90 g (11.1 mmol, 84% yield) of **4-Hb** as a yellow oil.

4-Hb: ^{1}H NMR (400 MHz, $CDCl_3$) (A mixture of rotamers in 85:15 ratio) δ 7.23–7.31 (3H, m), 7.18–7.20 (2H, m), 7.12 (2H, d, J = 8.3 Hz), 6.99 (2H, d, J = 8.0 Hz), 5.15 (0.3H, s), 4.90 (1.7H, s), 4.71 (1H, s), 3.65 (3H, s), 3.23 (0.15H, s), 3.19 (2H, q, J = 6.3 Hz), 2.82 (0.85H, s), 2.64 (1.7H, t, J = 7.8 Hz), 2.58 (0.3H, t, J = 7.8 Hz), 1.78–1.85 (2H, m); ^{13}C NMR (150 MHz, $CDCl_3$) δ 157.01, 153.17, 141.78, 138.92, 128.98 (×2), 128.71 (×2), 128.45 (×2), 128.35 (×2), 127.61, 80.07, 76.23, 52.42, 52.02, 40.52, 32.53, 31.31; IR (neat) 3343, 2945, 2109, 1714, 1634, 1515, 1395, 1260, 1029, 735 cm^{-1}; HRMS (ESI) m/z [M+Na]$^+$ calcd for $C_{21}H_{22}N_2NaO_3$ 373.1528, found 373.1512.

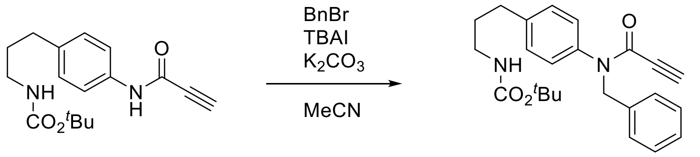

S02 **4-Hc**

To a stirred solution of 3.60 g (12.0 mmol) of **S02** in 50 mL of MeCN were added 2.50 g (18.0 mmol, 1.5 equiv) of K_2CO_3, 0.170 g of TBAI (1.20 mmol, 0.1 equiv.), and 2.10 mL (18.0 mmol, 1.5 equiv) of benzyl bromide at rt, and stirring was continued at the same temperature for 24 h. The reaction mixture was diluted with AcOEt and filtered through a pad of Celite, and concentrated in vacuo. The crude product was chromatographed on silica gel with a gradient of 20–60% AcOEt/hexane to give 3.37 g (8.59 mmol, 72 %) of **4-Hc** as a pale yellow oil.

4-Hc: ^{1}H NMR (400 MHz, $CDCl_3$) (A mixture of rotamer in 85:15 ratio) δ 7.25–7.30 (3H, m), 7.20 (2H, dd, J = 7.2, 2.1 Hz), 7.12 (2H, d, J = 8.5 Hz), 6.99 (2H, d, J = 8.3 Hz), 5.15 (0.3H, s), 4.90 (1.7H, s), 4.53 (0.85H, s), 4.26 (0.15H, s) 3.23 (0.15H, s), 3.00–3.15 (2H, m), 2.83 (0.85H, s), 2.63 (1.7H, t, J = 8.0 Hz), 2.59 (0.3H, t, J = 8.4 Hz), 1.76–1.83 (2H, m), 1.44 (9H, s); ^{13}C NMR (100 MHz, $CDCl_3$) δ 155.81, 153.02, 141.83, 138.63, 136.12, 128.86 (×2), 128.49 (×2), 128.29 (×2), 128.09 (×2), 127.44, 80.13, 78.87, 76.07, 55.89*, 52.25, 39.88, 32.43, 31.25, 28.24 (×3); IR (neat)

2977, 2108, 1652, 1635, 1511, 1394, 1367, 1278, 737 cm^{-1}; HRMS (ESI) *m*/*z* $[M+Na]^+$ calcd for $C_{24}H_{28}N_2NaO_3$ 415.1998, found 415.1998.

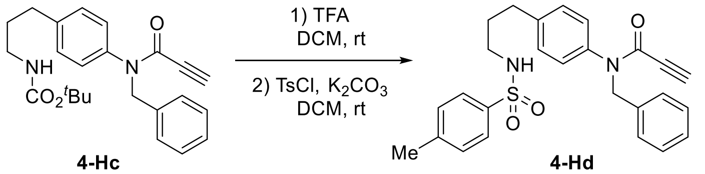

4-Hc 4-Hd

To a stirred solution of 0.392 g (1.00 mmol) of **4-Hc** in 2.0 mL of CH_2Cl_2 was added 0.78 mL (10.0 mmol, 10 equiv) of TFA at rt, and the stirring was continued at the same temperature for 1 h. The reaction mixture was concentrated in vacuo. The resulting orange oil was dissolved in 3.0 mL of CH_2Cl_2, and to the solution were added 0.415 g (3.00 mmol, 3 equiv.) of K_2CO_3 and 0.191 g (1.00 mmol, 1 equiv) of TsCl at 0 °C, and the stirring was continued at rt for 24 h. The reaction mixture was diluted with CH_2Cl_2 and washed with water and brine, dried over Na_2SO_4, concentrated in vacuo. The crude product was chromatographed on silica gel with a gradient of 10–40% AcOEt/hexane to give 0.300 g (0.670 mmol, 67%) of **4-Hd** as a pale yellow oil.

4-Hd: ^{1}H NMR (600 MHz, $CDCl_3$) (A mixture of rotamers in 85:15 ratio) δ 7.71 (2H, d, *J* = 8.2 Hz), 7.25–7.30 (5.3H, m), 7.18–7.20 (1.7H, m), 7.03 (2H, d, *J* = 8.2 Hz), 6.96 (2H, d, *J* = 8.2 Hz), 5.14 (0.3H, s), 4.90 (1.7H, s), 4.62 (1H, t, *J* = 6.2 Hz), 3.23 (0.15H, s), 2.93 (2H, q, *J* = 6.6 Hz), 2.82 (0.75H, s), 2.60 (1.7H, t, *J* = 7.6 Hz), 2.54 (0.3H, t, *J* = 7.7 Hz), 2.42 (2.55H, s), 2.41 (0.45H, s), 1.71–1.80 (2H, m); ^{13}C NMR (150 MHz, $CDCl_3$) δ 153.18, 143.45, 141.29, 139.04, 136.83, 136.27, 129.70 (×2), 129.03 (×2), 128.71 (×2), 128.48 (×2), 128.43 (×2), 127.65, 127.04 (×2), 80.21, 76.21, 52.41, 42.55, 32.30, 30.84, 21.50; IR (neat) 3274, 2930, 2109, 1633, 1512, 1397, 1325, 1159, 1094, 753 cm^{-1}; HRMS (ESI) *m*/*z* $[M+Na]^+$ calcd for $C_{26}H_{26}N_2NaO_3S$ 469.1562, found 469.1581.

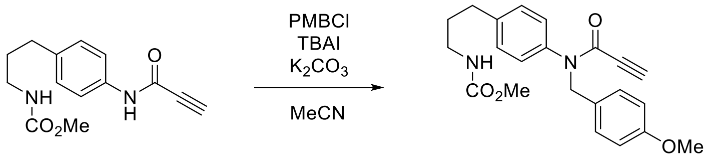

4-Ha 4-He

To a stirred solution of 0.300 g (1.20 mmol) of **4-Ha** in 6.0 mL of MeCN were added 0.480 g (3.50 mmol, 3 equiv) of K_2CO_3, 0.470 mL (3.50 mmol, 3 equiv) of 4-methoxybenzyl chloride, and 0.016 g (0.120 mmol, 0.1 equiv) of TBAI at rt, and the stirring was continued at the same temperature for 24 h. The reaction mixture was quenched with saturated aqueous NH_4Cl and diluted with AcOEt. After the organic layer separation, the aqueous layer was extracted with AcOEt. The combined organic layers were washed with brine, dried over Na_2SO_4, and concentrated in vacuo. The crude product was chromatographed on silica gel with a gradient of 10–40% AcOEt/hexane to give 0.380 g (1.00 mmol, 87% yield) of **4-He** as a colorless viscous oil,

4-He: ^{1}H NMR (600MHz, $CDCl_3$) δ 7.09 (2H, d, *J* = 8.4 Hz) 7.07 (2H, d, *J* = 8.4 Hz), 6.93 (2H, d, *J* = 8.2 Hz), 6.79 (0.15H, d, *J* = 8.6 Hz), 6.75 (1.85H, d, *J* = 8.9 Hz), 5.05 (0.3H, s), 4.85 (1H, s), 4.80 (1.7H, s), 3.74 (3H, s), 3.61 (3H, s), 3.25 (0.15H, s), 3.16 (2H, q, *J* = 6.3 Hz), 2.79 (0.75H, s), 2.61 (1.7H, t, *J* = 7.7 Hz), 2.55 (0.3H, t, *J* = 7.7 Hz), 1.75–1.83 (2H, m); ^{13}C NMR (150 MHz, $CDCl_3$) (Asterisks indicate peaks for the minor rotamer) δ 158.97, 157.01, 153.00, 141.73, 138.79, 130.11 (×2), 128.98* (×2), 128.91 (×2), 128.40 (×2), 126.71, 113.89* (×2), 113.71 (×2), 79.95, 76.25, 55.10, 51.91*, 51.74, 40.46, 32.49, 31.22; IR (neat) 3341, 3282, 2106, 1710, 1634, 1514, 1395, 1249, 1031, 762, 734 cm^{-1}; HRMS (ESI) *m/z* $[M+H]^+$ calcd for $C_{22}H_{25}N_2O_4$ 381.1814, found 381.1811.

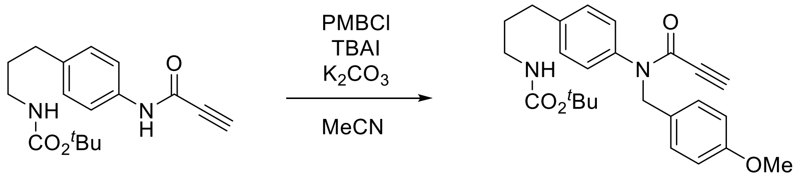

S02 **4-Hf**

To a stirred solution of 0.500 g (1.65 mmol) of **S02** in 8.0 mL of MeCN were added 0.343 g (2.48 mmol, 1.5 equiv) of K_2CO_3, 0.340 mL (2.48 mmol, 1.5 equiv) of 4-methoxybenzyl chloride, and 0.023 g (0.165 mmol, 0.1 equiv) of TBAI at rt, and the stirring was continued at the same temperature for 24 h. The reaction mixture was filtered through a pad of Celite, and concentrated in vacuo. The crude product was chromatographed on silica gel with a gradient of 20–40% AcOEt/hexane to give 0.314 g (1.65 mmol, 45% yield) of **4-Hf** as a colorless viscous oil.

4-Hf: ^{1}H NMR (600 MHz, $CDCl_3$) (A mixture of rotamers in 85:15 ratio) δ 7.11–7.15 (4H, m), 6.96 (2H, d, *J* = 8.2 Hz), 6.83 (0.3H, d, *J* = 8.6 Hz) 6.79 (1.7H, d, *J* = 8.6 Hz), 5.08 (0.3H, s), 4.83 (1.7H, s), 4.54 (1H, s), 3.78 (3H, s), 3.24 (0.15H, s), 3.10–3.16 (2H, m), 2.81 (0.85H, s), 2.63 (1.7H, t, *J* = 7.7 Hz), 2.58 (0.3H, t, *J* = 7.9 Hz), 2.17 (0.15H, s) 1.75–1.83 (2H, m), 1.44 (9H, s); ^{13}C NMR (150 MHz, $CDCl_3$) δ 159.05, 155.92, 153.09, 141.89, 138.86, 130.23, 129.10, 129.00, 128.51, 128.47, 126.77, 113.97, 113.79, 79.91, 79.24, 76.35, 55.20, 51.85, 40.10, 32.65, 31.47, 28.40; IR (neat) 2976, 2107, 1703, 1635, 1513, 1392, 1249, 759 cm^{-1}; HRMS (ESI) *m/z* $[M+H]^+$ calcd for $C_{25}H_{31}N_2O_4$ 423.2284, found 423.2260.

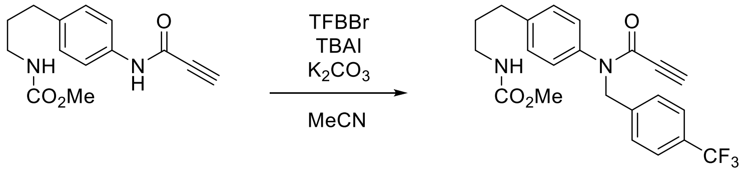

4-Ha **4-Hg**

To a stirred solution of 0.380 g (1.46 mmol) of **4-Ha** in 8.0 mL of MeCN were added 0.303 g (2.19 mmol, 1.5 equiv) of K_2CO_3, 0.523 g (2.19 mmol, 1.5 equiv) of 4-(trifluoromethyl)benzyl bromide,

and 0.021 g (0.146 mmol, 0.1 equiv) of TBAI at rt, and the stirring was continued at the same temperature for 24 h. The reaction mixture was quenched with saturated aqueous NH_4Cl and diluted with AcOEt. After the organic layer separation, the aqueous layer was extracted with AcOEt. The combined organic layers were washed with brine, dried over Na_2SO_4, and concentrated in vacuo. The crude product was chromatographed on silica gel with a gradient of 20–50% AcOEt/hexane to give 0.504 g (1.20 mmol, 83% yield) of **4-Hg** as colorless viscous oil.

4-Hg: ^{1}H NMR (400 MHz, $CDCl_3$) (A mixture of rotamers in 85:15 ratio) δ 7.59 (0.3H, d, *J* = 8.0 Hz), 7.54 (1.7H, d, *J* = 8.0 Hz), 7.37 (0.3H, d, *J* = 8.0 Hz), 7.33 (1.7H, d, *J* = 8.0 Hz), 7.15 (2H, d, *J* = 8.3 Hz), 7.00 (2H, d, *J* = 8.3 Hz), 5.21 (0.3H, s), 4.95 (1.7H, s), 4.68 (0.85H, s), 4.49 (0.15H, s), 3.65 (3H, s), 3.24 (0.15H, s), 3.20 (2H, q, *J* = 6.6 Hz), 2.86 (0.85H, s), 2.65 (1.7H, t, *J* = 7.8 Hz), 2.60 (0.3H, t, *J* = 7.8 Hz), 1.79–1.86 (2H, m); ^{13}C NMR (100 MHz, $CDCl_3$) (Asterisks indicate peaks for the minor rotamer) δ 157.01, 153.24, 142.08, 140.23, 138.57, 129.75 (q, *J* = 32.1 Hz), 129.15 (×2), 128.81 (×2), 128.05 (×2), 125.59 (q, *J* = 3.6 Hz)* (×2), 125.37 (q, *J* = 3.6 Hz) (×2), 123.92 (q, *J* = 270 Hz), 80.54, 75.86, 55.46*, 51.98, 51.92, 40.40, 32.43, 31.20; IR (neat) 3344, 2946, 2019, 1713, 1641, 1514, 1327, 1226, 1113 cm^{-1}; HRMS (ESI) *m/z* [M+Na]$^+$ calcd for $C_{22}H_{21}F_3N_2NaO_3$ 441.1402, found 441.1391.

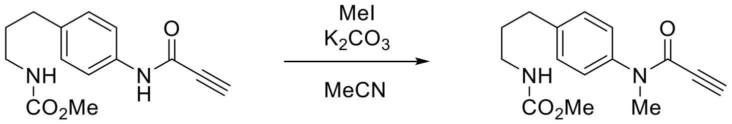

4-Ha **4-Hh**

To a stirred solution of 0.500 g (1.92 mmol) of **4-Ha** in 10.0 mL of MeCN were added 0.796 g (5.76 mmol, 3 equiv) of K_2CO_3, 0.480 mL (7.68 mmol, 4 equiv) of methyl iodide at rt, and the stirring was continued at the same temperature for 24 h. The reaction mixture was quenched with saturated aqueous NH_4Cl and diluted with AcOEt. After the organic layer separation, the aqueous layer was extracted with AcOEt. The combined organic layers were dried over Na_2SO_4, and concentrated in vacuo. The crude product was chromatographed on silica gel with a gradient of 20–60% AcOEt/hexane to give 0.480 g (1.75 mmol, 91% yield) of **4-Hh** as colorless solid.

4-Hh: ^{1}H NMR (400 MHz, $CDCl_3$) (A mixture of rotamers in 85:15 ratio) δ 7.15–7.21 (4H, m), 4.92 (1H, s), 3.63 (3H, s), 3.55 (0.45H, s) 3.29 (0.15H, s) 3.27 (2.55H, s), 3.15–3.21 (2H, m), 2.81 (0.85H, s), 2.63 (1.7H, t, *J* = 7.7 Hz), 2.61 (0.3H, t, *J* = 7.8 Hz) 1.79–1.86 (2H, m); ^{13}C NMR (100 MHz, $CDCl_3$) (Asterisks indicate peaks for the minor rotamer) δ 157.02, 152.97, 141.57, 140.32, 129.08 (×2), 129.00* (×2), 127.03 (×2), 125.22* (×2), 79.57, 76.16, 51.94, 40.42, 36.43, 32.44, 31.33; IR (KBr plates) 3337, 3305, 3233, 3227, 3219, 2496, 2106, 1706, 1640, 1514, 1262 cm^{-1}; HRMS (ESI) *m/z* [M+Na]$^+$ calcd for $C_{15}H_{18}N_2NaO_3$ 297.1215, found 297.1244.

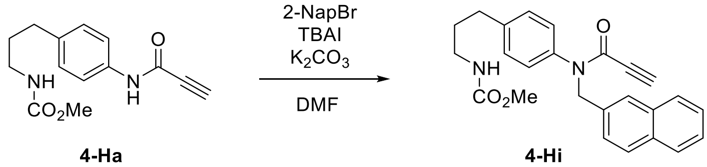

4-Ha **4-Hi**

To a stirred solution of 0.600 g (1.21 mmol) of **4-Ha** in 5.0 mL of DMF were added 0.420 g (3.04 mmol, 2.5 equiv) of K_2CO_3, 0.400 g (1.82 mmol, 1.5 equiv) of 2-naphthylmethyl bromide, and 0.045 g (0.121 mmol, 0.1 equiv) of TBAI at rt, and the stirring was continued at the same temperature for 20 h. The reaction mixture was quenched with saturated aqueous NH_4Cl and diluted with AcOEt. After the organic layer separation, the aqueous layer was extracted with AcOEt. The combined organic layers were washed with water and brine, dried over Na_2SO_4 and concentrated in vacuo. The crude product was chromatographed on silica gel with a gradient of 10–50% AcOEt/hexane to give 0.158 g (0.395 mmol, 32% yield) of **4-Hi** as a yellow viscous oil.

4-Hi: ^{1}H NMR (600 MHz, $CDCl_3$) (A mixture of rotamers in 85:15 ratio) δ 7.80–7.84 (1H, m), 7.77–7.79 (1H, m), 7.73–7.76 (1H, m), 7.64 (0.15H, s), 7.60 (0.85H, s), 7.43–7.49 (2H, m), 7.41 (0.15H, dd, J = 8.4, 1.5 Hz), 7.38 (0.85H, dd, J = 8.6, 1.7 Hz), 7.10 (2H, d, J = 8.2 Hz), 7.04 (0.3H, d, J = 8.6 Hz), 7.00 (1.7H, d, J = 8.6 Hz), 5.32 (0.3H, s), 5.07 (1.7H, s), 4.63 (0.85H, s), 4.45 (0.15H, s), 3.65 (3H, s), 3.24 (0.15H, s), 3.18 (1.7H, q, J = 6.5 Hz), 3.08–3.21 (0.3H, m) 2.85 (0.85H, s), 2.62 (1.7H, t, J = 7.7 Hz), 2.57 (0.3H, t, J = 7.7 Hz), 1.75–1.83 (2H, m).; ^{13}C NMR (150 MHz, $CDCl_3$) (Asterisks indicate peaks for the minor rotamer) δ 156.99, 153.30, 141.82, 138.82, 133.81, 133.14, 132.75, 129.06* (×2), 129.00 (×2), 128.34 (×2), 128.32* (×2), 127.78, 127.64, 127.59, 126.54, 126.49, 126.07, 125.94, 80.23, 76.22, 56.23*, 52.50, 51.98, 40.47, 32.49, 31.23; IR (neat) 3319, 2945, 2109, 1714, 1635, 1513, 1397, 1278, 757 cm^{-1}; HRMS (ESI) m/z $[M+Na]^+$ calcd for $C_{25}H_{24}N_2NaO_3$ 423.1685, found 423.1666.

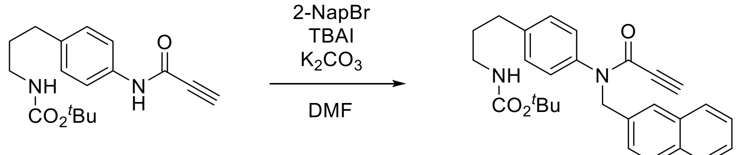

S02 **4-Hj**

To a stirred solution of 0.500 g (1.65 mmol) of **4-Ha** in 8.0 mL of DMF were added 0.343 g (2.48 mmol, 1.5 equiv) of K_2CO_3, 0.548 g (2.48 mmol, 1.5 equiv) of 2-naphthylmethyl bromide, and 0.023 g (0.165 mmol, 0.1 equiv) of TBAI at rt, and the stirring was continued at the same temperature for 21 h. The reaction mixture was quenched with saturated aqueous NH_4Cl and diluted with AcOEt. After organic layer separation, the aqueous layer was extracted with AcOEt. The combined organic layers were washed with water and brine, dried over Na_2SO_4 and concentrated in vacuo. The crude product was chromatographed on silica gel with a gradient of 20–60% AcOEt/hexane to give 0.300 g (0.165 mmol, 41% yield) of **4-Hj** as a yellow oil.

4-Hj: ^{1}H NMR (600 MHz, $CDCl_3$) (A mixture of rotamers in 85:15 ratio) δ 7.74–7.83 (3H, m), 7.64 (0.15H, s), 7.61 (0.85H, s), 7.44–7.49 (2H, m), 7.41 (0.15H, dd, *J* = 8.4, 1.5 Hz), 7.38 (0.85H, dd, *J* = 8.6, 1.7 Hz), 7.10 (2H, d, *J* = 8.2 Hz), 7.04 (0.3H, d, *J* = 8.2 Hz), 6.99 (1.7H, d, *J* = 8.2 Hz), 5.32 (0.3H, s), 5.07 (1.7H, s), 4.51 (0.85H, s), 4.24 (0.15H, s), 3.24 (0.15H, s), 3.08–3.15 (2H, m), 2.85 (0.75H, s), 2.61 (1.7H, t, *J* = 7.7 Hz), 2.56 (0.3H, t, *J* = 7.7 Hz), 1.75–1.80 (2H, m), 1.43 (9H, s); ^{13}C NMR (150 MHz, $CDCl_3$) (Asterisks indicate peaks for the minor rotamer) δ 155.88, 153.29, 141.95, 138.78, 133.83, 133.13, 132.74, 129.07* (×2), 129.01 (×2), 128.54* (×2), 128.29 (×2), 127.79, 127.63, 127.58, 126.54 (×2), 126.06, 125.93, 80.22, 79.14, 76.24, 56.22*, 52.50, 40.02, 32.55, 31.37, 28.35 (×3); IR (neat) 3296, 3056, 2977, 2108, 1703, 1623, 1511, 1394, 1170, 910, 735 cm^{-1}; HRMS (ESI) *m*/*z* $[M+Na]^+$ calcd for $C_{28}H_{30}N_2NaO_3$ 465.2154, found 465.2164.

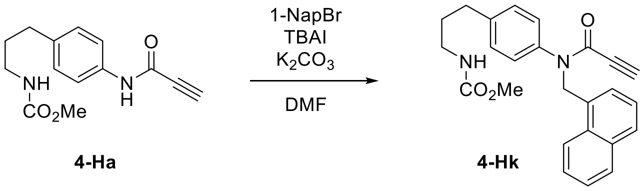

4-Ha **4-Hk**

To a stirred solution of 0.316 g (1.21 mmol) of **4-Ha** in 5.0 mL of DMF were added 0.420 g (3.04 mmol, 2.5 equiv) of K_2CO_3, 0.400 g (1.82 mmol, 1.5 equiv) of 1-naphthylmethyl bromide, and 0.045 g (0.121 mmol, 0.1 equiv) of TBAI at rt, and the stirring was continued at the same temperature for 20 h. The reaction mixture was quenched with saturated aqueous NH_4Cl and diluted with AcOEt. After the organic layer separation, the aqueous layer was extracted with AcOEt. The combined organic layers were washed with water and brine, dried over Na_2SO_4 and concentrated in vacuo. The crude product was chromatographed on silica gel with a gradient of 20–50% AcOEt/hexane to give 0.158 g (0.395 mmol, 32% yield) of **4-Hk** as a yellow viscous oil.

4-Hk: ^{1}H NMR (600 MHz, $CDCl_3$) (A mixture of rotamers in 85:15 ratio) δ 8.08 (0.85H, d, *J* = 8.2 Hz), 7.97 (0.15H, d, *J* = 7.6 Hz), 7.88–7.89 (0.15H, m), 7.84–7.85 (0.85H, m), 7.74–7.79 (0.15H, m), 7.75 (0.85H, d, *J* = 8.2 Hz), 7.48–7.54 (2H, m), 7.40–7.42 (0.15H, m), 7.25–7.27 (0.85H, m), 7.11 (0.85H, d, *J* = 7.2 Hz), 7.05–7.08 (0.3H, m), 7.00 (1.7H, d, *J* = 8.2 Hz), 6.83 (1.7H, d, *J* = 8.2 Hz), 5.65 (0.30H, s), 5.40 (1.7H, s), 4.62 (0.85H, s), 4.21 (0.15H, s), 3.65 (3H, s), 3.13 (1.7H, q, *J* = 6.4 Hz), 3.05–3.18 (0.45H, m), 2.82 (0.85H, s), 2.58 (1.7H, t, *J* = 7.7 Hz), 2.55 (0.3H, t, *J* = 7.7 Hz) 1.74–1.79 (2H, m), 1.57 (2H, s); ^{13}C NMR (150 MHz, $CDCl_3$) δ 156.97, 152.95, 141.74, 138.15, 133.59, 131.47, 131.32, 128.75 (×2), 128.59, 128.57, 128.42 (×2), 128.18, 126.55, 125.81, 124.93, 123.72, 80.18, 76.21, 51.94, 49.83, 40.40, 32.43, 31.18; IR (neat) 3341, 3288, 2944, 2107, 1713, 1635, 1513, 1264, 780, 755 cm^{-1}; HRMS (ESI) *m*/*z* $[M+H]^+$ calcd for $C_{25}H_{25}N_2O_3$ 401.1865, found 401.1885.

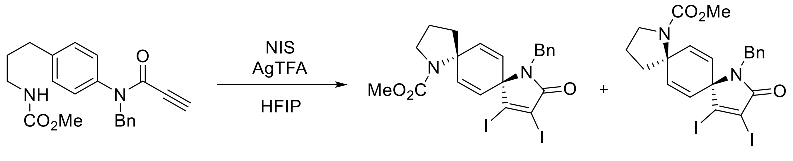

4-Hb **6b** **9b**

To a stirred solution of 64.5 mg (0.180 mmol) of **4-Hb** in 2.6 mL of HFIP were added 4.0 mg (0.018 mmol, 0.1 equiv) of AgTFA followed by 91 mg (0.40 mmol, 2.2 equiv) of NIS at 0 °C, then the stirring was continued at the same temperature for 24 h. The reaction mixture was quenched with 10% aqueous Na_2SO_3 and diluted with $CHCl_3$. After the organic layer separation, the aqueous layer was extracted with $CHCl_3$. The combined organic layers were dried over Na_2SO_4 and concentrated in vacuo. The crude product was chromatographed on silica gel with a gradient of 10–40% AcOEt/hexane to give 12.9 mg (0.022 mmol, 12% yield) of **9b** as a colorless solid followed by 83 mg (0.135 mmol, 75% yield) of **6b** as a colorless solid.

6b: ^{1}H NMR (400 MHz, $CDCl_3$) δ 7.18–7.28 (5H, m), 6.14 (2H, d, *J* = 10.0 Hz), 5.15–5.28 (2H, m), 4.48 (2H, s), 3.68 (3H, s), 3.55–3.67 (2H, m), 1.95–2.05 (2H, m), 1.91–1.95 (2H, m);^{13}C NMR (101 MHz, $CDCl_3$) (Asterisks indicate peaks for the minor rotamer) δ 166.88, 154.39, 139.19 (x2), 138.77* (x2), 137.36, 130.31, 128.28 (x2), 127.24 (x2), 127.12, 123.63 (x2), 122.94* (x2), 109.51, 72.45, 58.85, 52.21, 47.65, 45.15, 41.67, 22.89; IR (KBr plates) 3343, 2945, 2109, 1714, 1634, 1515, 1395, 1260, 1029, 735 cm^{-1}; HRMS (ESI) *m/z* [M+Na]$^+$ calcd for $C_{21}H_{20}I_2N_2NaO_3$ 624.9461, found 624.9439.

9b: ^{1}H NMR (600 MHz, $CDCl_3$) (A mixture of rotamer in 75:25 ratio) δ 7.54 (1.5H, d, *J* = 7.2 Hz), 7.38–7.45 (0.5H, d, *J* = 9.6 Hz), 7.18–7.30 (3H, m), 6.18–6.24 (0.5H, m), 6.10 (1.5H, d, *J* = 9.3 Hz), 5.21–5.27 (0.5H, m), 5.18 (1.5H, d, *J* = 9.6 Hz) 4.81 (1.5H, s), 4.53 (0.5H, s), 3.68 (2.25H, s) 3.65–3.74 (0.5H, m) 3.60 (0.75H, s) 3.58–3.62 (1.5H, m), 2.25–2.32 (0.5H, m), 2.12–2.17 (1.5H, m), 1.95–1.99 (2H, m); ^{13}C NMR (150 MHz, $CDCl_3$) (Asterisks indicate peaks for the minor rotamer) δ 166.5, 154.3, 138.8, 137.7*, 136.9 (x2), 131.39, 129.5 (x2), 129.2*, 128.4*, 128.0 (x2), 127.5*, 126.9, 124.14 (x2), 123.7*, 109.7, 73.46, 59.10, 52.52*, 52.12, 48.81*, 47.53, 45.76*, 45.53, 42.97*, 40.97, 22.91, 22.29*; IR (KBr plates) 3004, 1694, 1443, 1367 cm^{-1}; HRMS (ESI) *m/z* [M+Na]$^+$ calcd for $C_{21}H_{20}I_2N_2NaO_3$ 624.9461, found 624.9452.

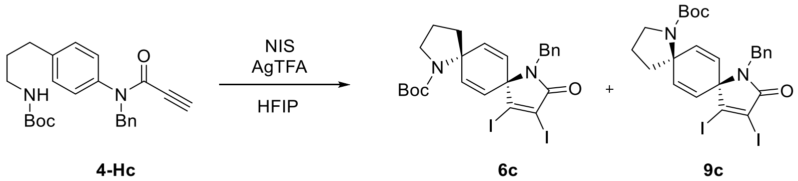

4-Hc **6c** **9c**

To a stirred solution of 60.0 mg (0.153 mmol) of **4-Hc** in 4.4 mL of HFIP were added 6.26 mg (0.031 mmol, 0.1 equiv) of AgTFA followed by 113 mg (0.504 mmol, 3.3 equiv) of NIS at 0 °C, then the stirring was continued at the same temperature for 24 h. The reaction mixture was quenched with

10% aqueous Na_2SO_3 and diluted with $CHCl_3$. After the organic layer separation, the aqueous layer was extracted with $CHCl_3$. The combined organic layers were dried over Na_2SO_4 and concentrated in vacuo. The crude product was chromatographed on silica gel with a gradient of 10–50% AcOEt/hexane to give 9.2 mg (0.014 mmol, 9.3% yield) of **9c** as a colorless solid followed by 71 mg (0.11 mmol, 72% yield) of **6c** as a colorless viscous oil.

6c: ^{1}H NMR (600 MHz, $CDCl_3$) δ 7.20–7.27 (5H, m), 6.14 (2H, d, *J* = 10.0 Hz), 5.19 (2H, d, *J* = 10.3 Hz), 4.47 (2H, s), 3.53 (2H, t, *J* = 6.5 Hz), 1.89–1.94 (4H, m), 1.43 (9H, s); ^{13}C NMR (150 MHz, $CDCl_3$) δ 167.07, 152.84, 139.04 (×2), 137.62, 130.90, 128.25 (×2), 127.51 (×2), 127.10, 123.10 (×2), 109.15, 79.33, 72.84, 58.62, 47.49, 45.26, 41.69, 28.54, 22.64 (×3); IR (neat) 2974, 1702, 1378, 1169, 1122, 727 cm^{-1}; HRMS (ESI) *m/z* $[M+Na]^+$ calcd for $C_{24}H_{26}I_2N_2NaO_3$ 666.9931, found 666.9939.

9c: ^{1}H NMR (400 MHz, $CDCl_3$) δ 7.59 (2H, dd, *J* = 7.3, 2.0 Hz), 7.17–7.23 (3H, m), 6.09 (2H, d, *J* = 10.0 Hz), 5.12 (2H, d, *J* = 9.8 Hz), 4.82 (2H, s), 3.55 (2H, t, *J* = 6.9 Hz), 2.11 (2H, t, *J* = 6.9 Hz), 1.90–1.96 (2H, m), 1.47 (9H, s); ^{13}C NMR (150 MHz, $CDCl_3$) δ 166.46, 153.01, 139.03 (×2), 137.39, 131.78, 129.68 (×2), 127.77 (×2), 126.81, 123.50 (×2), 109.44, 79.34, 73.52, 58.64, 47.66, 45.36, 40.97, 28.47, 22.70 (×3); IR (KBr plates) 2976, 2872, 1685, 1382, 1165, 1067, 935, 733 cm^{-1}; HRMS (ESI) *m/z* $[M+Na]^+$ calcd for $C_{24}H_{26}I_2N_2NaO_3$ 666.9931, found 666.9941.

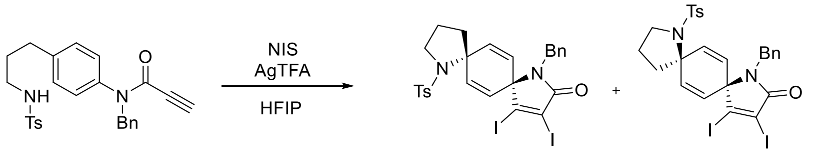

4-Hd **6d** **9d**

To a stirred solution of 18.0 mg (0.040 mmol) of **4-Hd** in 1.1 mL of HFIP were added 0.9 mg (0.004 mmol, 0.1 equiv) of AgTFA followed by 21.0 mg (0.089 mmol, 2.3 equiv) of NIS at 0 °C, then the stirring was continued at the same temperature for 24 h. The reaction mixture was quenched with 10% aqueous Na_2SO_3 and diluted with $CHCl_3$. After the organic layer separation, the aqueous layer was extracted with $CHCl_3$. The combined organic layers were dried over Na_2SO_4 and concentrated in vacuo. The crude product was chromatographed on silica gel with a gradient of 10–40% AcOEt/hexane to give 11.3 mg (0.016 mmol, 40% yield) of **9d** as a pale yellow oil solid followed by 14.1 mg (0.020 mmol, 50% yield) of **6d** as a colorless solid.

6d: ^{1}H NMR (400 MHz, $CDCl_3$) δ 7.84 (2H, d, *J* = 8.3 Hz), 7.18–7.28 (9H, m), 7.13–7.15 (2H, m), 6.06 (2H, d, *J* = 10.0 Hz), 5.22 (2H, d, *J* = 10.0 Hz), 4.43 (2H, s), 3.55 (2H, t, *J* = 6.7 Hz), 2.41 (3H, s), 1.96–2.00 (2H, m), 1.88–1.93 (2H, m); ^{13}C NMR (100 MHz, $CDCl_3$) δ 166.99, 143.27, 138.49 (×2), 137.39, 137.15, 130.28, 129.35 (×4), 128.32 (×2), 127.77 (×2), 127.18, 123.60 (×2), 109.70, 72.33, 61.92, 49.14, 45.10, 42.98, 22.98, 21.53; IR (KBr plates) 1697, 1345, 1157, 1095, 749, 666 cm^{-1}; HRMS (ESI) *m/z* $[M+H]^+$ calcd for $C_{26}H_{25}I_2N_2O_3S$ 698.9675, found 698.9687.

9d: ^{1}H NMR (600 MHz, $CDCl_3$) δ 7.45 (4H, m), 7.25–7.29 (3H, m), 7.11 (2H, d, *J* = 8.2 Hz), 6.10 (2H, d, *J* = 9.6 Hz), 5.24 (2H, d, *J* = 10.3 Hz), 4.86 (2H, s), 3.49 (2H, t, *J* = 6.5 Hz), 2.39 (3H, s), 2.18 (2H, t, *J* = 7.2 Hz), 1.92–1.96 (2H, m); ^{13}C NMR (150 MHz, $CDCl_3$) δ 166.75, 143.16, 138.24, 137.31, 136.99 (×2), 131.39, 129.37 (×2), 128.23 (×2), 128.11 (×2), 127.41 (×2), 126.87, 123.89 (×2), 109.92, 73.24, 62.24, 48.98, 45.41, 42.40, 23.17, 21.49; IR (neat) 3031, 1698, 1340, 1156, 1095, 751, 679 cm^{-1}; HRMS (ESI) *m/z* $[M+Na]^+$ calcd for $C_{26}H_{24}I_2N_2NaO_3S$ 720.9495, found 720.9496.

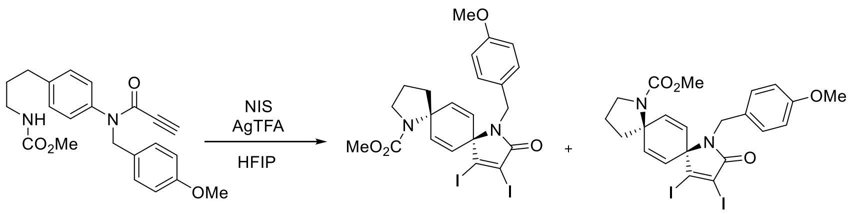

4-He 6e 9e

To a stirred solution of 98.0 mg (0.258 mmol) of **4-He** in 7.4 mL of HFIP were added 5.8 mg (0.026 mmol, 0.1 equiv) of AgTFA followed by 128 mg (0.567 mmol, 2.2 equiv) of NIS at 0 °C, then the stirring was continued at the same temperature for 24 h. The reaction mixture was quenched with 10% aqueous Na_2SO_3 and diluted with $CHCl_3$. After the organic layer separation, the aqueous layer was extracted with $CHCl_3$. The combined organic layers were dried over Na_2SO_4 and concentrated in vacuo. The crude product was chromatographed on silica gel with a gradient of 10–40% AcOEt/hexane to give 32.3 mg (0.052 mmol, 20% yield) of **9e** as a colorless viscous oil followed by112 mg (0.180 mmol, 69% yield) of **6e** as a colorless viscous oil.

6e: ^{1}H NMR (600 MHz, $CDCl_3$) δ 7.14 (2H, d, *J* = 8.2 Hz), 6.79 (2H, d, *J* = 8.9 Hz), 6.16 (2H, d, *J* = 10.0 Hz), 5.12–5.32 (2H, m), 4.40 (2H, s), 3.78 (3H, s), 3.60–3.72 (2H, m), 1.92–2.04 (2H, m), 1.92–1.97 (2H, m); ^{13}C NMR (150 MHz, 0 °C, $CDCl_3$) (Asterisks indicate peaks for the minor rotamer) δ 166.95, 158.51, 155.48*, 154.50, 139.25* (×2), 138.65 (×2), 130.23, 129.57, 129.34*, 128.86 (×2), 128.71*, 123.76 (×2), 122.87* (×2), 113.55 (×2), 109.49, 72.47, 72.34*, 58.99, 58.71*, 55.20, 53.11, 52.25, 48.48*, 47.60, 44.64, 43.19*, 41.70, 23.07, 22.40*; IR (neat) 2977, 1687, 1512, 1448, 1380, 1246, 753 cm^{-1}; HRMS (ESI) $[M+H]^+$ calcd for $C_{22}H_{23}I_2N_2O_4$ 632.9747, found 632.9731.

9e: ^{1}H NMR (600 MHz, $CDCl_3$) (A mixture of rotamers in 75:25 ratio) δ 7.51 (1.5H, d, *J* = 7.9 Hz), 7.36–7.42 (0.5H, m), 6.76–6.79 (2H, m), 6.18–6.24 (0.5H, m), 6.11 (1.5H, d, *J* = 9.6 Hz), 5.19–5.24 (0.5H, m), 5.18 (1.5H, d, *J* = 9.6 Hz) 4.74 (1.5H, s), 4.47 (0.5H, s), 3.77 (3H, s), 3.67 (2.25H, s), 3.61 (0.75H, s), 3.56–3.76 (2H, m) 2.26–2.34 (0.5H, m), 2.16 (1.5H, t, *J* = 6.9 Hz), 1.95–2.00 (2H, m); ^{13}C NMR (100 MHz, $CDCl_3$) (Asterisks indicate peaks for the minor rotamer) δ 166.40, 158.55, 154.33, 137.56* (×2), 136.83 (×2), 131.23, 131.08 (×2), 130.92, 124.25 (×2), 123.81* (×2), 113.69* (×2), 113.27 (×2), 109.88, 73.45, 59.15, 55.20, 52.19, 47.60, 44.99, 41.05, 22.97; IR (neat) 2977,

1696, 1513, 1445, 1373, 1247, 1180, 751 cm^{-1}; HRMS (ESI) *m*/*z* [M+H]$^+$ calcd for $C_{22}H_{23}I_2N_2O_4$ 632.9747, found 632.9731.

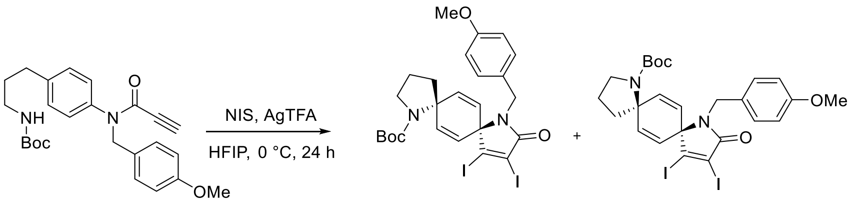

4-Hf **6f** **9f**

To a stirred solution of 31.9 mg (0.076 mmol) of **4-Hj** in 2.2 mL of HFIP were added 1.6 mg (0.008 mmol, 0.1 equiv) of AgTFA followed by 37.4 mg (0.166 mmol, 2.2 equiv) of NIS at 0 °C, then the stirring was continued at the same temperature for 24 h. The reaction mixture was quenched with 10% aqueous Na_2SO_3 and diluted with $CHCl_3$. After the organic layer separation, the aqueous layer was extracted with $CHCl_3$. The combined organic layers were dried over Na_2SO_4 and concentrated in vacuo. The crude product was chromatographed on silica gel with a gradient of 10–40% AcOEt/hexane to give 11.0 mg (0.016 mmol, 22% yield) of **9f** as a colorless solid followed by 33.5 mg (0.050 mmol, 66% yield) of **6f** as a colorless solid.

6f: ^{1}H NMR (600 MHz, $CDCl_3$) δ 7.16 (2H, d, *J* = 8.6 Hz), 6.79 (2H, d, *J* = 8.6 Hz), 6.16 (2H, d, *J* = 10.0 Hz), 5.19 (2H, d, *J* = 10.0 Hz), 4.39 (2H, s), 3.78 (3H, s), 3.54 (2H, t, *J* = 6.7 Hz), 1.91–1.98 (4H, m), 1.43 (9H, s); ^{13}C NMR (150 MHz, $CDCl_3$) δ 167.01, 158.66, 152.87, 138.97 (×2), 130.69, 129.84, 129.09 (×2), 123.26 (×2), 113.60 (×2), 109.36, 79.36, 72.84, 58.67, 55.22, 47.53, 44.79, 41.83, 28.57, 22.70 (×3); IR (KBr plates) 2969, 1686, 1510, 1380, 1243, 1164, 774 cm^{-1}; HRMS (ESI) *m*/*z* [M+Na]$^+$ calcd for $C_{25}H_{28}I_2N_2NaO_4$ 697.0036, found 697.0063.

9f: ^{1}H NMR (600 MHz, $CDCl_3$) δ 7.55 (2H, d, *J* = 8.6 Hz), 6.73 (2H, d, *J* = 8.6 Hz), 6.10 (2H, d, *J* = 10.0 Hz), 5.11 (2H, d, *J* = 10.0 Hz), 4.75 (2H, s), 3.76 (3H, s), 3.55 (2H, t, *J* = 6.9 Hz), 2.11 (2H, t, *J* = 6.9 Hz), 1.91–1.96 (2H, m), 1.47 (9H, s); ^{13}C NMR (150 MHz, $CDCl_3$) δ 166.39, 158.48, 153.08, 137.30 (×2), 131.46, 131.28 (×2), 131.19, 123.67 (×2), 113.07 (×2), 109.62, 79.38, 73.50, 58.69, 55.15, 47.70, 44.81, 41.03, 28.49, 22.74 (×3); IR (KBr plates) 2974, 1695, 1513, 1381, 1247, 1168, 751 cm^{-1}; HRMS (ESI) *m*/*z* [M+Na]$^+$ calcd for $C_{25}H_{28}I_2N_2NaO_4$ 697.0036, found 697.0062.

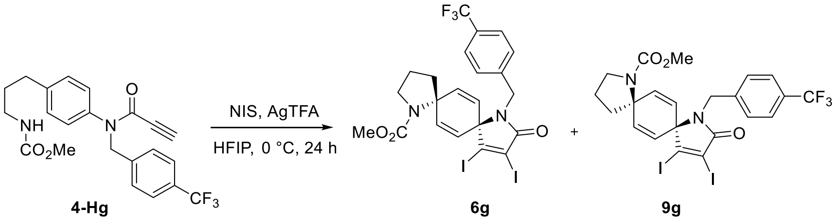

4-Hg **6g** **9g**

To a stirred solution of 22.0 mg (0.053 mmol) of **4-Hg** in 1.5 mL of HFIP were added 1.1 mg (0.005 mmol, 0.1 equiv) of AgTFA followed by 26.0 mg (0.116 mmol, 2.2 equiv) of NIS at 0 °C, then the

stirring was continued at the same temperature for 24 h. The reaction mixture was quenched with 10% aqueous Na_2SO_3 and diluted with $CHCl_3$. After the organic layer separation, the aqueous layer was extracted with $CHCl_3$. The combined organic layers were dried over Na_2SO_4 and concentrated in vacuo. The crude product was chromatographed on silica gel with a gradient of 10–40% AcOEt/hexane to give 3.3 mg (0.005 mmol, 9.4% yield) of **9g** as a colorless viscous oil followed by 23.8 mg (0.036 mmol, 68% yield) of **6g** as a colorless viscous oil.

6g: ^{1}H NMR (600 MHz, $CDCl_3$) δ 7.53 (2H, d, *J* = 7.9 Hz), 7.31 (2H, d, *J* = 7.9 Hz), 6.18 (2H, d, *J* = 10.0 Hz), 5.24–5.24 (2H, m), 4.51 (2H, s), 3.69 (3H, s), 3.57–3.67 (2H, m) 1.93–1.95 (4H, m); ^{13}C NMR (150 MHz, $CDCl_3$) (Asterisks indicate peaks for the minor rotamer) δ 167.01, 155.26*, 154.38, 141.46, 139.67* (×2), 139.22 (×2), 130.69, 129.50 (q, *J* = 31.7 Hz), 127.47 (×2), 125.34 (q, *J* = 3.0 Hz) (×2), 123.99 (q, *J* = 270 Hz), 123.44 (×2), 122.70* (×2), 109.00, 72.50, 58.81, 53.00*, 52.28, 48.41*, 47.61, 44.79, 43.09*, 41.72, 23.02; IR (neat) 3004, 1691, 1448, 1380, 1326, 1164, 1125, 1066, 930, 755 cm^{-1}; HRMS (ESI) *m*/*z* $[M+H]^+$ calcd for $C_{22}H_{20}F_3I_2N_2O_3$ 670.9515, found 670.9501.

9g: ^{1}H NMR (400 MHz, $CDCl_3$) δ 7.67 (2H, d, *J* = 8.0 Hz), 7.48 (2H, d, *J* = 8.0 Hz), 6.11 (2H, d, *J* = 10.0 Hz), 5.15 (2H, d, *J* = 9.8 Hz), 4.85 (2H, s), 3.68 (3H, s), 3.60 (2H, t, *J* = 6.8 Hz), 2.15 (2H, t, *J* = 6.8 Hz), 1.94–2.01 (2H, m); ^{13}C NMR (150 MHz, $CDCl_3$) δ 166.63, 154.36, 142.78, 137.19 (×2), 131.73, 129.71 (×2), 129.12 (q, *J* = 31.7 Hz), 124.92 (×2), 124.28 (q, *J* = 270 Hz) 123.92 (×2), 109.33, 73.43, 59.09, 52.16, 47.59, 45.02, 40.94, 22.94; IR (neat) 2954, 1698, 1561, 1446, 1375, 1326, 1187, 1124, 750 cm^{-1}; HRMS (ESI) *m*/*z* $[M+H]^+$ calcd for $C_{22}H_{20}F_3I_2N_2O_3$ 670.9515, found 670.9501.

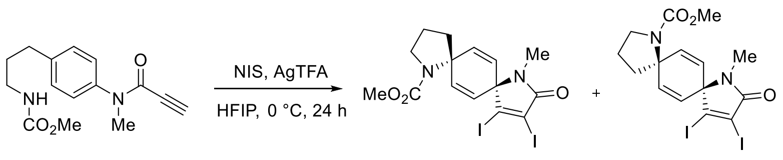

4-Hh **6h** **9h**

To a stirred solution of 10.0 mg (0.036 mmol) of **4-Hh** in 1.0 mL of HFIP were added 0.81 mg (0.004 mmol, 0.1 equiv) of AgTFA followed by 18.0 mg (0.080 mmol, 2.2 equiv) of NIS at 0 °C, then the stirring was continued at the same temperature for 24 h. The reaction mixture was quenched with 10% aqueous Na_2SO_3 and diluted with $CHCl_3$. After the organic layer separation, the aqueous layer was extracted with $CHCl_3$. The combined organic layers were dried over Na_2SO_4 and concentrated in vacuo. The crude product was chromatographed on silica gel with a gradient of 15–100% AcOEt/hexane to give 7.0 mg (0.013 mmol, 40% yield) of **9h** as a colorless solid followed by 9.5 mg (0.018 mmol, 50% yield) of **6h** as a colorless solid.

6h: ^{1}H NMR (600 MHz, $CDCl_3$) δ 6.28 (2H, d, *J* = 10.0 Hz), 5.26 (2H, s), 3.71 (3H, s) 3.56–3.74 (2H, m), 2.84 (3H, s), 2.01–2.08 (2H, m), 1.95–1.99 (2H, m); ^{13}C NMR (150 MHz, $CDCl_3$)

(Asterisks indicate peaks for the minor rotamer) δ 166.06, 155.47, 154.50*, 139.75 (×2), 139.16* (×2), 129.05*, 128.76, 123.14* (×2), 122.30 (×2), 109.94, 109.34*, 71.77, 59.02, 58.91*, 52.95, 52.23*, 48.44, 47.62*, 43.03, 41.69*, 29.55*, 27.16, 23.07*, 22.50; IR (KBr plates) 2967, 2875, 1663, 1561, 1452, 1383, 966, 745 cm^{-1}; HRMS (ESI) *m/z* $[M+H]^+$ calcd for $C_{15}H_{17}I_2N_2O_3$ 526.9329, found 526.9346.

9h: ^1H NMR (600 MHz, $CDCl_3$) (A mixture of rotamers in 70:30 ratio) δ 6.15 (2H, d, *J* = 9.3 Hz), 5.25 (1.4H, d, *J* = 9.6 Hz), 5.15–5.22 (0.6H, m), 3.57–3.70 (2H, m), 3.66 (2.1H, s), 3.60 (0.9H, s) 3.11 (2.1H, s) 2.98 (0.9H, s) 2.23–2.28 (0.6H, m), 2.15–2.19 (1.4H, m), 1.95–2.01 (2H, m); ^{13}C NMR (150 MHz, $CDCl_3$) (Asterisks indicate peaks for the minor rotamer) δ 166.23, 154.45, 138.08* (×2), 137.20 (×2), 130.46, 123.79 (×2), 123.05* (×2), 109.64, 72.73, 59.13, 52.14, 48.63*, 47.65, 42.62*, 41.08, 28.39, 22.93, 22.36*; IR (KBr plates) 2971, 2954, 2868, 1685, 1559, 1450, 1377 cm^{-1}; HRMS (ESI) *m/z* $[M+H]^+$ calcd for $C_{15}H_{17}I_2N_2O_3$ 526.9329, found 526.9346.

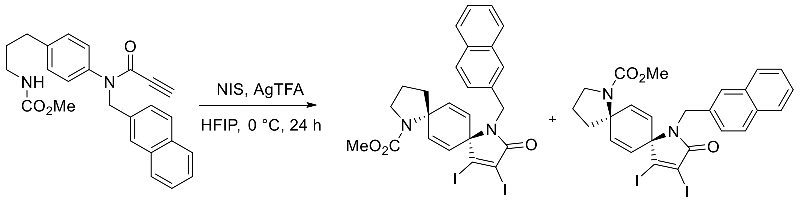

4-Hi **6i** **9i**

To a stirred solution of 20.0 mg (0.050 mmol) of **4-Hi** in 0.70 mL of HFIP were added 1.1 mg (0.005 mmol, 0.1 equiv) of AgTFA followed by 28.0 mg (0.12 mmol, 2.5 equiv) of NIS at 0 °C, then the stirring was continued at the same temperature for 24 h. The reaction mixture was quenched with 10% aqueous Na_2SO_3 and diluted with $CHCl_3$. After the organic layer separation, the aqueous layer was extracted with $CHCl_3$. The combined organic layers were dried over Na_2SO_4 and concentrated in vacuo. The crude product was chromatographed on silica gel with a gradient of 10–40% AcOEt/hexane give 3.6 mg (0.006 mmol, 11% yield) of **9i** as a colorless viscous oil followed by 28.0 mg (0.043 mmol, 86% yield) of **6i** as a colorless viscous oil.

6i: ^1H NMR (600 MHz, $CDCl_3$) δ 7.74–7.80 (3H, m), 7.60 (1H, s), 7.42–7.46 (2H, m), 7.32 (1H, d, *J* = 7.9 Hz), 6.11 (2H, d, *J* = 8.2 Hz), 5.15–5.33 (2H, m), 4.65 (2H, s), 3.67 (3H, s) 3.52–3.73 (2H, m), 1.96–2.08 (2H, m), 1.86–1.95 (2H, m); ^{13}C NMR (150 MHz, $CDCl_3$) (Asterisks indicate peaks for the minor rotamer) δ 166.97, 155.47*, 154.47, 139.25* (×2), 138.83 (×2), 134.94, 133.07, 132.51, 130.50, 128.13, 127.67, 127.57, 126.11, 125.87, 125.77, 125.30, 123.64 (×2), 122.89* (×2), 109.36, 72.51, 58.88, 52.91*, 52.19, 48.33*, 47.58, 45.27, 43.10*, 41.67, 22.94; IR (neat) 3001, 1688, 1448, 1377, 750 cm^{-1}; HRMS (ESI) *m/z* $[M+Na]^+$ calcd for $C_{25}H_{22}I_2N_2NaO_3$ 674.9618, found 674.9643.

9i: ^1H NMR (600 MHz, $CDCl_3$) (A mixture of rotamers in 80:20 ratio) δ 8.02–8.04 (0.8H, s), 7.68–7.80 (4H, m), 7.41–7.44 (2.2H, m), 6.22–6.25 (0.4H, m), 6.09 (1.6H, d, *J* = 9.6 Hz), 5.28–5.30 (0.4H, m), 5.17 (1.6H, d, *J* = 9.6 Hz), 4.97 (1.6H, s), 4.70 (0.4H, s), 3.75 (2.4H, s), 3.72–3.76 (0.4H, m)

3.61 (0.6H, s), 3.58–3.64 (1.6H, m) 2.28–2.32 (0.4H, m), 2.15 (1.6H, t, *J* = 6.9 Hz), 1.94–1.99 (2H, m); ^{13}C NMR (150 MHz, $CDCl_3$) δ 166.60, 154.32, 136.94 (×2), 136.44, 133.18, 132.54, 131.59, 128.42, 128.34, 128.04, 127.59, 127.53, 125.56, 125.51, 124.15 (×2), 109.67, 73.49, 59.15, 52.19, 47.53, 45.63, 40.99, 22.92; IR (neat) 2980, 1697, 1445, 1373, 1215, 750 cm^{-1}; HRMS (ESI) *m*/*z* [M+Na]$^+$ calcd for $C_{25}H_{22}I_2N_2NaO_3$ 674.9618, found 674.9638.

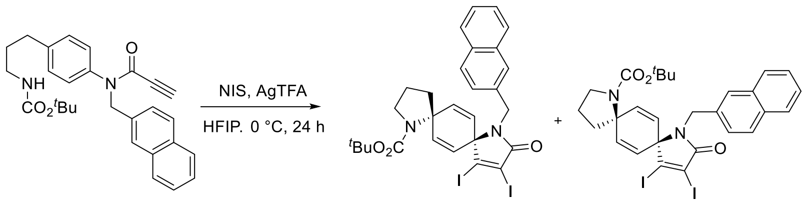

4-Hj 6j 9j

To a stirred solution of 25.0 mg (0.057 mmol) of **4-Hj** in 1.6 mL of HFIP were added 1.3 mg (0.006 mmol, 0.1 equiv) of AgTFA followed by 31.8 mg (0.141 mmol, 2.5 equiv) of NIS at 0 °C, then the stirring was continued at the same temperature for 24 h. The reaction mixture was quenched with 10% aqueous Na_2SO_3 and diluted with $CHCl_3$. After the organic layer separation, the aqueous layer was extracted with $CHCl_3$. The combined organic layers were dried over Na_2SO_4 and concentrated in vacuo. The crude product was chromatographed on silica gel with a gradient of 10–60% AcOEt/hexane as eluent to give 3.9 mg (0.006 mmol, 10% yield) of **9j** as a colorless solid followed by 30.2 mg (0.046 mmol, 82% yield) of **6j** as a colorless solid.

6j: ^{1}H NMR (600 MHz, $CDCl_3$) δ 7.74–7.80 (3H, m), 7.62 (1H, s), 7.43–7.47 (2H, m), 7.35 (1H, dd, *J* = 8.2, 1.4 Hz), 6.12 (2H, d, *J* = 10.3 Hz), 5.20 (2H, d, *J* = 10.3 Hz), 4.65 (2H, s), 3.52 (2H, t, *J* = 6.9 Hz), 1.96 (2H, t, *J* = 6.5 Hz), 1.86–1.91 (2H, m), 1.42 (9H, s); ^{13}C NMR (150 MHz, $CDCl_3$) δ 167.13, 152.77, 139.06 (×2), 135.16, 133.06, 132.50, 131.07, 128.05, 127.66, 127.55, 126.10, 126.03, 125.71, 125.55, 123.04 (×2), 109.09, 79.26, 72.85, 58.59, 47.43, 45.34, 41.65, 28.50, 22.60 (×3); IR (KBr plates) 2981, 1686, 1378, 1164 cm^{-1}; HRMS (ESI) *m*/*z* [M+Na]$^+$ calcd for $C_{28}H_{28}I_2N_2NaO_3$ 717.0087, found 717.0092.

9j: ^{1}H NMR (600 MHz, $CDCl_3$) δ 8.03 (1H, s), 7.73–7.78 (3H, m), 7.69 (1H, d, *J* = 8.2 Hz), 7.39–7.42 (2H, m), 6.08 (2H, d, *J* = 10.0 Hz), 5.11 (2H, d, *J* = 10.0 Hz), 4.99 (2H, s), 3.56 (2H, t, *J* = 6.9 Hz), 2.11 (2H, t, *J* = 7.0 Hz), 1.90–1.95 (2H, m), 1.53 (9H, s); ^{13}C NMR (150 MHz, $CDCl_3$) δ 166.60, 153.08, 137.46 (×2), 136.78, 133.20, 132.53, 131.86, 128.35, 127.98 (×2), 127.50, 127.40, 125.52, 125.45, 123.61 (×2), 109.50, 79.39, 73.62, 58.69, 47.77, 45.52, 41.08, 28.62, 22.76 (×3); IR (KBr plates) 2975, 1695, 1561, 1380, 1168, 957, 749 cm^{-1}; HRMS (ESI) *m*/*z* [M+Na]$^+$ calcd for $C_{28}H_{28}I_2N_2NaO_3$ 717.0087, found 717.0092.

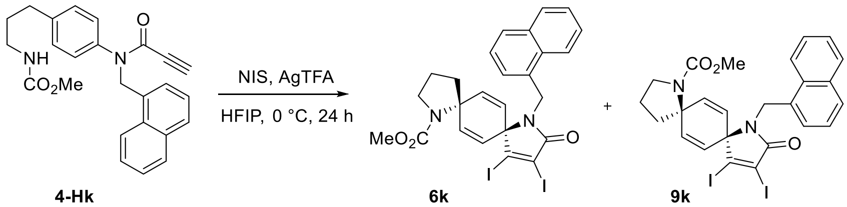

4-Hk **6k** **9k**

To a stirred solution of 0.215 g (0.537 mmol) of **4-Hk** in 13.0 mL of HFIP were added slowly 11.0 mg (0.054 mmol, 0.1 equiv) of AgTFA followed by 0.302 g (0.134 mmol, 2.5 equiv) of NIS at 0 °C, then the stirring was continued at the same temperature for 24 h. The reaction mixture was quenched with 10% aqueous Na_2SO_3 and diluted with $CHCl_3$. After the organic layer separation, the aqueous layer was extracted with $CHCl_3$. The combined organic layers were dried over Na_2SO_4 and concentrated in vacuo. The crude product was chromatographed on silica gel with a gradient of 7–50% AcOEt/hexane to give 31.3 mg (0.048 mmol, 9% yield) of **9k** as a colorless solid followed by 0.285 g (0.437 mmol, 81% yield) of **6k** as a colorless viscous oil.

6k: ^{1}H NMR (600 MHz, $CDCl_3$) δ 7.86 (2H, dd, *J* = 8.4, 6.7 Hz), 7.74 (1H, d, *J* = 8.2 Hz), 7.47–7.52 (2H, m), 7.40 (1H, t, *J* = 7.7 Hz), 7.17 (1H, d, *J* = 6.9 Hz), 6.08 (2H, d, *J* = 10.0 Hz), 5.31–5.40 (2H, m), 4.98 (2H, s), 3.67 (3H, s), 3.45–3.54 (2H, m), 1.62–1.68 (2H, m), 1.53–1.58 (2H, m); ^{13}C NMR (150 MHz, $CDCl_3$) (Asterisks indicate peaks for the minor rotamer) δ 167.08, 155.40, 154.47*, 139.51* (×2), 139.09 (×2), 133.48, 131.48, 130.76, 130.18, 128.93, 127.44, 126.08, 125.56, 125.29, 123.35 (×2), 123.08, 122.60* (×2), 121.86, 109.54*, 109.10, 72.67, 58.81, 52.89, 52.22*, 48.35*, 47.57, 43.06*, 42.80, 41.76, 22.85, 22.38*; IR (neat) 3008, 1690, 1448, 1376, 752 cm^{-1}; HRMS (ESI) *m*/*z* $[M+Na]^+$ calcd for $C_{25}H_{22}I_2N_2NaO_3$ 674.9618, found 674.9641.

9k: ^{1}H NMR (600 MHz, $CDCl_3$) (A mixture of rotamers in 80:20 ratio) δ 8.21–8.24 (0.8H, m), 8.08–8.12 (0.2H, m), 7.80 (1H, d, *J* = 6.9 Hz), 7.70 (1H, d, *J* = 7.6 Hz) 7.44 (2H, t, *J* = 7.2 Hz), 7.37 (1H, t, *J* = 6.5 Hz) 7.30–7.34 (0.2H, m) 7.26–7.28 (0.8H, m), 6.15–6.21 (0.4H, m), 6.06 (1.6H, d, *J* = 9.3 Hz), 5.42 (1.6H, s) 5.40–5.45 (0.4H, m) 5.38 (1.6H, d, *J* = 9.3 Hz) 5.06 (0.4H, s), 3.62–3.65 (0.4H, m), 3.44–3.52 (1.6H, m) 3.47 (2.4H, s), 3.04 (0.6H, s), 2.26–2.33 (0.4H, m), 2.08–2.13 (1.6H, m), 1.89–1.96 (1.6H, m), 1.62–1.72 (0.4H, m); ^{13}C NMR (150 MHz, $CDCl_3$) δ 166.71, 154.02, 137.01, 133.61, 132.82, 132.16, 130.75 (×2), 128.17, 127.11, 125.30, 125.17, 124.31, 123.83 (×2), 123.44, 123.24, 109.42, 73.67, 58.95, 51.88, 47.17, 43.55, 40.59, 22.79; IR (KBr plates) 3005, 1696, 1445, 1373, 749 cm^{-1}; HRMS (ESI) *m*/*z* $[M+Na]^+$ calcd for $C_{25}H_{22}I_2N_2NaO_3$ 674.9618, found 674.9610.

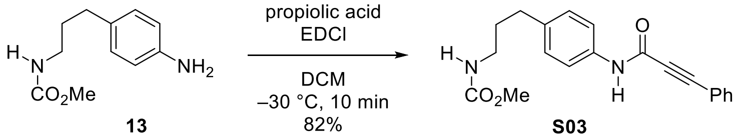

13 **S03**

To a stirred solution of 1.05 g (5.05 mmol) of **13** in 15 mL of CH_2Cl_2 were added 0.738 g (5.05 mmol, 1.0 equiv) of phenylpropiolic acid and 0.968 g (5.05 mmol, 1.0 equiv) of EDC at 0 °C, and the stirring was continued at the same temperature for 10 min. The reaction was diluted with water. After the organic layer separation, the aqueous layer was extracted with $CHCl_3$. The combined organic layers were dried over Na_2SO_4 and concentrated in vacuo. The crude product was chromatographed on silica gel eluting with a gradient of 2–9% MeOH/$CHCl_3$ to give 1.70 g (4.39 mmol, 87% yield) of **S03** as a pale yellow solid.

S03: ^{1}H NMR (400 MHz, $CDCl_3$) δ 7.57–7.60 (3H, m), 7.36–7.49 (5H, m), 7.16 (2H, d, *J* = 8.3 Hz), 4.68 (1H, s), 3.67 (3H, s), 3.21 (2H, q, *J* = 6.6 Hz), 2.62 (2H, t, *J* = 7.7 Hz), 1.78–1.85 (2H, m); ^{13}C NMR (100 MHz, $CDCl_3$) δ 157.11, 150.98, 138.04, 135.35, 132.54, 130.21, 128.87 (×2), 128.52 (×2), 120.14 (×2), 119.96 (×2), 85.56, 83.47, 52.06, 40.52, 32.38, 31.55; IR (KBr plates) 3393, 3289, 2215, 1701, 1654 cm^{-1}; HRMS (ESI) *m*/*z* $[M+Na]^+$ calcd for $C_{20}H_{20}N_2NaO_3$ 359.1372, found 359.1345.

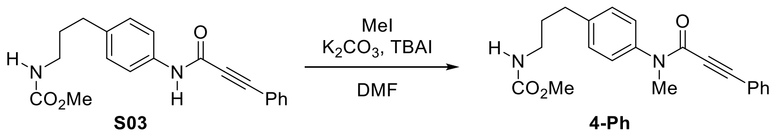

S03 **4-Ph**

To a stirred solution of 12.2 g (36.3 mmol) of **S03** in 145 mL of DMF were added 15.0 g (109 mmol, 3 equiv) and 14.8 mL (145 mmol, 4 equiv) of MeI at rt, and the stirring was continued at the same temperature for 23 h. The reaction mixture was quenched with saturated aqueous NH_4Cl, and extracted with AcOEt. The combined organic layers were washed with water and brine, and dried over Na_2SO_4, and concentrated in vacuo. The crude product was chromatographed on silica gel with a gradient of 20–60% AcOEt/hexane to give 7.80 g (22.1 mmol, 61%) of **4-Ph** as a yellow solid.

4-Ph: ^{1}H-NMR (400 MHz, $CDCl_3$) (A mixture of rotamers in 85:15 ratio) δ 7.60 (0.3 H, d, *J* = 7.0 Hz), 7.40 (0.3H, d, *J* = 7.8 Hz), 7.30–7.34 (1H, m), 7.22–7.29 (5.4H, m), 7.12–7.15 (2H, m), 4.70 (0.85H, s), 4.54 (0.15H, s), 3.67 (3H, s), 3.65 (0.45H, s), 3.37 (2.55H, s), 3.23 (2H, q, *J* = 6.4 Hz), 2.70 (1.7H, t, *J* = 7.8 Hz), 2.65 (0.3H, t, *J* = 7.9 Hz), 1.82–1.90 (2H, m); ^{13}C NMR (100 MHz, $CDCl_3$) δ 157.03, 154.27, 141.33, 141.04, 132.27, 129.83, 128.96 (×2), 128.21 (×2), 127.27 (×2), 120.36 (×2), 90.69, 82.54, 51.98, 40.42, 36.29, 32.49, 31.63; IR (KBr plates) 3337, 2943, 2217, 1721, 1635, 1513 cm^{-1}; HRMS (ESI) *m*/*z* $[M+Na]^+$ calcd for $C_{21}H_{22}N_2NaO_3$ 373.1528, found 373.1520.

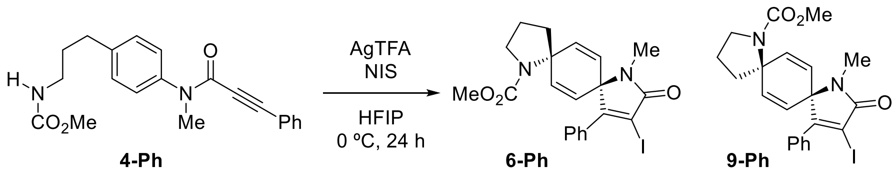

4-Ph **6-Ph** **9-Ph**

To a stirred solution of 50.0 mg (0.140 mmol) of **4-Ph** in 4.0 mL of HFIP were added 2.9 mg (0.014 mmol, 0.1 equiv) of AgTFA and 35.0 mg (0.16 mmo, 1.1 equiv) of NIS at 0 °C, and the stirring was continued at the same temperature for 24 h. The reaction mixture was quenched with 10% aqueous Na_2SO_3, and extracted with $CHCl_3$, dried over Na_2SO_4, and concentrated in vacuo. The crude product was purified on PTLC with 25% $CHCl_3$/hexane to give 38.0 mg (0.080 mmol, 56% yield) of **9-Ph** as a yellow solid followed by 20.0 mg (0.042 mmol, 29% yield) of **6-Ph** as a colorless solid.

6-Ph: ^{1}H NMR (600 MHz, $CDCl_3$) (A mixture of rotamers in 65:35 ratio) δ 7.92 (0.7H, s), 7.25–7.35 (3H, m), 7.27 (1H, d, *J* = 16.5 Hz), 6.17 (2H, s), 5.45 (2H, d, *J* = 8.6 Hz), 3.59 (3H, s), 2.84 (3H, s), 2.72 (1H, s), 2.04 (2H, t, *J* = 6.9 Hz), 1.92–1.97 (2H, m); ^{13}C NMR (150 MHz, –10 °C, $CDCl_3$) (Asterisks indicate peaks for the minor rotamer) δ167.27*, 166.73, 163.96, 162.02*, 155.34, 154.46*, 138.64(x 2), 137.71* (x 2), 134.08, 131.65*, 130.01*, 129.07, 128.93* (x 2), 128.34 (x 2), 128.15 (x 2), 128.05*, (x 2), 123.74* (x2), 121.84, (x 2), 98.10, 94.44*, 68.71, 68.55*, 59.19*, 58.65, 51.96*, 51.64, 48.08, 47.33*, 42.45, 41.36*, 26.05, 25.95*, 23.01*, 22.34167.03, 164.04, 155.32, 138.71 (×2), 137.83* (×2), 130.00, 129.09, 128.56 (×2), 128.30 (×2), 128.00, 123.95* (×2), 122.14 (×2), 68.78, 58.85, 51.70, 48.11, 47.62*, 42.64, 41.49*, 29.67*, 26.00, 23.00*, 22.45; IR (KBr plates) 2966, 1719, 1382, 1025, 749 cm^{-1}; HRMS (ESI) *m*/*z* [M+H]$^+$ calcd for $C_{21}H_{22}IN_2O_3$ 477.0675, found 477.0691.

9-Ph: ^{1}H NMR (600 MHz, $CDCl_3$) (A mixture of rotamers in 65:35 ratio) δ 7.36 (3H, q, *J* = 4.8 Hz), 7.15–7.20 (2H, m), 5.98 (2H, d, *J* = 10.0 Hz), 5.43 (1.3H, d, *J* = 9.3 Hz), 5.35 (0.7H, d, *J* = 9.3 Hz), 3.63 (2H, s), 3.50–3.55 (0.6H, s), 3.47 (1.3H, t, *J* = 6.4 Hz), 3.11 (2H, s), 2.98 (1H, s), 1.68–1.73 (2H, m), 1.16 (1.3H, t, *J* = 6.5 Hz), 1.09–1.14 (0.7H, s); ^{13}C NMR (150 MHz, $CDCl_3$) (Asterisks indicate peaks for the minor rotamer) δ 166.96, 163.28, 154.39, 137.34* (×2), 136.51 (×2), 133.69, 129.02, 128.25 (×2), 127.95 (×2), 123.40 (×2), 122.57* (×2), 96.69, 69.91, 59.09, 52.02, 48.32, 47.44, 41.53*, 40.28, 27.27, 22.68, 22.10*; IR (KBr plates) 2967, 1699, 1442, 1368, 1127, 750 cm^{-1}; HRMS (ESI) *m*/*z* [M+H]$^+$ calcd for $C_{21}H_{22}IN_2O_3$ 477.0675, found 477.0672.

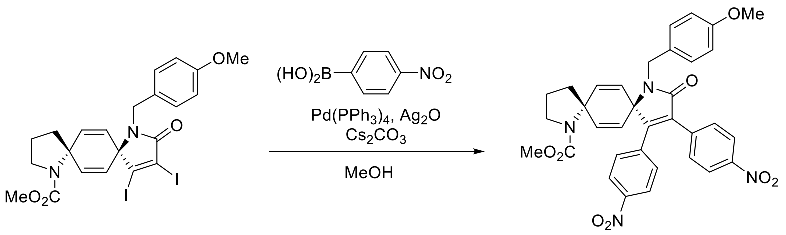

6e **10e-1**

In a dried flask were placed 20.0 mg (0.032 mmol) of **6e**, 3.7 mg (3.2 μmol, 0.1 equiv) of $Pd(PPh_3)_4$, 22.0 mg (0.095 mmol, 3 equiv) of Ag_2O, 9.60 mg (0.063 mmol, 2 equiv) of CsF, and 13.0 mg (0.079 mmol, 2.5 equiv) of (4-nitrophenyl)boronic acid. To the flask was added 0.50 mL of degassed MeOH at rt, and the reaction mixture was stirred for 2 h at rt. The reaction mixture was filtered

through a pad of Celite and rinsed with AcOEt, and concentrated in vacuo. The crude product was chromatographed on silica gel with a gradient of 10–60% AcOEt/hexane to give 15.4 mg (0.025 mmol, 78% yield) of **10e-1** as a pale orange solid.

10e-1: ^{1}H NMR (400 MHz, $CDCl_3$) δ 8.14 (2H, d, *J* = 8.8 Hz), 8.12 (2H, d, *J* = 8.8 Hz), 7.52–7.70 (4H, m), 7.25 (2H, d, *J* = 8.8 Hz), 6.84 (2H, d, *J* = 8.8 Hz), 6.15 (2H, d, *J* = 10.0 Hz), 5.53 (2H, d, *J* = 10.0 Hz), 4.44 (2H, s), 3.79 (3H, s), 3.55–3.61 (5H, m), 2.07 (2H, t, *J* = 6.7 Hz), 1.93-1.99 (2H, m); ^{13}C NMR (100 MHz, $CDCl_3$) δ 168.39, 158.68, 155.56, 154.27, 148.10, 147.53, 138.52, 137.92, 137.28, 132.94 (×2), 130.85 (×2), 130.78 (×2), 129.75 (×2), 128.92 (×2), 124.09 (×2), 123.67 (×2), 123.49 (×2), 113.71, 66.20, 59.11, 55.22, 51.97, 47.38, 43.18, 41.77, 23.01; IR (KBr plates) 3001, 2954, 1689, 1600, 1515, 1445, 1374, 1347, 1247, 753 cm^{-1}; HRMS (ESI) *m/z* $[M+H]^+$ calcd for $C_{34}H_{31}N_4O_8$ 623.2142, found 623.2157.

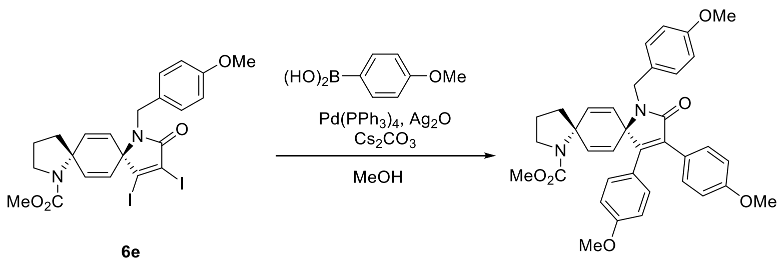

To a dried flask were placed 20.0 mg (0.032 mmol) of **6e**, 3.7 mg (3.2 μmol, 0.1 equiv) of $Pd(PPh_3)_4$, 22.0 mg (0.095 mmol, 3 equiv) of Ag_2O, 9.60 mg (0.063 mmol, 2 equiv) of CsF, and 19.0 mg (0.130 mmol, 4 equiv) of (4-methoxyphenyl)boronic acid. To the flask was added 0.50 mL of degassed MeOH at rt, and the reaction mixture was stirred for 30 min at rt. The reaction mixture was filtered through a pad of Celite and rinsed with $CHCl_3$, and concentrated in vacuo. The crude product was chromatographed on silica gel with ith a gradient of 10–60% AcOEt/hexane to give 11.7 mg (0.020 mmol, 62% yield) of **10e-2** as a pale yellow solid.

10e-2: ^{1}H NMR (400 MHz, $CDCl_3$) δ 7.30–7.52 (3H, m), 7.11–7.28 (5H, m), 6.76–6.84 (7H, m), 6.08 (2H, s), 5.54 (2H, d, *J* = 8.8 Hz), 4.41 (2H, s), 3.78 (7H, s), 3.76 (4H, s), 3.61 (4H, m), 2.07 (2H, t, *J* = 6.8 Hz), 1.89–1.96 (2H, m); ^{13}C NMR (150 MHz, $CDCl_3$) (Asterisk indicates peak for the minor rotamer) δ 170.74, 159.68, 159.16, 158.36, 154.49, 153.70, 137.34 (×3), 136.96 (×2), 131.45 (×4), 131.15 (×4), 130.70, 128.67 (×2), 126.10 (×2), 124.61* (×2), 113.54 (×2), 65.62, 59.38, 55.19, 55.13, 55.03, 51.85, 47.40, 42.70, 42.14, 22.96; IR (KBr plates) 3002, 2954, 1681, 1605, 1513, 1445, 1375, 1249, 1178, 1034, 838, 755 cm^{-1}; HRMS (ESI) *m/z* $[M+H]^+$ calcd for $C_{36}H_{37}N_2O_6$ 593.2652, found 593.2632.

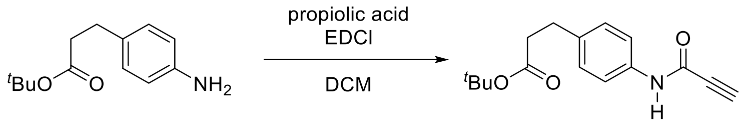

S04 **S05**

To a stirred solution of 0.087 g (0.393 mmol) of **S04**[2] in 3.0 mL of CH_2Cl_2 were added 0.110 g (0.590 mmol, 1.5 equiv) of EDC and 0.036 mL (0.590 mmol, 1.5 equiv) of propiolic acid at 0 °C, and the stirring was continued at the same temperature for 30 min The reaction mixture was quenched with water, extracted with CH_2Cl_2, dried over Na_2SO_4, and concentrated in vacuo. The crude material was chromatographed on silica gel with a gradient of 10–40% AcOEt/hexane to give 0.107 g (0.391 mmol, quant.) of **S05** as a colorless oil.

S05: ^{1}H NMR ($CDCl_3$, 600 MHz) δ 7.51 (1H, s), 7.42 (2H, d, J = 8.0 Hz), 7.17 (2H, d, J = 8.0 Hz), 2.91 (1H, s), 2.87 (2H, t, J = 7.7 Hz), 2.51 (2H, t, J = 7.7 Hz), 1.41 (9H, s); ^{13}C NMR ($CDCl_3$, 150 MHz) δ 172.1, 149.5, 137.9, 134.98, 129.0 (×2), 120.1 (×2), 80.5, 77.61, 73.92, 36.95, 30.52, 28.06 (×3); IR (neat) 3285, 2107, 1725, 1714, 1658, 1651, 1604, 1537, 1517, 1414, 1368, 1320, 1256, 1149, 844, 736 cm^{-1}; HRMS (ESI) m/z $[M+Na]^+$ calcd for $C_{16}H_{19}NNaO_3$ 296.1263, found 296.1258.

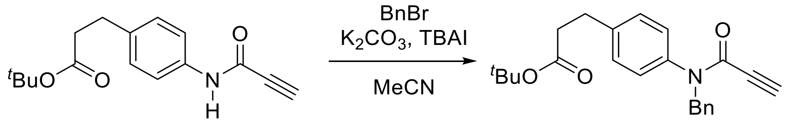

S05 **S06**

To a stirred solution of 0.110 g (0.402 mmol) of **S05** in 0.50 mL of MeCN were added 0.167 g (1.21 mmol, 3 equiv) of K_2CO_3, 5.7 mg (0.002 mmol, 0.04 equiv) of TBAI, and 0.143 mL (1.21 mmol, 3 equiv) of benzyl bromide at rt, and the stirring was continued at the same temperature for 22 h. The reaction mixture was diluted with AcOEt, filtered through a pad of Celite, and concentrated in vacuo. The crude material was chromatographed on silica gel with a gradient of 3–30% AcOEt/hexane to give 0.135 g (0.371 mmol, 92% yield) of **S06** as a colorless oil.

S06: ^{1}H NMR ($CDCl_3$, 600 MHz) (A mixture of rotamers in 85:15 ratio) δ 7.21–7.31 (5H, m), 7.17–7.19 (2H, m), 7.14 (2H, d, J = 8.0 Hz), 6.99 (2H, d, J = 8.0 Hz), 5.15 (0.3H, s), 4.90 (1.7H, s), 3.23 (0.15H, s) 2.89 (1.7H, t, J = 7.8 Hz), 2.85 (0.3H, t, J = 7.8 Hz), 2.79 (0.85H, s), 2.53 (1.7H, t, J = 7.8 Hz), 2.49 (0.3H, t, J = 7.8 Hz) 1.38 (9H, s); ^{13}C NMR ($CDCl_3$, 150 MHz) δ 171.9, 153.2, 141.1, 139.0, 136.3, 129.1 (×2), 128.8 (×2), 128.5 (×2), 128.3 (×2), 127.7, 126.6, 80.5, 80.0, 52.5, 36.6, 30.6, 28.0 (×3); IR (neat) 2978, 2107, 1725, 1643, 1513, 1391, 1147, 847, 737, 701 cm^{-1}; HRMS (ESI) m/z $[M+Na]^+$ calcd for $C_{23}H_{25}NNaO_3$ 386.1732, found 386.1724.

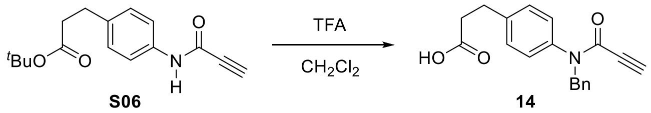

To a stirred solution of 0.045 mg (0.120 mmol) of **S06** in 0.30 mL of CH_2Cl_2 was added 0.290 mL (3.70 mmol, 30 equiv) of trifluoroacetic acid at rt, and the stirring was continued at the same temperature for 2 h. The reaction mixture was concentrated and chromatographed on silica gel with a gradient of 10–100% AcOEt/hexane to give 34.9 mg (0.114 mmol, 92% yield) of **14** as a colorless solid.

14: ^{1}H NMR (600 MHz, $CDCl_3$) (A mixture of rotamers in 85:15 ratio) δ 7.24–7.29 (3H, m), 7.19 (2H, d, *J* = 7.6 Hz), 7.16 (2H, d, *J* = 8.2 Hz), 7.00–7.03 (2H, m), 5.15 (0.3H, s), 4.91 (1.7H, s), 3.23 (0.15H, s), 2.96 (1.70H, t, *J* = 7.7 Hz), 2.91 (0.3H, t, *J* = 7.7 Hz), 2.81 (0.85H, s), 2.67 (1.7H, t, *J* = 7.7 Hz), 2.64 (0.3H, t, *J* = 7.7 Hz); ^{13}C NMR (150 MHz, $CDCl_3$) δ 178.00, 153.23, 140.56, 139.24, 136.17, 128.97 (×2), 128.70 (×2), 128.47 (×2), 128.45 (×2), 127.66, 80.28, 76.13, 52.47, 35.15, 30.07; IR (KBr plates) 3276, 3234, 3033, 2110, 1721, 1637, 1513, 1400, 738, 701 cm^{-1}; HRMS (ESI) *m*/*z* [M+Na]$^+$ calcd for $C_{19}H_{17}NNaO_3$ 330.1106, found 330.1127.

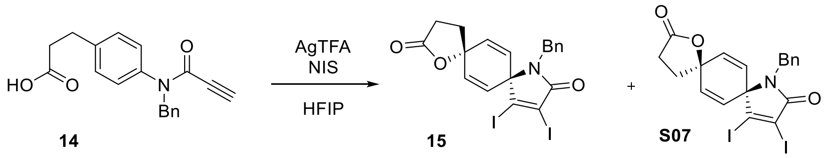

To a stirred solution of 13.3 mg (0.043 mmol) of **14** in 3.4 mL of HFIP were added 0.89 mg (0.0043 mmol, 0.1 equiv) of AgTFA followed by 21.4 mg (0.095 mmol, 2.2 equiv) of NIS at 0 °C, then the stirring was continued at the same temperature for 24 h. The reaction mixture was quenched with 10% aqueous Na_2SO_3 and diluted with $CHCl_3$. After the organic layer separation, the aqueous layer was extracted with $CHCl_3$. The combined organic layers were dried over Na_2SO_4, and concentrated in vacuo. The crude product was chromatographed on silica gel with a gradient of 10–60% AcOEt/hexane to give 7.7 mg (0.014 mmol, 32% yield) of **S07** as a colorless solid followed by 14.2 mg (0.025 mmol, 59% yield) of **15** as a colorless solid.

15: ^{1}H NMR (600 MHz, $CDCl_3$) δ 7.23–7.29 (5H, m), 6.14 (2H, d, *J* = 10.0 Hz), 5.26 (2H, d, *J* = 10.0 Hz), 4.54 (2H, s), 2.73 (2H, t, *J* = 8.2 Hz), 2.38 (2H, t, *J* = 8.4 Hz); ^{13}C NMR (150 MHz, $CDCl_3$) δ 175.59, 166.92, 137.26, 133.23 (×2), 129.42, 129.05 (×2), 128.45 (×2), 128.23 (×2), 127.77, 109.63, 77.44, 72.13, 45.98, 33.85, 28.27; IR (KBr plates) 2921, 2363, 2343, 1772, 1697, 1179 cm^{-1}; HRMS (ESI) *m*/*z* [M+H]$^+$ calcd for $C_{19}H_{16}I_2NO_3$ 559.9220, found 559.9216.

S07: ^{1}H NMR (600 MHz, $CDCl_3$) δ 7.23–7.29 (5H, m), 6.15 (2H, d, *J* = 10.0 Hz), 5.26 (2H, d, *J* = 10.0 Hz), 4.55 (2H, s), 2.73 (2H, t, *J* = 8.4 Hz), 2.38 (2H, t, *J* = 8.4 Hz); ^{13}C NMR (150 MHz, $CDCl_3$) δ 175.74, 166.33, 137.15, 133.07 (×2), 129.25 (×2), 128.42 (×2), 127.77 (×2), 127.67 (×2),

110.31, 77.39, 72.30, 46.08, 34.20, 28.18; IR (KBr plates) 2919, 1751, 1682 cm^{-1}; HRMS (ESI) *m/z* $[M+H]^+$ calcd for $C_{19}H_{16}I_2NO_3$ 559.9220, found 559.9247.

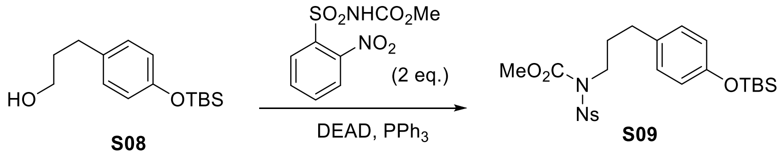

According to the literature procedure,[3] to a stirred solution of 0.235 g (1.35 mmol, 2.0 equiv.) of diethyl azodicarboxylate in 0.60 mL of toluene solution were added 0.180 g (0.675 mmol, 1.0 equiv.) of **S08**[4], and 0.351 g (1.35 mmol, 2.0 equiv.) of methyl ((2-nitrophenyl)sulfonyl)carbamate, and a solution of 0.354 g (1.35 mmol, 2.0 equiv.) of PPh_3 in 2.0 mL of toluene successively at rt, and the stirring was continued at the same temperature for 3 h. To the reaction mixture was added a mixed solution of 70% AcOEt/hexane, then filtered, and the filtrate was concentrated in vacuo. The crude product was chromatographed on silica gel with a gradient of 5–20% CH_2Cl_2/hexane to give 0.250 g (0.491 mmol, 73% yield) of **S09** as a colorless solid

S09: ^{1}H NMR (400 MHz, $CDCl_3$) δ 8.36–8.39 (1H, m), 7.75–7.77 (3H, m), 7.07 (2H, d, *J* = 8.5 Hz), 6.76 (2H, d, *J* = 8.5 Hz), 3.87 (2H, t, *J* = 7.5 Hz), 3.72 (3H, s), 2.65 (2H, t, *J* = 8.0 Hz), 2.01–2.09 (2H, m), 0.97 (9H, t, *J* = 3.0 Hz), 0.18 (6H, s); ^{13}C NMR (100 MHz, $CDCl_3$) δ 153.75, 152.51, 147.89, 134.48, 134.34, 133.65, 132.79, 131.71, 129.11 (×2), 124.42, 119.92 (×2), 54.02, 47.97, 32.03, 31.88, 25.68 (×3), 18.18, –4.45 (×2); IR (KBr plates) 3455, 2956, 2930, 2896, 2858, 1739, 1545, 1510, 1367, 1261, 1172 cm^{-1}; HRMS (ESI) *m/z* $[M+Na]^+$ calcd for $C_{23}H_{32}N_2NaO_7SSi$ 531.1597, found 531.1597.

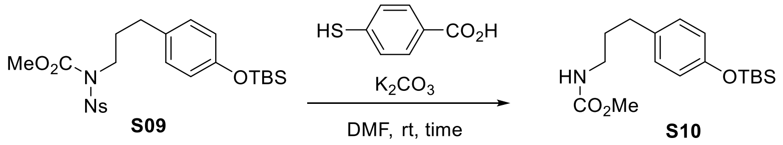

To a stirred solution of 2.19 g (4.31 mmol) of **S09** in 25 mL of DMF were added 0.86 mL (5.60 mmol, 1.3 equiv) of 4-mercaptobenzoic acid, 1.19 g (8.62 mmol, 2.0 equiv) of K_2CO_3 at rt, then the stirring was continued at the same temperature for 21 h. The reaction mixture was quenched with saturated aqueous $NaHCO_3$, and extracted with AcOEt. The combined organic layers were washed with saturated aqueous $NaHCO_3$, water, and brine, dried over Na_2SO_4, and concentrated in vacuo. The crude material was chromatographed on silica gel with a gradient of 20–60% AcOEt/hexane to give 1.39 g (2.32 mmol, 54% yield) of **S10** as a yellow oil.

S10: ^{1}H NMR (400 MHz, $CDCl_3$) δ 7.01 (2H, d, *J* = 8.5 Hz), 6.75 (2H, d, *J* = 8.3 Hz), 4.65 (1H, s), 3.65 (3H, s), 3.19 (2H, q, *J* = 6.6 Hz), 2.57 (2H, t, *J* = 7.7 Hz), 1.75–1.83 (2H, m), 0.97 (9H, s), 0.18

(6H, s); ^{13}C NMR (100 MHz, $CDCl_3$) δ 157.02, 153.69, 133.97, 129.09 (×2), 119.90 (×2), 51.94, 40.57, 32.14, 31.68, 25.63 (×3), 18.12, –4.50 (×2) IR (neat) 3338, 2955, 2930, 2893, 2858, 1707, 1509, 1259 cm^{-1} ; HRMS (ESI) *m/z* $[M+H]^+$ calcd for $C_{17}H_{30}NO_3Si$ 324.1995, found 324.1996.

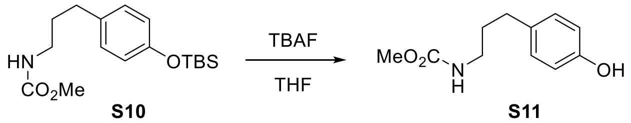

To a stirred solution of 0.080 g (0.240 mmol) of **S10** in 5.0 mL of THF was added 0.48 mL (0.47 mmol, 2.0 equiv) of TBAF at rt, and the stirring was continued at the same temperature for 1 h. The reaction mixture was diluted with AcOEt, then washed with brine, dried over Na_2SO_4, and concentrated in vacuo. The crude material was chromatographed on silica gel with a gradient of 5–30% AcOEt/hexane to give 0.050 g (0.230 mmol, 96% yield) of **S11** as a yellow oil.

S11: ^{1}H NMR (400 MHz, $CDCl_3$) δ 6.98 (2H, d, *J* = 8.3 Hz), 6.77 (2H, d, *J* = 8.5 Hz), 5.99 (1H, s), 4.85 (1H, s), 3.67 (3H, s), 3.15–3.23 (2H, m), 2.54 (2H, t, *J* = 7.5 Hz), 1.73–1.80 (2H, m); ^{13}C NMR (100 MHz, $CDCl_3$) δ 157.82, 154.50, 132.76, 129.34 (×2), 115.40 (×2), 52.37, 40.71, 32.05, 31.72; IR (neat) 3342, 2944, 2360, 1698, 1516, 1264 cm^{-1}; HRMS (ESI) *m/z* $[M+Na]^+$ calcd for $C_{11}H_{15}NNaO_3$ 232.0950, found 232.0932.

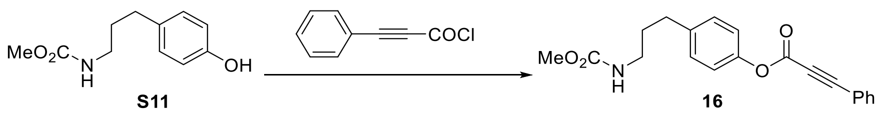

To a stirred solution of 0.36 g (1.86 mmol, 1.3 equiv) of phenylpropiolic acid in 6.0 mL of CH_2Cl_2 was added dropwise 0.19 mL (2.24 mmol, 1.6 equiv) of $COCl_2$ containing one drop of DMF at rt, then the stirring was continued at the same temperature for 1 h. After evaporation, the reaction mixture was dissolved in 6.0 mL of CH_2Cl_2, then to the stirred solution were added 0.60 mL (4.30 mmol, 3.0 equiv) of Et_3N, and a solution of 0.30 g (1.44 mmol, 1.0 eq.) of **S11** in 2.0 mL of CH_2Cl_2 at 0 °C. The reaction mixture was stirred at rt for 20 h, quenched with saturated aqueous $NaHCO_3$, and diluted with AcOEt. After the organic layer separation, the aqueous layer was extracted with AcOEt. The combined organic layers were washed with saturated aqueous $NaHCO_3$, dried over Na_2SO_4, concentrated in vacuo. The crude material was chromatographed on silica gel with a gradient of 5–30% AcOEt/hexane to give 0.49 g (1.36 mmol, 73% yield) of **16** as a yellow oil.

16: ^{1}H NMR (400 MHz, $CDCl_3$) δ 7.62–7.65 (2H, m), 7.47–7.52 (1H, m), 7.39–7.43 (2H, m), 7.22 (2H, d, *J* = 8.8 Hz), 7.10 (2H, d, *J* = 8.5 Hz), 4.69 (1H, s), 3.67 (3H, s), 3.23 (2H, q, *J* = 6.6 Hz), 2.66 (2H, t, *J* = 7.8 Hz), 1.80–1.87 (2H, m); ^{13}C NMR (100 MHz, $CDCl_3$) δ 157.02, 152.39, 148.16, 139.58, 133.04 (×2), 130.93, 129.29 (×2), 128.56 (×2), 121.21 (×2), 119.09, 88.52, 80.15, 51.94,

40.43, 32.26, 31.48; IR (neat) 3338, 2945, 2864, 2234, 1726, 1507, 1284, 1195, 1169, 1149 cm^{-1}; HRMS (ESI) *m/z* $[M+H]^+$ calcd for $C_{20}H_{20}NO_4$ 338.1392, found 338.1392.

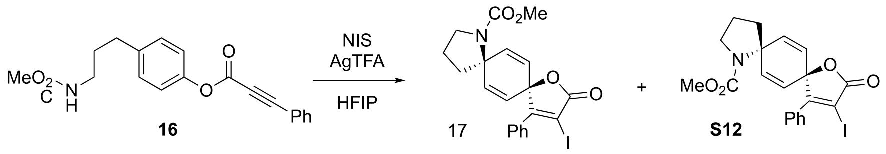

To a stirred solution of 20.0 mg (0.060 mmol) of **16** in 1.6 mL of HFIP were added 1.0 mg (0.006 mmol, 0.1 equiv) of AgTFA followed by 15.0 mg (0.065 mmol, 1.1 equiv) of NIS at 0 °C, then the stirring was continued at the same temperature for 24 h. The reaction mixture was quenched with 10% aqueous Na_2SO_3 and diluted with $CHCl_3$. After the organic layer separation, the aqueous layer was extracted with $CHCl_3$. The combined organic layers were washed with water and brine, dried over Na_2SO_4, and concentrated in vacuo. The crude product was purified on PTLC with 25% AcOEt/$CHCl_3$ to give 15.7 mg (0.034 mmol, 57% yield) of **17** as a colorless solid followed by 7.4 mg (0.016 mmol, 27% yield) of **S12** as a colorless solid.

17: ^{1}H NMR (400 MHz, $CDCl_3$) (A mixture of rotamers in 55:45 ratio) δ 7.38–7.44 (3H, m), 7.31 (2H, d, *J* = 6.3 Hz), 5.96–6.06 (2H, m), 5.65–5.73 (0.9H, m), 5.57–5.65 (1.1H, m), 3.60 (3H, s), 3.52–3.58 (1.1H, m), 3.45–3.52 (0.9H, m), 1.73-1.80 (2H, m), 1.35–1.39 (1.1H, m), 1.21–1.35 (0.9H, m); ^{13}C NMR (100 MHz, $CDCl_3$) (Asterisks indicate peaks for the minor rotamer) δ 171.11, 169.43, 155.38, 137.21 (×2), 136.85* (×2), 131.73, 130.16*, 129.99, 128.32 (×2), 127.56 (×2), 122.30* (×2), 121.71 (×2), 86.78, 85.68, 58.73, 52.10, 47.81, 47.19*, 40.16, 22.92*, 22.33; IR (KBr plates) 2952, 2880, 2362, 2340, 1766, 1750, 1692, 1446, 1377 cm^{-1}; HRMS (ESI) *m/z* $[M+Na]^+$ calcd for $C_{20}H_{18}INNaO_4$ 486.0178, found 486.0178.

S12: ^{1}H NMR (600 MHz, $CDCl_3$) (A mixture of rotamers in 65:35 ratio) δ 8.18–8.26 (1H, m), 7.56–7.64 (0.6H, m), 7.43–7.49 (3.4H, m), 6.24–6.28 (2H, m), 5.68–5.76 (2H, m), 3.68 (1.95H, s), 3.58–3.72 (2H, m), 3.12 (1.05H, s), 2.04–2.08 (2H, m), 1.94–2.02 (2H, m); ^{13}C NMR (150 MHz, –10 °C, $CDCl_{3,}$) (Asterisks indicate peaks for the minor rotamer) δ 170.11, 167.27, 154.88, 140.84* (×2), 139.67 (×2), 131.73, 130.55*, 129.29, 129.08 (×2), 128.54* (×2), 128.43 (×2), 127.86* (×2), 123.16 (×2), 121.29* (×2), 86.83*, 85.15, 84.92, 82.90*, 59.81, 59.41*, 52.21, 52.12*, 48.35*, 47.53, 42.16*, 40.84, 23.43, 22.74*; IR (KBr plates) 2972, 2949, 2884, 1740, 1688, 1447, 1381, 1229 cm^{-1} ; HRMS (ESI) *m/z* $[M+Na]^+$ calcd for $C_{20}H_{18}INNaO_4$ 486.0178, found 486.0179.

References

[1] N. Sakumoto, Y. Takayama, WO 2011149071, **2011**.

[2] G. Cristalli, E. Camaioni, S. Vittori, R. Volpini, P. A. Borea, A. Conti, S. Dionisotti, E. Ongini, A. Monopoli, *J. Med. Chem.* **1995**, *38*, 1462–1472.

[3] T. Fukuyama, M. Cheung, T. Kan, *Synlett* **1999**, 1301–1303.

[4] H. Liang, M. A. Ciufolini, *Org. Lett.* **2010**, *12*, 1760–1763.

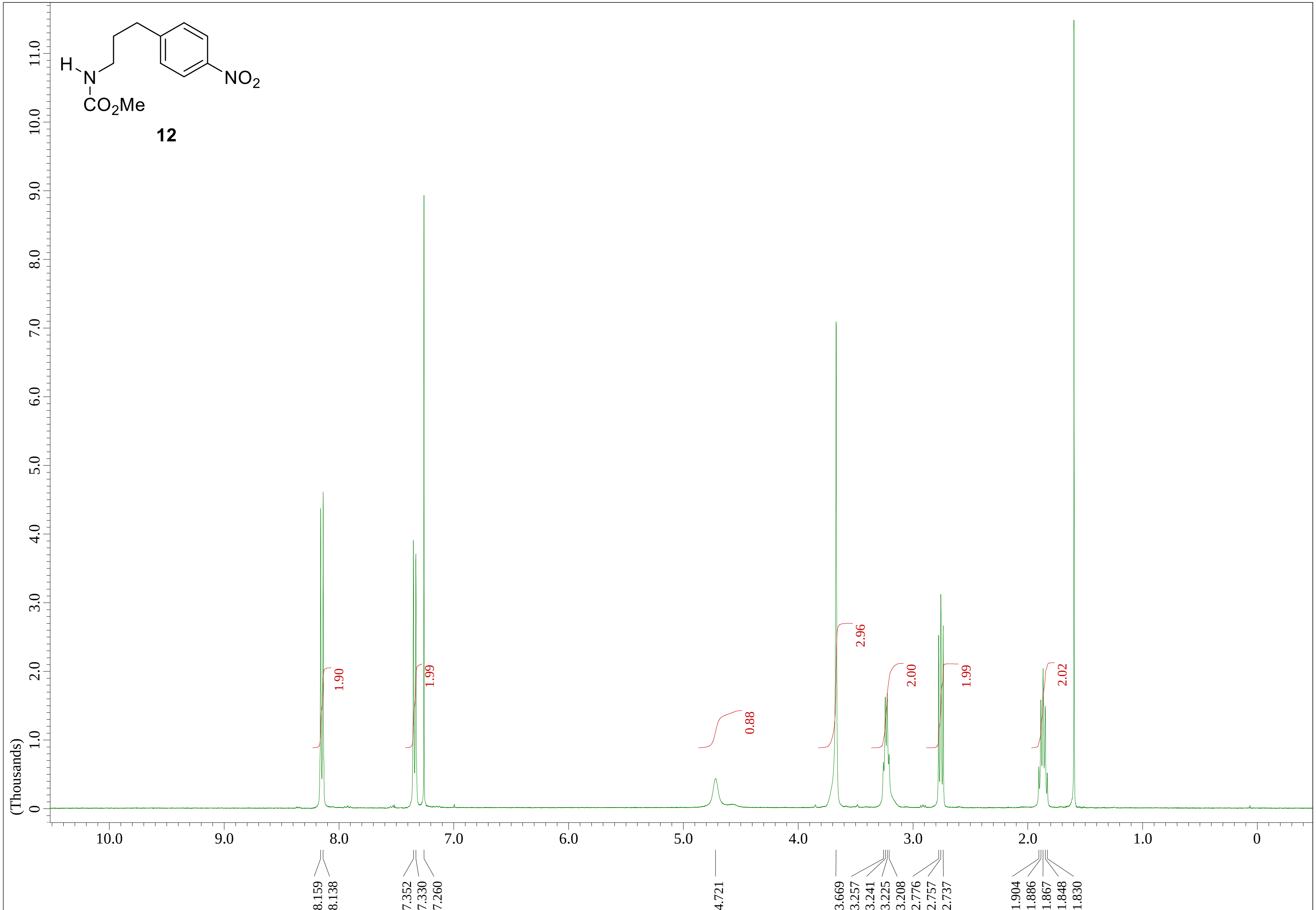

H
N
CO_2Me
NO_2
12
(Thousands)
0
1.0
2.0
3.0
4.0
5.0
6.0
7.0
8.0
9.0
10.0
11.0
1.90
1.99
0.88
2.96
2.00
1.99
2.02
10.0
9.0
8.0
7.0
6.0
5.0
4.0
3.0
2.0
1.0
0
8.159
8.138
7.352
7.330
7.260
4.721
3.669
3.257
3.241
3.225
3.208
2.776
2.757
2.737
1.904
1.886
1.867
1.848
1.830
X : parts per Million : 1H

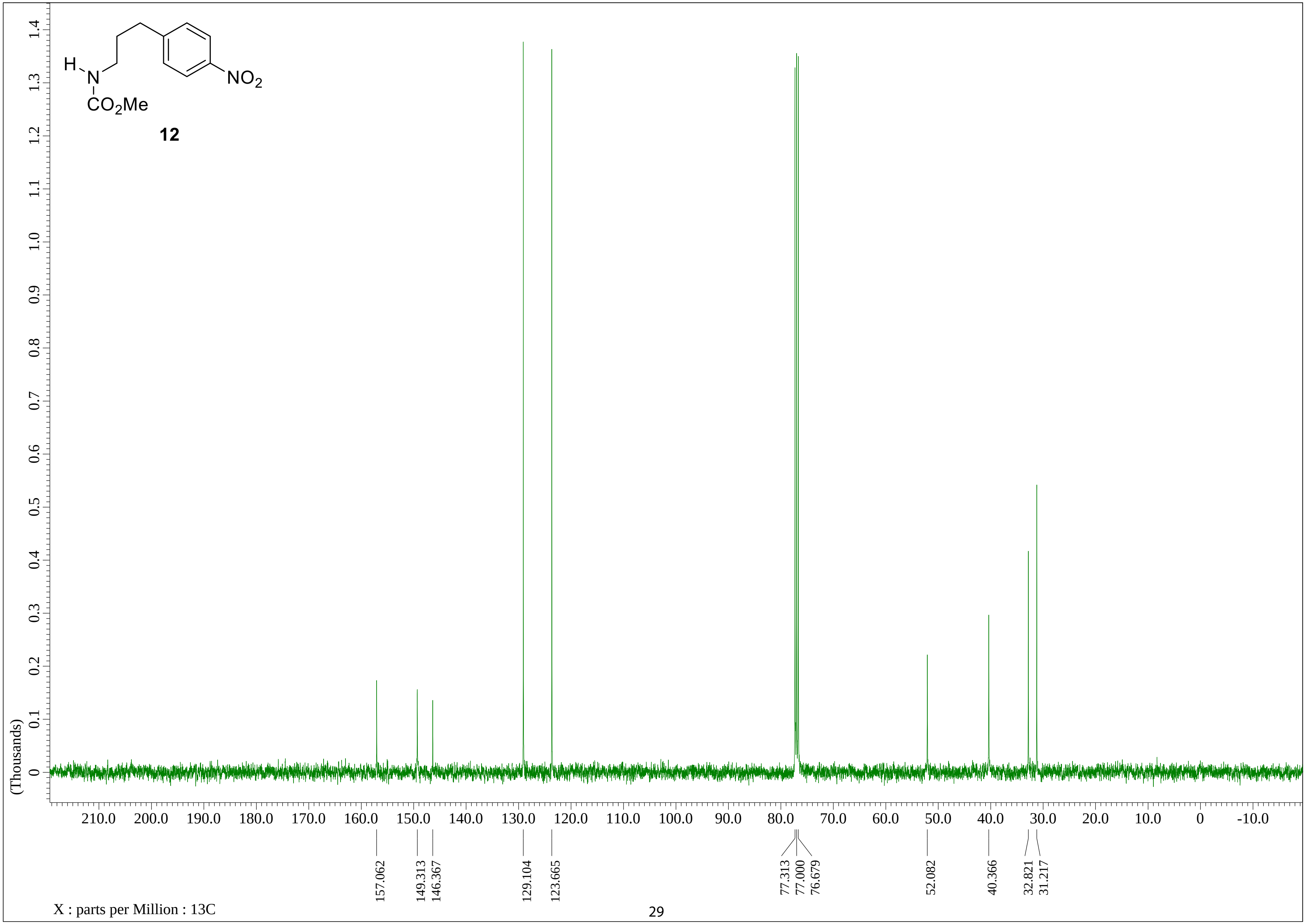
H
N
CO2Me
NO2
12
(Thousands)
1.4
1.3
1.2
1.1
1.0
0.9
0.8
0.7
0.6
0.5
0.4
0.3
0.2
0.1
0
210.0 200.0 190.0 180.0 170.0 160.0 150.0 140.0 130.0 120.0 110.0 100.0 90.0 80.0 70.0 60.0 50.0 40.0 30.0 20.0 10.0 0 -10.0
157.062
149.313
146.367
129.104
123.665
77.313
77.000
76.679
52.082
40.366
32.821
31.217
X : parts per Million : 13C

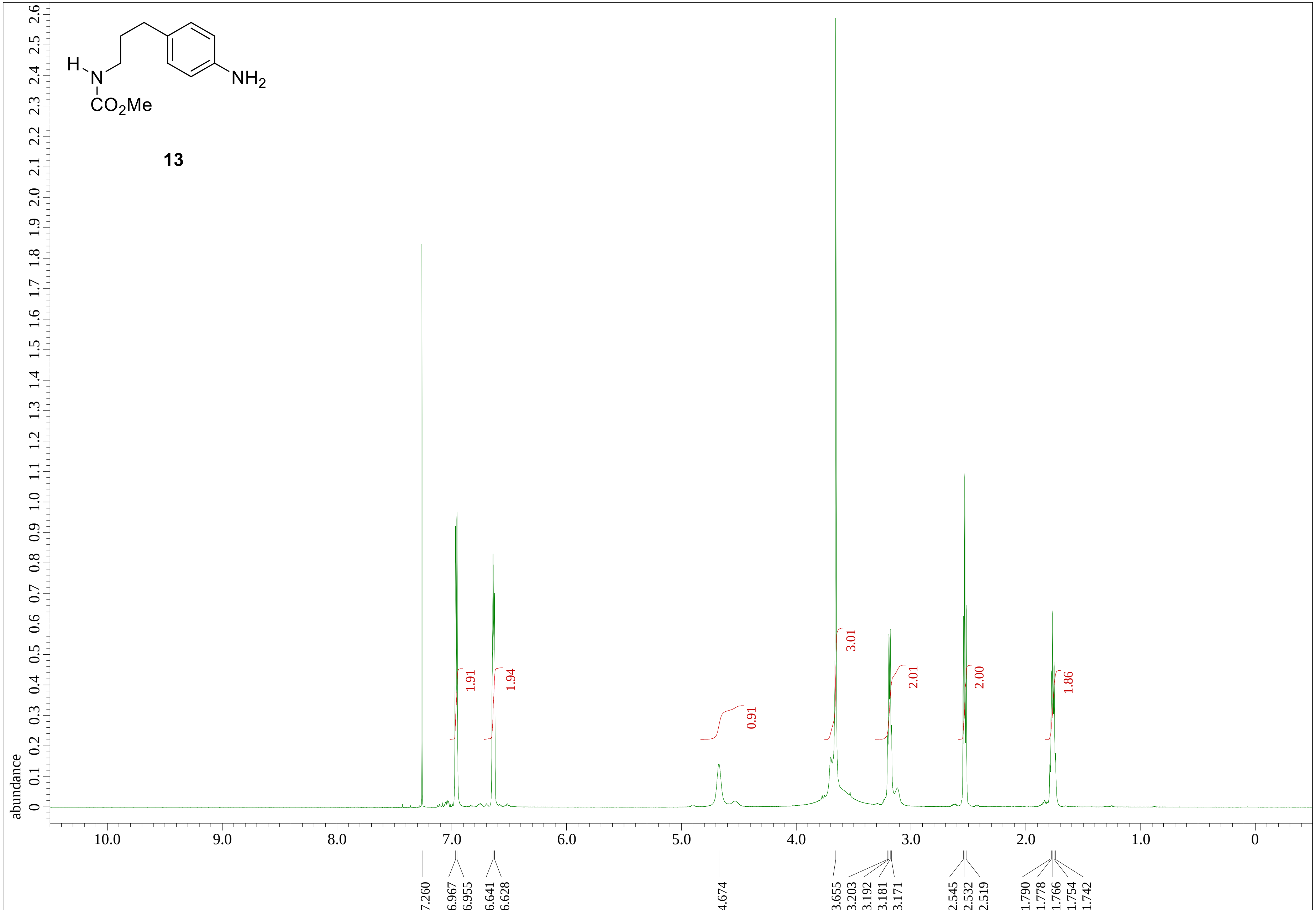
H
N
CO2Me
NH2
13
abundance
X : parts per Million : Proton
10.0
9.0
8.0
7.0
6.0
5.0
4.0
3.0
2.0
1.0
0
1.91
1.94
0.91
3.01
2.01
2.00
1.86
7.260
6.967
6.955
6.641
6.628
4.674
3.655
3.203
3.192
3.181
3.171
2.545
2.532
2.519
1.790
1.778
1.766
1.754
1.742

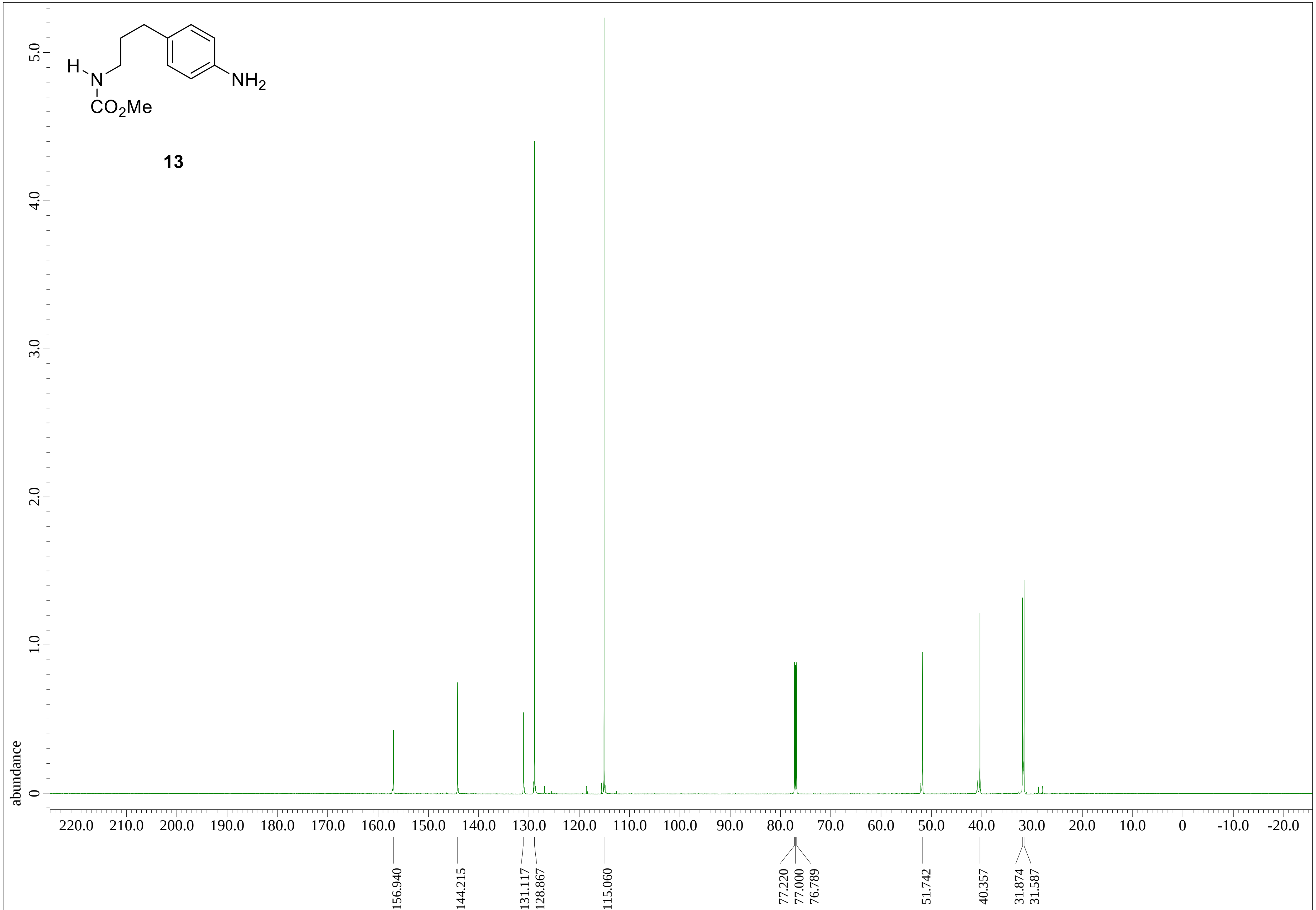
H
N
CO2Me
NH2
13
5.0
4.0
3.0
2.0
1.0
0
abundance
220.0 210.0 200.0 190.0 180.0 170.0 160.0 150.0 140.0 130.0 120.0 110.0 100.0 90.0 80.0 70.0 60.0 50.0 40.0 30.0 20.0 10.0 0 -10.0 -20.0
156.940
144.215
131.117
128.867
115.060
77.220
77.000
76.789
51.742
40.357
31.874
31.587
X : parts per Million : Carbon13

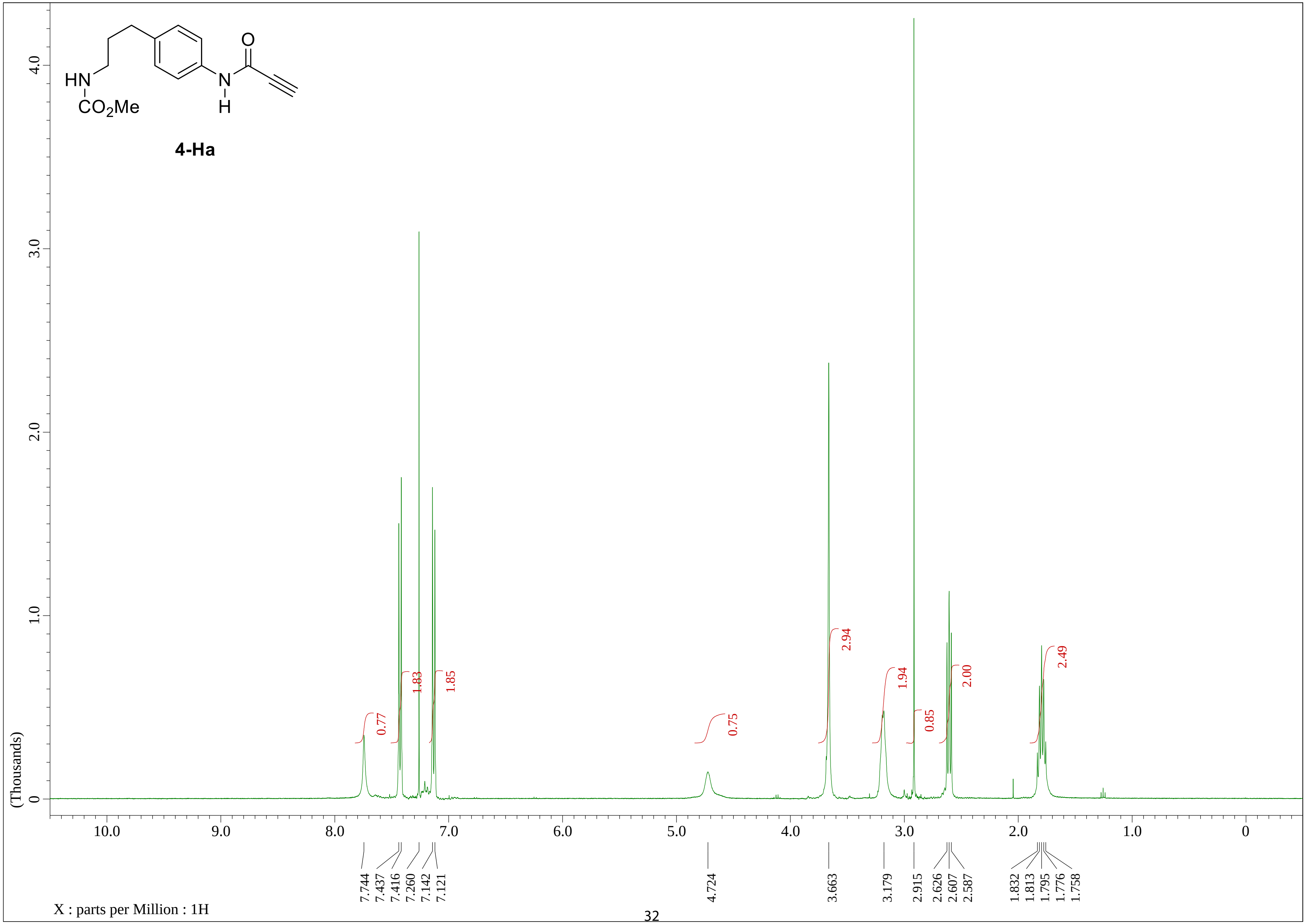
HN
CO2Me
N
H
O
4-Ha
(Thousands)
4.0
3.0
2.0
1.0
0
0.77
1.83
1.85
0.75
2.94
1.94
0.85
2.00
2.49
10.0
9.0
8.0
7.0
6.0
5.0
4.0
3.0
2.0
1.0
0
7.744
7.437
7.416
7.260
7.142
7.121
4.724
3.663
3.179
2.915
2.626
2.607
2.587
1.832
1.813
1.795
1.776
1.758
X : parts per Million : 1H

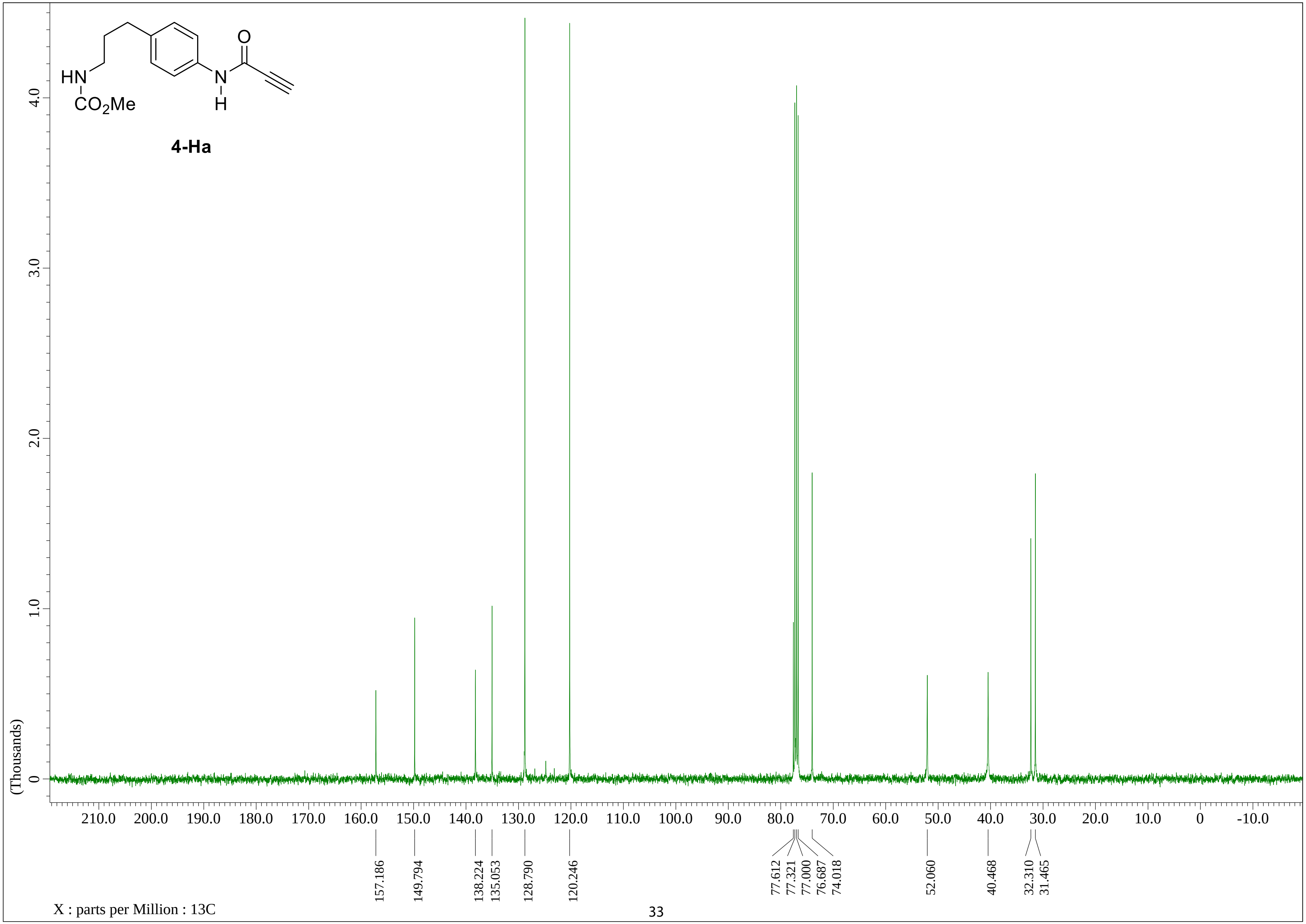
HN
CO2Me
N
H
O
4-Ha
(Thousands)
4.0
3.0
2.0
1.0
0
210.0 200.0 190.0 180.0 170.0 160.0 150.0 140.0 130.0 120.0 110.0 100.0 90.0 80.0 70.0 60.0 50.0 40.0 30.0 20.0 10.0 0 -10.0
157.186
149.794
138.224
135.053
128.790
120.246
77.612
77.321
77.000
76.687
74.018
52.060
40.468
32.310
31.465
X : parts per Million : 13C

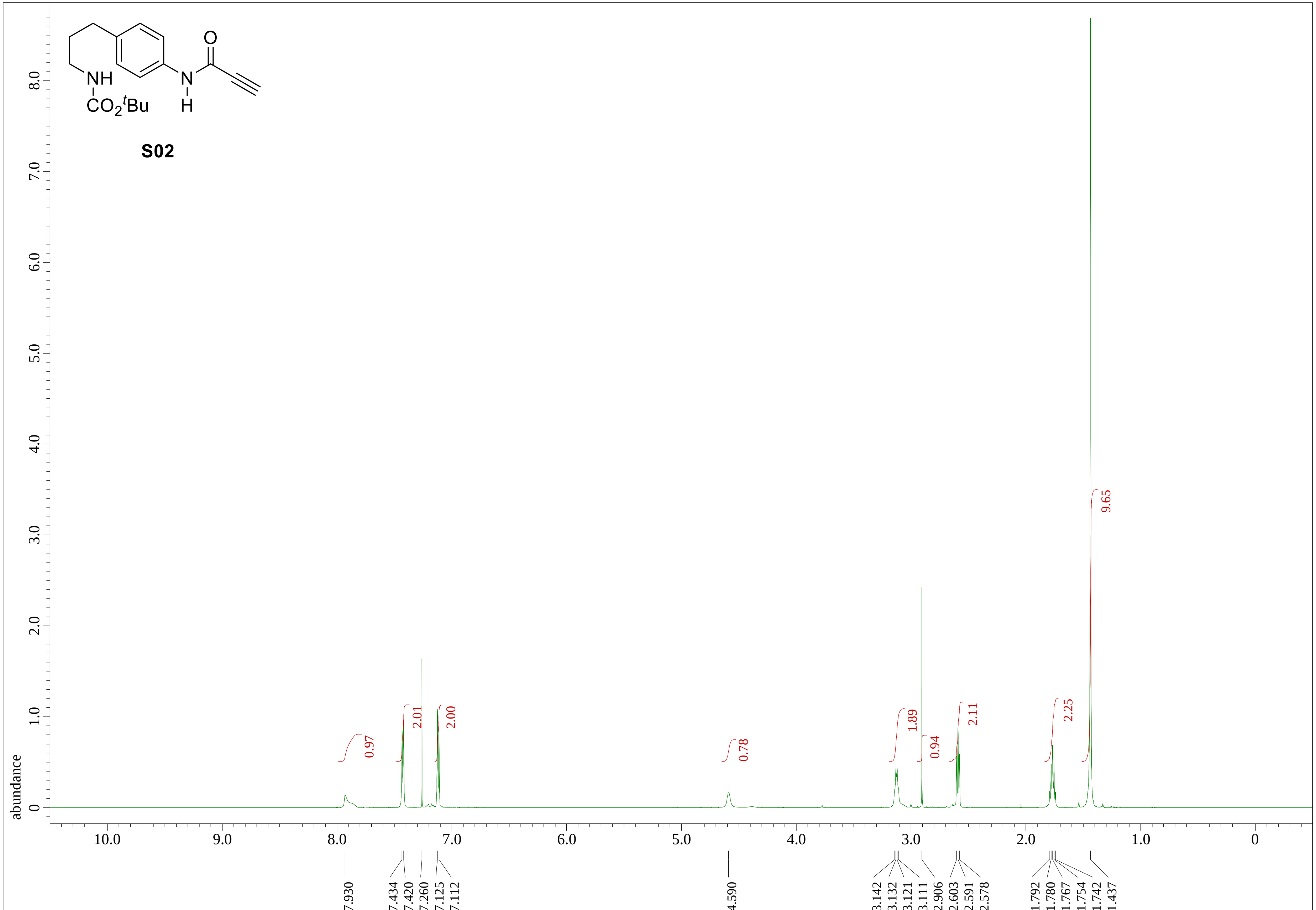

S02
NH
$CO_2{}^tBu$
N
H
O
abundance
X : parts per Million : Proton
7.930
7.434
7.420
7.260
7.125
7.112
4.590
3.142
3.132
3.121
3.111
2.906
2.603
2.591
2.578
1.792
1.780
1.767
1.754
1.742
1.437
0.97
2.01
2.00
0.78
1.89
0.94
2.11
2.25
9.65

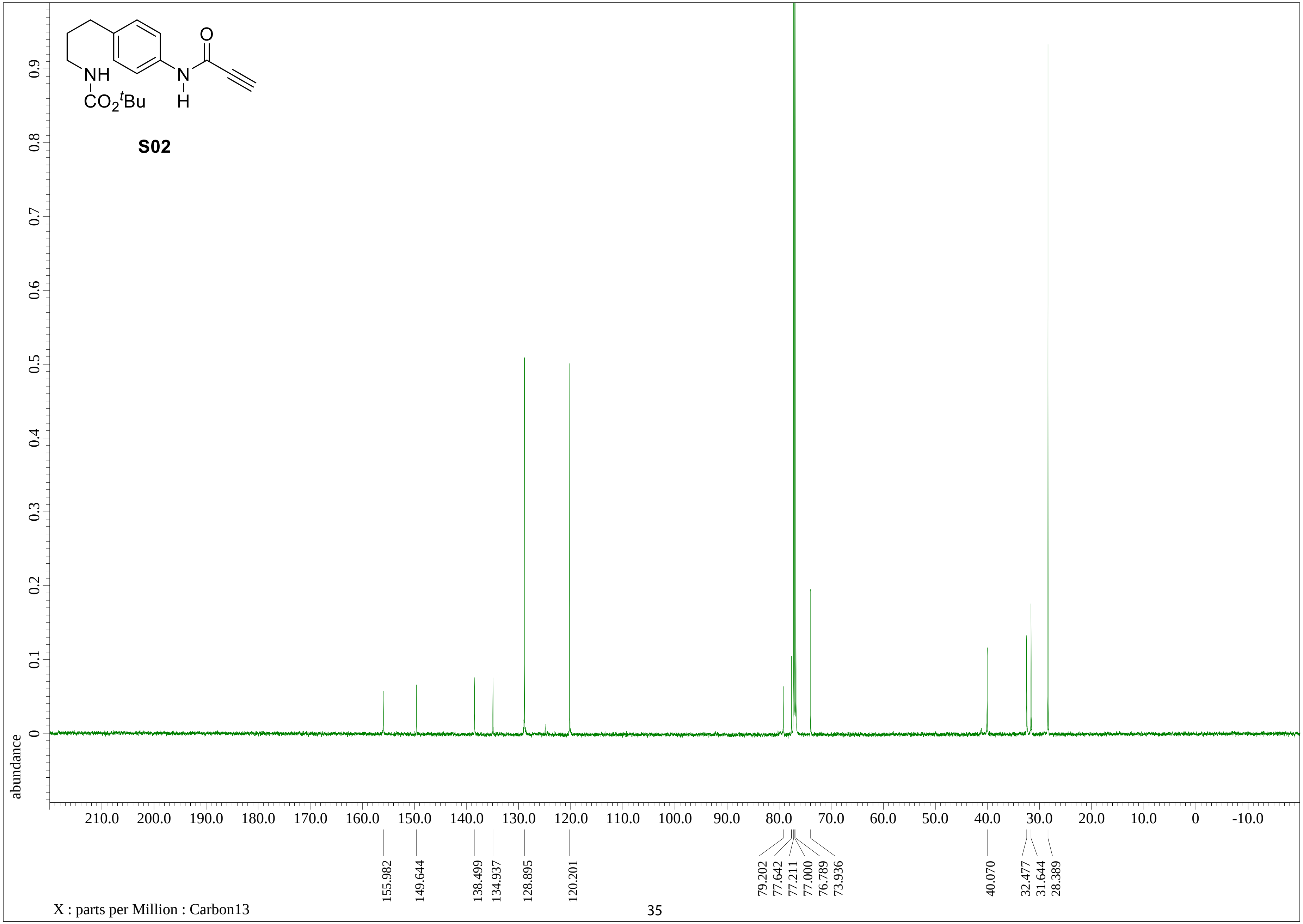
NH
$CO_2{}^tBu$
O
N
H
S02
abundance
0.9
0.8
0.7
0.6
0.5
0.4
0.3
0.2
0.1
0
210.0 200.0 190.0 180.0 170.0 160.0 150.0 140.0 130.0 120.0 110.0 100.0 90.0 80.0 70.0 60.0 50.0 40.0 30.0 20.0 10.0 0 -10.0
155.982
149.644
138.499
134.937
128.895
120.201
79.202
77.642
77.211
77.000
76.789
73.936
40.070
32.477
31.644
28.389
X : parts per Million : Carbon13

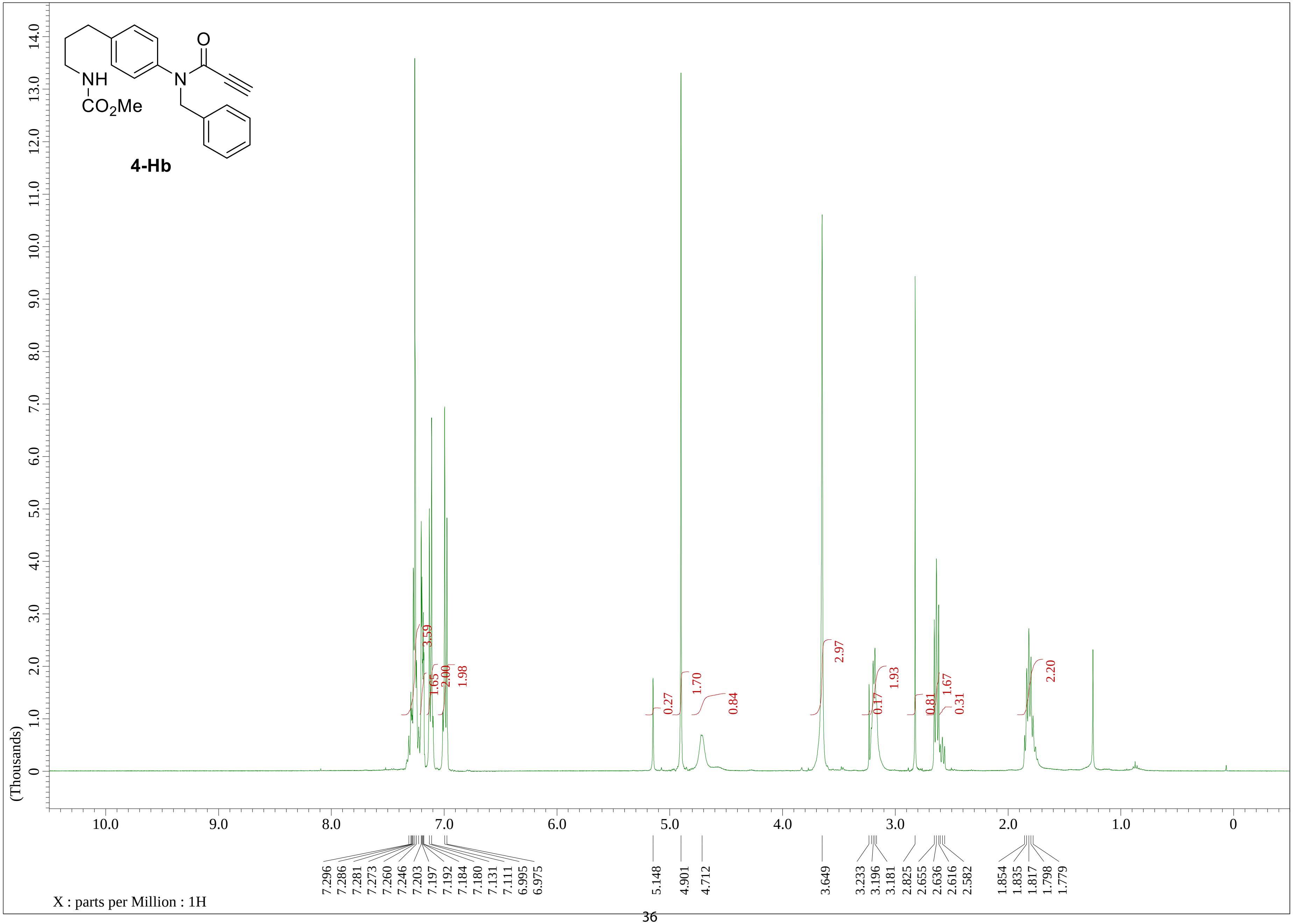

4-Hb
NH
CO2Me
N
O
(Thousands)
X : parts per Million : 1H
3.59
1.65
2.00
1.98
0.27
1.70
0.84
2.97
0.17
1.93
0.81
1.67
0.31
2.20
7.296
7.286
7.281
7.273
7.260
7.246
7.203
7.197
7.192
7.184
7.180
7.131
7.111
6.995
6.975
5.148
4.901
4.712
3.649
3.233
3.196
3.181
2.825
2.655
2.636
2.616
2.582
1.854
1.835
1.817
1.798
1.779

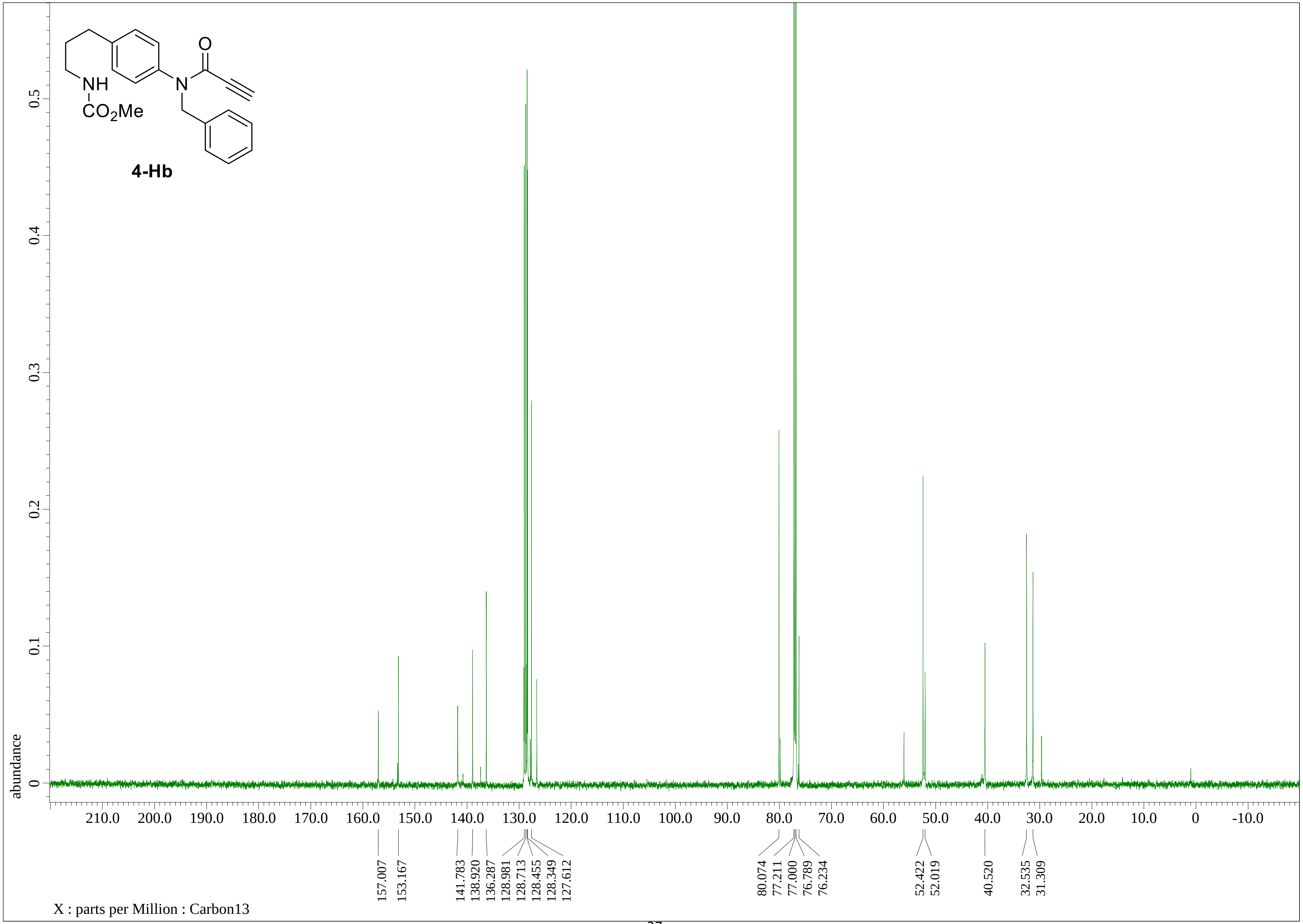
NH
CO2Me
N
O
4-Hb
abundance
0.5
0.4
0.3
0.2
0.1
0
210.0 200.0 190.0 180.0 170.0 160.0 150.0 140.0 130.0 120.0 110.0 100.0 90.0 80.0 70.0 60.0 50.0 40.0 30.0 20.0 10.0 0 -10.0
157.007
153.167
141.783
138.920
136.287
128.981
128.713
128.455
128.349
127.612
80.074
77.211
77.000
76.789
76.234
52.422
52.019
40.520
32.535
31.309
X : parts per Million : Carbon13

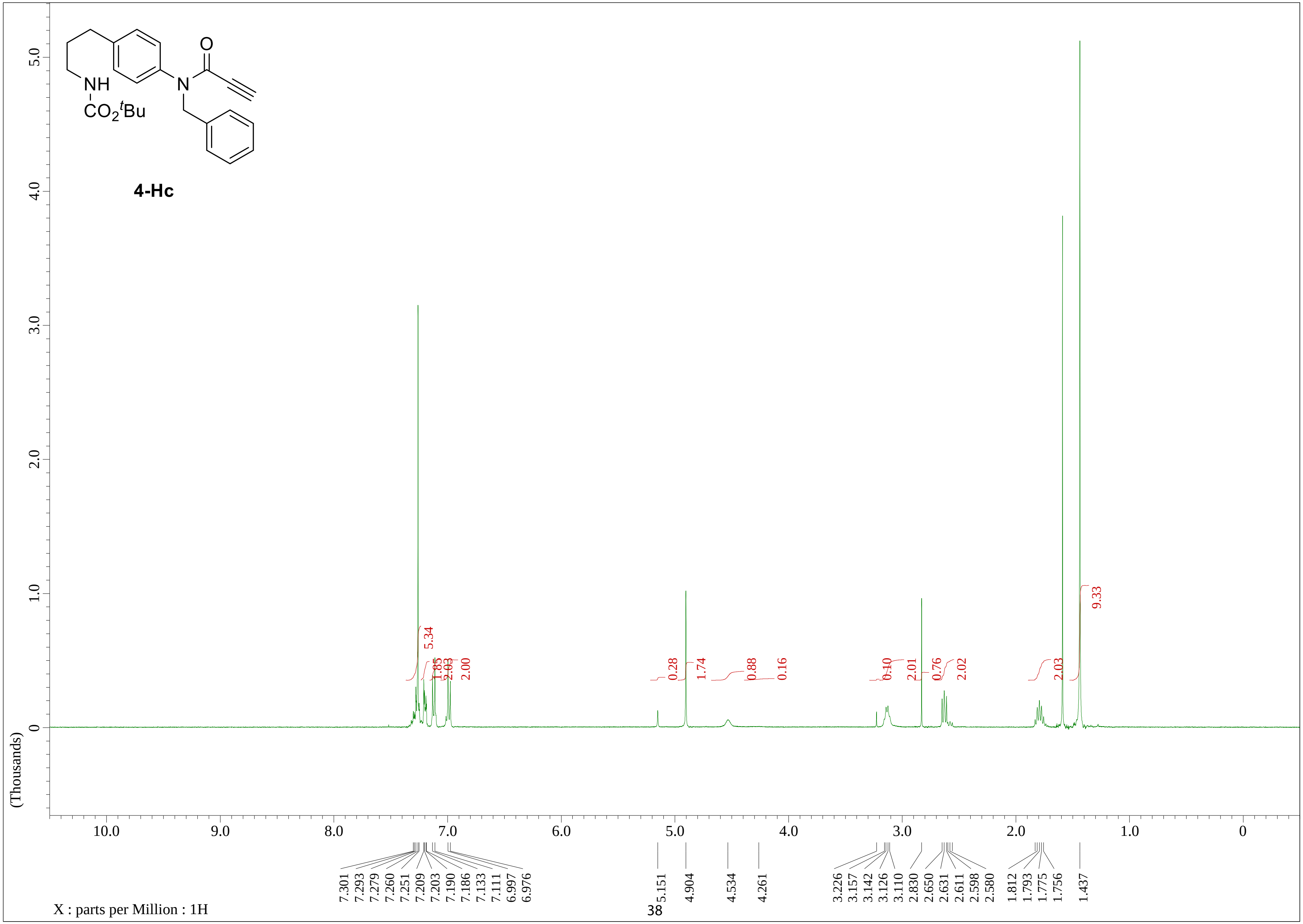
NH
$CO_2{}^tBu$
N
O
4-Hc
5.0
4.0
3.0
2.0
1.0
0
(Thousands)
10.0
9.0
8.0
7.0
6.0
5.0
4.0
3.0
2.0
1.0
0
5.34
1.85
2.03
2.00
0.28
1.74
0.88
0.16
0.10
2.01
0.76
2.02
2.03
9.33
7.301 7.293 7.279 7.260 7.251 7.209 7.203 7.190 7.186 7.133 7.111 6.997 6.976
5.151 4.904 4.534 4.261
3.226 3.157 3.142 3.126 3.110 2.830 2.650 2.631 2.611 2.598 2.580
1.812 1.793 1.775 1.756 1.437
X : parts per Million : 1H

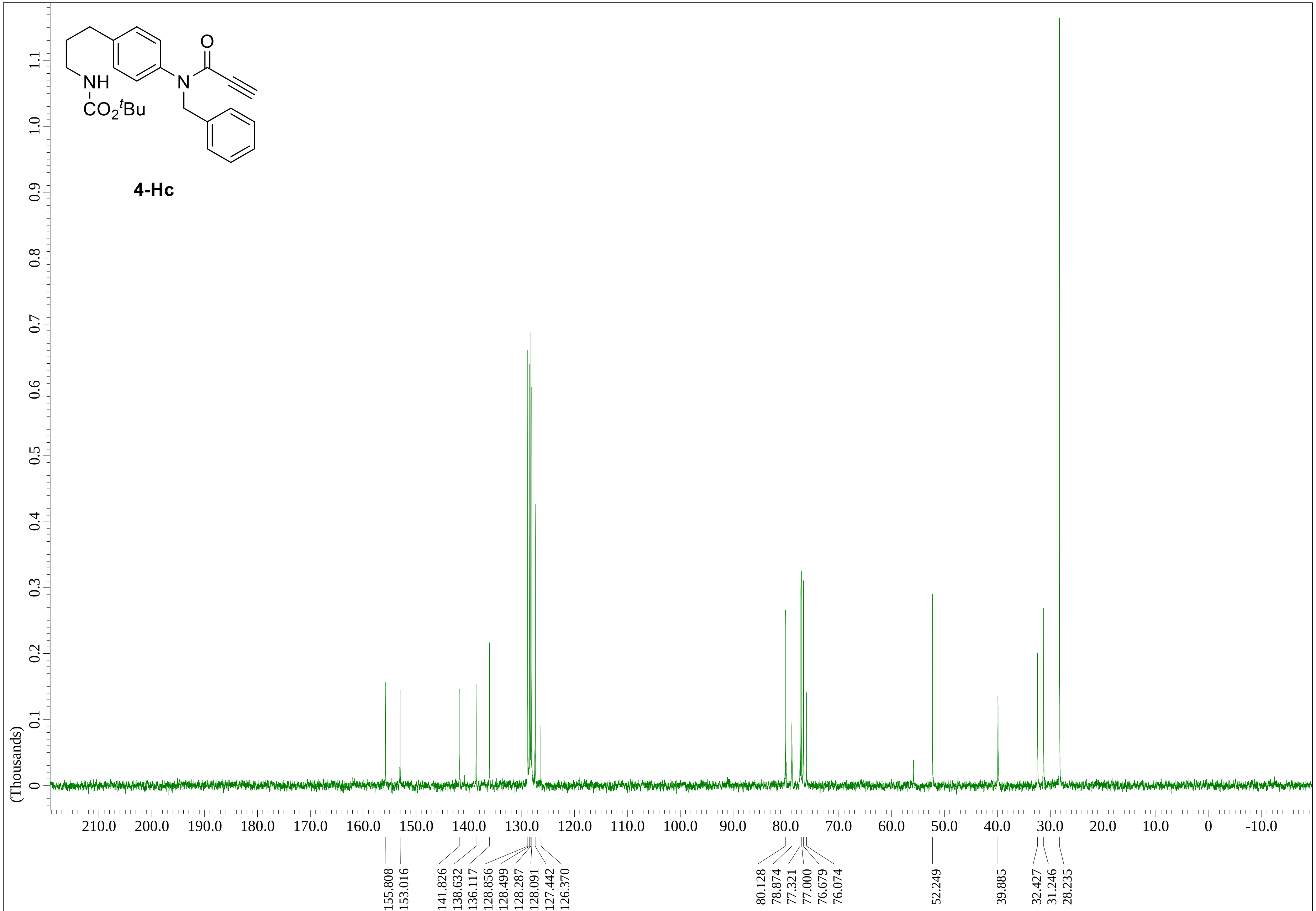
NH
$CO_2{}^tBu$
N
O
4-Hc
(Thousands)
0
0.1
0.2
0.3
0.4
0.5
0.6
0.7
0.8
0.9
1.0
1.1
210.0 200.0 190.0 180.0 170.0 160.0 150.0 140.0 130.0 120.0 110.0 100.0 90.0 80.0 70.0 60.0 50.0 40.0 30.0 20.0 10.0 0 -10.0
155.808
153.016
141.826
138.632
136.117
128.856
128.499
128.287
128.091
127.442
126.370
80.128
78.874
77.321
77.000
76.679
76.074
52.249
39.885
32.427
31.246
28.235
X : parts per Million : 13C

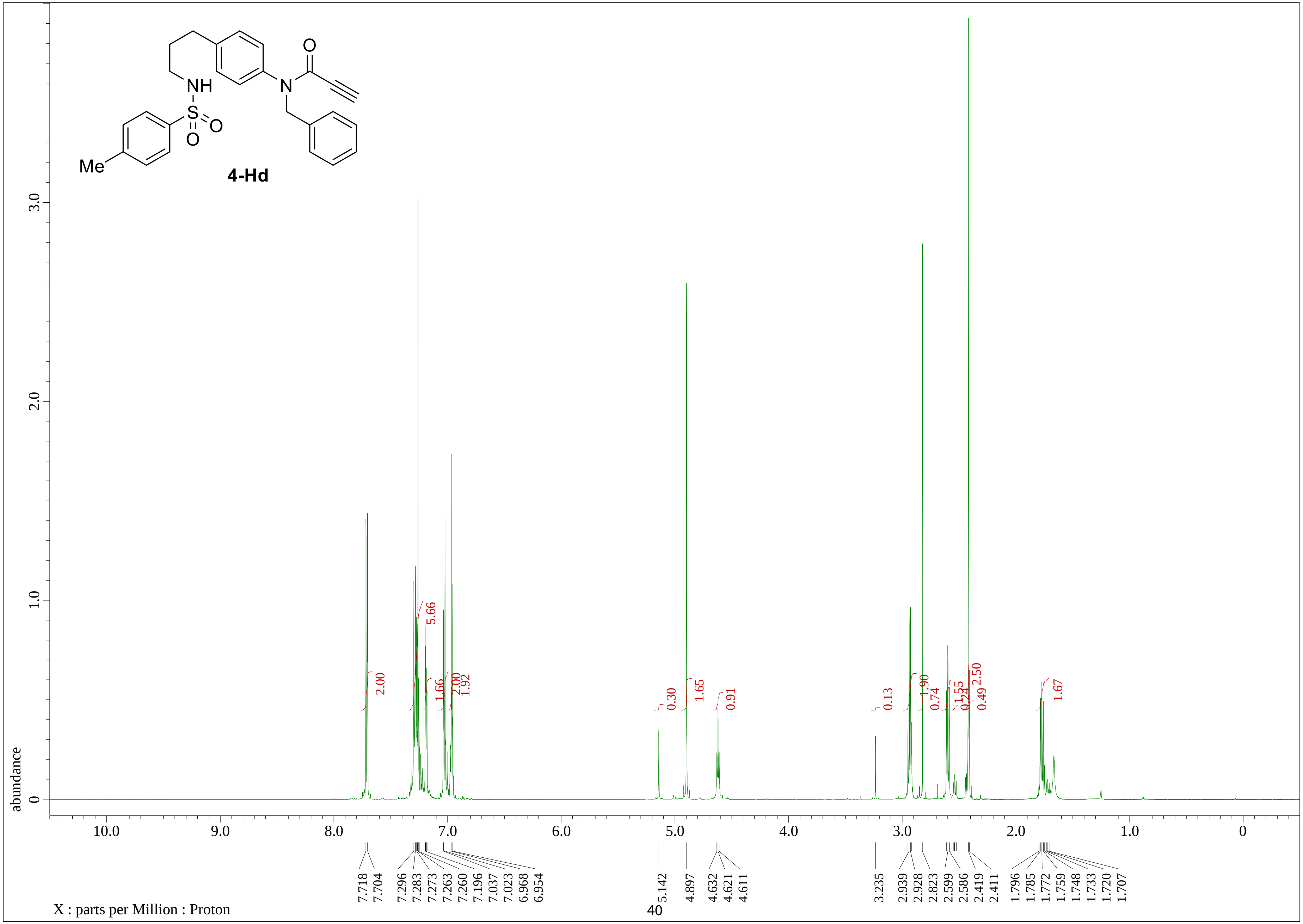

NH
S
O
O
Me
O
N
4-Hd
abundance
X : parts per Million : Proton

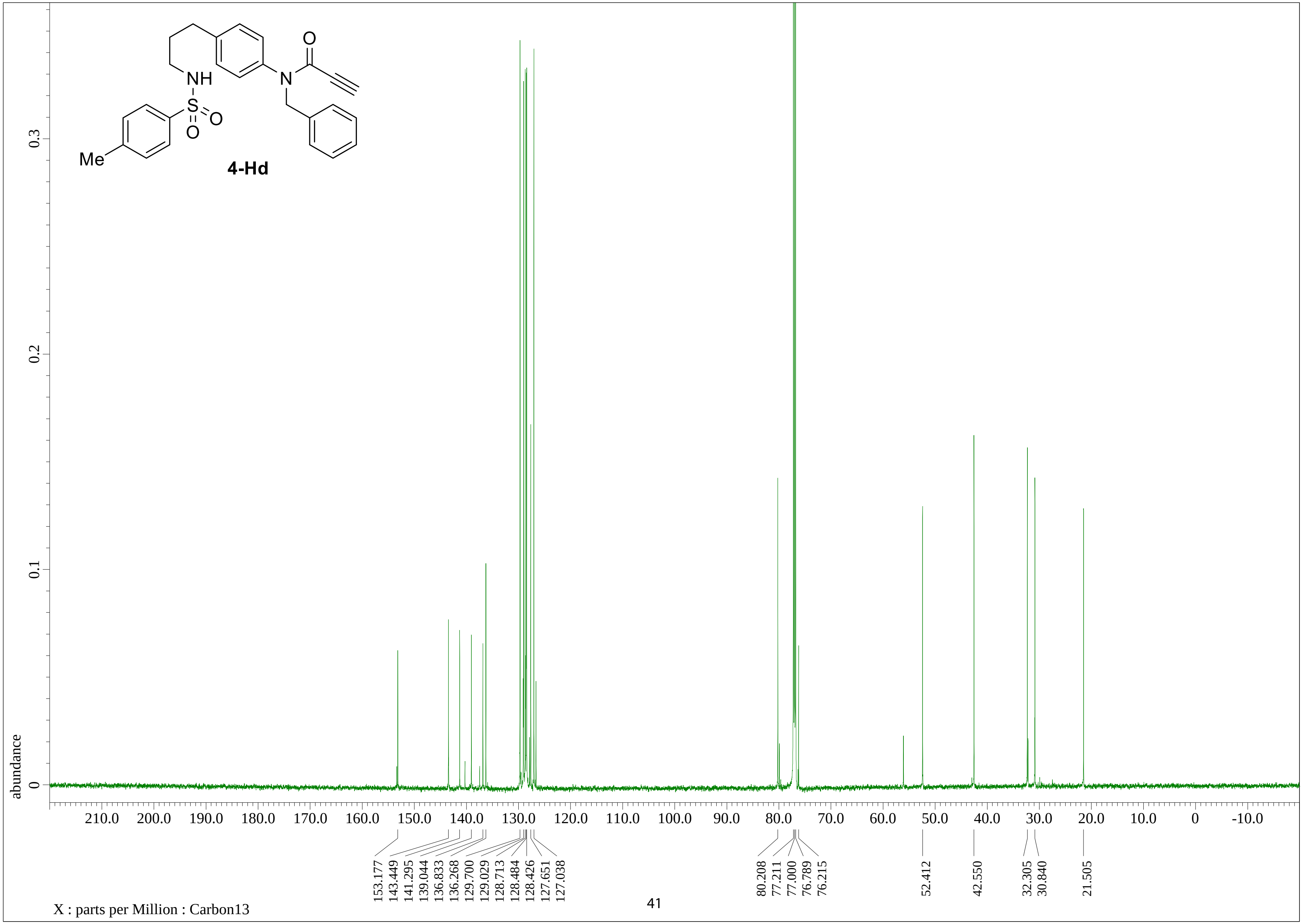
4-Hd
Me
NH
S
O
O
N
O
abundance
0.3
0.2
0.1
0
210.0 200.0 190.0 180.0 170.0 160.0 150.0 140.0 130.0 120.0 110.0 100.0 90.0 80.0 70.0 60.0 50.0 40.0 30.0 20.0 10.0 0 -10.0
153.177
143.449
141.295
139.044
136.833
136.268
129.700
129.029
128.713
128.484
128.426
127.651
127.038
80.208
77.211
77.000
76.789
76.215
52.412
42.550
32.305
30.840
21.505
X : parts per Million : Carbon13

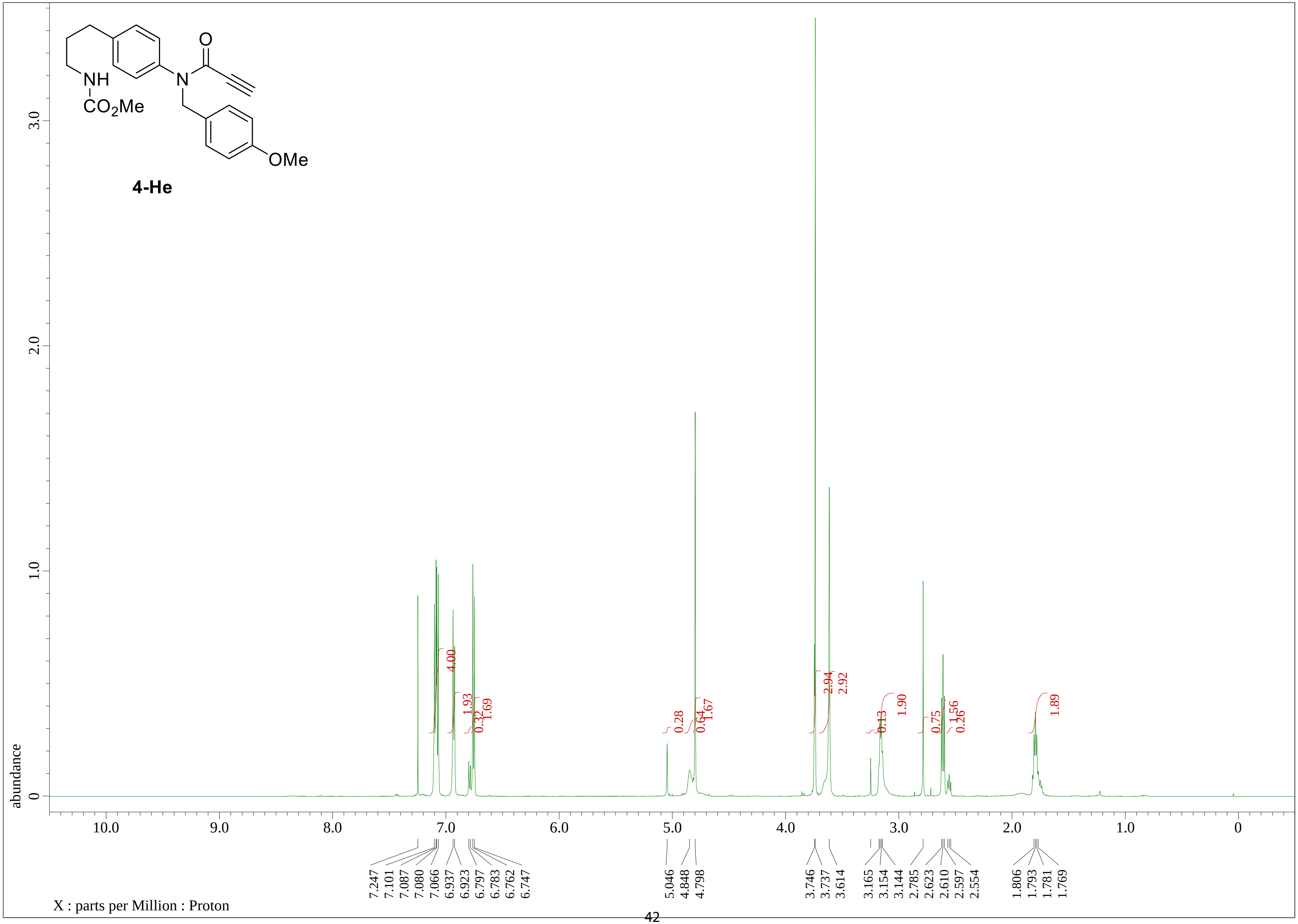
4-He
NH
CO2Me
N
O
OMe
abundance
X : parts per Million : Proton
10.0 9.0 8.0 7.0 6.0 5.0 4.0 3.0 2.0 1.0 0
0 1.0 2.0 3.0
7.247 7.101 7.087 7.080 7.066 6.937 6.923 6.797 6.783 6.762 6.747
5.046 4.848 4.798
3.746 3.737 3.614
3.165 3.154 3.144 2.785 2.623 2.610 2.597 2.554
1.806 1.793 1.781 1.769
4.00 1.93 0.32 1.69 0.28 0.64 1.67 2.94 2.92 0.13 1.90 0.75 1.56 0.26 1.89

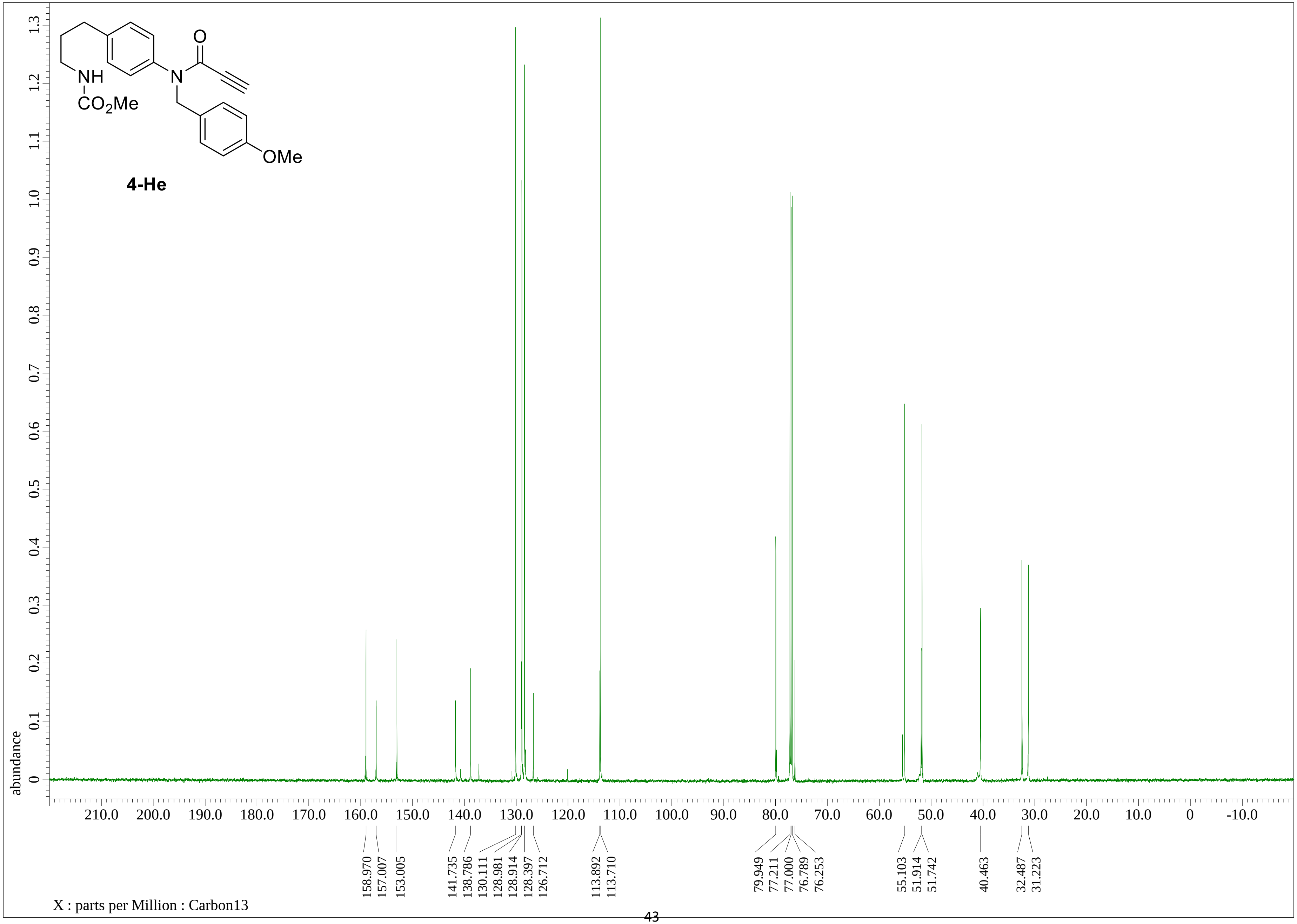

NH
CO2Me
N
O
OMe
4-He
abundance
X : parts per Million : Carbon13
158.970
157.007
153.005
141.735
138.786
130.111
128.981
128.914
128.397
126.712
113.892
113.710
79.949
77.211
77.000
76.789
76.253
55.103
51.914
51.742
40.463
32.487
31.223

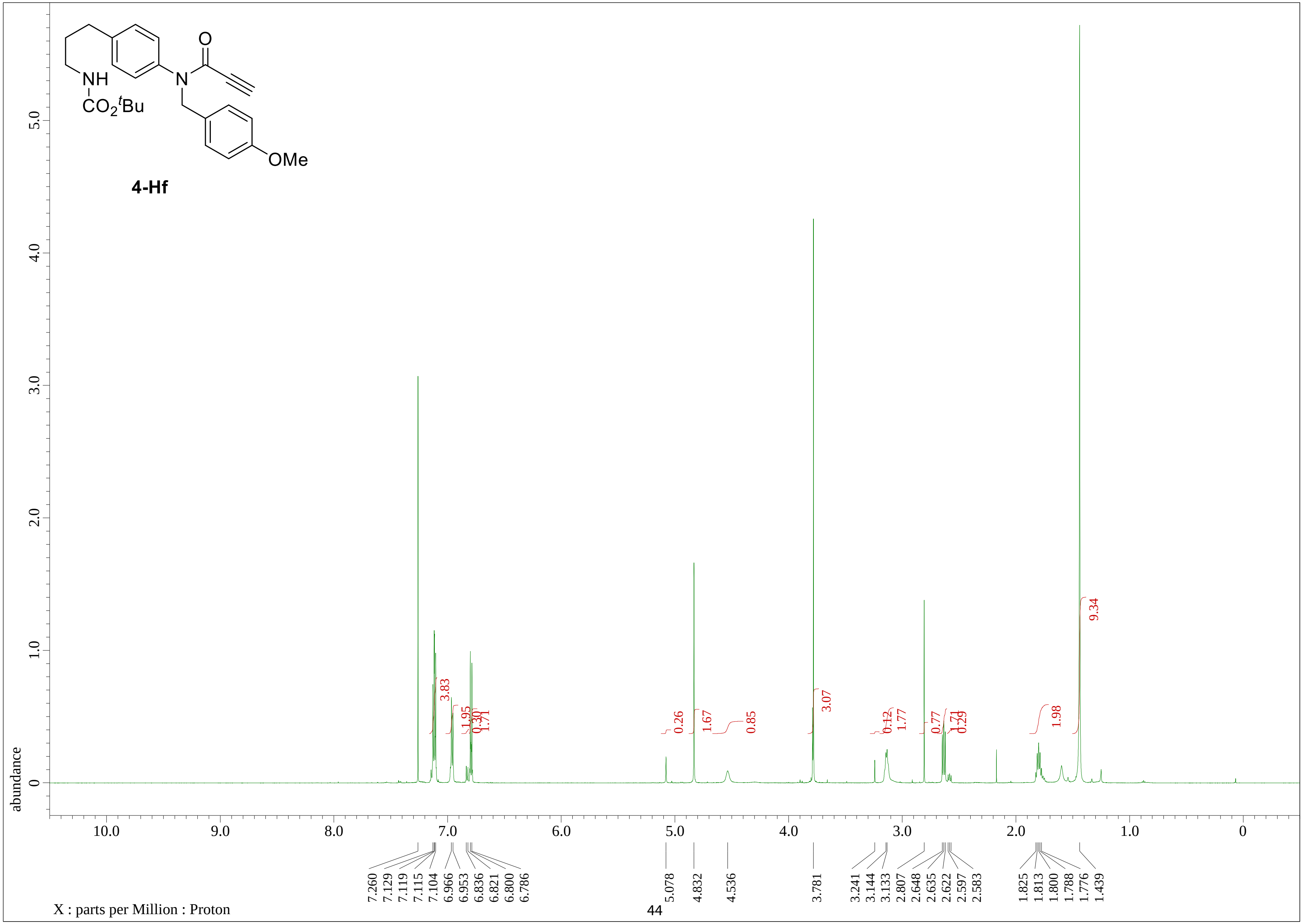

NH
CO2tBu
N
O
OMe
4-Hf
abundance
5.0
4.0
3.0
2.0
1.0
0
3.83
1.95
0.30
1.71
0.26
1.67
0.85
3.07
0.12
1.77
0.77
1.71
0.29
1.98
9.34
10.0
9.0
8.0
7.0
6.0
5.0
4.0
3.0
2.0
1.0
0
7.260
7.129
7.119
7.115
7.104
6.966
6.953
6.836
6.821
6.800
6.786
5.078
4.832
4.536
3.781
3.241
3.144
3.133
2.807
2.648
2.635
2.622
2.597
2.583
1.825
1.813
1.800
1.788
1.776
1.439
X : parts per Million : Proton

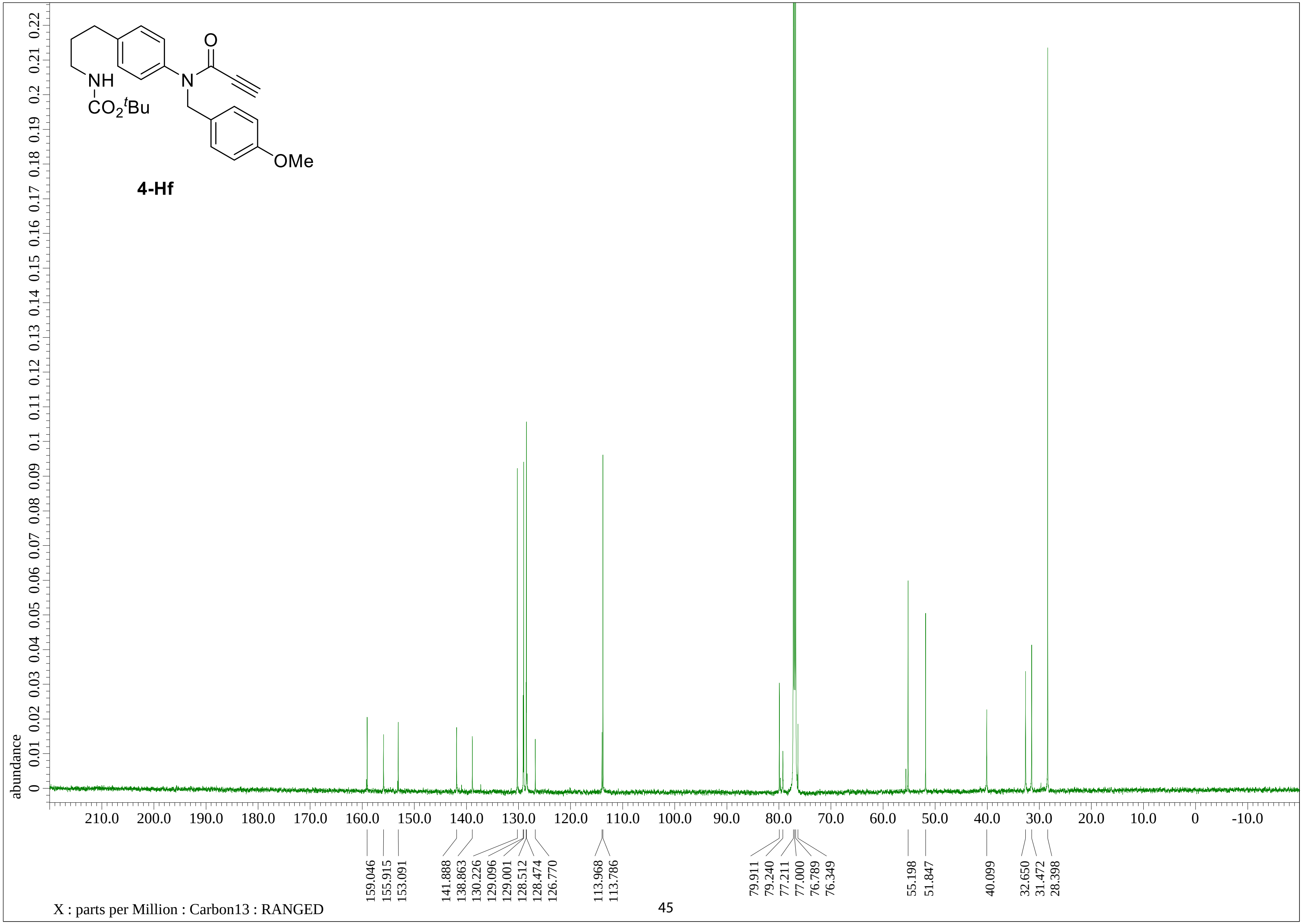

NH
CO2tBu
N
O
OMe
4-Hf
abundance
X : parts per Million : Carbon13 : RANGED
159.046
155.915
153.091
141.888
138.863
130.226
129.096
129.001
128.512
128.474
126.770
113.968
113.786
79.911
79.240
77.211
77.000
76.789
76.349
55.198
51.847
40.099
32.650
31.472
28.398

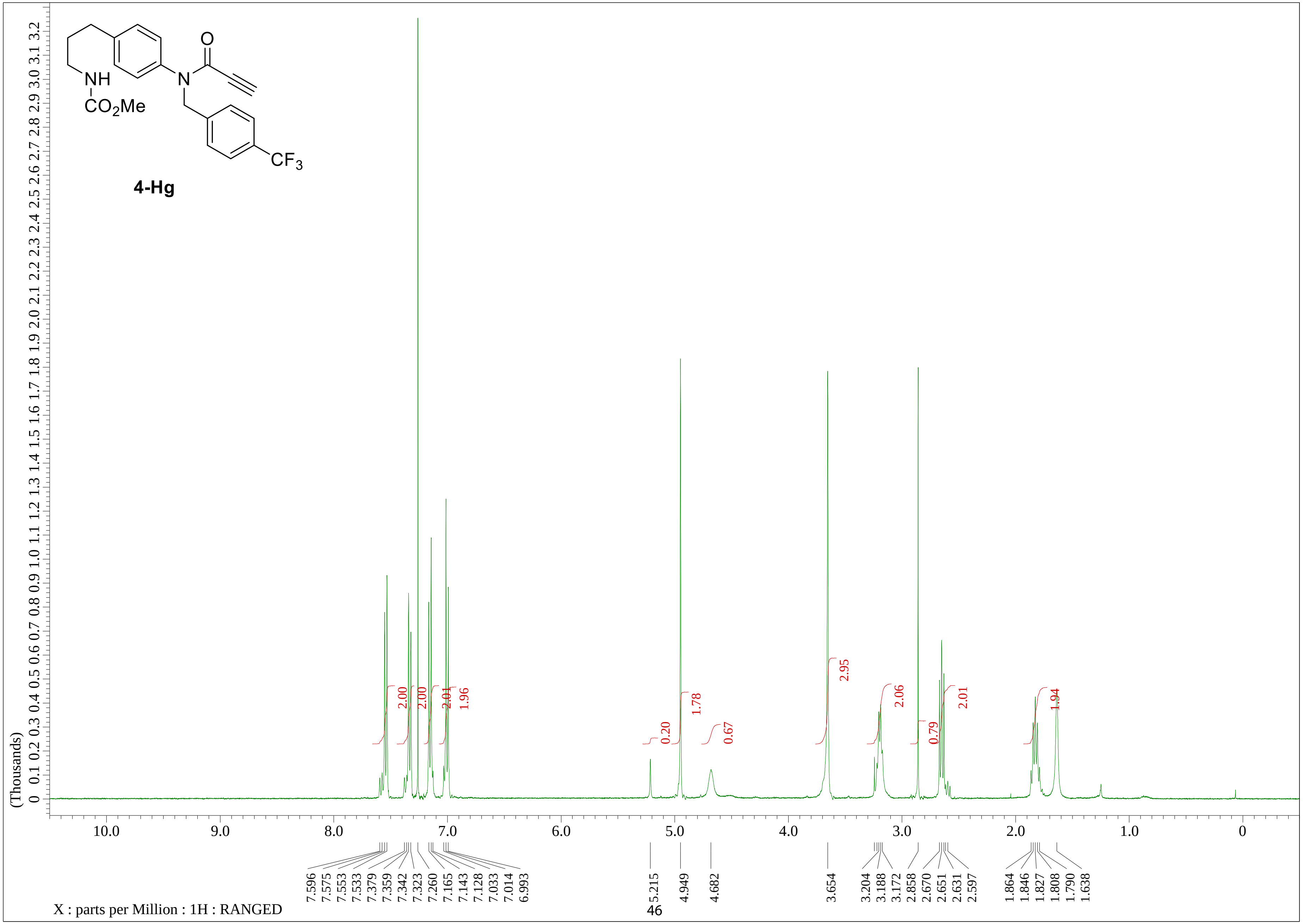

4-Hg
X : parts per Million : 1H : RANGED
(Thousands)

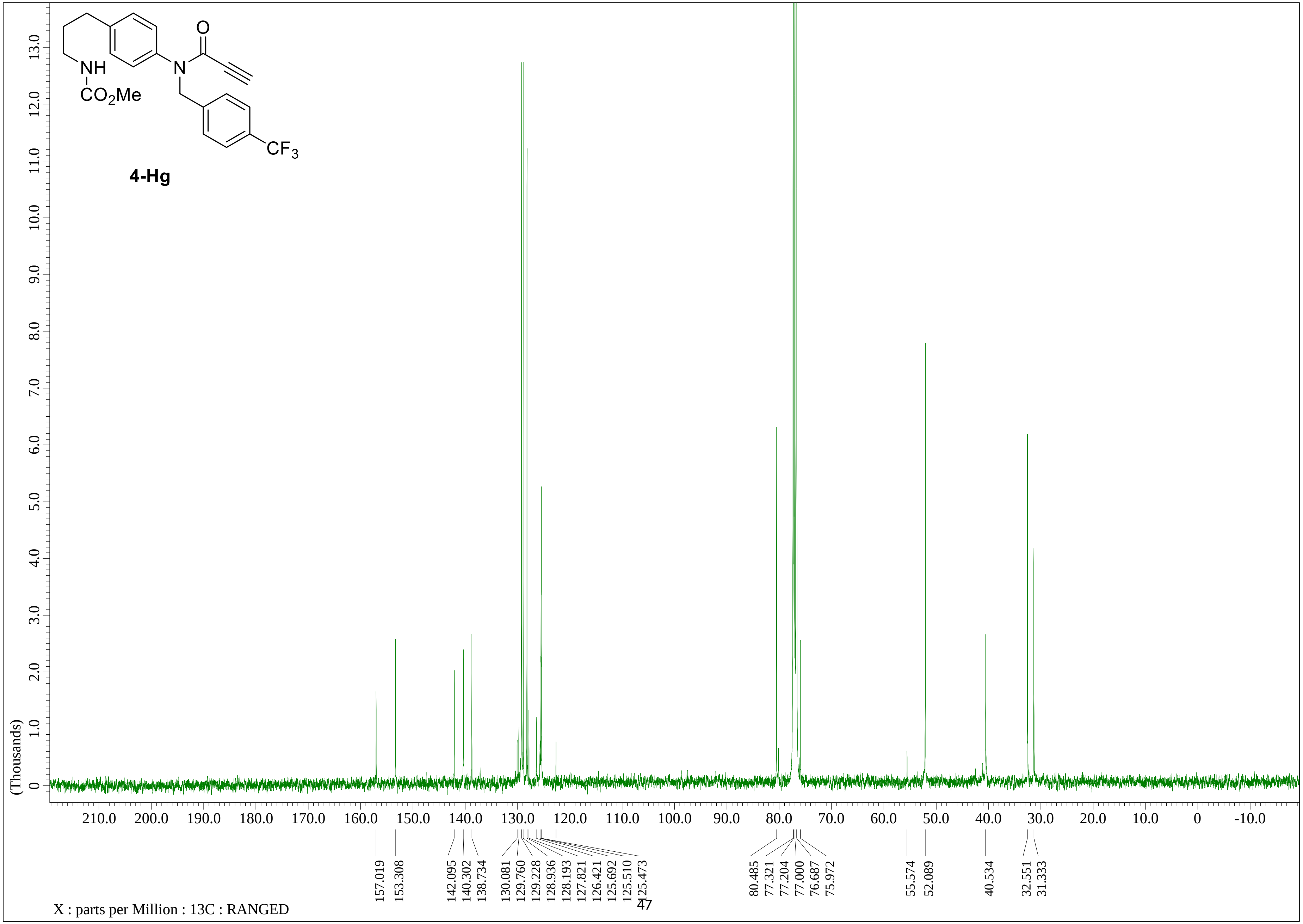

NH
CO2Me
N
O
CF3
4-Hg
(Thousands)
13.0
12.0
11.0
10.0
9.0
8.0
7.0
6.0
5.0
4.0
3.0
2.0
1.0
0
210.0 200.0 190.0 180.0 170.0 160.0 150.0 140.0 130.0 120.0 110.0 100.0 90.0 80.0 70.0 60.0 50.0 40.0 30.0 20.0 10.0 0 -10.0
157.019
153.308
142.095
140.302
138.734
130.081
129.760
129.228
128.936
128.193
127.821
126.421
125.692
125.510
125.473
80.485
77.321
77.204
77.000
76.687
75.972
55.574
52.089
40.534
32.551
31.333
X : parts per Million : 13C : RANGED

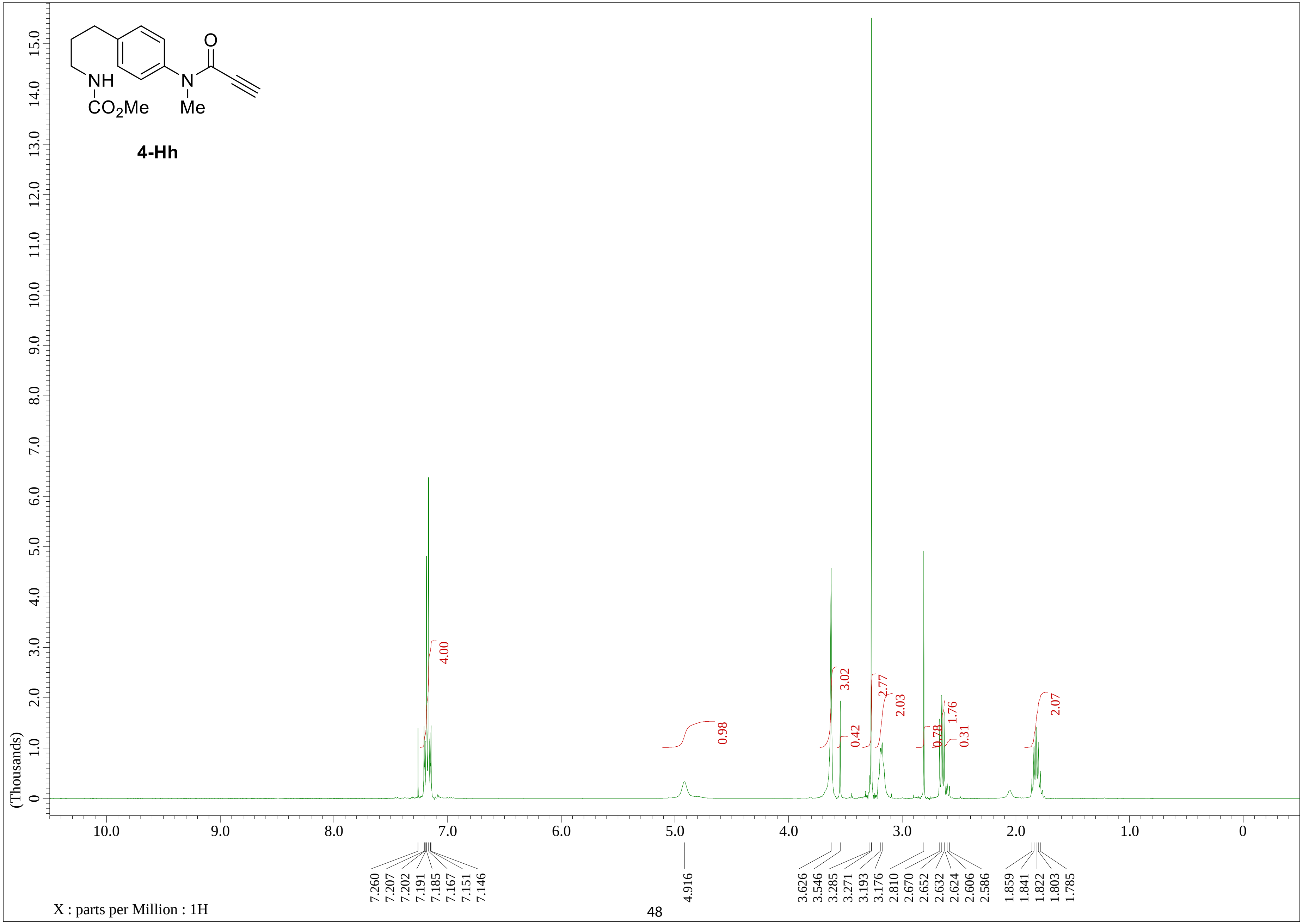
NH
CO2Me
N
Me
O
4-Hh
(Thousands)
X : parts per Million : 1H
4.00
0.98
3.02
0.42
2.77
2.03
0.78
1.76
0.31
2.07
7.260
7.207
7.202
7.191
7.185
7.167
7.151
7.146
4.916
3.626
3.546
3.285
3.271
3.193
3.176
2.810
2.670
2.652
2.632
2.624
2.606
2.586
1.859
1.841
1.822
1.803
1.785

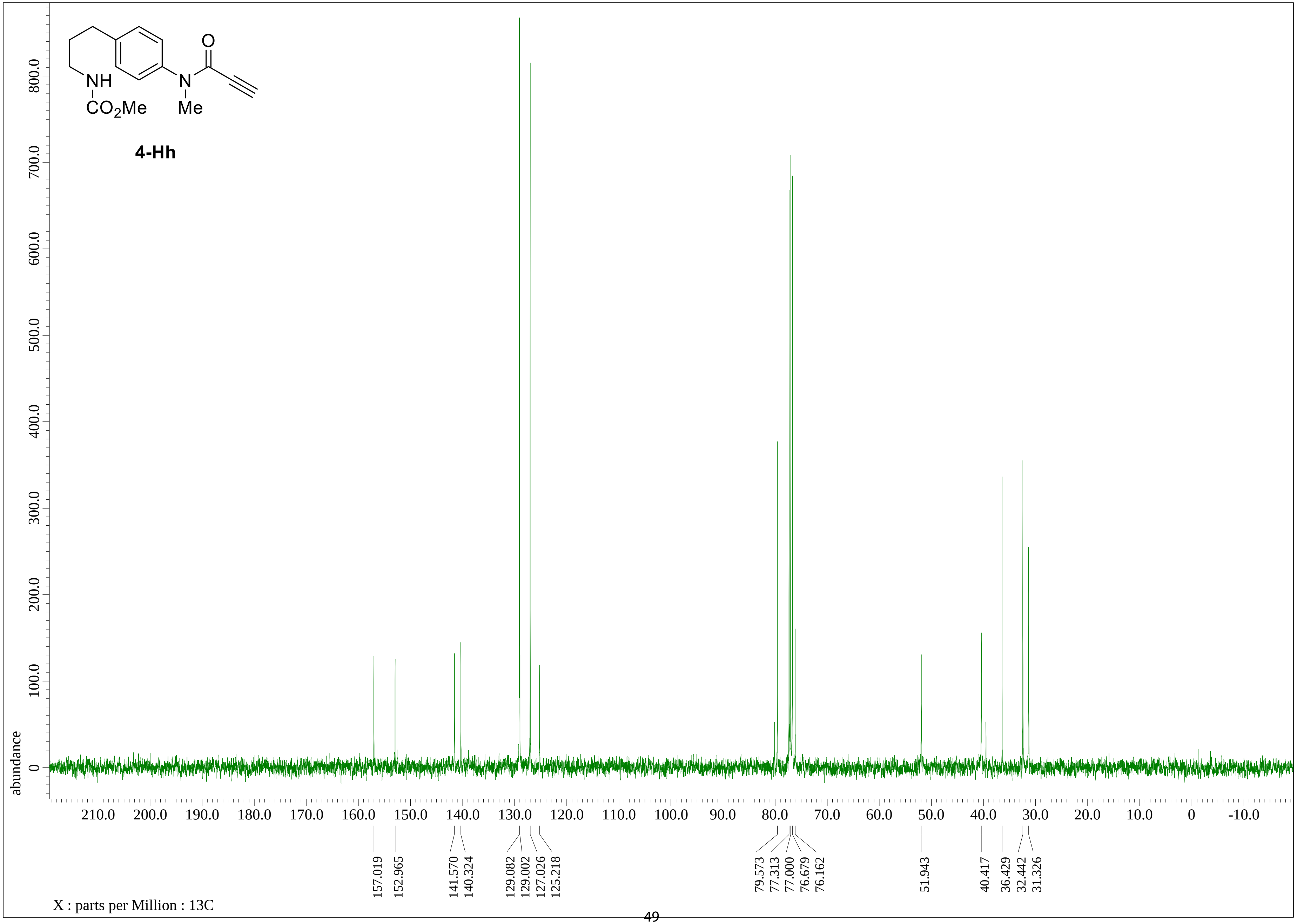

NH
CO2Me
N
Me
O
4-Hh
abundance
X : parts per Million : 13C
157.019
152.965
141.570
140.324
129.082
129.002
127.026
125.218
79.573
77.313
77.000
76.679
76.162
51.943
40.417
36.429
32.442
31.326

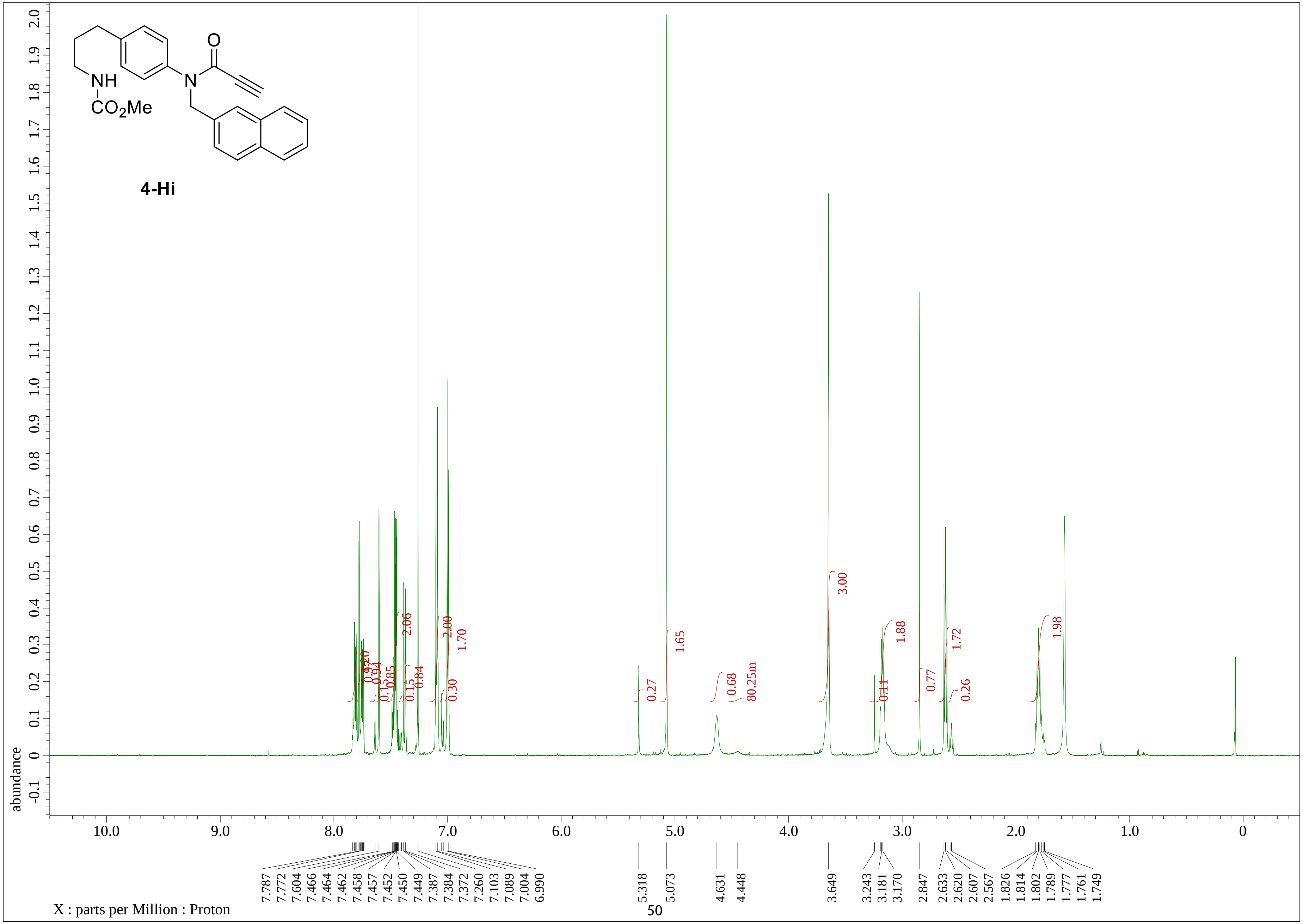
4-Hi
X : parts per Million : Proton
abundance
7.787
7.772
7.604
7.466
7.464
7.462
7.458
7.457
7.452
7.450
7.449
7.387
7.384
7.372
7.260
7.103
7.089
7.004
6.990
5.318
5.073
4.631
4.448
3.649
3.243
3.181
3.170
2.847
2.633
2.620
2.607
2.567
1.826
1.814
1.802
1.789
1.777
1.761
1.749

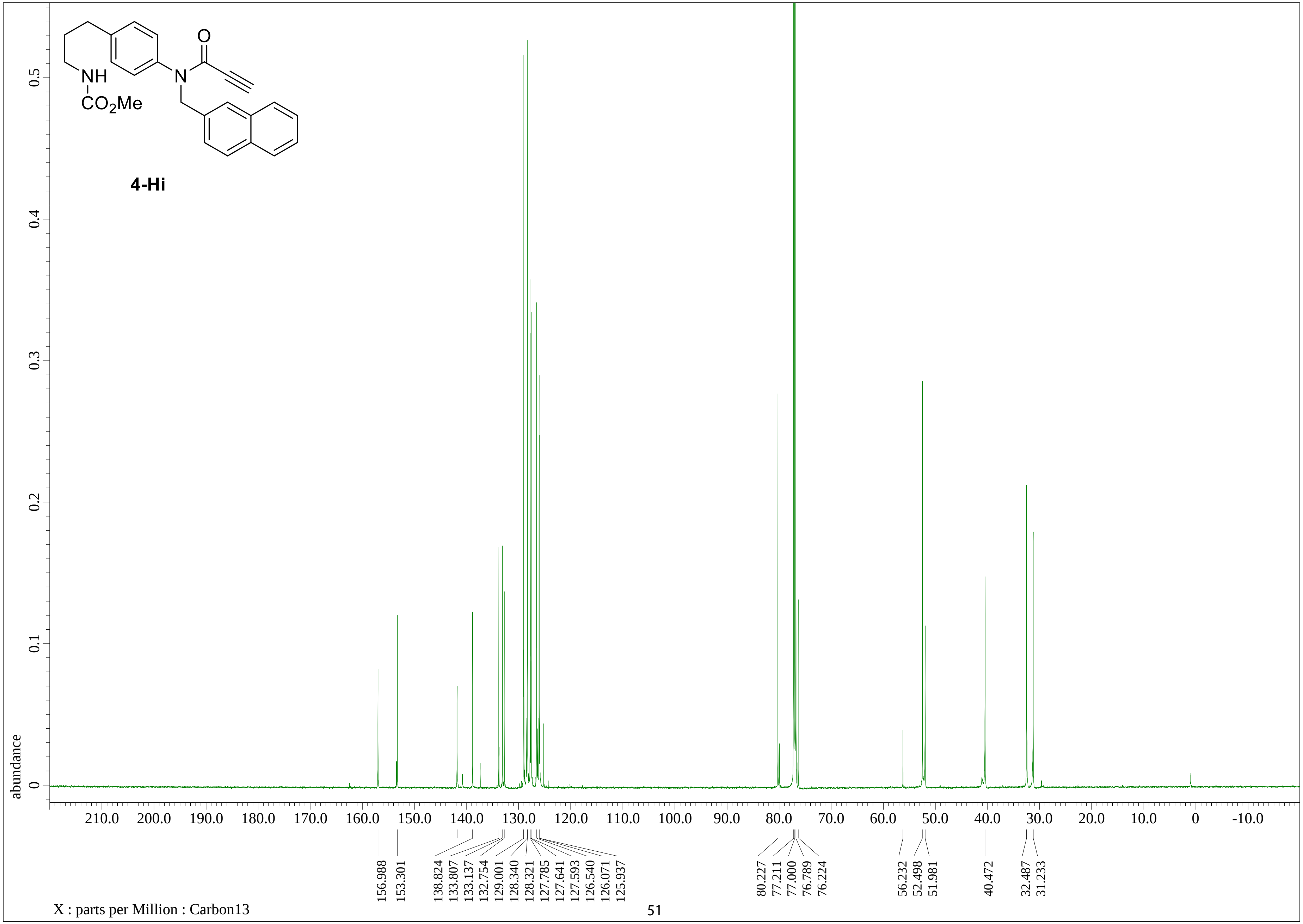
NH
CO2Me
O
N
4-Hi
abundance
0.5
0.4
0.3
0.2
0.1
0
210.0 200.0 190.0 180.0 170.0 160.0 150.0 140.0 130.0 120.0 110.0 100.0 90.0 80.0 70.0 60.0 50.0 40.0 30.0 20.0 10.0 0 -10.0
156.988
153.301
138.824
133.807
133.137
132.754
129.001
128.340
128.321
127.785
127.641
127.593
126.540
126.071
125.937
80.227
77.211
77.000
76.789
76.224
56.232
52.498
51.981
40.472
32.487
31.233
X : parts per Million : Carbon13

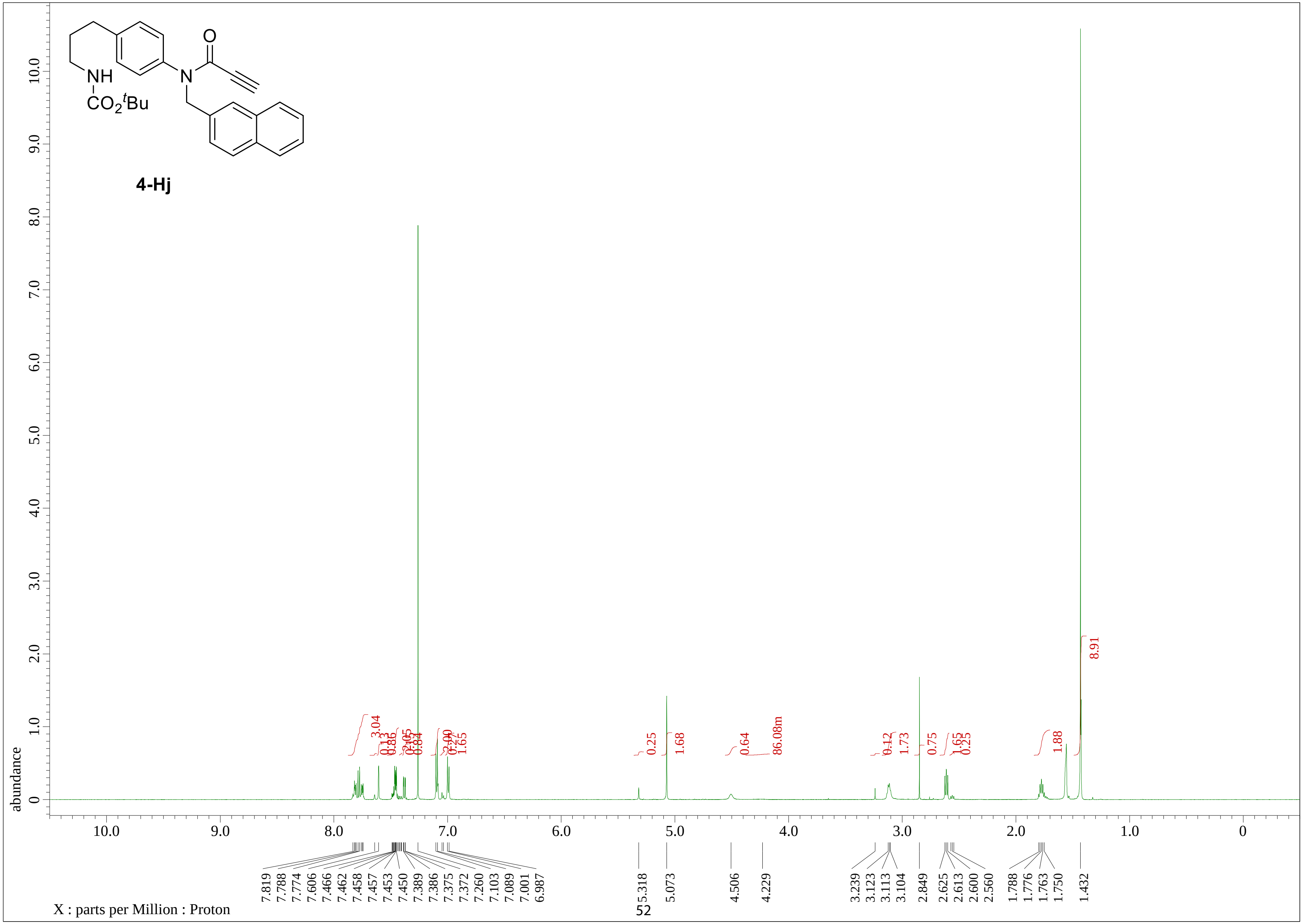

NH
$CO_2{}^tBu$
N
O
4-Hj
abundance
X : parts per Million : Proton
7.819 7.788 7.774 7.606 7.466 7.462 7.458 7.457 7.453 7.450 7.389 7.386 7.375 7.372 7.260 7.103 7.089 7.001 6.987
5.318 5.073 4.506 4.229
3.239 3.123 3.113 3.104 2.849 2.625 2.613 2.600 2.560 1.788 1.776 1.763 1.750 1.432
8.91 1.88 1.73 0.75 0.64 86.08m 0.25 1.68 3.04 1.65

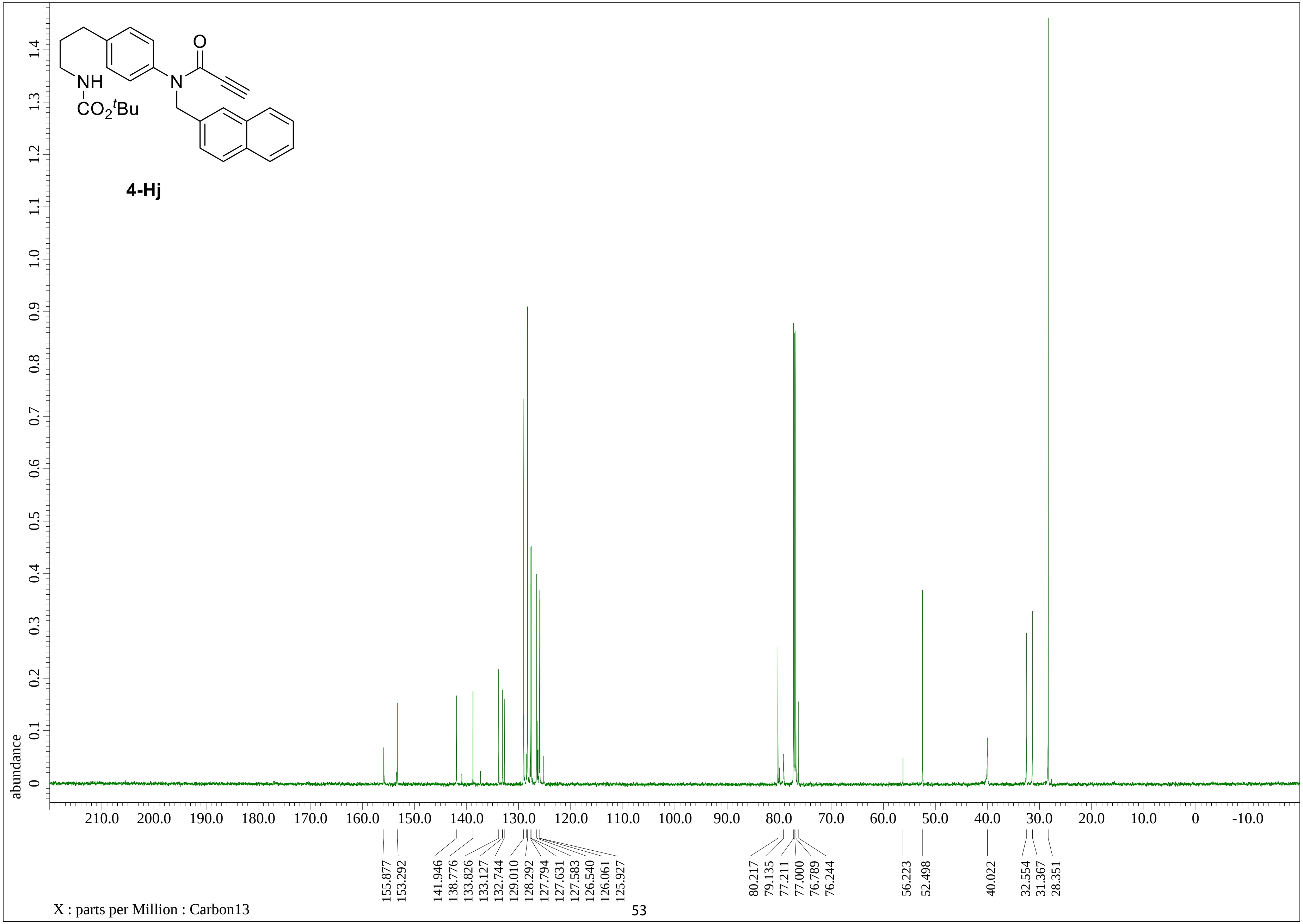
NH
CO2tBu
N
O
4-Hj
abundance
X : parts per Million : Carbon13
155.877
153.292
141.946
138.776
133.826
133.127
132.744
129.010
128.292
127.794
127.631
127.583
126.540
126.061
125.927
80.217
79.135
77.211
77.000
76.789
76.244
56.223
52.498
40.022
32.554
31.367
28.351

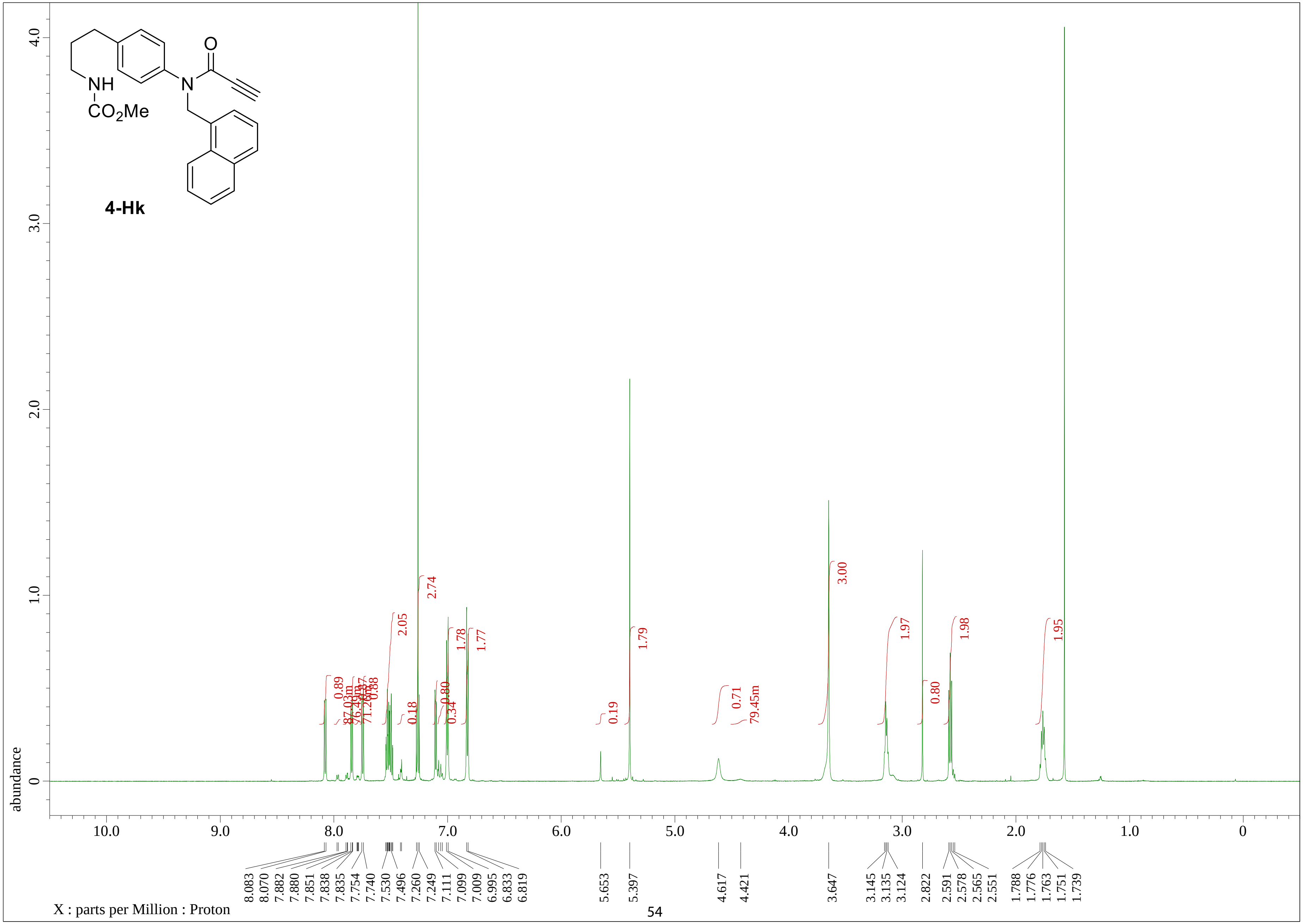
NH
CO2Me
N
O
4-Hk
abundance
X : parts per Million : Proton
0.89
2.05
0.18
2.74
0.80
0.34
1.78
1.77
0.19
1.79
0.71
79.45m
3.00
1.97
0.80
1.98
1.95
8.083
8.070
7.882
7.880
7.851
7.838
7.835
7.754
7.740
7.530
7.496
7.260
7.249
7.111
7.099
7.009
6.995
6.833
6.819
5.653
5.397
4.617
4.421
3.647
3.145
3.135
3.124
2.822
2.591
2.578
2.565
2.551
1.788
1.776
1.763
1.751
1.739

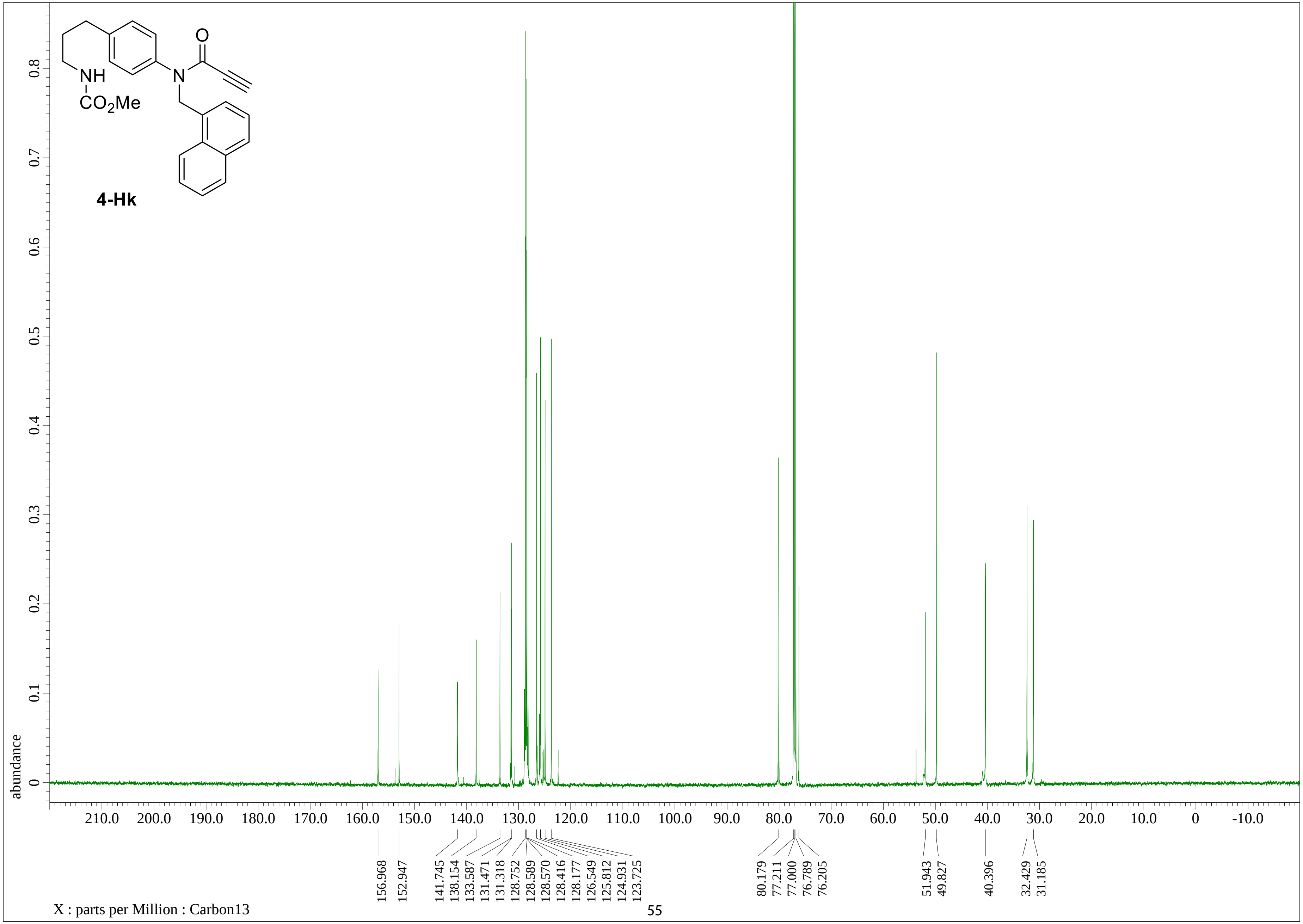
NH
CO2Me
N
O
4-Hk
abundance
0.8
0.7
0.6
0.5
0.4
0.3
0.2
0.1
0
210.0 200.0 190.0 180.0 170.0 160.0 150.0 140.0 130.0 120.0 110.0 100.0 90.0 80.0 70.0 60.0 50.0 40.0 30.0 20.0 10.0 0 -10.0
156.968
152.947
141.745
138.154
133.587
131.471
131.318
128.752
128.589
128.570
128.416
128.177
126.549
125.812
124.931
123.725
80.179
77.211
77.000
76.789
76.205
51.943
49.827
40.396
32.429
31.185
X : parts per Million : Carbon13

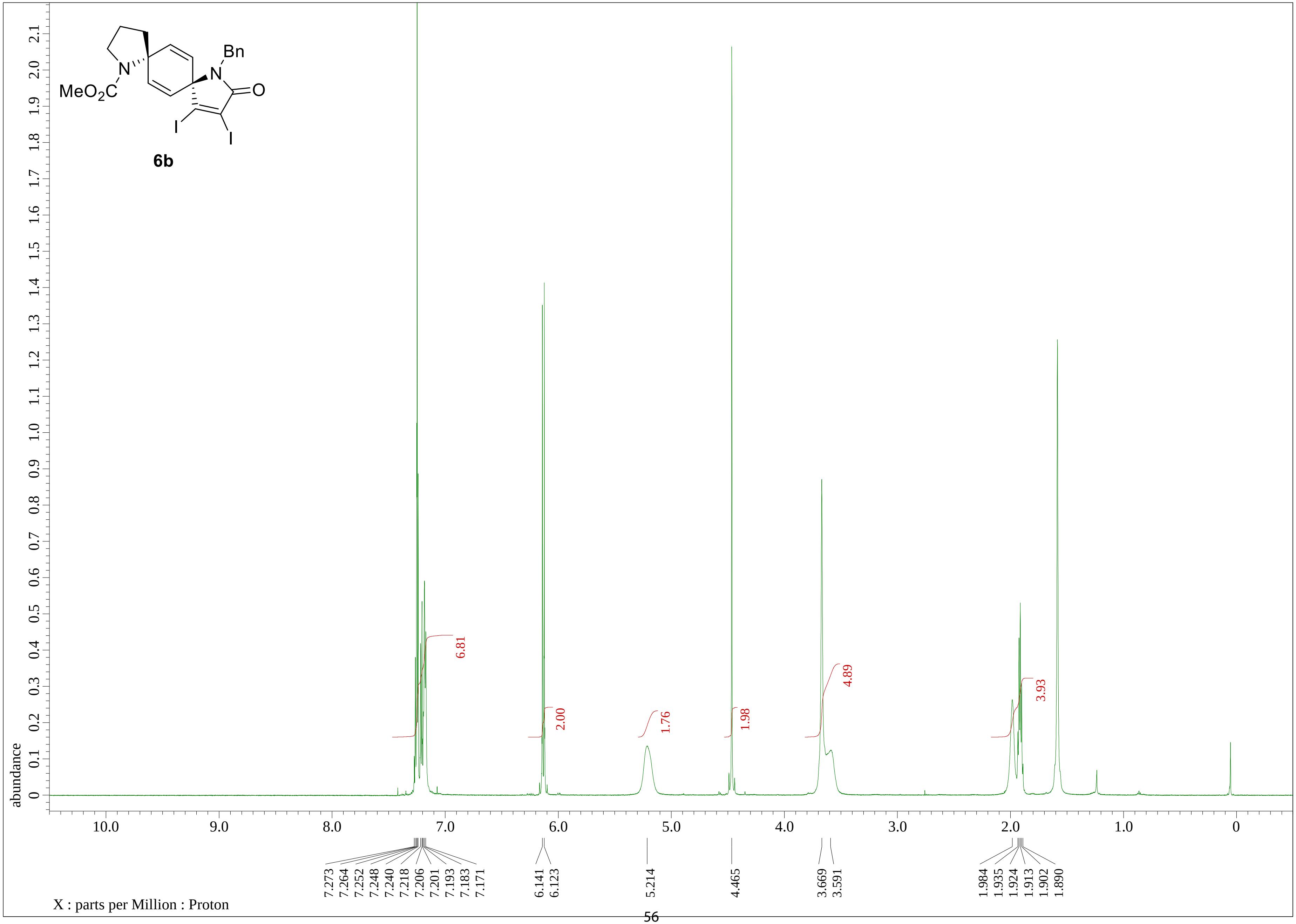

MeO2C
N
Bn
N
O
I
I
6b
abundance
X : parts per Million : Proton
6.81
2.00
1.76
1.98
4.89
3.93
7.273
7.264
7.252
7.248
7.240
7.218
7.206
7.201
7.193
7.183
7.171
6.141
6.123
5.214
4.465
3.669
3.591
1.984
1.935
1.924
1.913
1.902
1.890

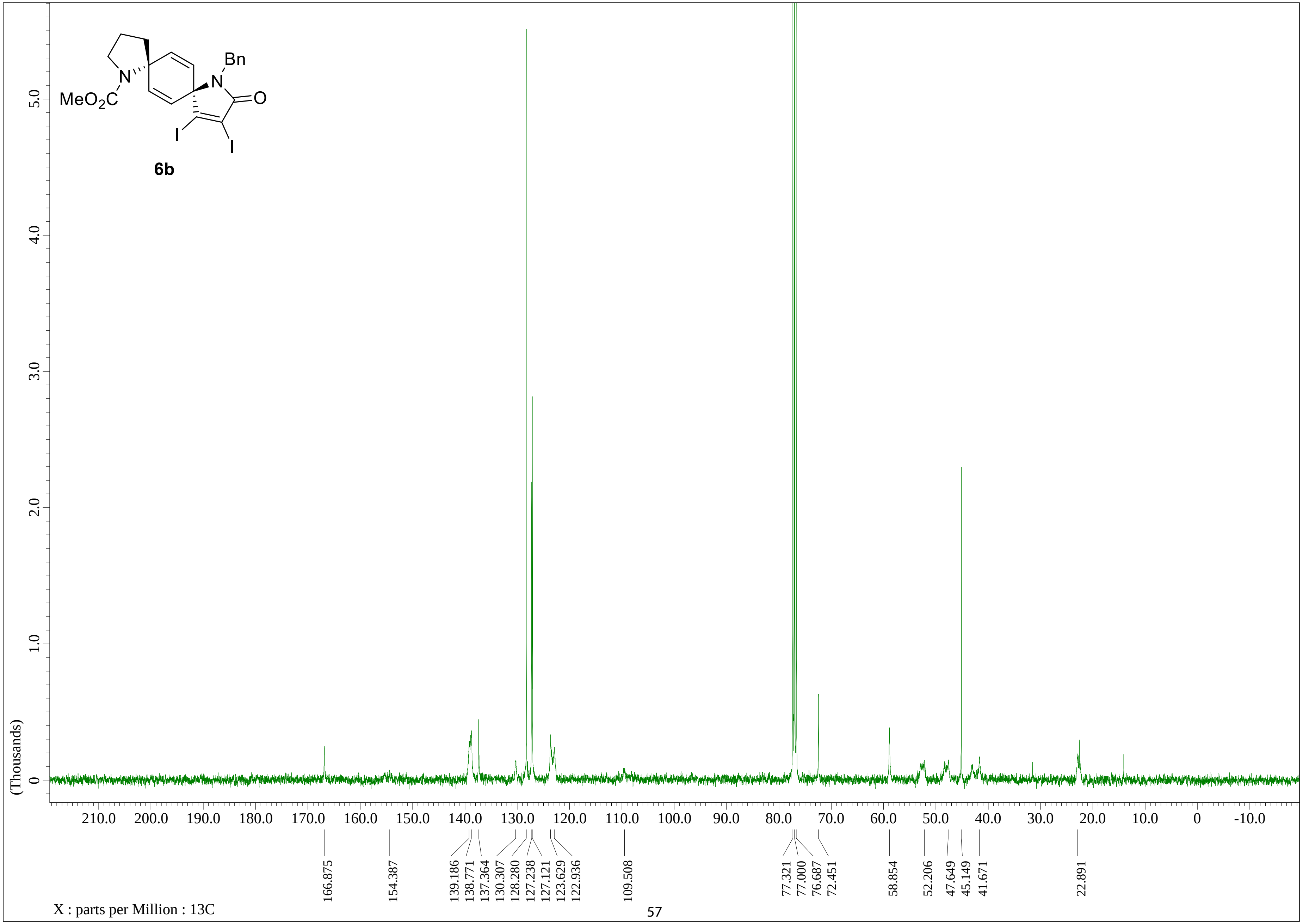
MeO2C
N
Bn
N
O
I
I
6b
(Thousands)
X : parts per Million : 13C
166.875
154.387
139.186
138.771
137.364
130.307
128.280
127.238
127.121
123.629
122.936
109.508
77.321
77.000
76.687
72.451
58.854
52.206
47.649
45.149
41.671
22.891

NOESY

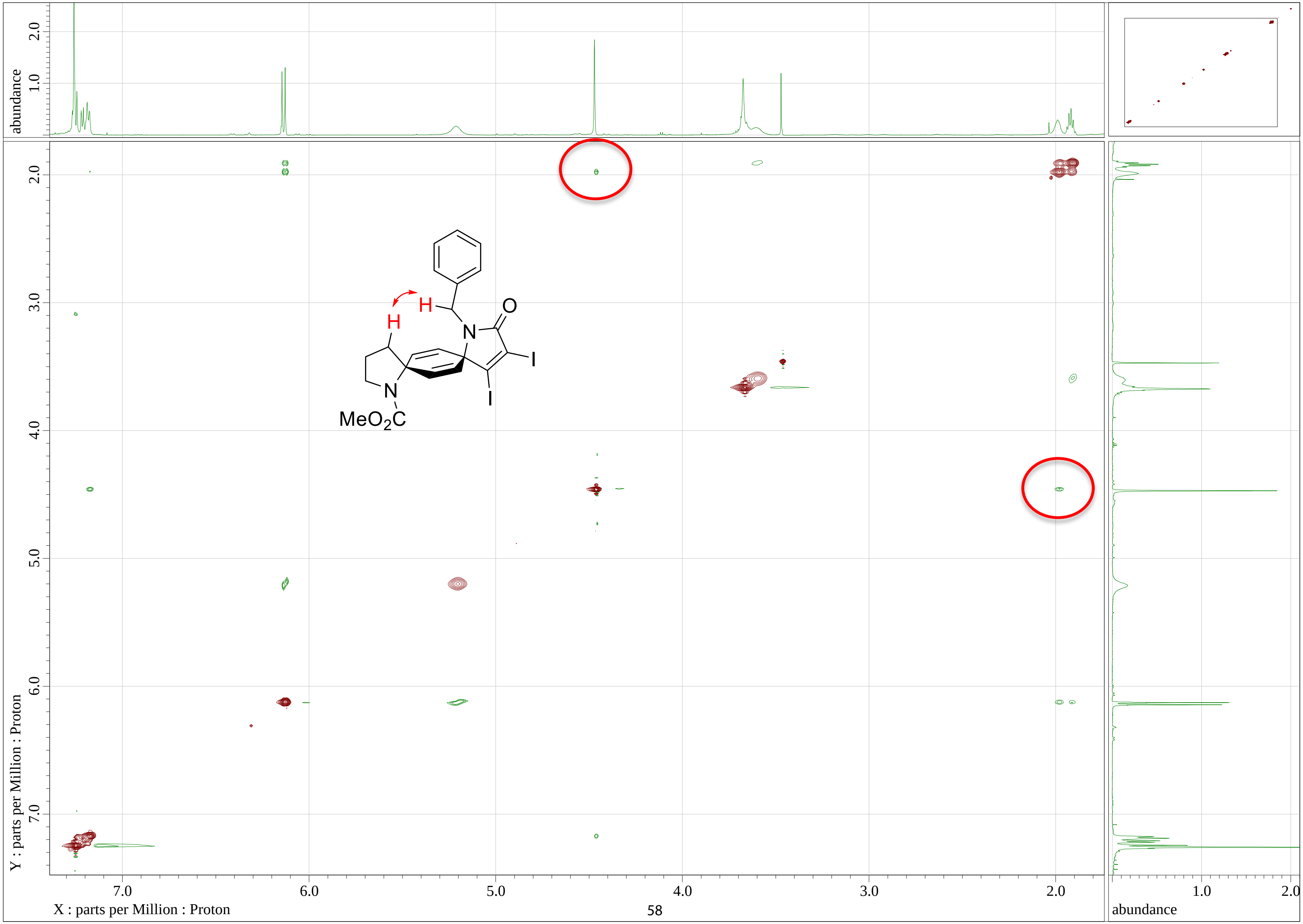
abundance
2.0
1.0
2.0
3.0
4.0
5.0
6.0
7.0
Y : parts per Million : Proton
7.0
6.0
5.0
4.0
3.0
2.0
X : parts per Million : Proton
1.0
2.0
abundance
H
H
O
N
N
I
I
MeO2C

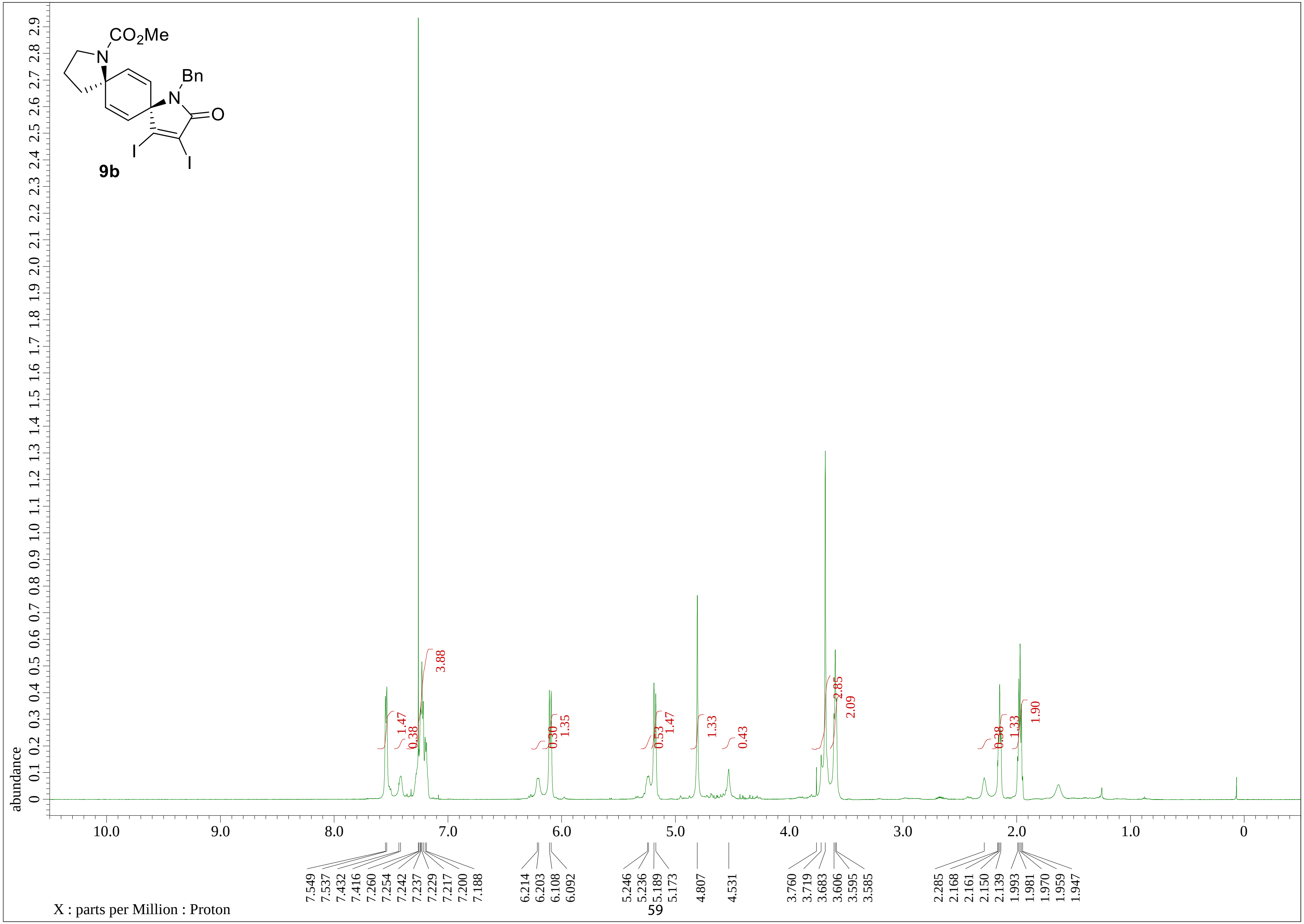

CO2Me
N
Bn
N
O
I
I
9b
abundance
X : parts per Million : Proton
7.549
7.537
7.432
7.416
7.260
7.254
7.242
7.237
7.229
7.217
7.200
7.188
6.214
6.203
6.108
6.092
5.246
5.236
5.189
5.173
4.807
4.531
3.760
3.719
3.683
3.606
3.595
3.585
2.285
2.168
2.161
2.150
2.139
1.993
1.981
1.970
1.959
1.947
3.88
1.47
0.38
0.30
1.35
0.53
1.47
1.33
0.43
2.85
2.09
0.38
1.33
1.90

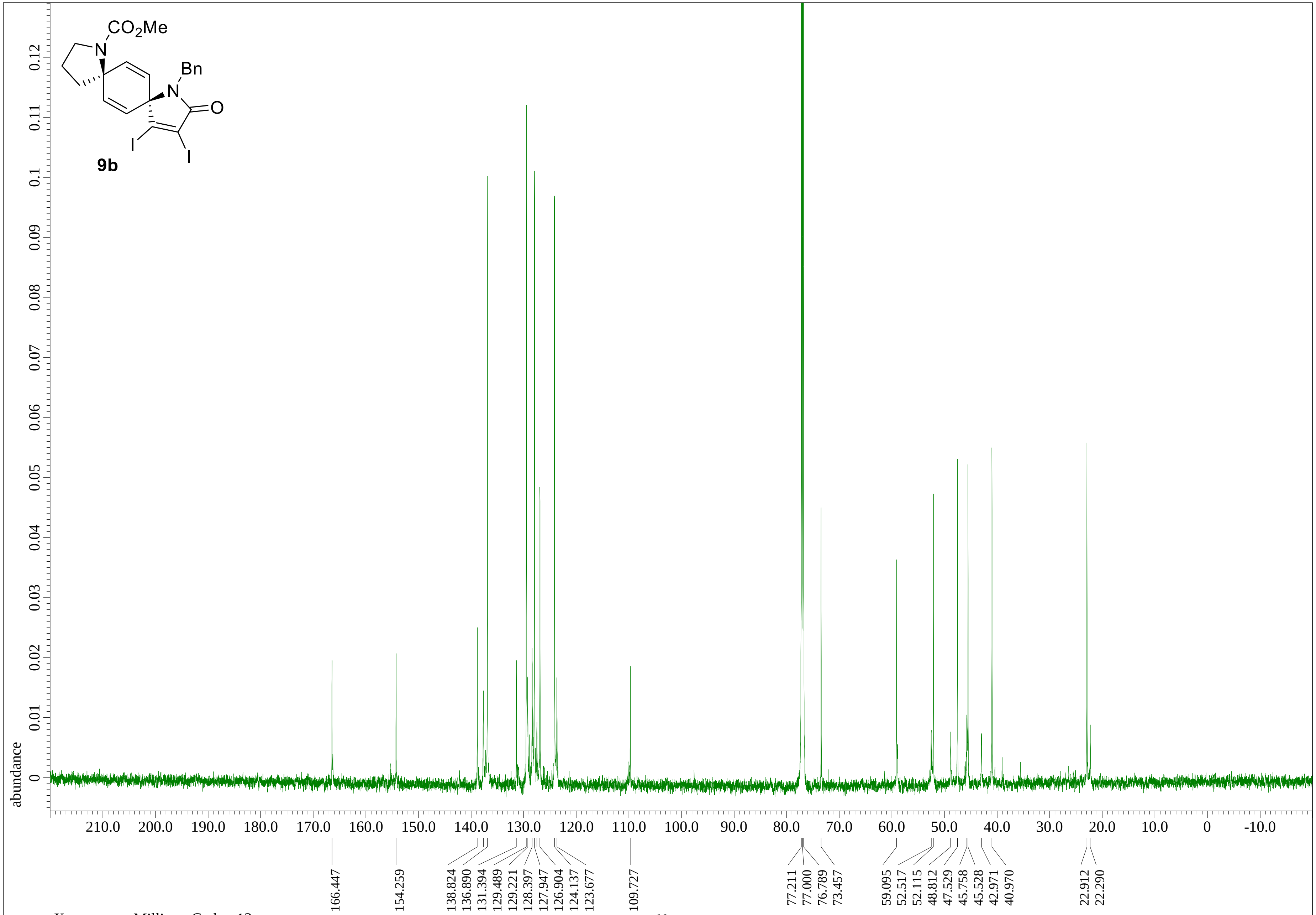

CO2Me
N
Bn
N
O
I
I
9b
abundance
X : parts per Million : Carbon13
166.447
154.259
138.824
136.890
131.394
129.489
129.221
128.397
127.947
126.904
124.137
123.677
109.727
77.211
77.000
76.789
73.457
59.095
52.517
52.115
48.812
47.529
45.758
45.528
42.971
40.970
22.912
22.290

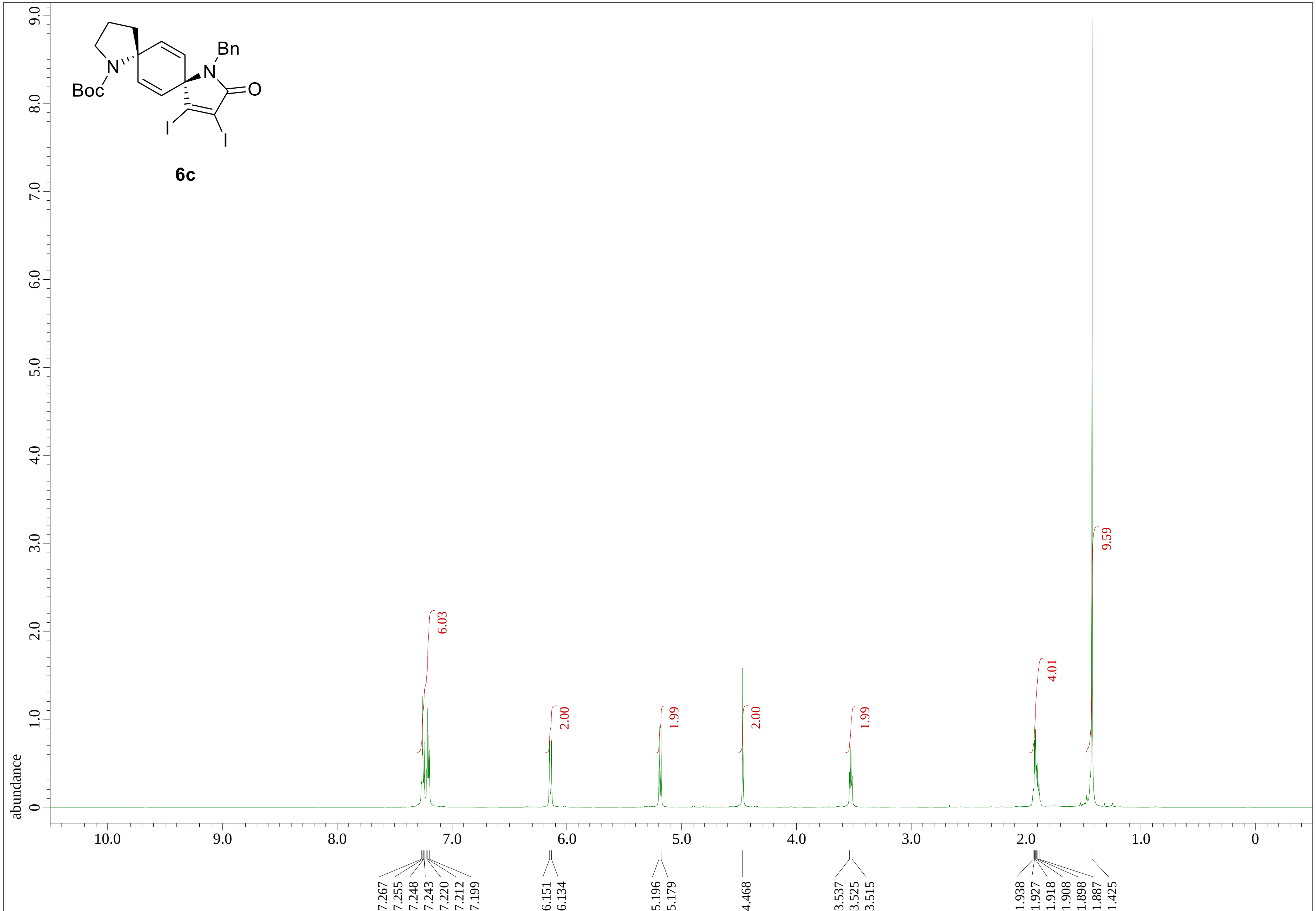

Bn
N
Boc
O
I
6c
abundance
9.0
8.0
7.0
6.0
5.0
4.0
3.0
2.0
1.0
0
10.0
9.0
8.0
7.0
6.0
5.0
4.0
3.0
2.0
1.0
0
6.03
2.00
1.99
2.00
1.99
4.01
9.59
7.267
7.255
7.248
7.243
7.220
7.212
7.199
6.151
6.134
5.196
5.179
4.468
3.537
3.525
3.515
1.938
1.927
1.918
1.908
1.898
1.887
1.425
X : parts per Million : Proton

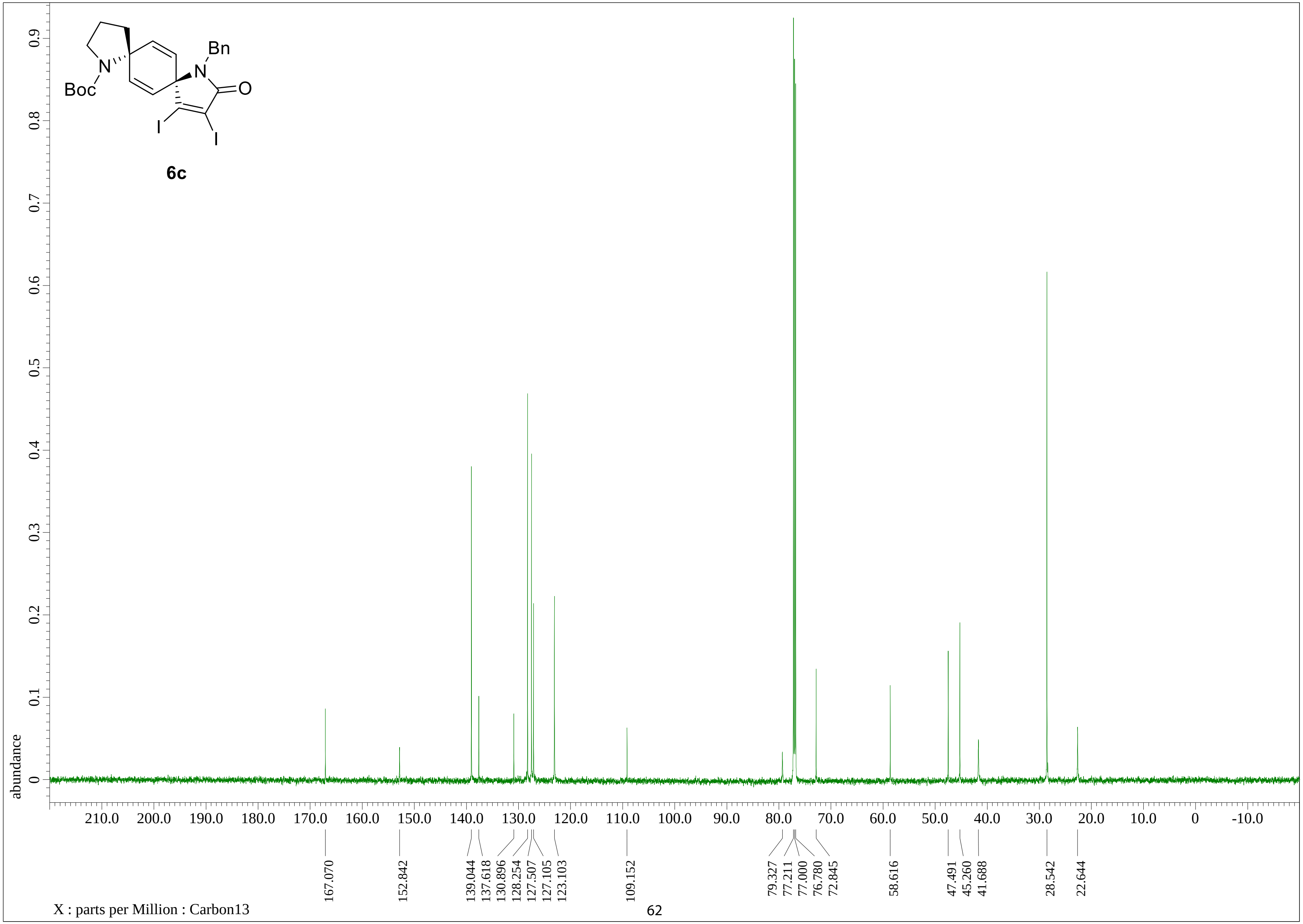
6c
Bn
N
O
Boc
I
abundance
0.9
0.8
0.7
0.6
0.5
0.4
0.3
0.2
0.1
0
210.0 200.0 190.0 180.0 170.0 160.0 150.0 140.0 130.0 120.0 110.0 100.0 90.0 80.0 70.0 60.0 50.0 40.0 30.0 20.0 10.0 0 -10.0
167.070
152.842
139.044
137.618
130.896
128.254
127.507
127.105
123.103
109.152
79.327
77.211
77.000
76.780
72.845
58.616
47.491
45.260
41.688
28.542
22.644
X : parts per Million : Carbon13

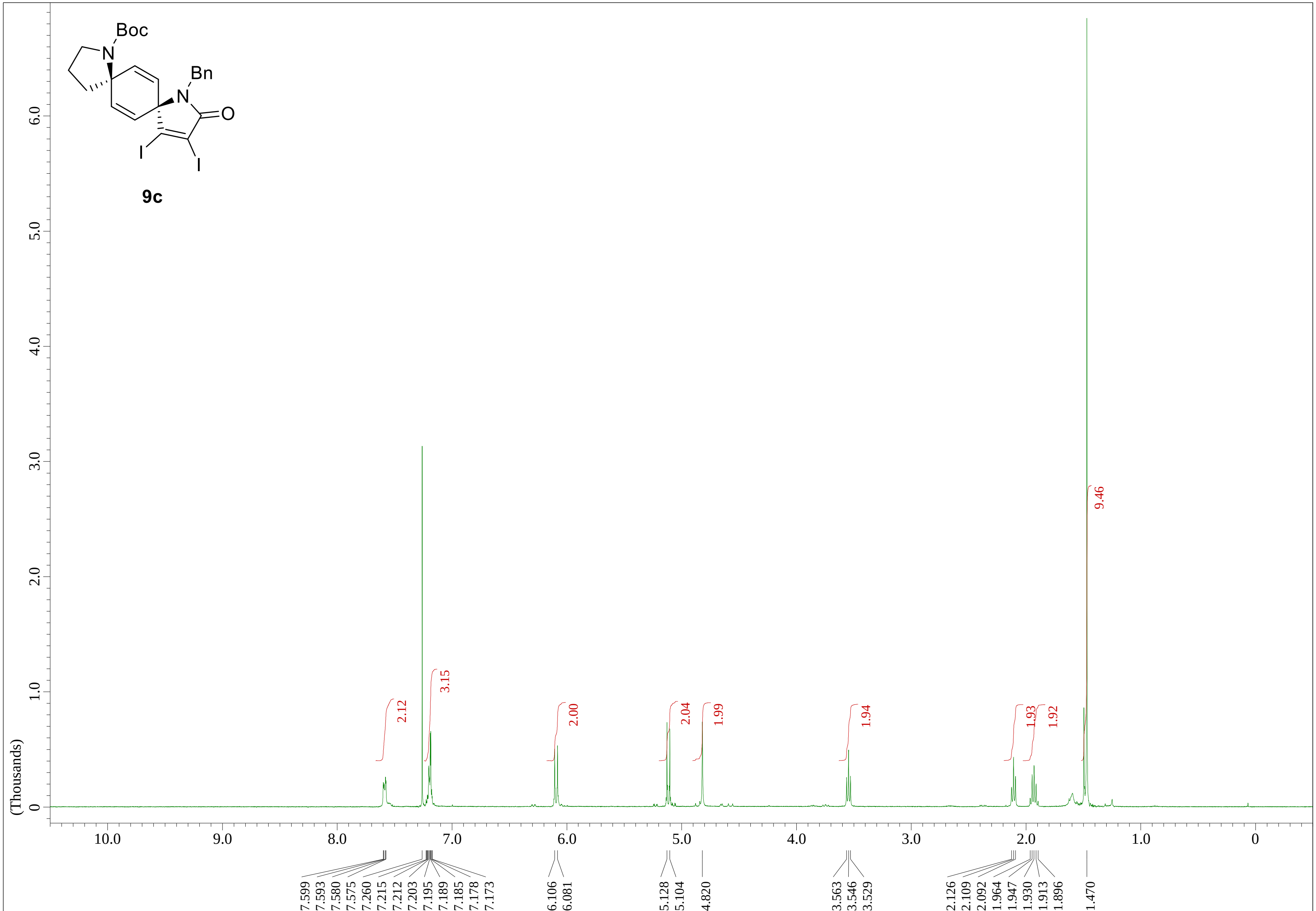
Boc
Bn
N
O
I
9c
(Thousands)
0
1.0
2.0
3.0
4.0
5.0
6.0
2.12
3.15
2.00
2.04
1.99
1.94
1.93
1.92
9.46
10.0
9.0
8.0
7.0
6.0
5.0
4.0
3.0
2.0
1.0
0
7.599
7.593
7.580
7.575
7.260
7.215
7.212
7.203
7.195
7.189
7.185
7.178
7.173
6.106
6.081
5.128
5.104
4.820
3.563
3.546
3.529
2.126
2.109
2.092
1.964
1.947
1.930
1.913
1.896
1.470
X : parts per Million : 1H

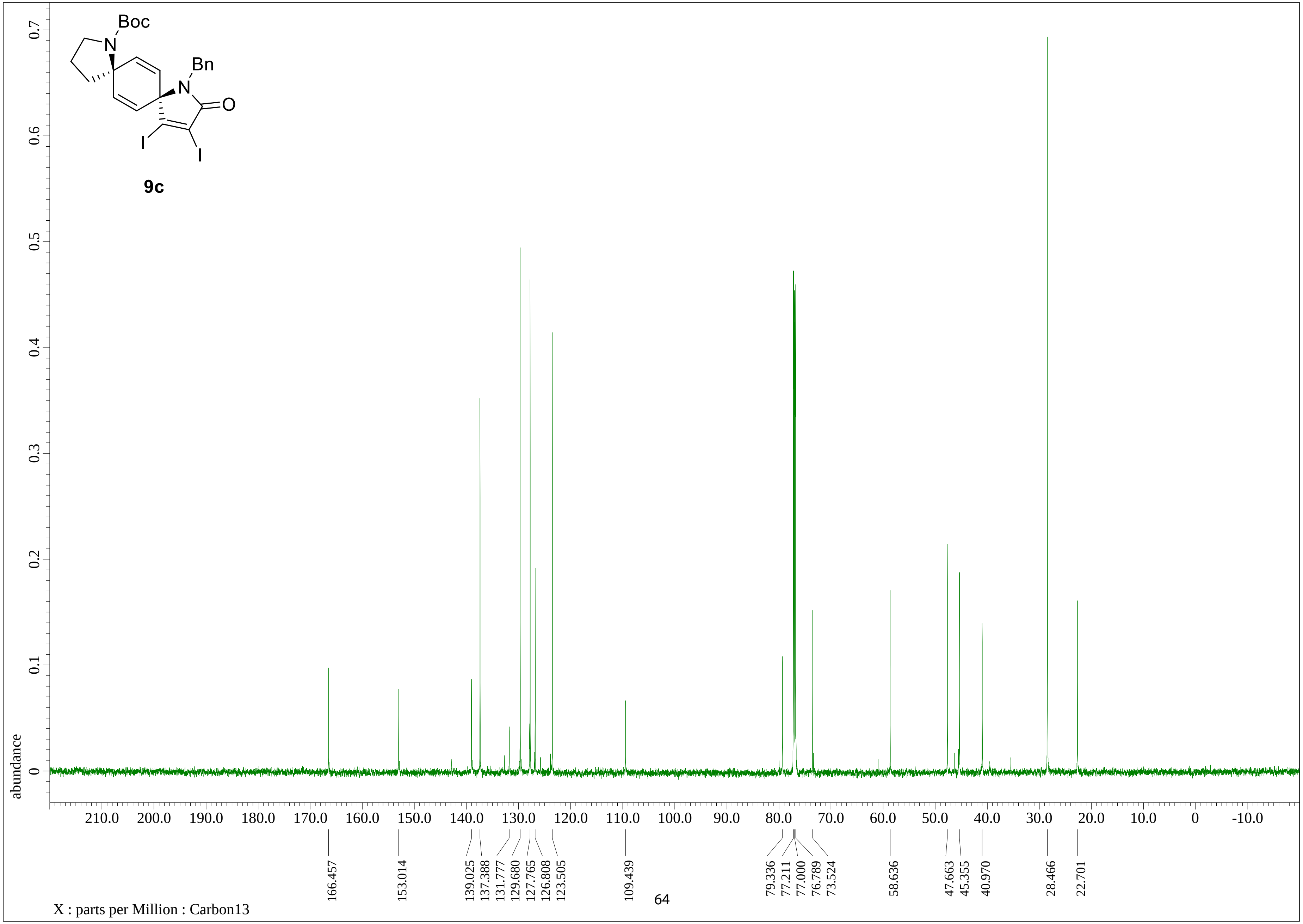
Boc
N
Bn
N
O
I
I
9c
abundance
0.7
0.6
0.5
0.4
0.3
0.2
0.1
0
210.0 200.0 190.0 180.0 170.0 160.0 150.0 140.0 130.0 120.0 110.0 100.0 90.0 80.0 70.0 60.0 50.0 40.0 30.0 20.0 10.0 0 -10.0
166.457
153.014
139.025
137.388
131.777
129.680
127.765
126.808
123.505
109.439
79.336
77.211
77.000
76.789
73.524
58.636
47.663
45.355
40.970
28.466
22.701
X : parts per Million : Carbon13

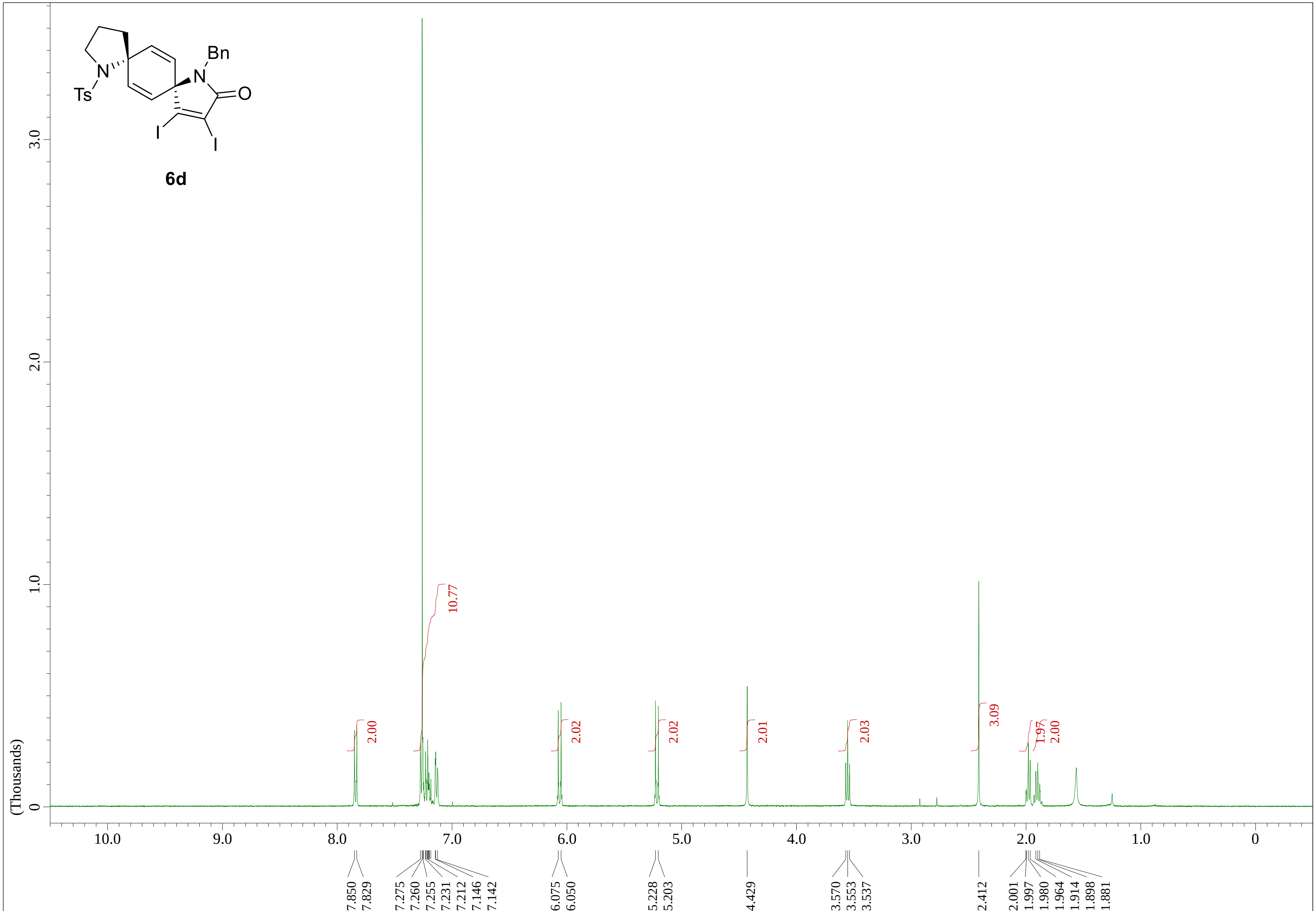

Bn
N
O
Ts
N
I
I
6d
(Thousands)
0
1.0
2.0
3.0
10.77
2.00
2.02
2.02
2.01
2.03
3.09
1.97
2.00
10.0
9.0
8.0
7.0
6.0
5.0
4.0
3.0
2.0
1.0
0
7.850
7.829
7.275
7.260
7.255
7.231
7.212
7.146
7.142
6.075
6.050
5.228
5.203
4.429
3.570
3.553
3.537
2.412
2.001
1.997
1.980
1.964
1.914
1.898
1.881
X : parts per Million : 1H

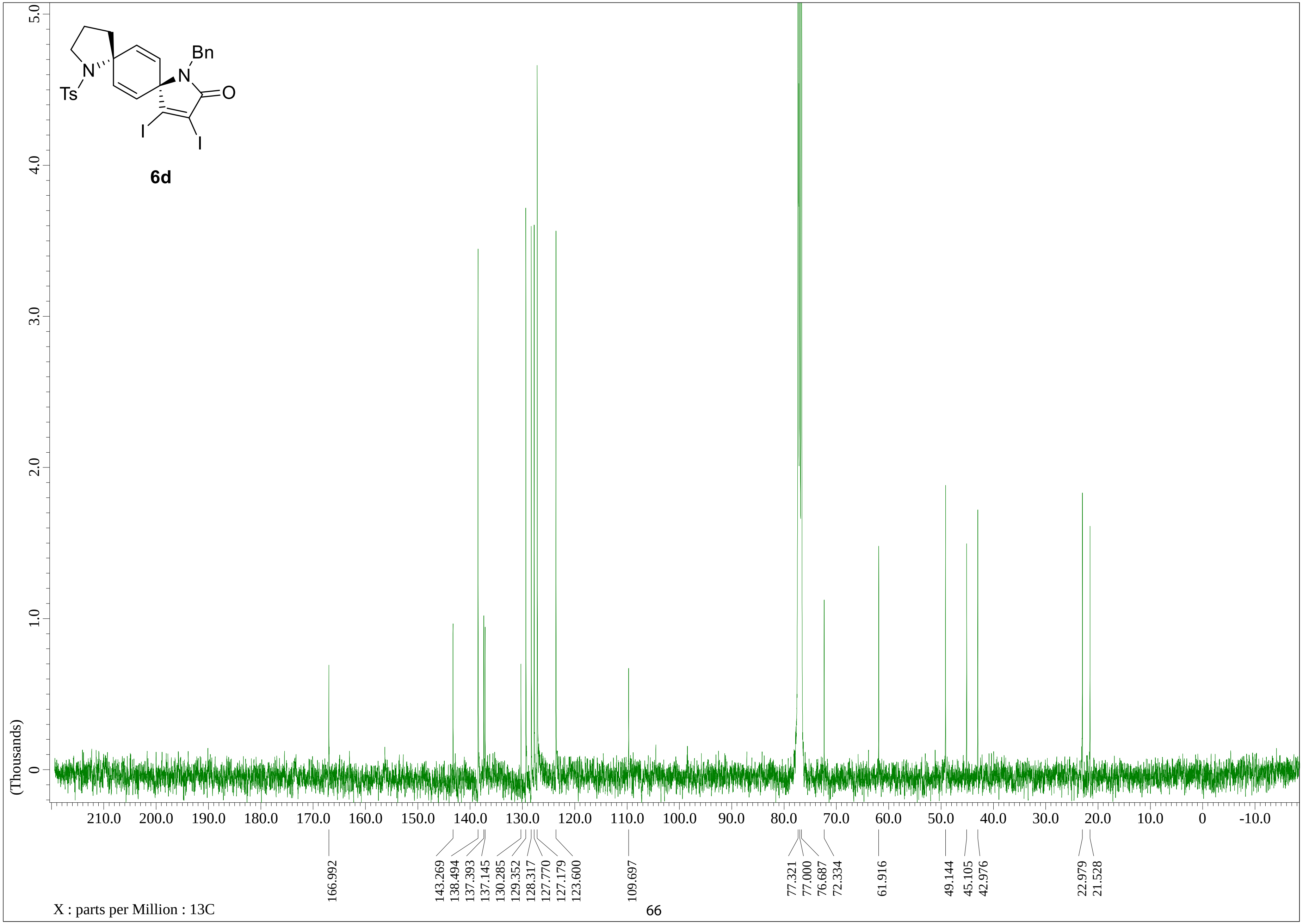
Bn
N
O
N
Ts
I
I
6d
(Thousands)
X : parts per Million : 13C
166.992
143.269
138.494
137.393
137.145
130.285
129.352
128.317
127.770
127.179
123.600
109.697
77.321
77.000
76.687
72.334
61.916
49.144
45.105
42.976
22.979
21.528

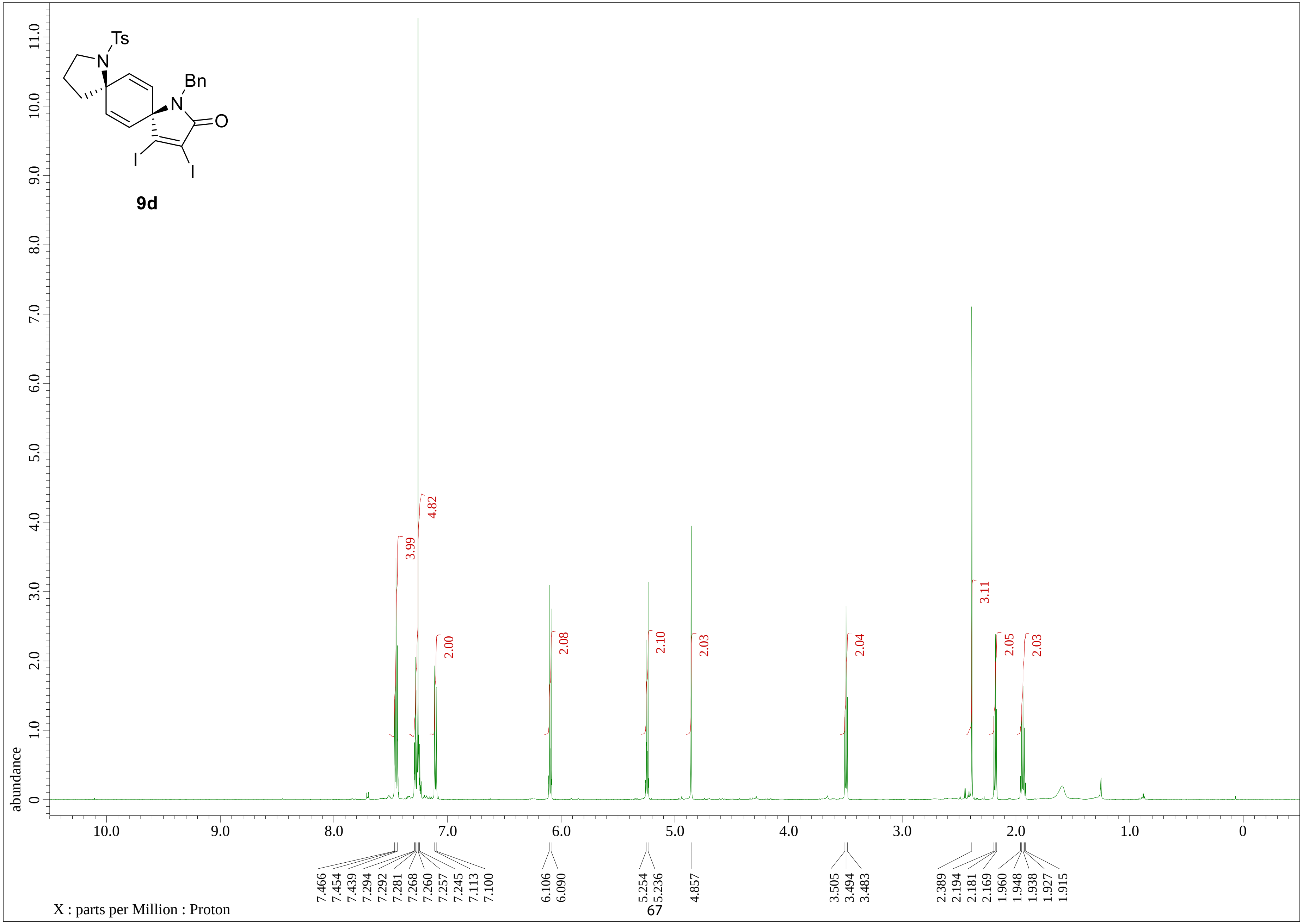

Ts
N
Bn
N
O
I
I
9d
abundance
X : parts per Million : Proton
7.466 7.454 7.439 7.294 7.292 7.281 7.268 7.260 7.257 7.245 7.113 7.100
6.106 6.090
5.254 5.236
4.857
3.505 3.494 3.483
2.389 2.194 2.181 2.169 1.960 1.948 1.938 1.927 1.915
3.99 4.82 2.00 2.08 2.10 2.03 2.04 3.11 2.05 2.03

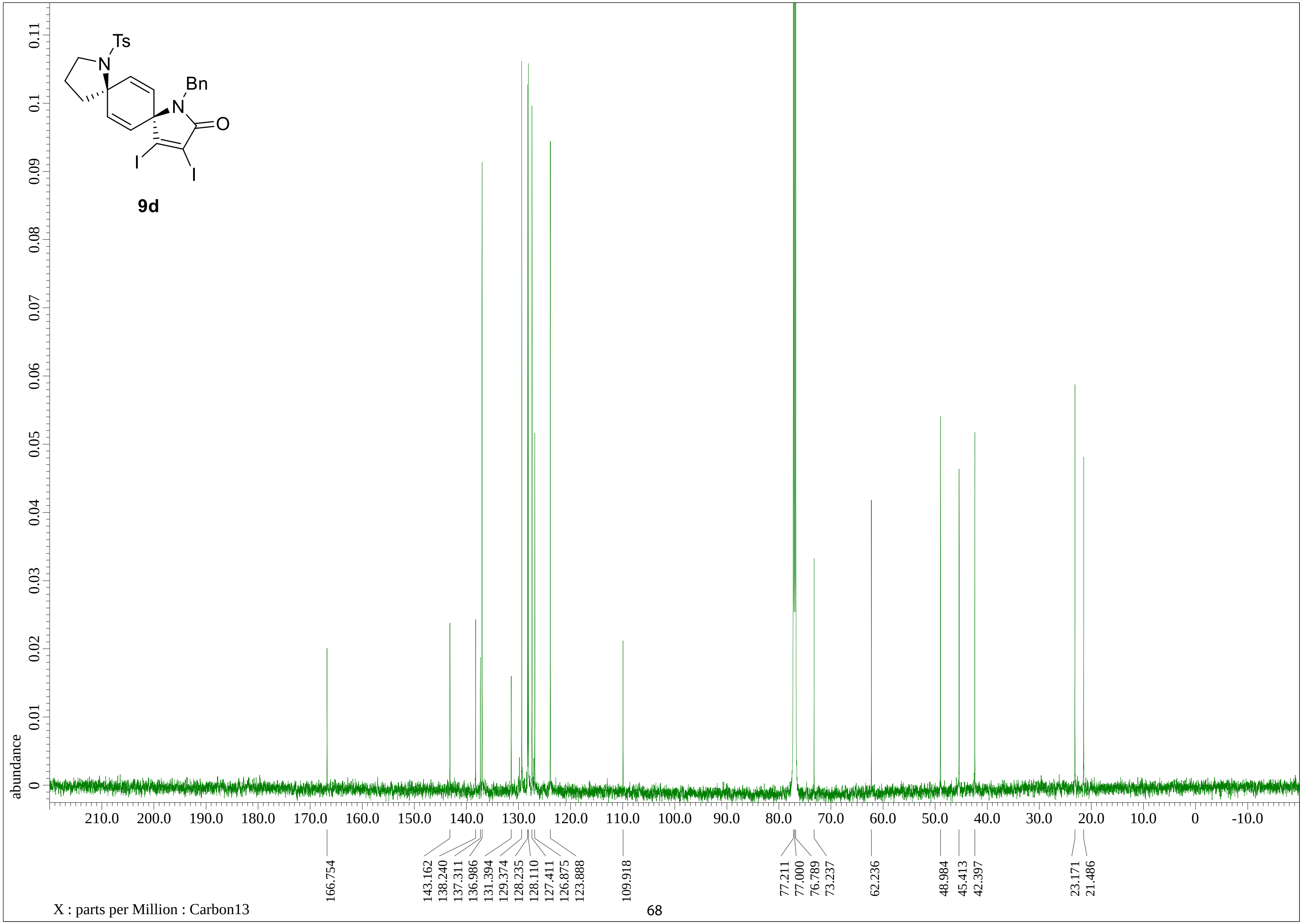
Ts
N
Bn
N
O
I
I
9d
abundance
X : parts per Million : Carbon13
166.754
143.162
138.240
137.311
136.986
131.394
129.374
128.235
128.110
127.411
126.875
123.888
109.918
77.211
77.000
76.789
73.237
62.236
48.984
45.413
42.397
23.171
21.486

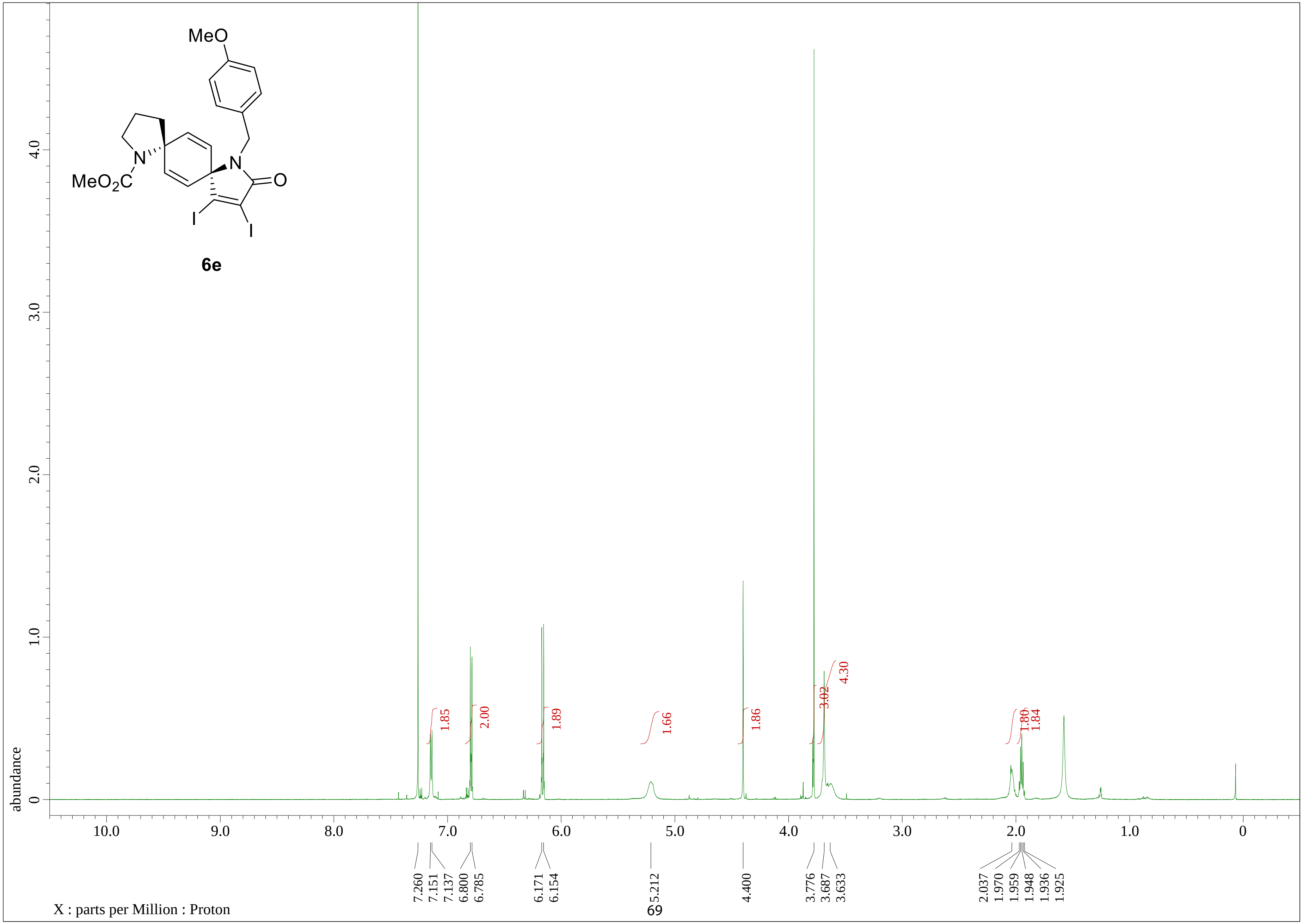
MeO
N
MeO2C
N
O
I
I
6e
abundance
X : parts per Million : Proton
7.260
7.151
7.137
6.800
6.785
6.171
6.154
5.212
4.400
3.776
3.687
3.633
2.037
1.970
1.959
1.948
1.936
1.925
1.85
2.00
1.89
1.66
1.86
3.02
4.30
1.80
1.84

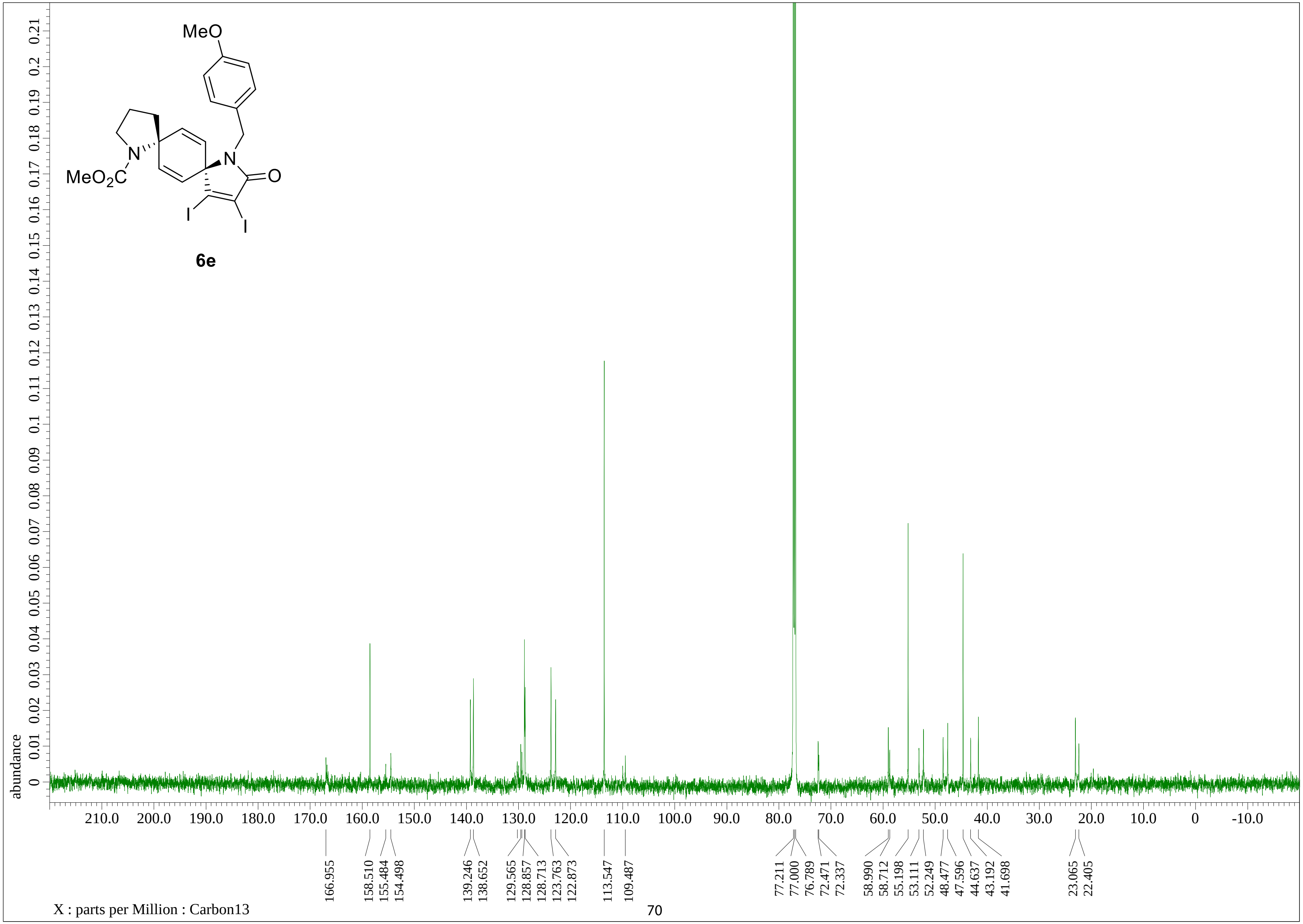
MeO
MeO2C
N
N
O
I
I
6e
abundance
X : parts per Million : Carbon13
166.955
158.510
155.484
154.498
139.246
138.652
129.565
128.857
128.713
123.763
122.873
113.547
109.487
77.211
77.000
76.789
72.471
72.337
58.990
58.712
55.198
53.111
52.249
48.477
47.596
44.637
43.192
41.698
23.065
22.405

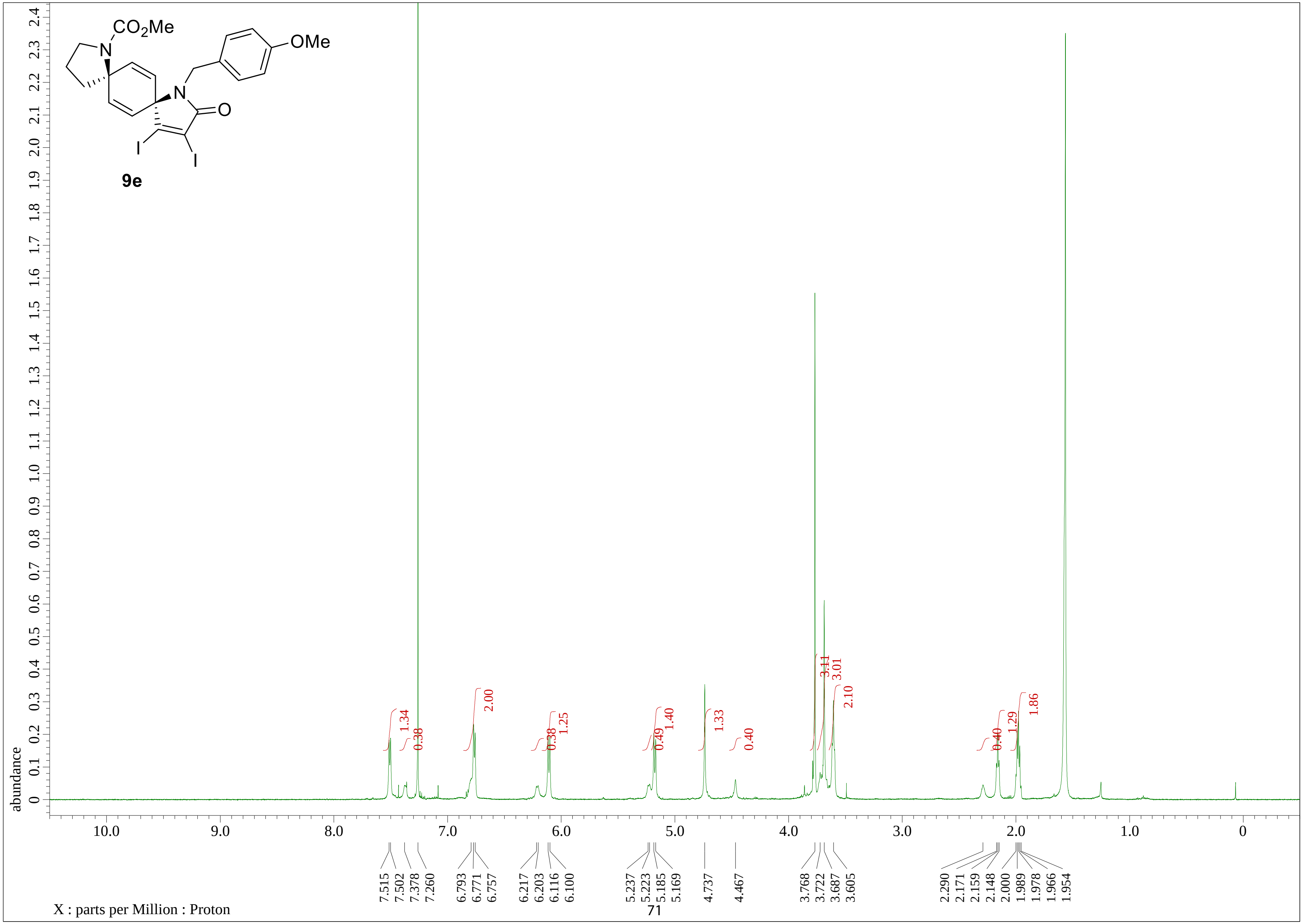

CO2Me
OMe
N
N
O
I
I
9e
abundance
X : parts per Million : Proton
7.515
7.502
7.378
7.260
6.793
6.771
6.757
6.217
6.203
6.116
6.100
5.237
5.223
5.185
5.169
4.737
4.467
3.768
3.722
3.687
3.605
2.290
2.171
2.159
2.148
2.000
1.989
1.978
1.966
1.954

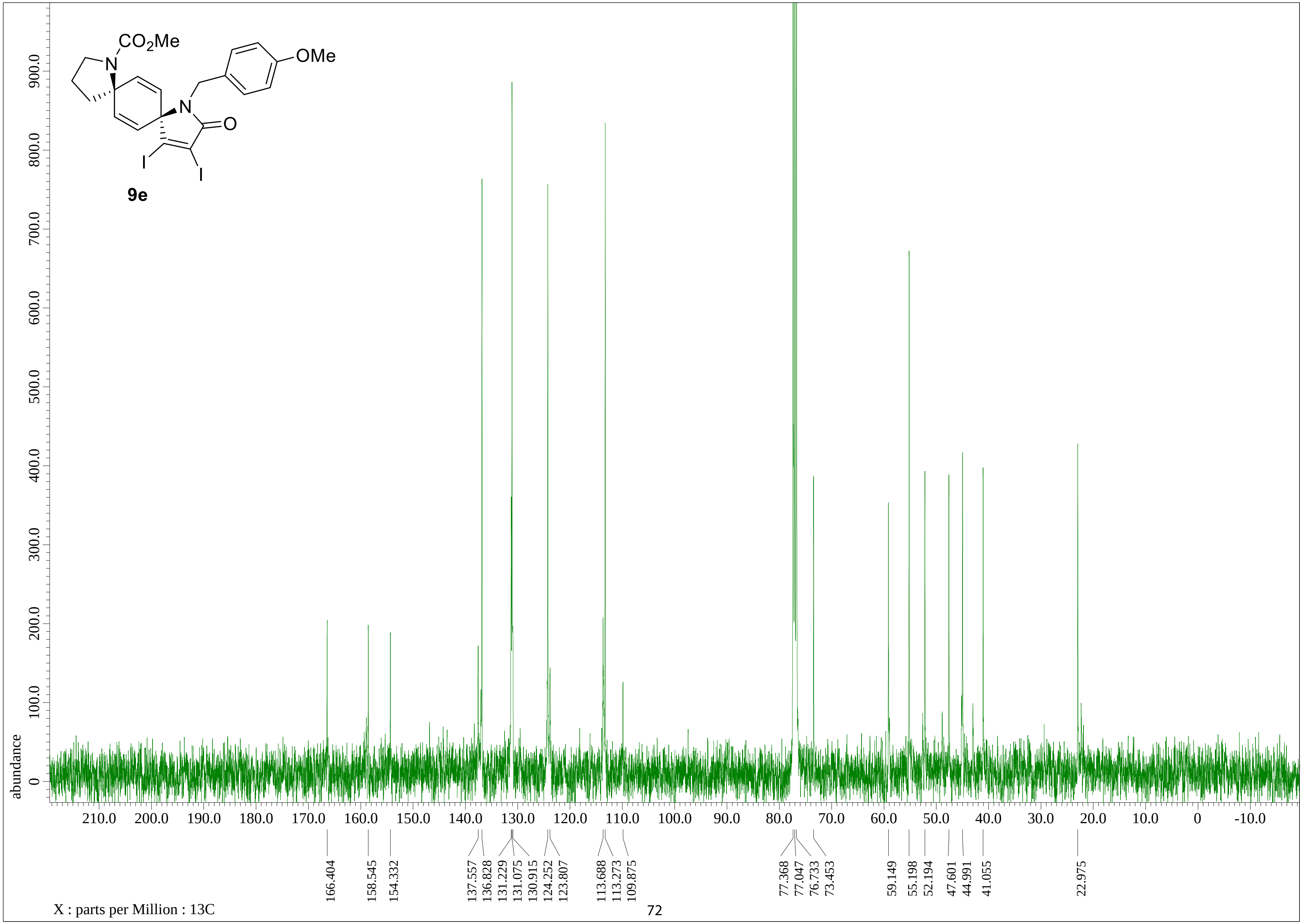
CO2Me
N
OMe
N
O
I
I
9e
abundance
X : parts per Million : 13C
166.404
158.545
154.332
137.557
136.828
131.229
131.075
130.915
124.252
123.807
113.688
113.273
109.875
77.368
77.047
76.733
73.453
59.149
55.198
52.194
47.601
44.991
41.055
22.975

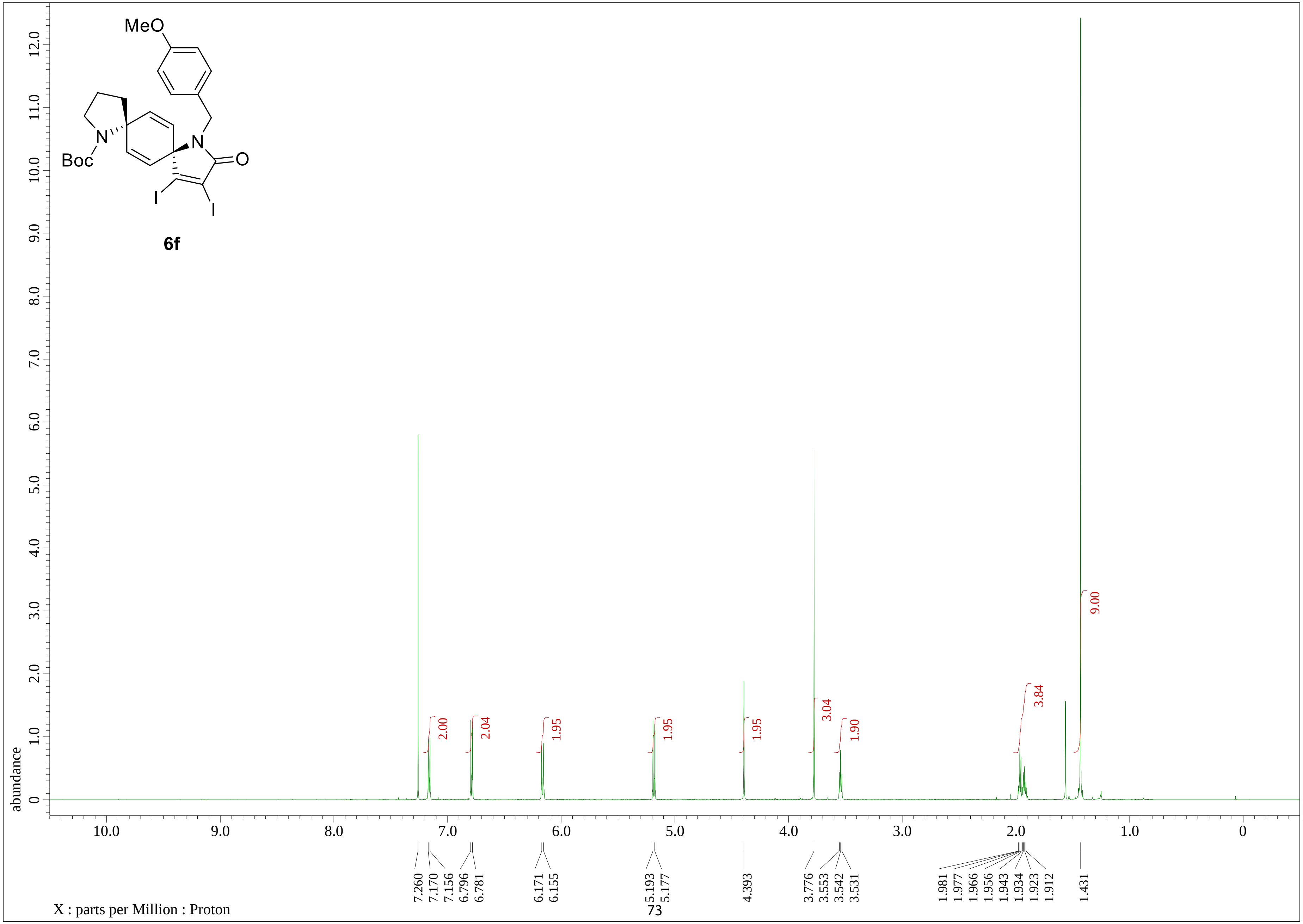
MeO
N
Boc
N
O
I
I
6f
abundance
X : parts per Million : Proton
9.00
3.84
3.04
1.90
1.95
1.95
1.95
2.04
2.00
7.260
7.170
7.156
6.796
6.781
6.171
6.155
5.193
5.177
4.393
3.776
3.553
3.542
3.531
1.981
1.977
1.966
1.956
1.943
1.934
1.923
1.912
1.431

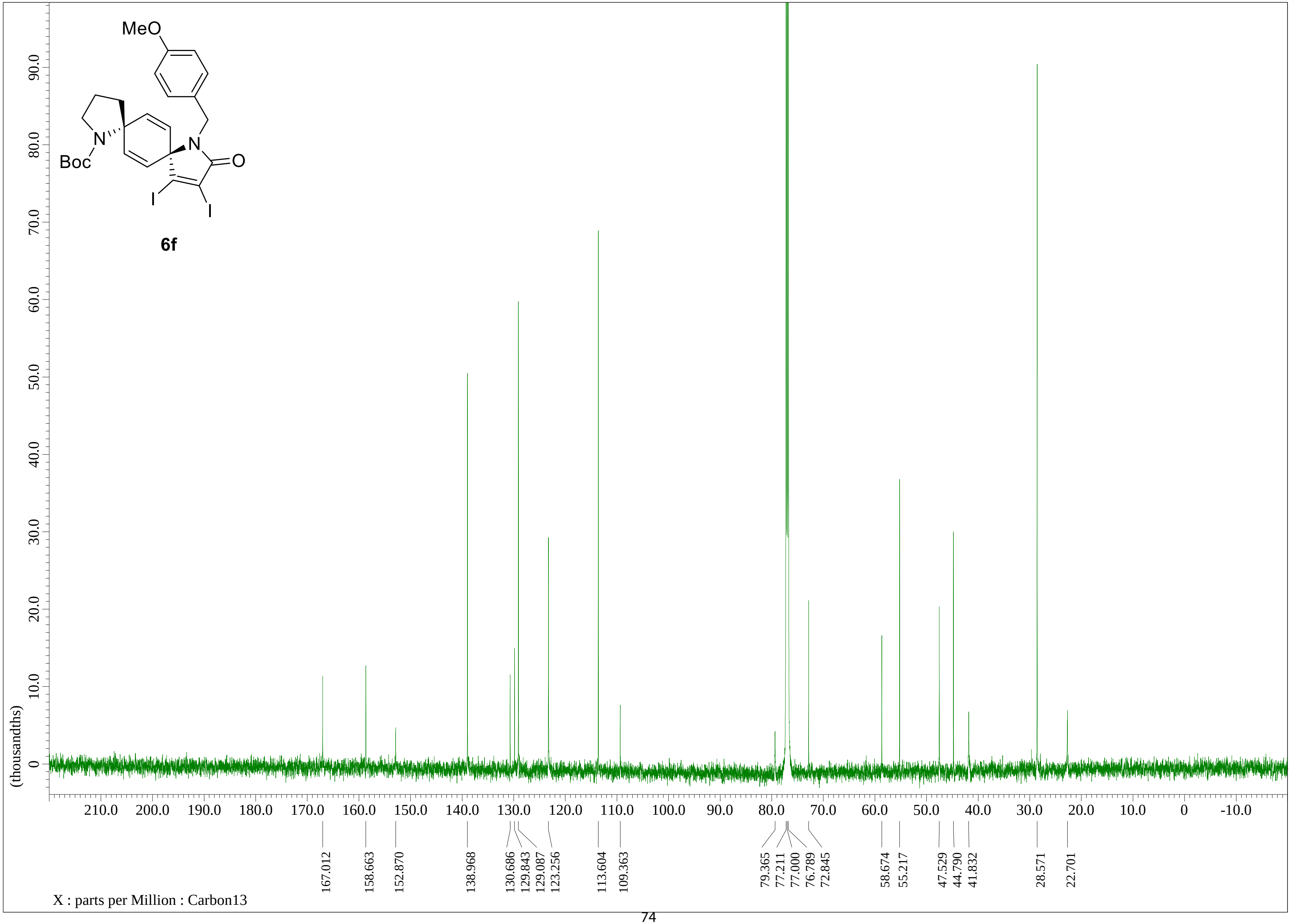
MeO
N
Boc
N
O
I
I
6f
(thousandths)
90.0
80.0
70.0
60.0
50.0
40.0
30.0
20.0
10.0
0
210.0 200.0 190.0 180.0 170.0 160.0 150.0 140.0 130.0 120.0 110.0 100.0 90.0 80.0 70.0 60.0 50.0 40.0 30.0 20.0 10.0 0 -10.0
167.012
158.663
152.870
138.968
130.686
129.843
129.087
123.256
113.604
109.363
79.365
77.211
77.000
76.789
72.845
58.674
55.217
47.529
44.790
41.832
28.571
22.701
X : parts per Million : Carbon13

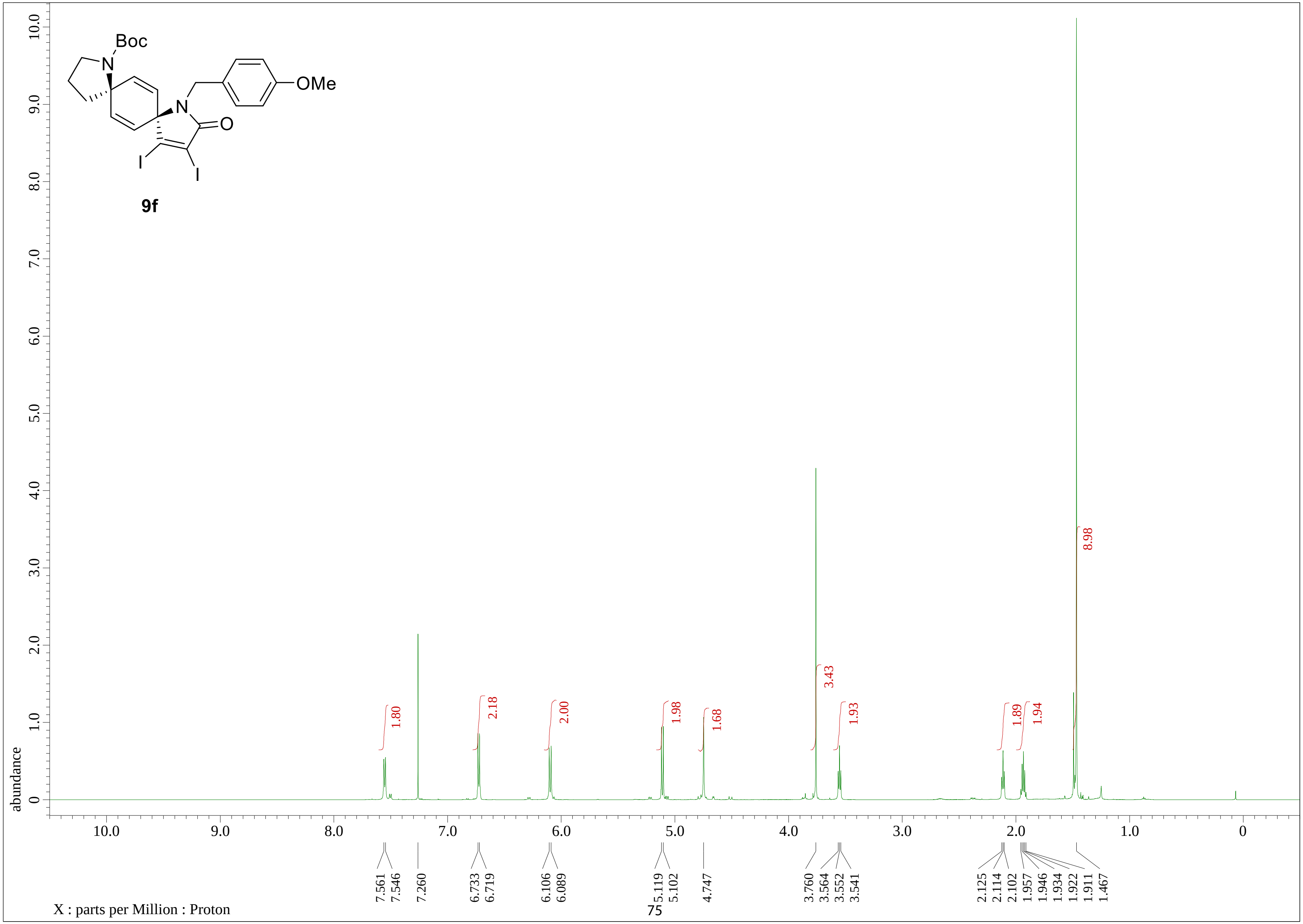

Boc
N
N
OMe
O
I
I
9f
abundance
X : parts per Million : Proton
1.80
2.18
2.00
1.98
1.68
3.43
1.93
1.89
1.94
8.98
7.561
7.546
7.260
6.733
6.719
6.106
6.089
5.119
5.102
4.747
3.760
3.564
3.552
3.541
2.125
2.114
2.102
1.957
1.946
1.934
1.922
1.911
1.467

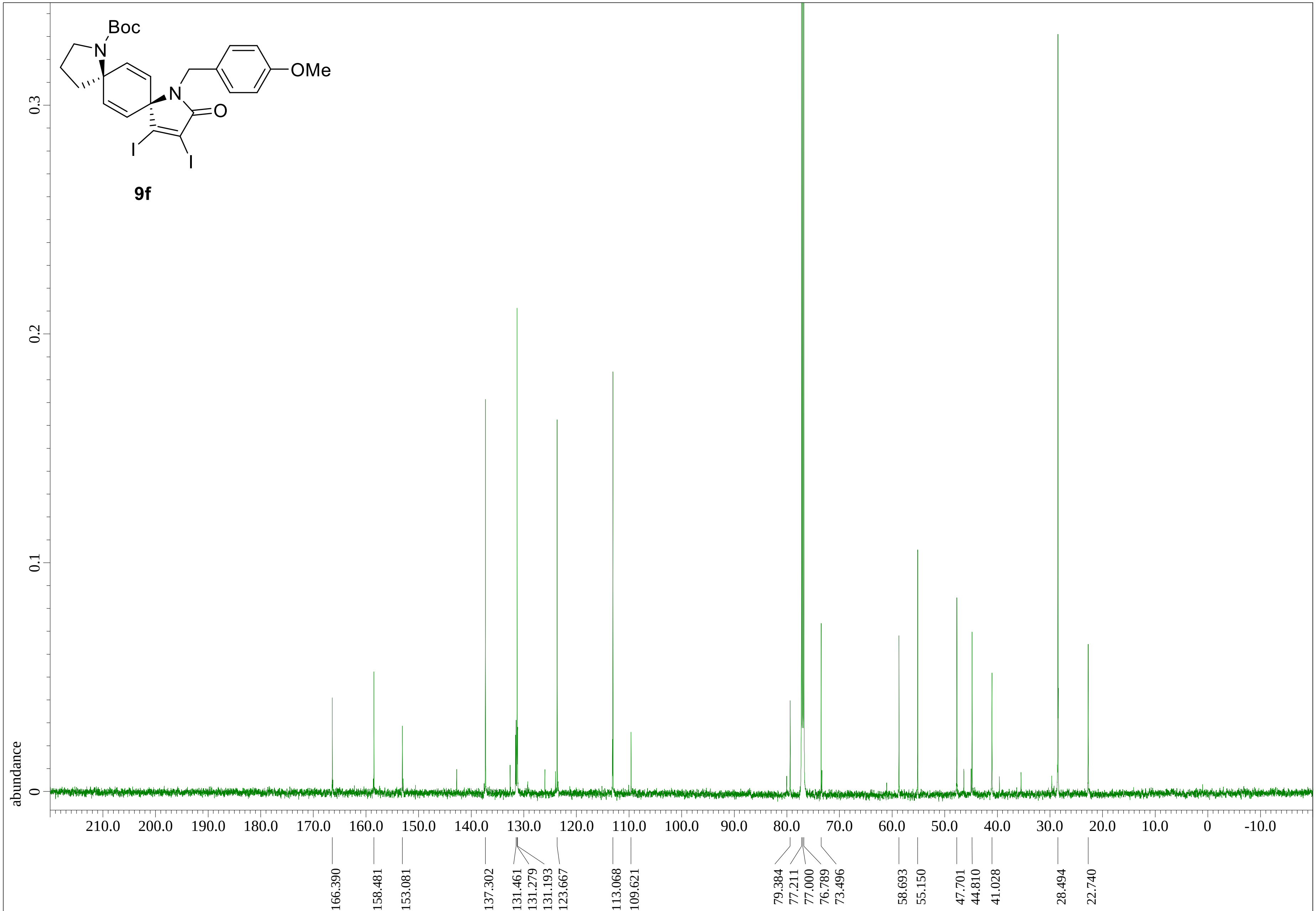
Boc
N
N
O
OMe
I
I
9f
abundance
0
0.1
0.2
0.3
210.0 200.0 190.0 180.0 170.0 160.0 150.0 140.0 130.0 120.0 110.0 100.0 90.0 80.0 70.0 60.0 50.0 40.0 30.0 20.0 10.0 0 -10.0
166.390
158.481
153.081
137.302
131.461
131.279
131.193
123.667
113.068
109.621
79.384
77.211
77.000
76.789
73.496
58.693
55.150
47.701
44.810
41.028
28.494
22.740
X : parts per Million : Carbon13

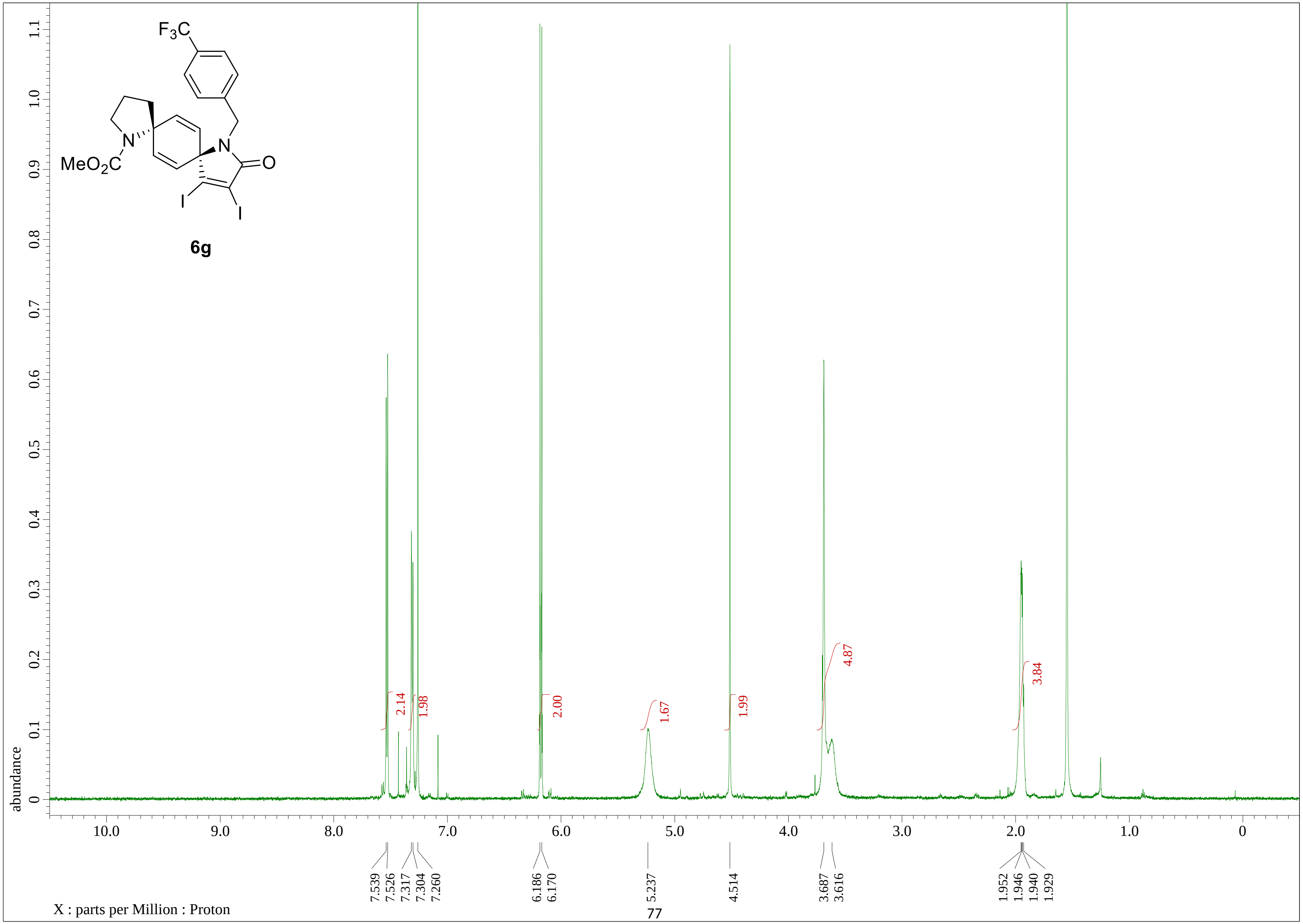
F3C
N
MeO2C
N
O
I
I
6g
abundance
X : parts per Million : Proton
7.539
7.526
7.317
7.304
7.260
6.186
6.170
5.237
4.514
3.687
3.616
1.952
1.946
1.940
1.929
2.14
1.98
2.00
1.67
1.99
4.87
3.84

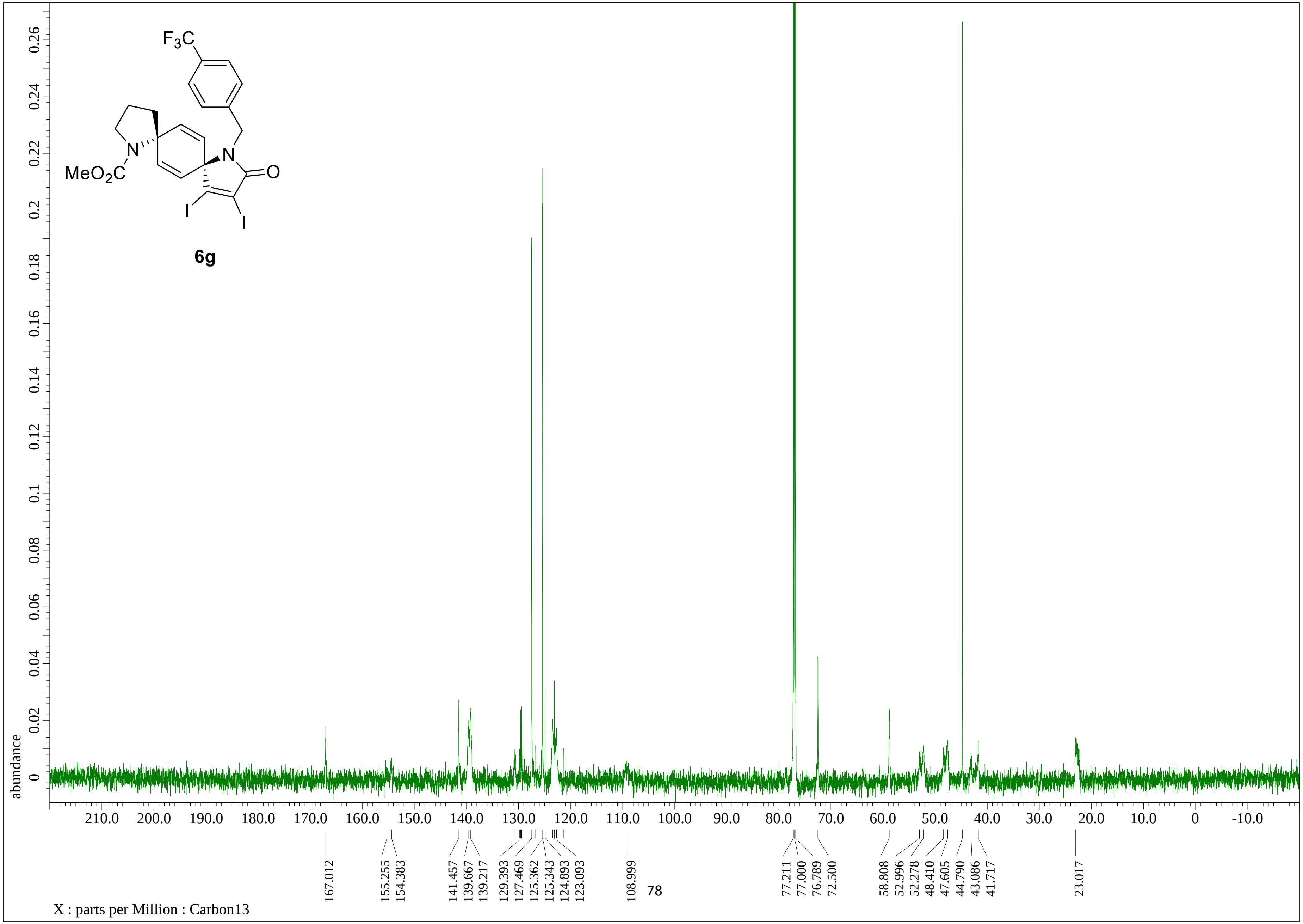
F3C
N
N
O
MeO2C
I
I
6g
abundance
0.26
0.24
0.22
0.2
0.18
0.16
0.14
0.12
0.1
0.08
0.06
0.04
0.02
0
210.0 200.0 190.0 180.0 170.0 160.0 150.0 140.0 130.0 120.0 110.0 100.0 90.0 80.0 70.0 60.0 50.0 40.0 30.0 20.0 10.0 0 -10.0
167.012
155.255
154.383
141.457
139.667
139.217
129.393
127.469
125.362
125.343
124.893
123.093
108.999
77.211
77.000
76.789
72.500
58.808
52.996
52.278
48.410
47.605
44.790
43.086
41.717
23.017
X : parts per Million : Carbon13

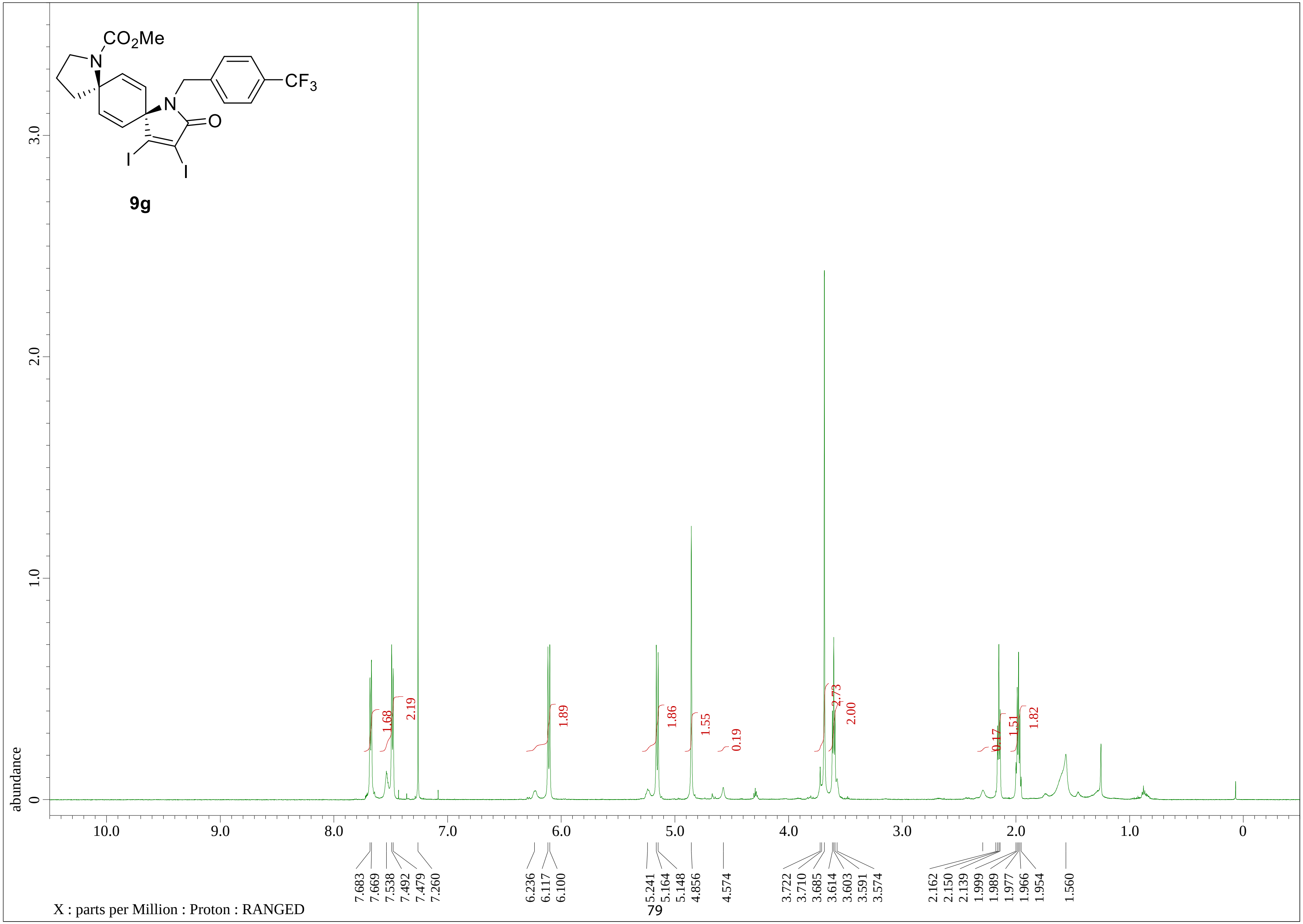
CO2Me
N
N
CF3
O
I
I
9g
abundance
X : parts per Million : Proton : RANGED
7.683 7.669 7.538 7.492 7.479 7.260 6.236 6.117 6.100 5.241 5.164 5.148 4.856 4.574 3.722 3.710 3.685 3.614 3.603 3.591 3.574 2.162 2.150 2.139 1.999 1.989 1.977 1.966 1.954 1.560
1.68 2.19 1.89 1.86 1.55 0.19 2.73 2.00 0.17 1.51 1.82

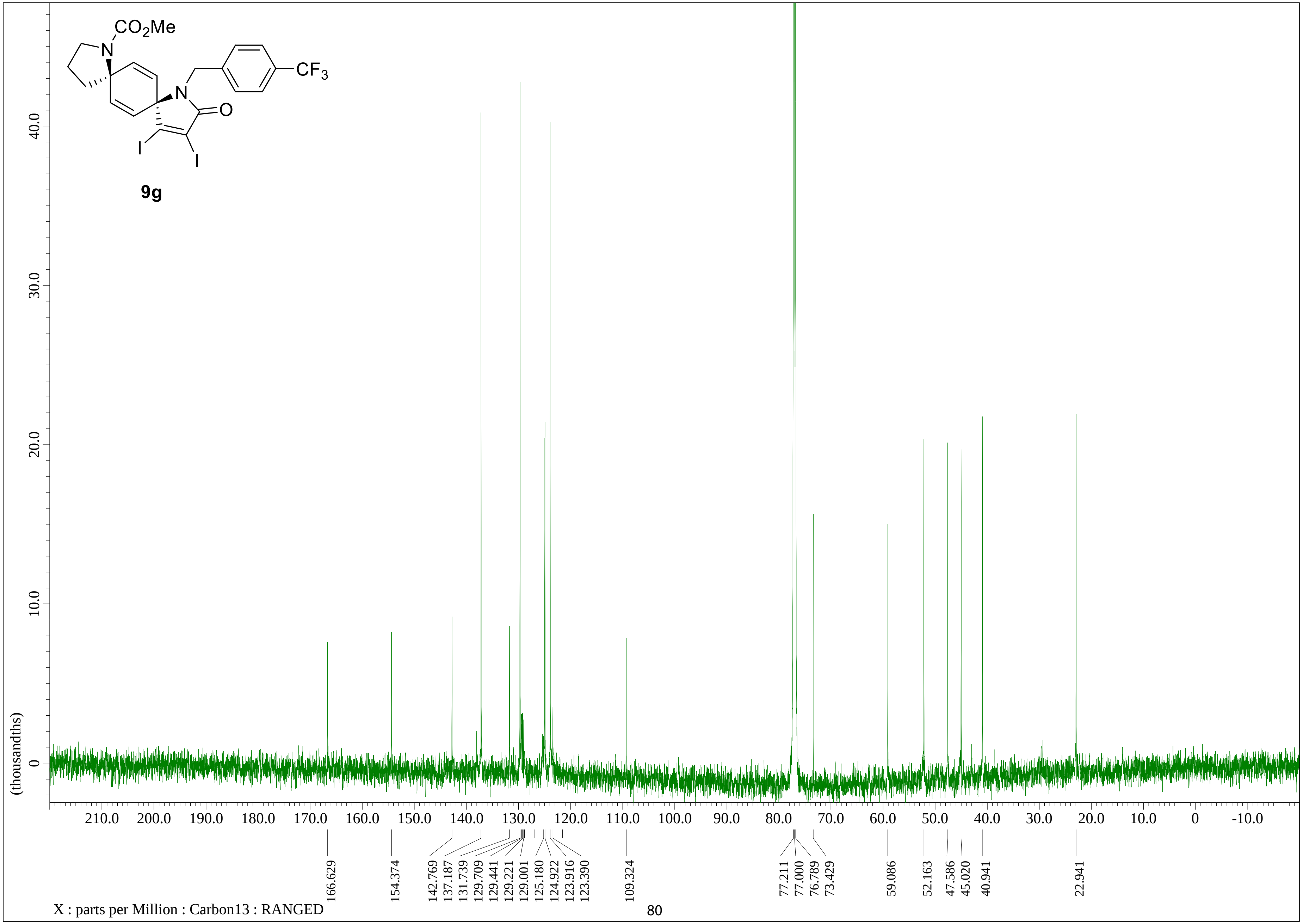
CO2Me
N
N
CF3
O
I
I
9g
(thousandths)
40.0
30.0
20.0
10.0
0
210.0 200.0 190.0 180.0 170.0 160.0 150.0 140.0 130.0 120.0 110.0 100.0 90.0 80.0 70.0 60.0 50.0 40.0 30.0 20.0 10.0 0 -10.0
166.629
154.374
142.769
137.187
131.739
129.709
129.441
129.221
129.001
125.180
124.922
123.916
123.390
109.324
77.211
77.000
76.789
73.429
59.086
52.163
47.586
45.020
40.941
22.941
X : parts per Million : Carbon13 : RANGED

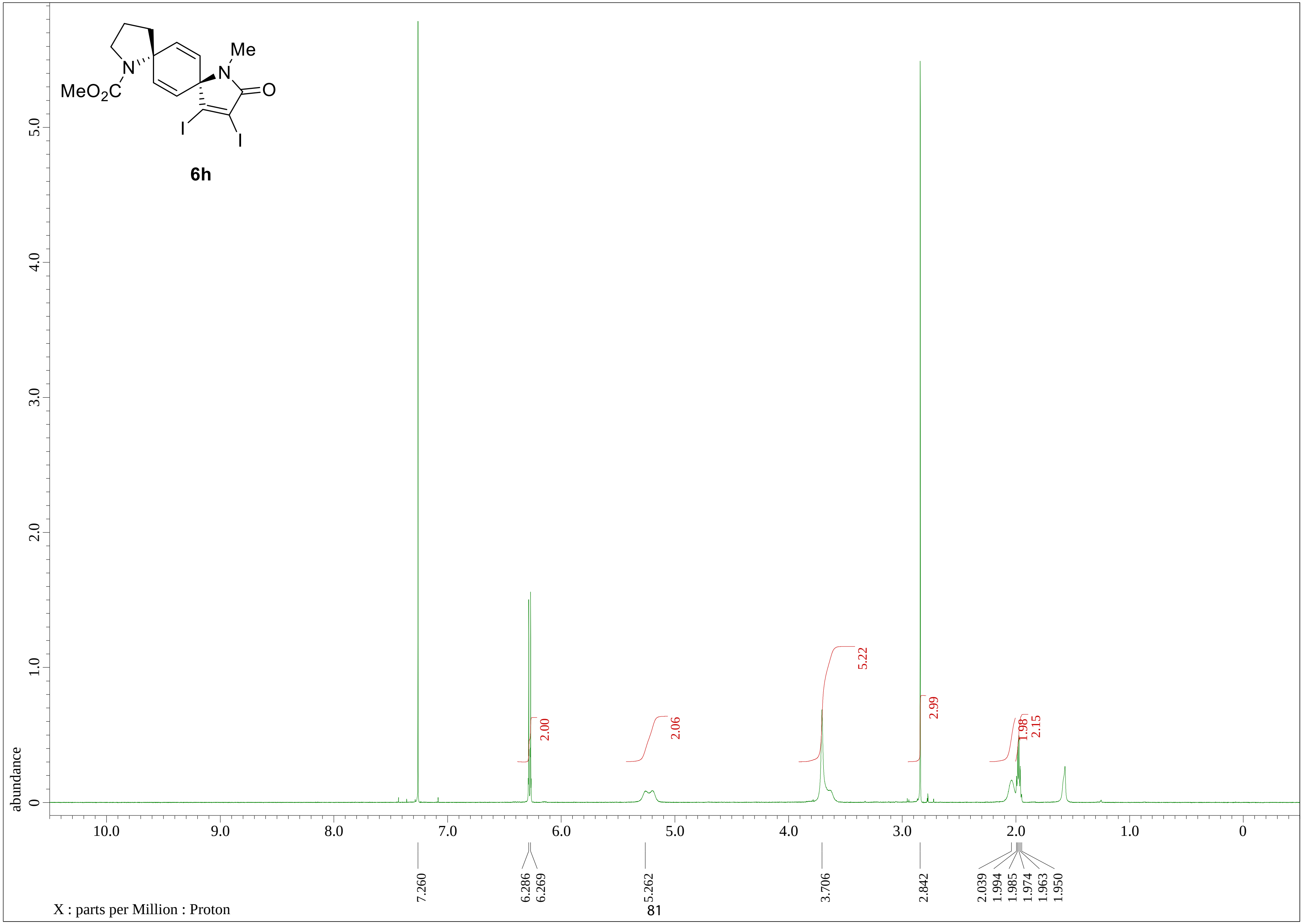
Me
N
MeO2C
N
O
I
I
6h
abundance
5.0
4.0
3.0
2.0
1.0
0
2.00
2.06
5.22
2.99
1.98
2.15
10.0
9.0
8.0
7.0
6.0
5.0
4.0
3.0
2.0
1.0
0
7.260
6.286
6.269
5.262
3.706
2.842
2.039
1.994
1.985
1.974
1.963
1.950
X : parts per Million : Proton

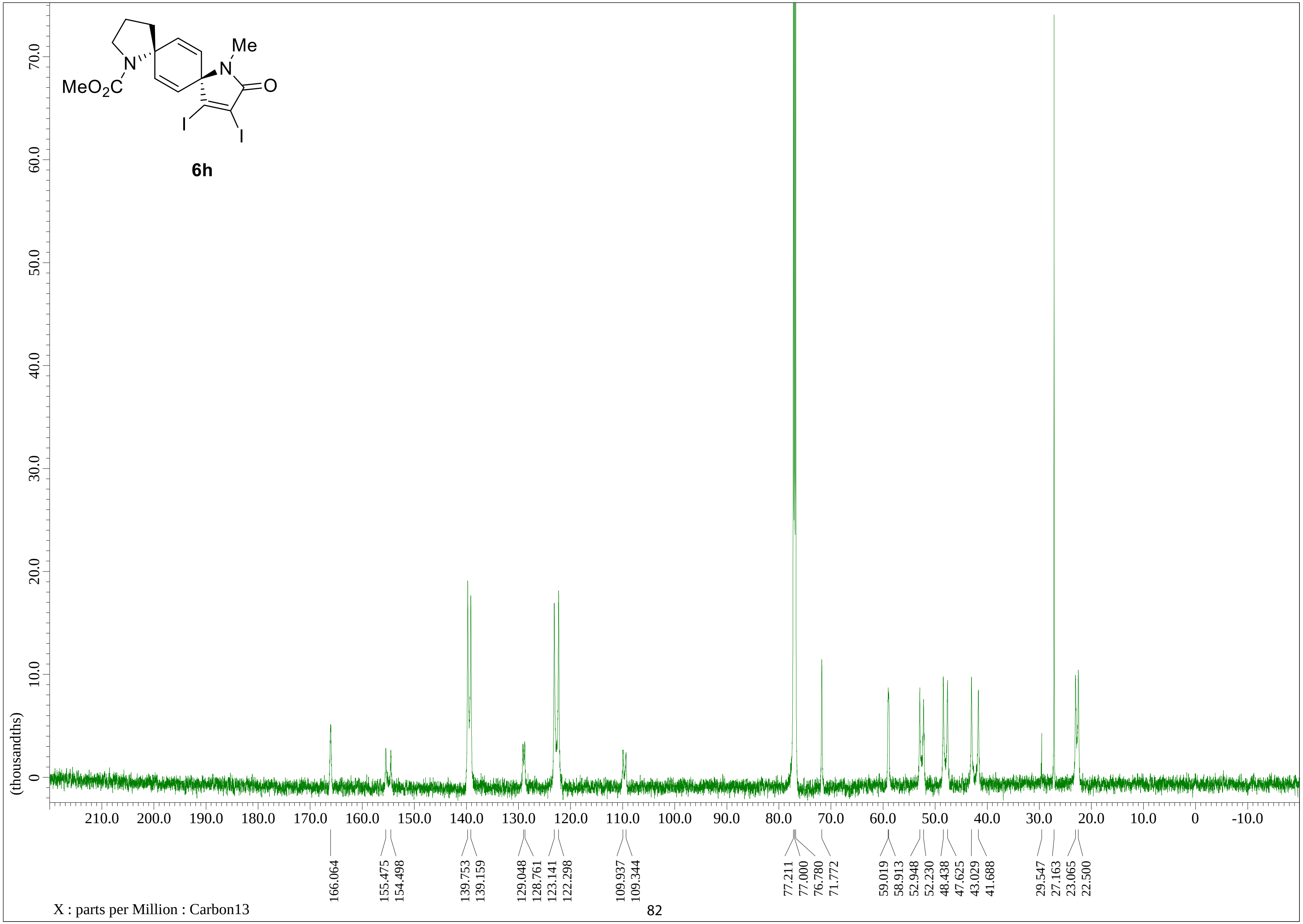
MeO2C
N
Me
N
O
I
I
6h
(thousandths)
X : parts per Million : Carbon13
166.064
155.475
154.498
139.753
139.159
129.048
128.761
123.141
122.298
109.937
109.344
77.211
77.000
76.780
71.772
59.019
58.913
52.948
52.230
48.438
47.625
43.029
41.688
29.547
27.163
23.065
22.500

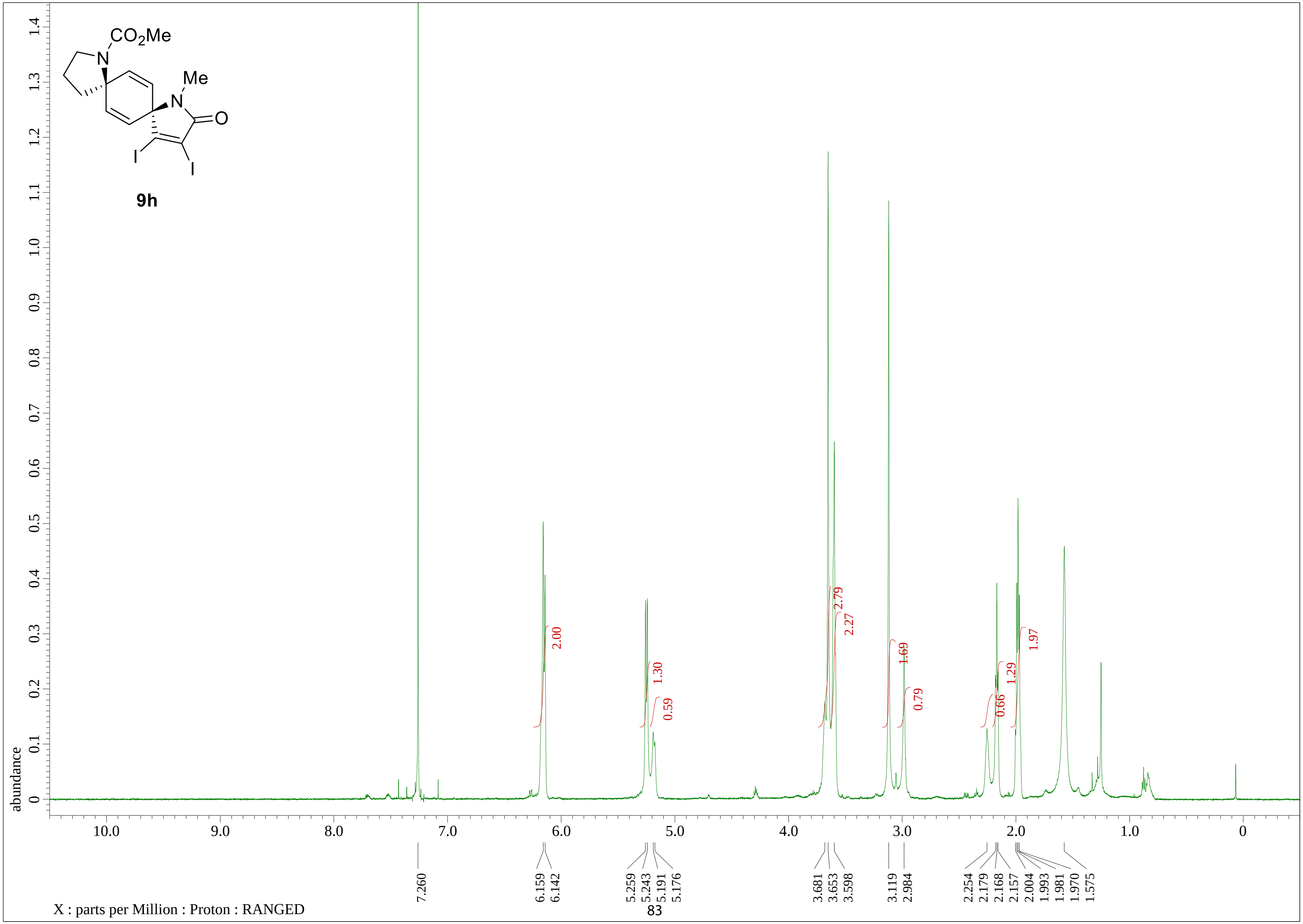
CO2Me
N
Me
N
O
I
I
9h
abundance
X : parts per Million : Proton : RANGED
7.260
6.159
6.142
5.259
5.243
5.191
5.176
3.681
3.653
3.598
3.119
2.984
2.254
2.179
2.168
2.157
2.004
1.993
1.981
1.970
1.575
2.00
1.30
0.59
2.79
2.27
1.69
0.79
0.66
1.29
1.97

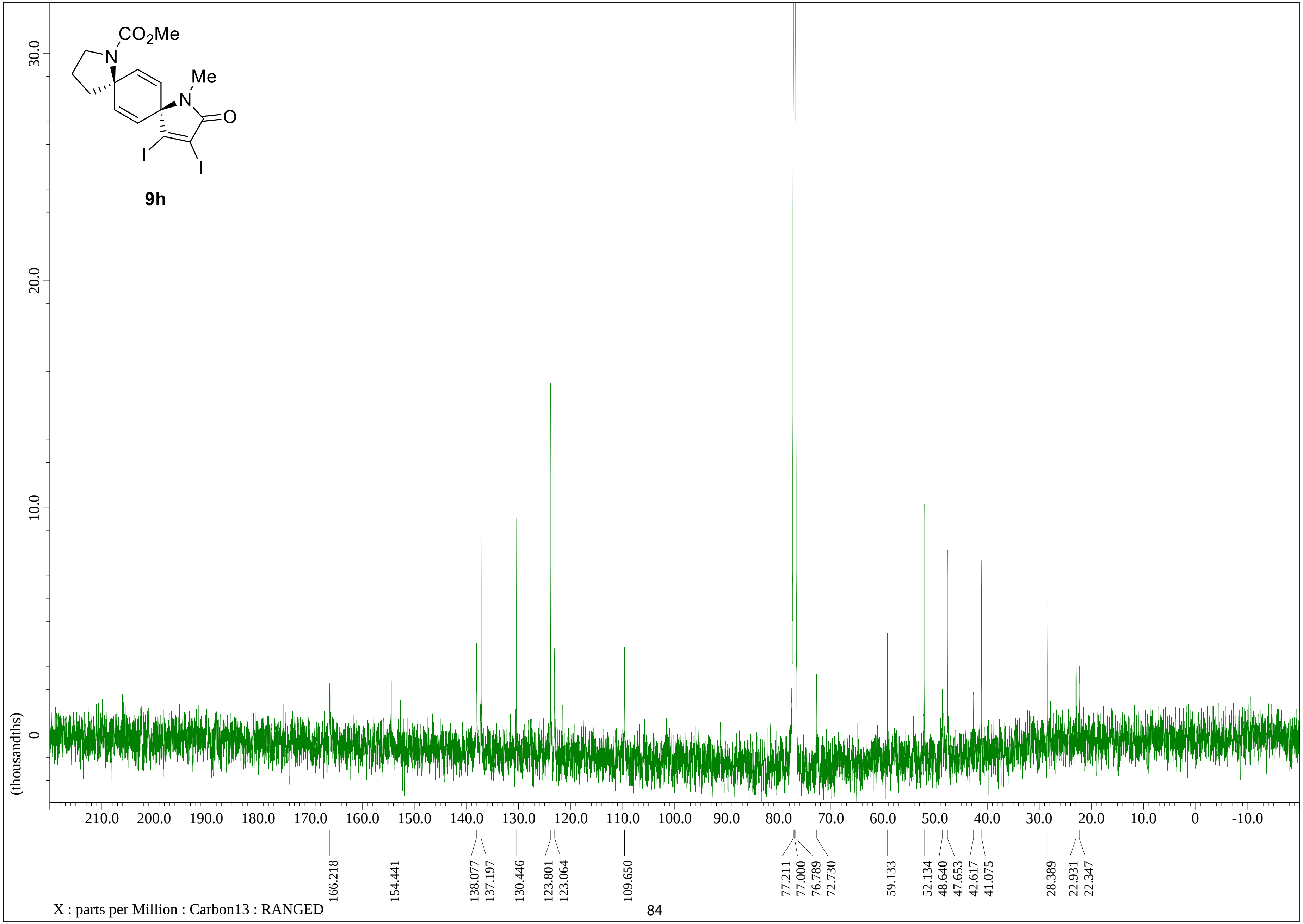

9h
30.0
20.0
10.0
0
(thousandths)
210.0 200.0 190.0 180.0 170.0 160.0 150.0 140.0 130.0 120.0 110.0 100.0 90.0 80.0 70.0 60.0 50.0 40.0 30.0 20.0 10.0 0 -10.0
166.218
154.441
138.077
137.197
130.446
123.801
123.064
109.650
77.211
77.000
76.789
72.730
59.133
52.134
48.640
47.653
42.617
41.075
28.389
22.931
22.347
X : parts per Million : Carbon13 : RANGED

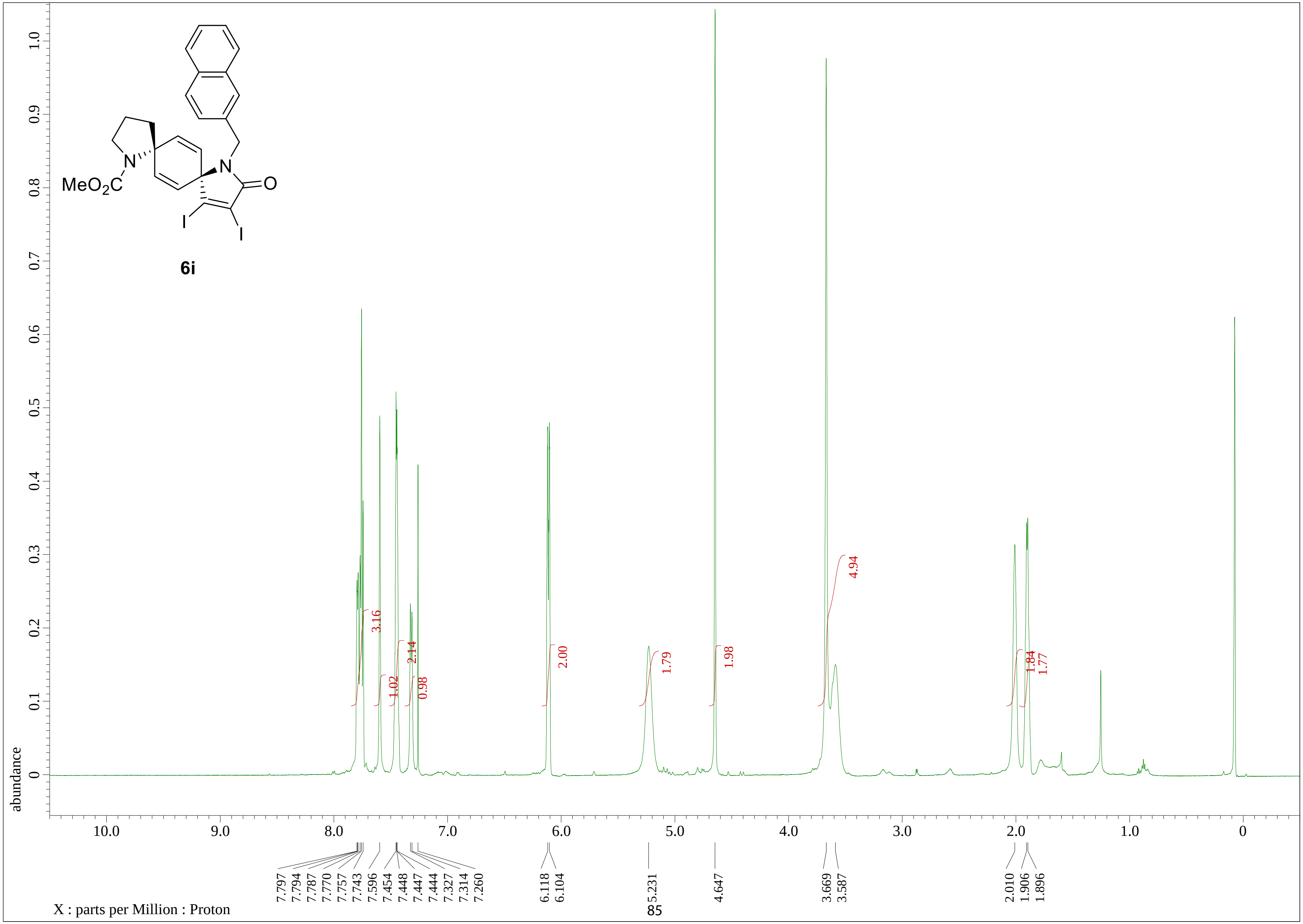
MeO2C
N
N
O
I
I
6i
abundance
X : parts per Million : Proton
7.797
7.794
7.787
7.770
7.757
7.743
7.596
7.454
7.448
7.447
7.444
7.327
7.314
7.260
6.118
6.104
5.231
4.647
3.669
3.587
2.010
1.906
1.896
3.16
1.02
2.14
0.98
2.00
1.79
1.98
4.94
1.84
1.77

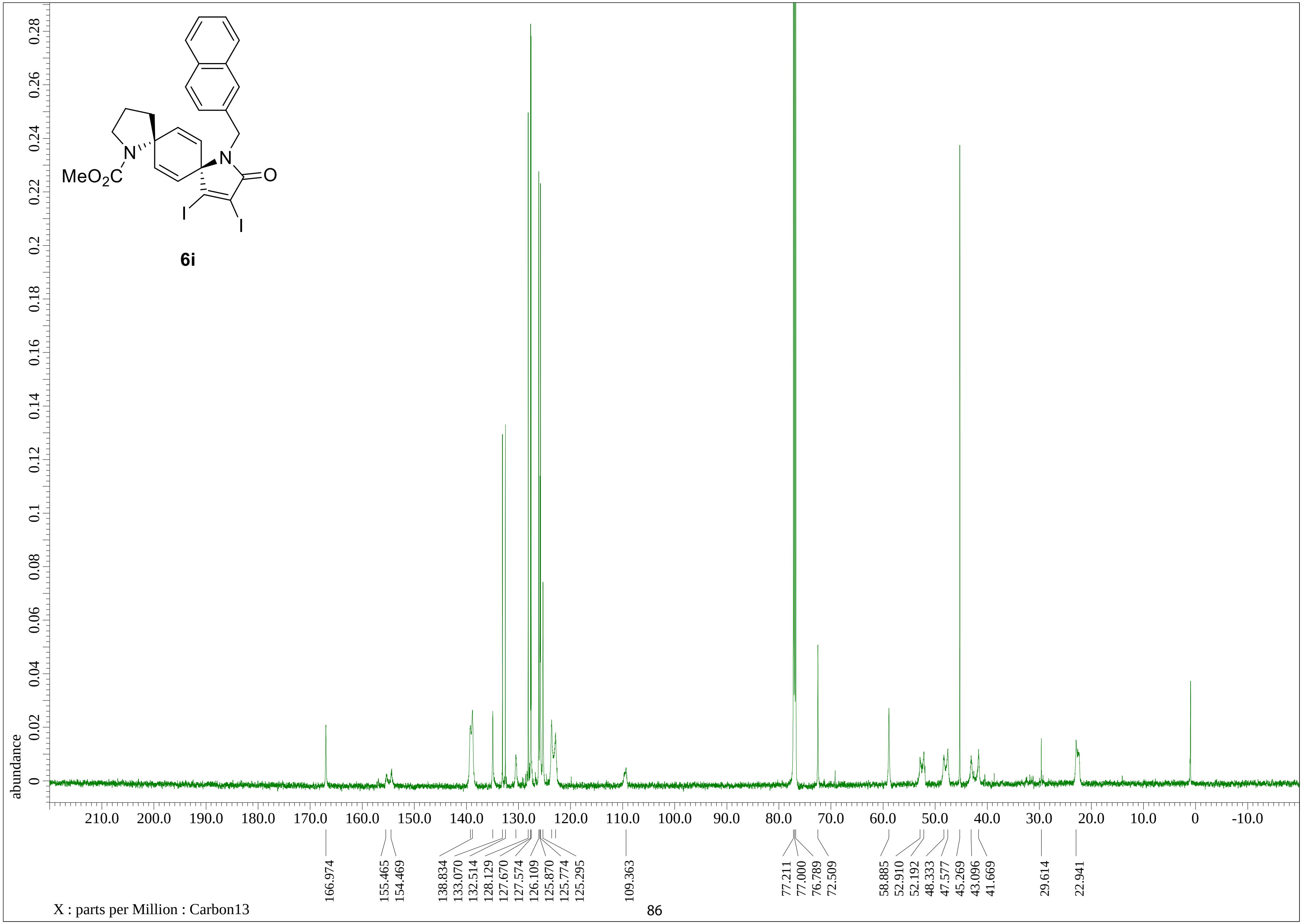
MeO2C
N
N
O
I
I
6i
abundance
X : parts per Million : Carbon13
166.974
155.465
154.469
138.834
133.070
132.514
128.129
127.670
127.574
126.109
125.870
125.774
125.295
109.363
77.211
77.000
76.789
72.509
58.885
52.910
52.192
48.333
47.577
45.269
43.096
41.669
29.614
22.941

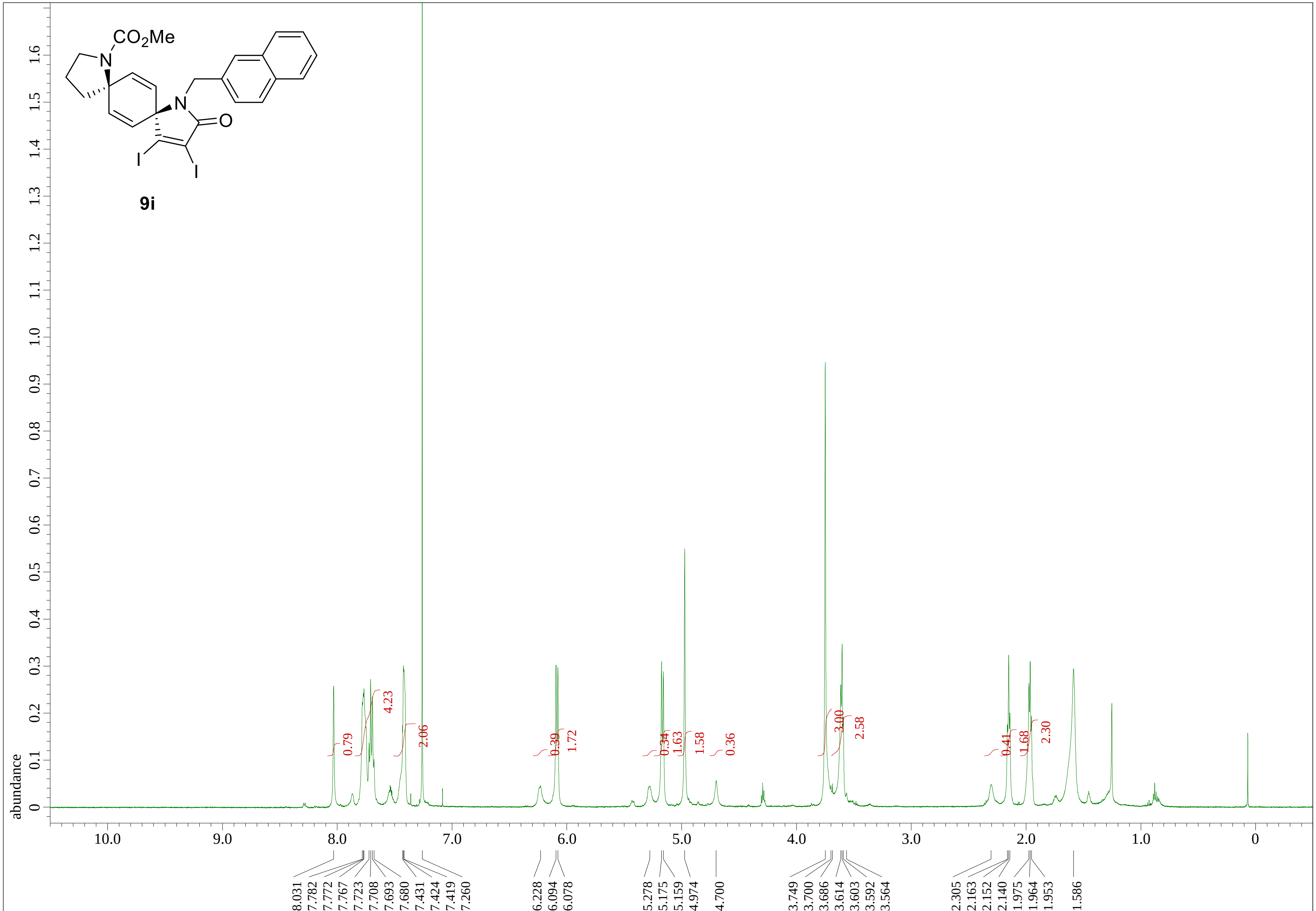
CO2Me
N
N
O
I
I
9i
abundance
8.031
7.782
7.772
7.767
7.723
7.708
7.693
7.680
7.431
7.424
7.419
7.260
6.228
6.094
6.078
5.278
5.175
5.159
4.974
4.700
3.749
3.700
3.686
3.614
3.603
3.592
3.564
2.305
2.163
2.152
2.140
1.975
1.964
1.953
1.586
0.79
4.23
2.06
0.39
1.72
0.34
1.63
1.58
0.36
3.00
2.58
0.41
1.68
2.30
X : parts per Million : Proton : RANGED

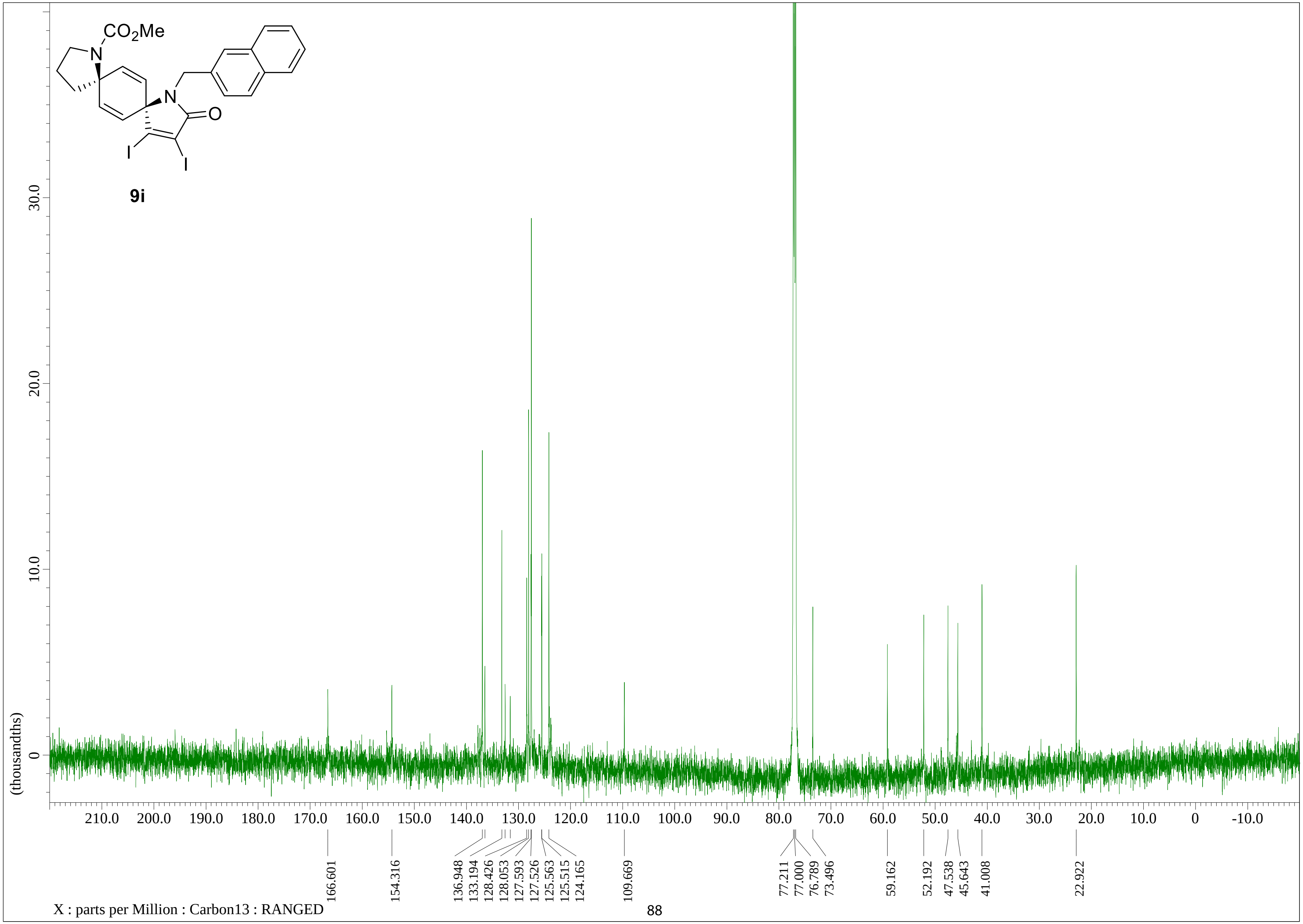
CO2Me
N
N
O
I
I
9i
(thousandths)
30.0
20.0
10.0
0
210.0 200.0 190.0 180.0 170.0 160.0 150.0 140.0 130.0 120.0 110.0 100.0 90.0 80.0 70.0 60.0 50.0 40.0 30.0 20.0 10.0 0 -10.0
166.601
154.316
136.948
133.194
128.426
128.053
127.593
127.526
125.563
125.515
124.165
109.669
77.211
77.000
76.789
73.496
59.162
52.192
47.538
45.643
41.008
22.922
X : parts per Million : Carbon13 : RANGED

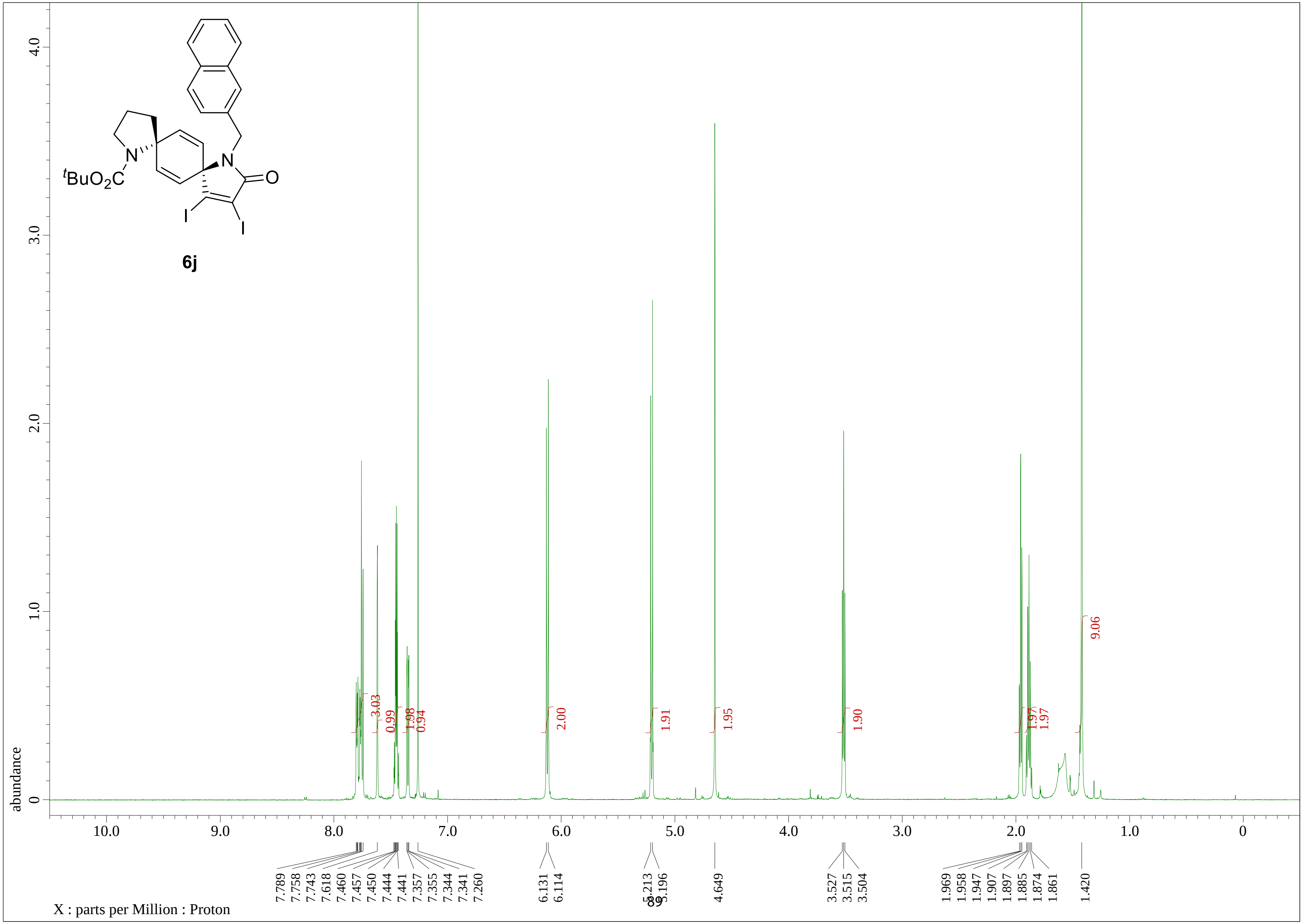

^{t}BuO$_2$C
N
N
O
I
I
6j
abundance
X : parts per Million : Proton

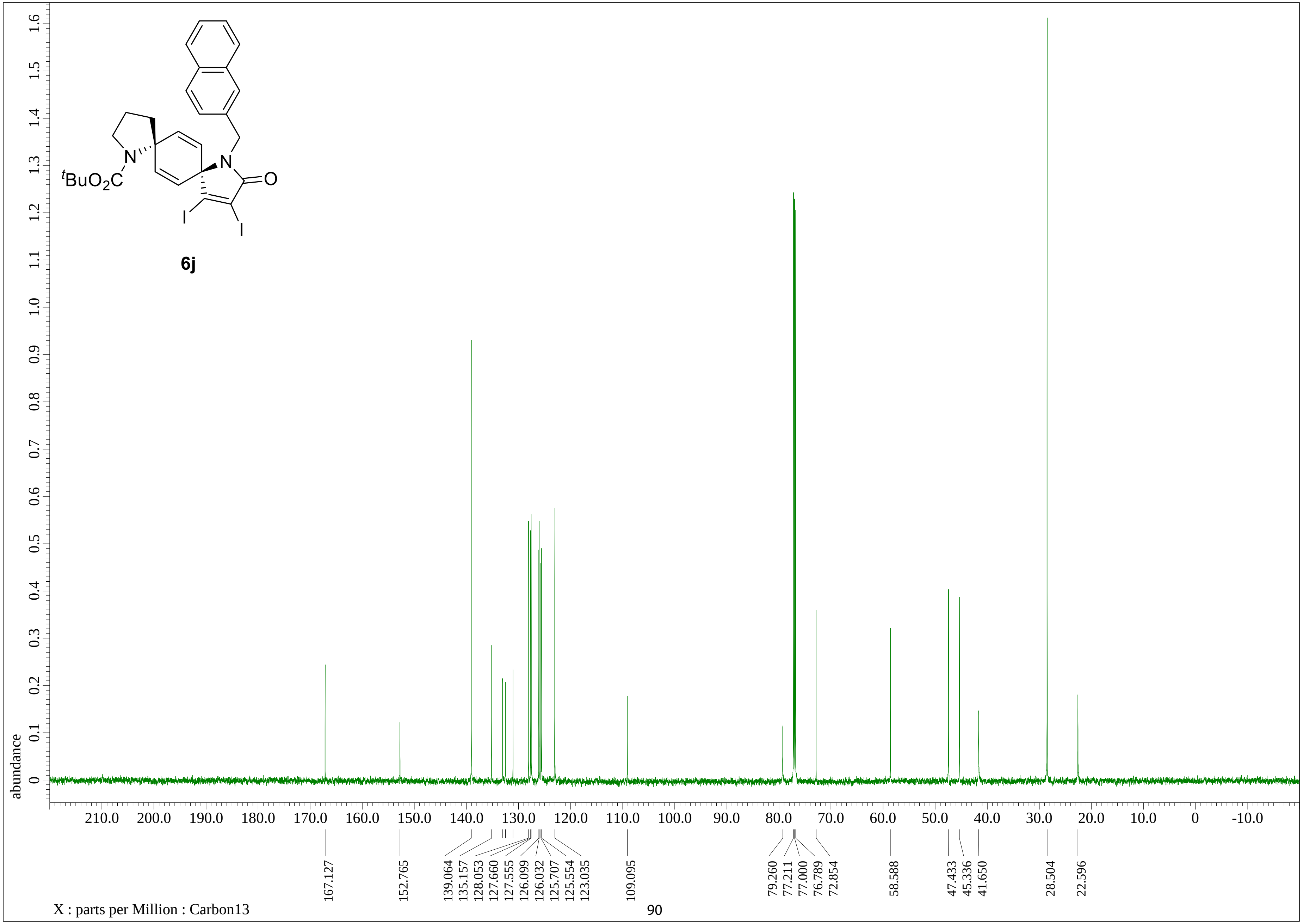

tBuO2C
N
N
O
I
I
6j
abundance
X : parts per Million : Carbon13
167.127
152.765
139.064
135.157
128.053
127.660
127.555
126.099
126.032
125.707
125.554
123.035
109.095
79.260
77.211
77.000
76.789
72.854
58.588
47.433
45.336
41.650
28.504
22.596

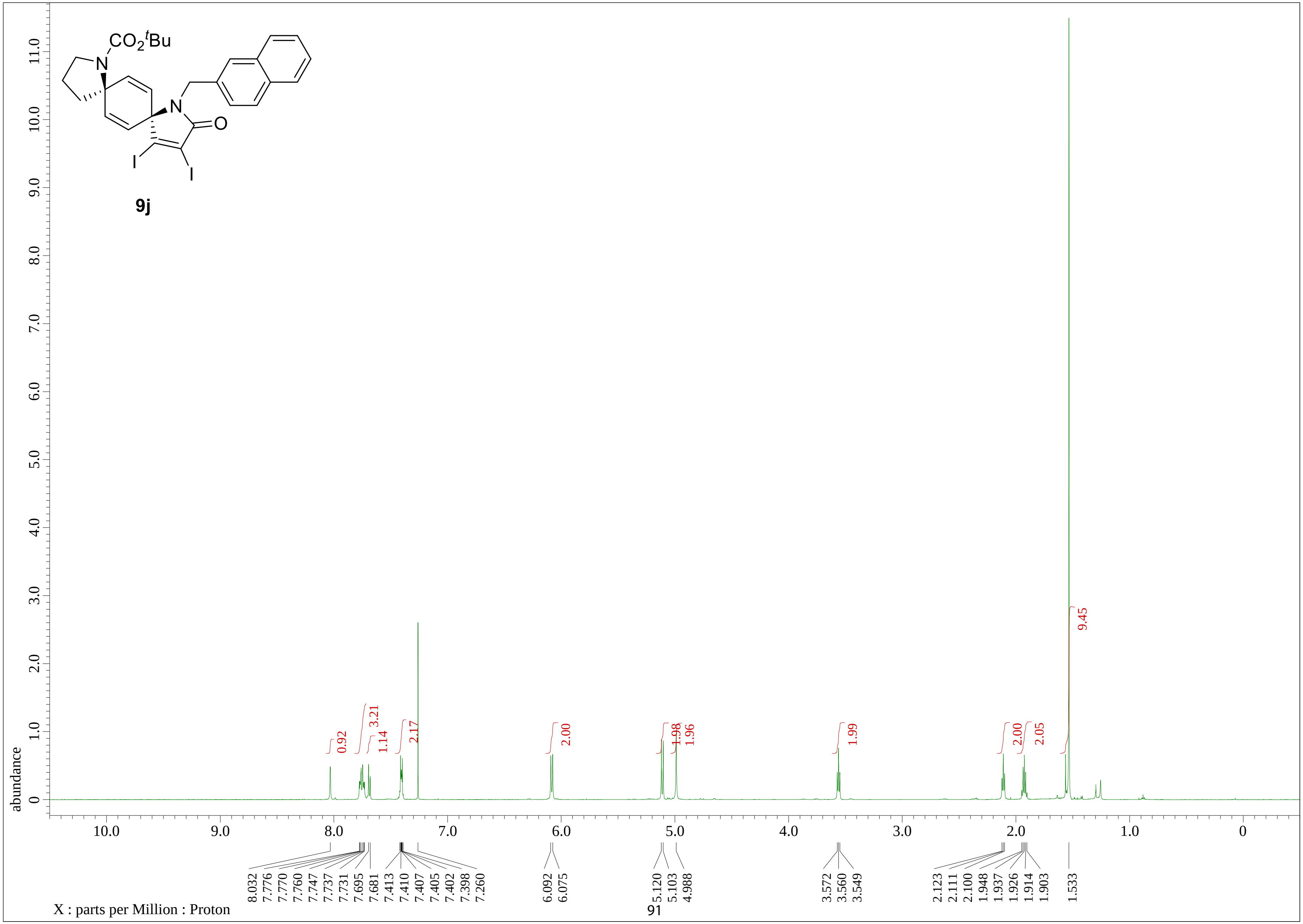
CO2tBu
N
N
O
I
I
9j
abundance
X : parts per Million : Proton
0.92
3.21
1.14
2.17
2.00
1.98
1.96
1.99
2.00
2.05
9.45
8.032
7.776
7.770
7.760
7.747
7.737
7.731
7.695
7.681
7.413
7.410
7.407
7.405
7.402
7.398
7.260
6.092
6.075
5.120
5.103
4.988
3.572
3.560
3.549
2.123
2.111
2.100
1.948
1.937
1.926
1.914
1.903
1.533

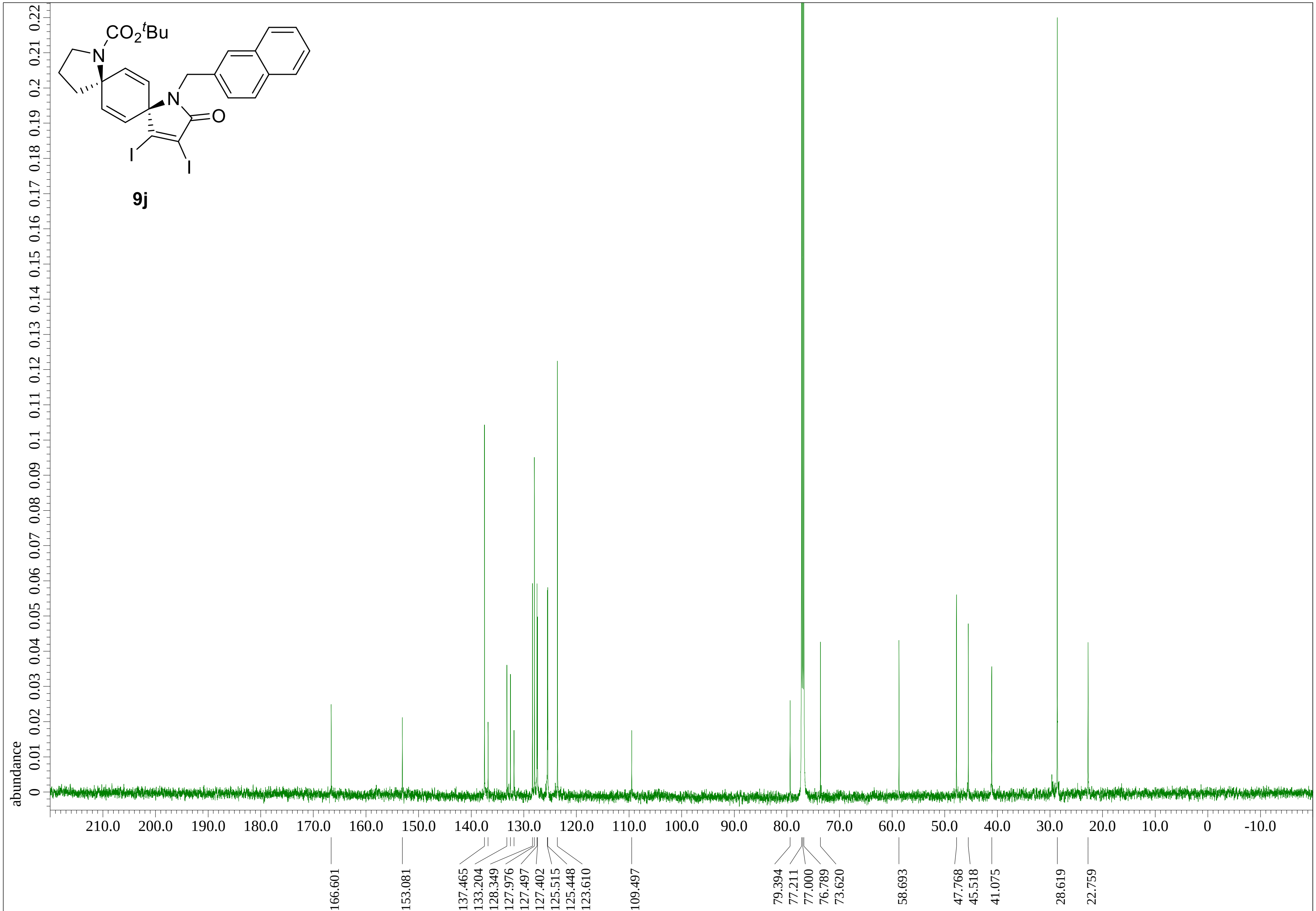
CO2tBu
N
N
O
I
I
9j
abundance
X : parts per Million : Carbon13
166.601
153.081
137.465
133.204
128.349
127.976
127.497
127.402
125.515
125.448
123.610
109.497
79.394
77.211
77.000
76.789
73.620
58.693
47.768
45.518
41.075
28.619
22.759

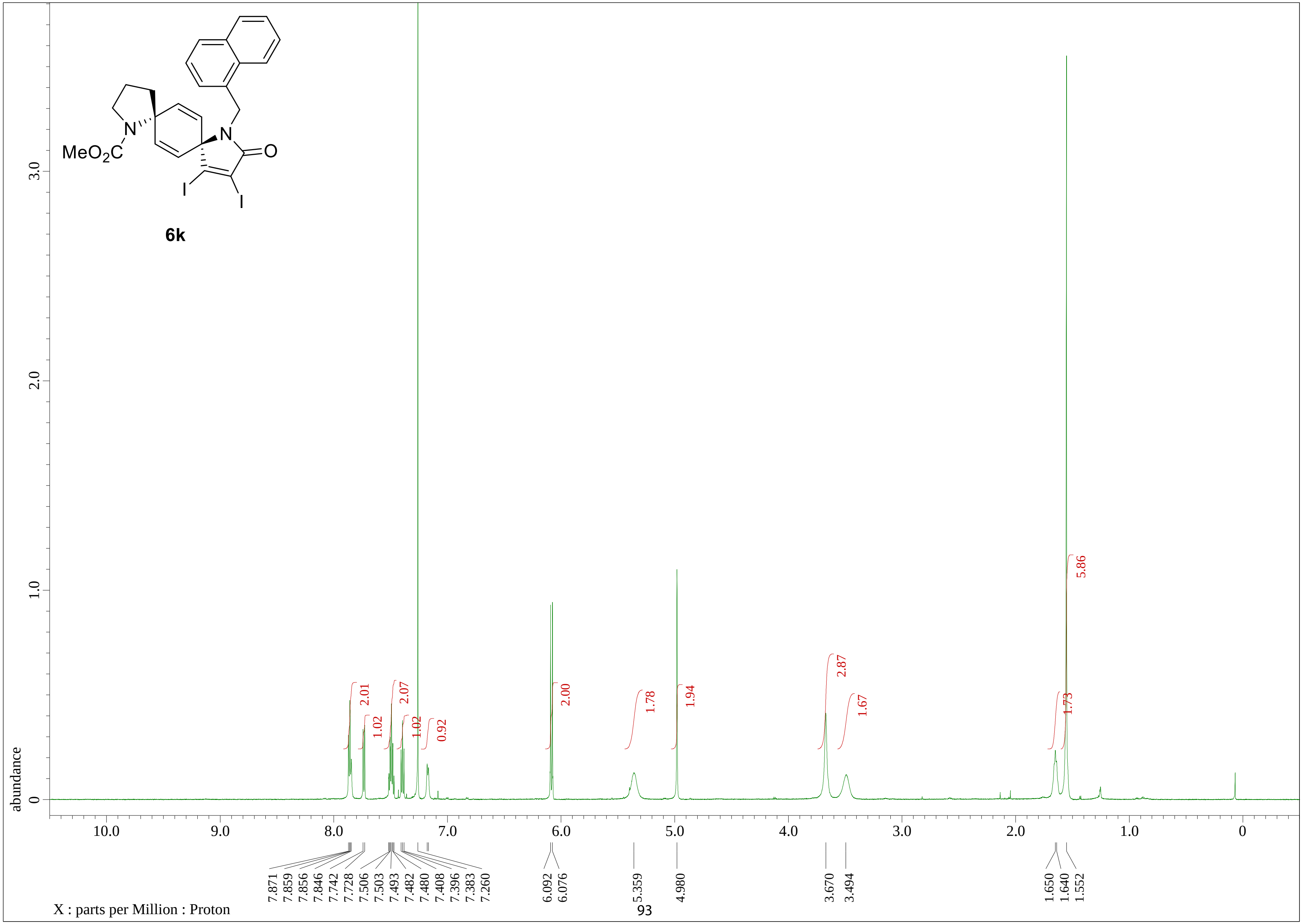
MeO2C
N
N
O
I
I
6k
abundance
3.0
2.0
1.0
0
2.01
1.02
2.07
1.02
0.92
2.00
1.78
1.94
2.87
1.67
1.73
5.86
10.0
9.0
8.0
7.0
6.0
5.0
4.0
3.0
2.0
1.0
0
7.871 7.859 7.856 7.846 7.742 7.728 7.506 7.503 7.493 7.482 7.480 7.408 7.396 7.383 7.260
6.092 6.076
5.359
4.980
3.670 3.494
1.650 1.640 1.552
X : parts per Million : Proton

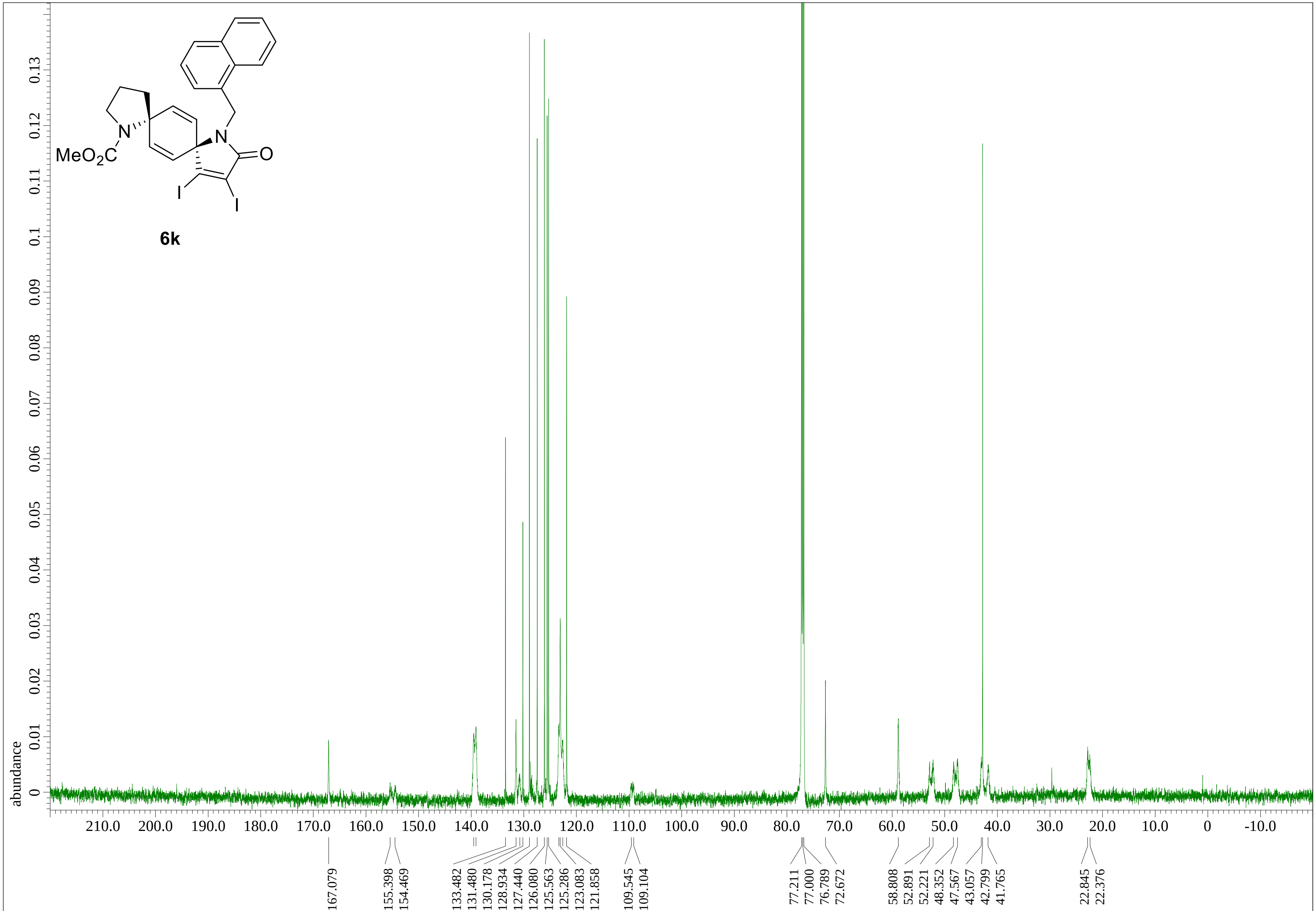
MeO2C
N
N
O
I
I
6k
abundance
0.13
0.12
0.11
0.1
0.09
0.08
0.07
0.06
0.05
0.04
0.03
0.02
0.01
0
210.0 200.0 190.0 180.0 170.0 160.0 150.0 140.0 130.0 120.0 110.0 100.0 90.0 80.0 70.0 60.0 50.0 40.0 30.0 20.0 10.0 0 -10.0
167.079
155.398
154.469
133.482
131.480
130.178
128.934
127.440
126.080
125.563
125.286
123.083
121.858
109.545
109.104
77.211
77.000
76.789
72.672
58.808
52.891
52.221
48.352
47.567
43.057
42.799
41.765
22.845
22.376
X : parts per Million : Carbon13

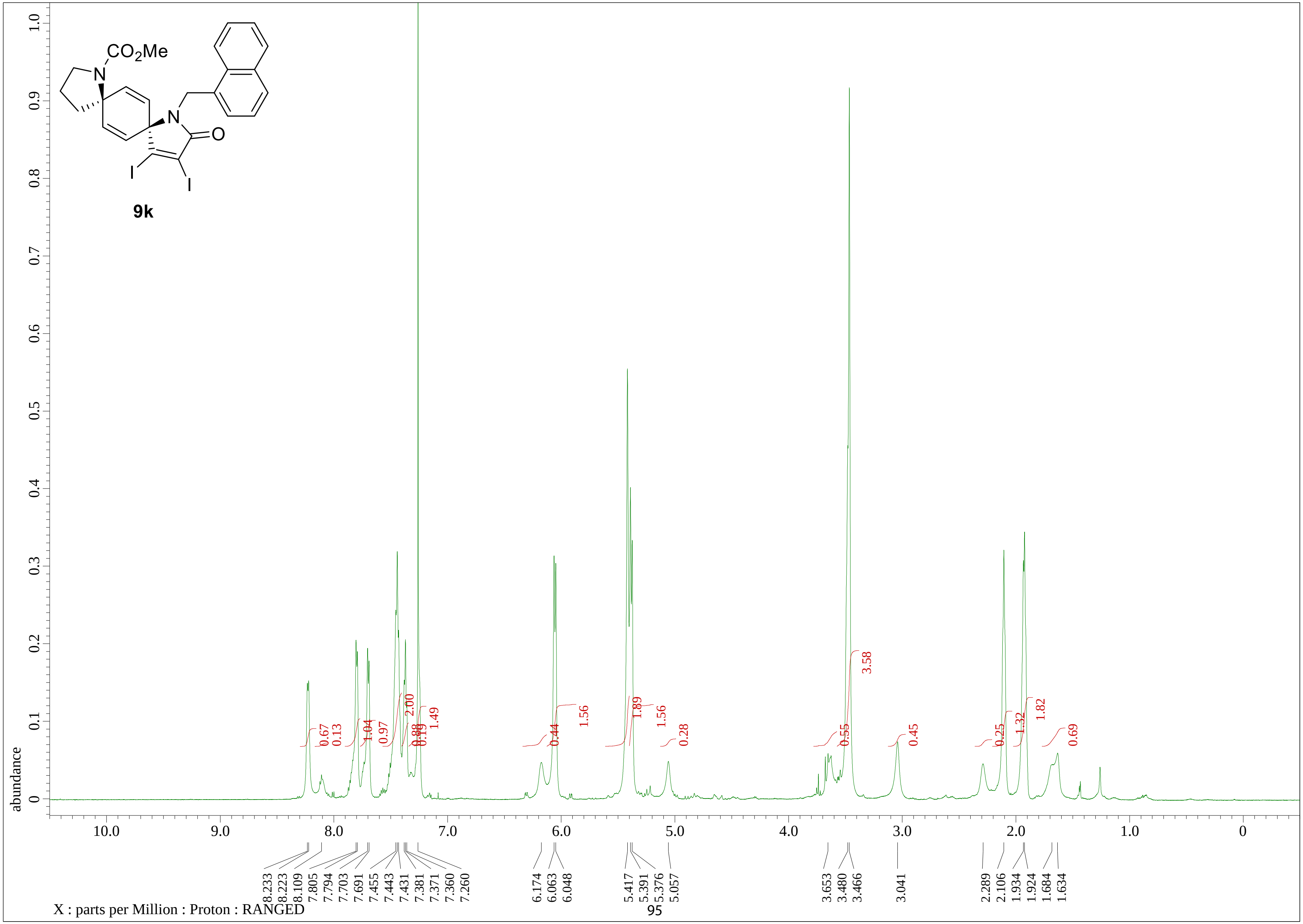
CO2Me
N
N
O
I
I
9k
abundance
1.0
0.9
0.8
0.7
0.6
0.5
0.4
0.3
0.2
0.1
0
10.0
9.0
8.0
7.0
6.0
5.0
4.0
3.0
2.0
1.0
0
0.67
0.13
1.04
0.97
2.00
0.88
0.19
1.49
0.44
1.56
1.89
1.56
0.28
3.58
0.55
0.45
0.25
1.32
1.82
0.69
8.233
8.223
8.109
7.805
7.794
7.703
7.691
7.455
7.443
7.431
7.381
7.371
7.360
7.260
6.174
6.063
6.048
5.417
5.391
5.376
5.057
3.653
3.480
3.466
3.041
2.289
2.106
1.934
1.924
1.684
1.634
X : parts per Million : Proton : RANGED

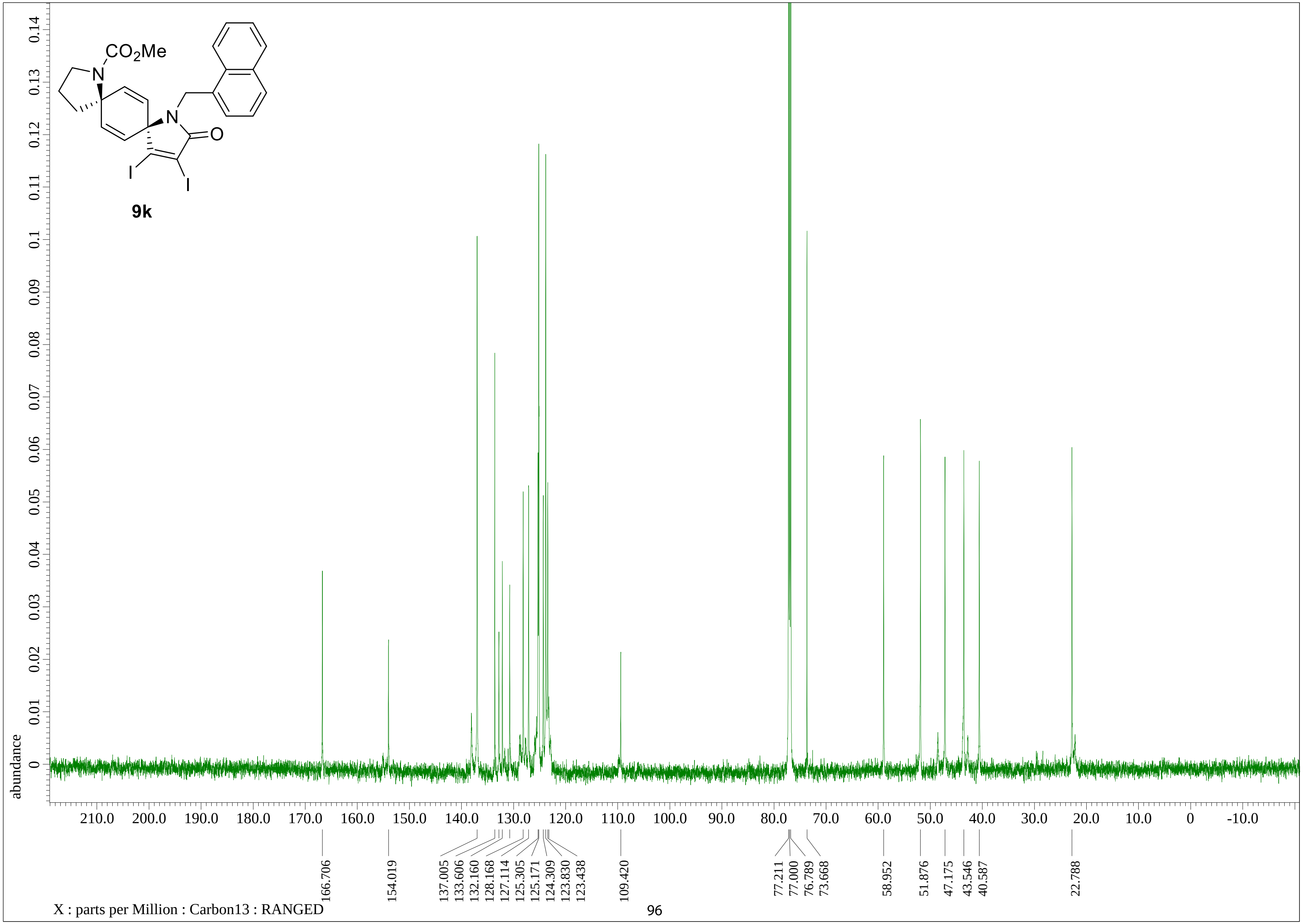

CO2Me
N
N
O
I
I
9k
abundance
X : parts per Million : Carbon13 : RANGED
166.706
154.019
137.005
133.606
132.160
128.168
127.114
125.305
125.171
124.309
123.830
123.438
109.420
77.211
77.000
76.789
73.668
58.952
51.876
47.175
43.546
40.587
22.788

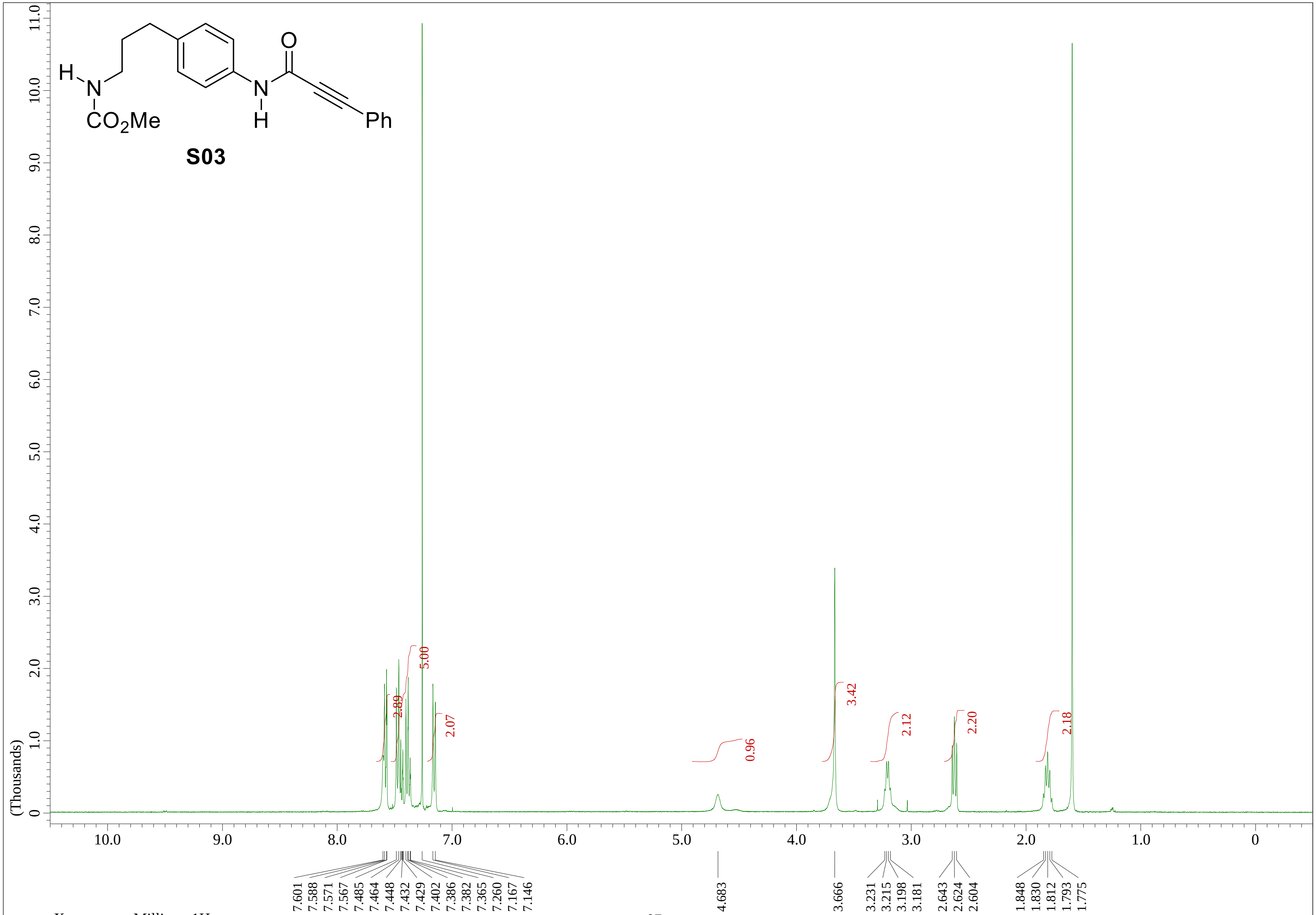

S03
(Thousands)
X : parts per Million : 1H
7.601
7.588
7.571
7.567
7.485
7.464
7.448
7.432
7.429
7.402
7.386
7.382
7.365
7.260
7.167
7.146
4.683
3.666
3.231
3.215
3.198
3.181
2.643
2.624
2.604
1.848
1.830
1.812
1.793
1.775

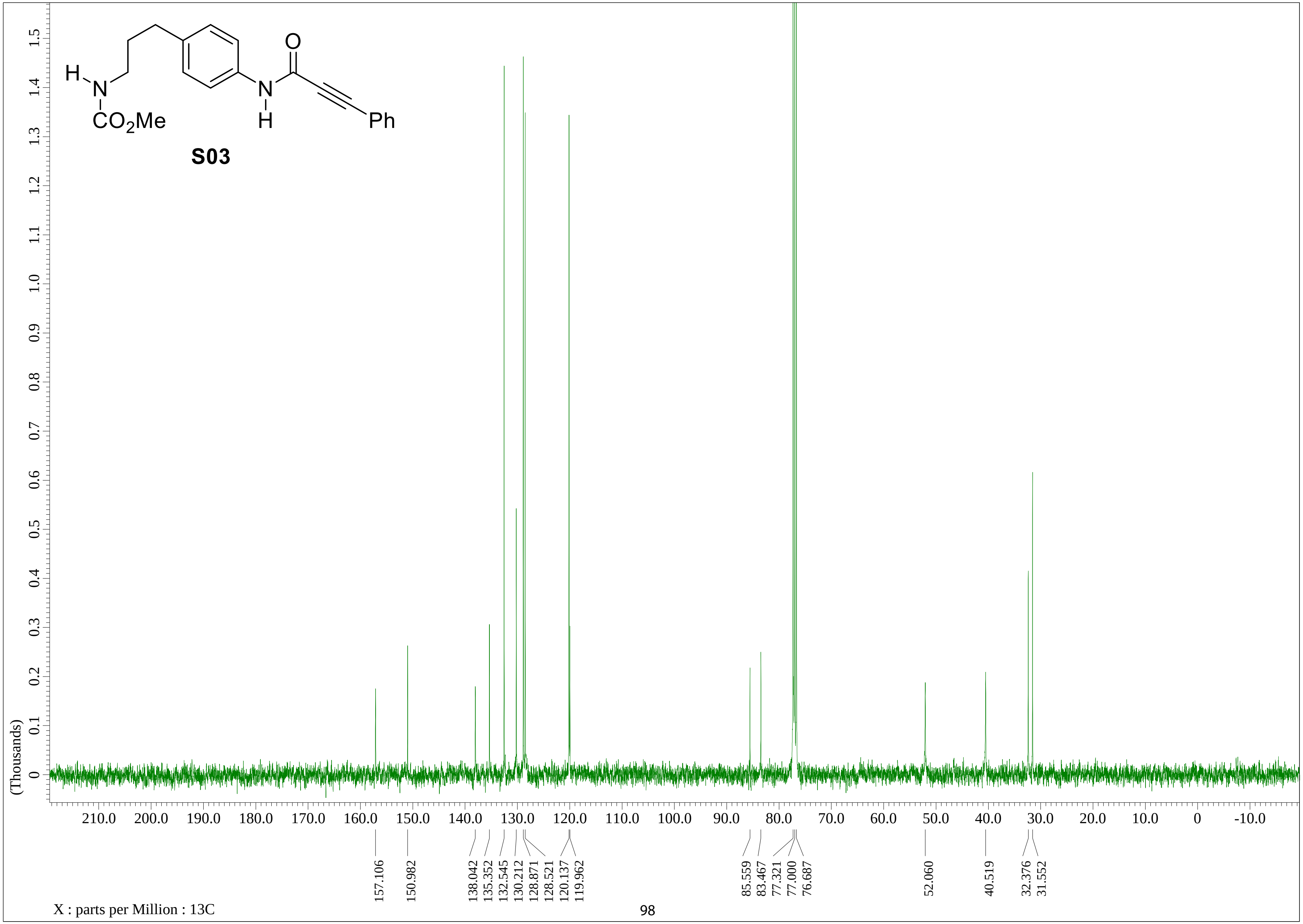
H
N
CO2Me
N
H
O
Ph
S03
(Thousands)
0
0.1
0.2
0.3
0.4
0.5
0.6
0.7
0.8
0.9
1.0
1.1
1.2
1.3
1.4
1.5
210.0
200.0
190.0
180.0
170.0
160.0
150.0
140.0
130.0
120.0
110.0
100.0
90.0
80.0
70.0
60.0
50.0
40.0
30.0
20.0
10.0
0
-10.0
157.106
150.982
138.042
135.352
132.545
130.212
128.871
128.521
120.137
119.962
85.559
83.467
77.321
77.000
76.687
52.060
40.519
32.376
31.552
X : parts per Million : 13C

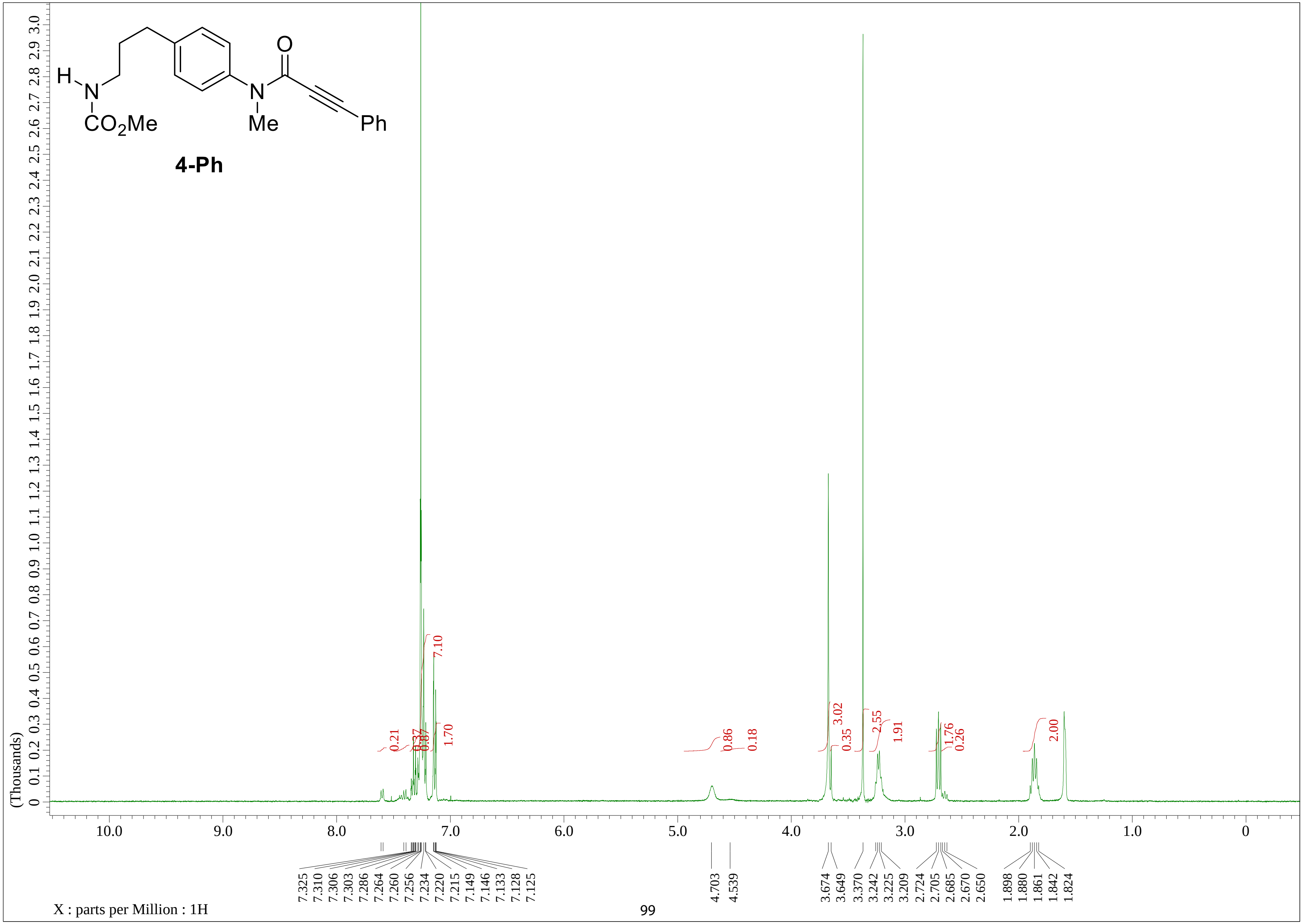

H
N
CO2Me
O
N
Me
Ph
4-Ph
(Thousands)
X : parts per Million : 1H

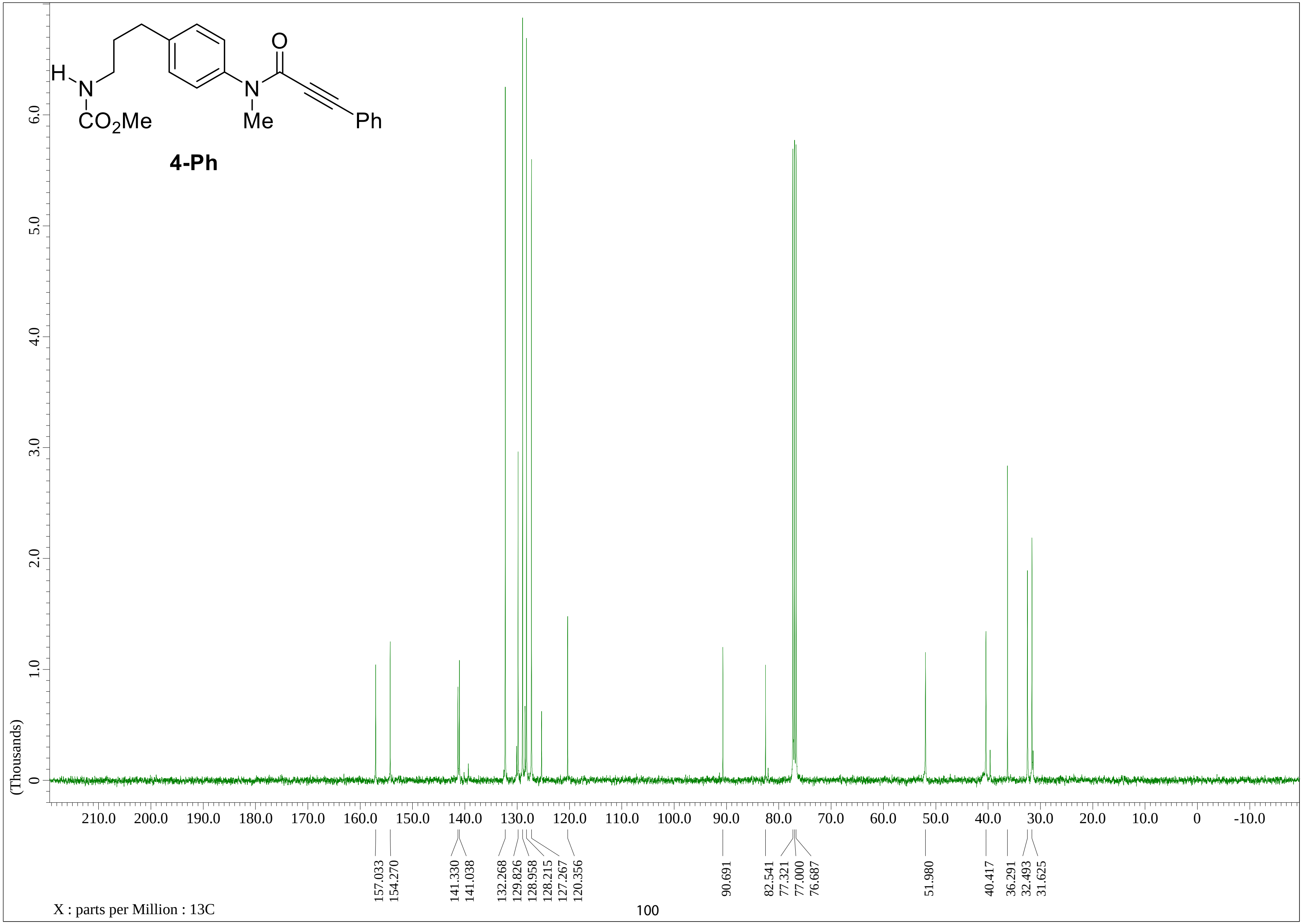

H
N
CO2Me
N
Me
O
Ph
4-Ph
(Thousands)
6.0
5.0
4.0
3.0
2.0
1.0
0
210.0 200.0 190.0 180.0 170.0 160.0 150.0 140.0 130.0 120.0 110.0 100.0 90.0 80.0 70.0 60.0 50.0 40.0 30.0 20.0 10.0 0 -10.0
157.033
154.270
141.330
141.038
132.268
129.826
128.958
128.215
127.267
120.356
90.691
82.541
77.321
77.000
76.687
51.980
40.417
36.291
32.493
31.625
X : parts per Million : 13C

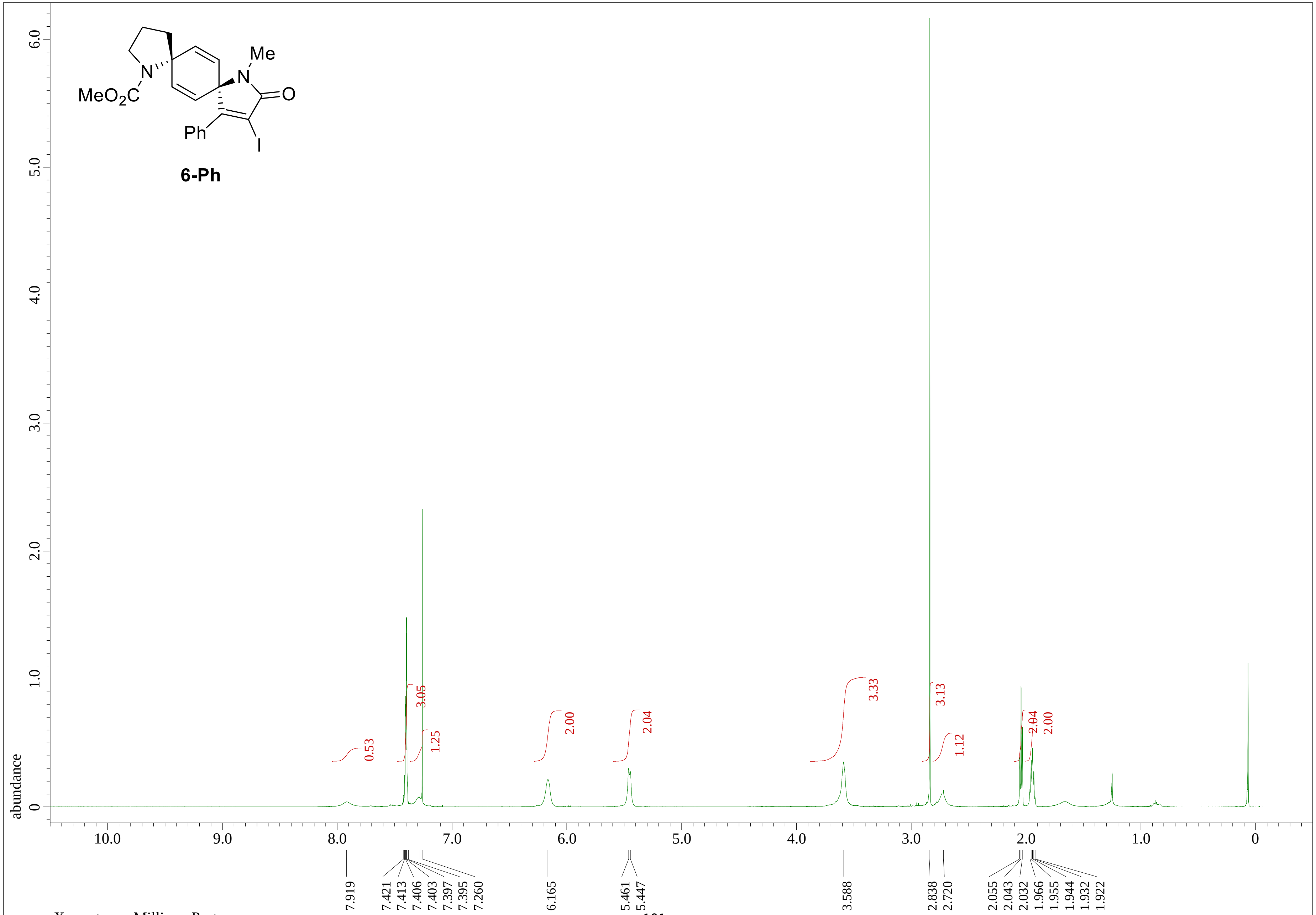
MeO2C
N
Me
N
O
Ph
I
6-Ph
abundance
X : parts per Million : Proton
7.919
7.421
7.413
7.406
7.403
7.397
7.395
7.260
6.165
5.461
5.447
3.588
2.838
2.720
2.055
2.043
2.032
1.966
1.955
1.944
1.932
1.922
0.53
3.05
1.25
2.00
2.04
3.33
3.13
1.12
2.04
2.00

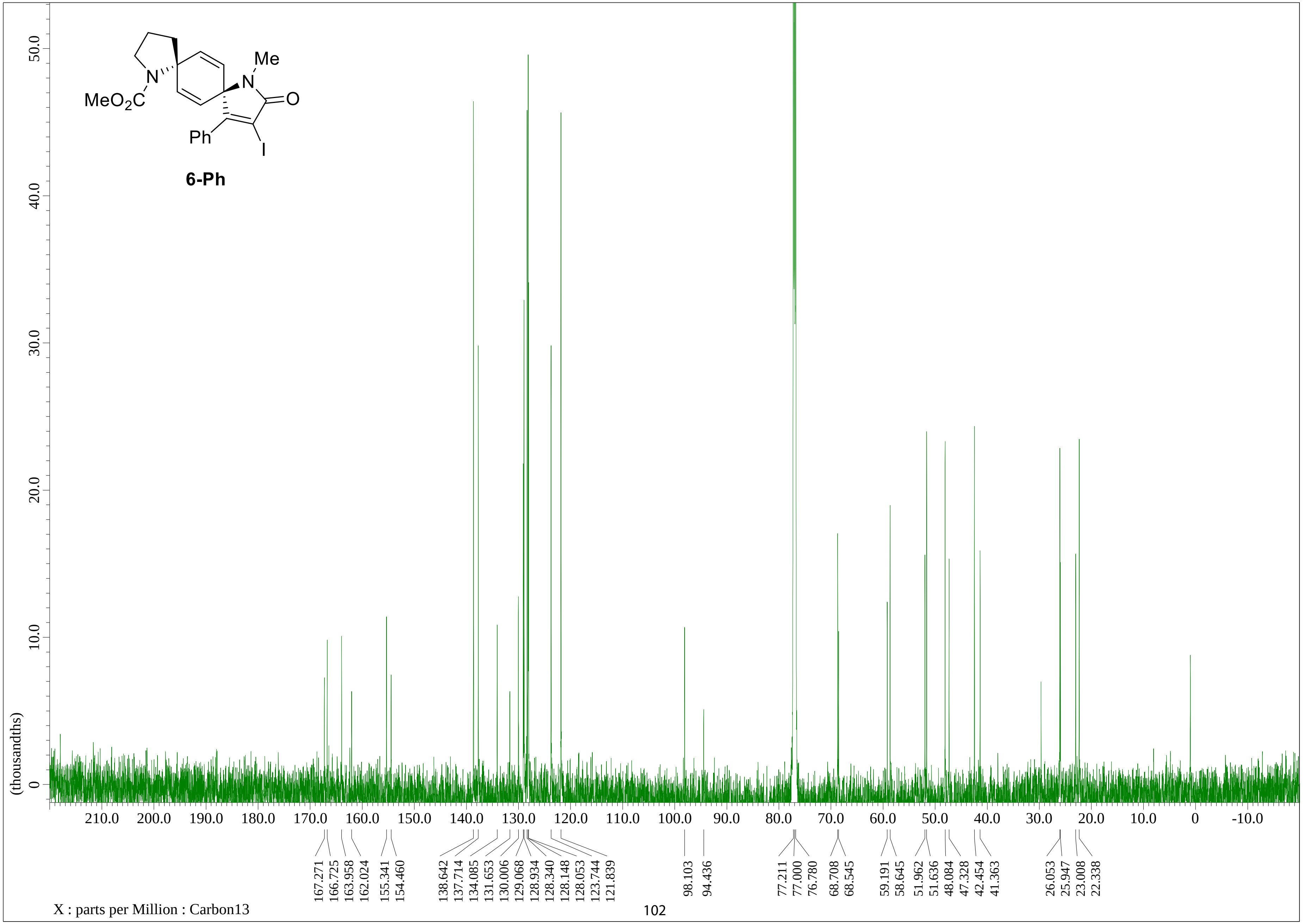

Me
MeO2C
N
N
O
Ph
I
6-Ph
(thousandths)
50.0
40.0
30.0
20.0
10.0
0
210.0
200.0
190.0
180.0
170.0
160.0
150.0
140.0
130.0
120.0
110.0
100.0
90.0
80.0
70.0
60.0
50.0
40.0
30.0
20.0
10.0
0
-10.0
167.271
166.725
163.958
162.024
155.341
154.460
138.642
137.714
134.085
131.653
130.006
129.068
128.934
128.340
128.148
128.053
123.744
121.839
98.103
94.436
77.211
77.000
76.780
68.708
68.545
59.191
58.645
51.962
51.636
48.084
47.328
42.454
41.363
26.053
25.947
23.008
22.338
X : parts per Million : Carbon13

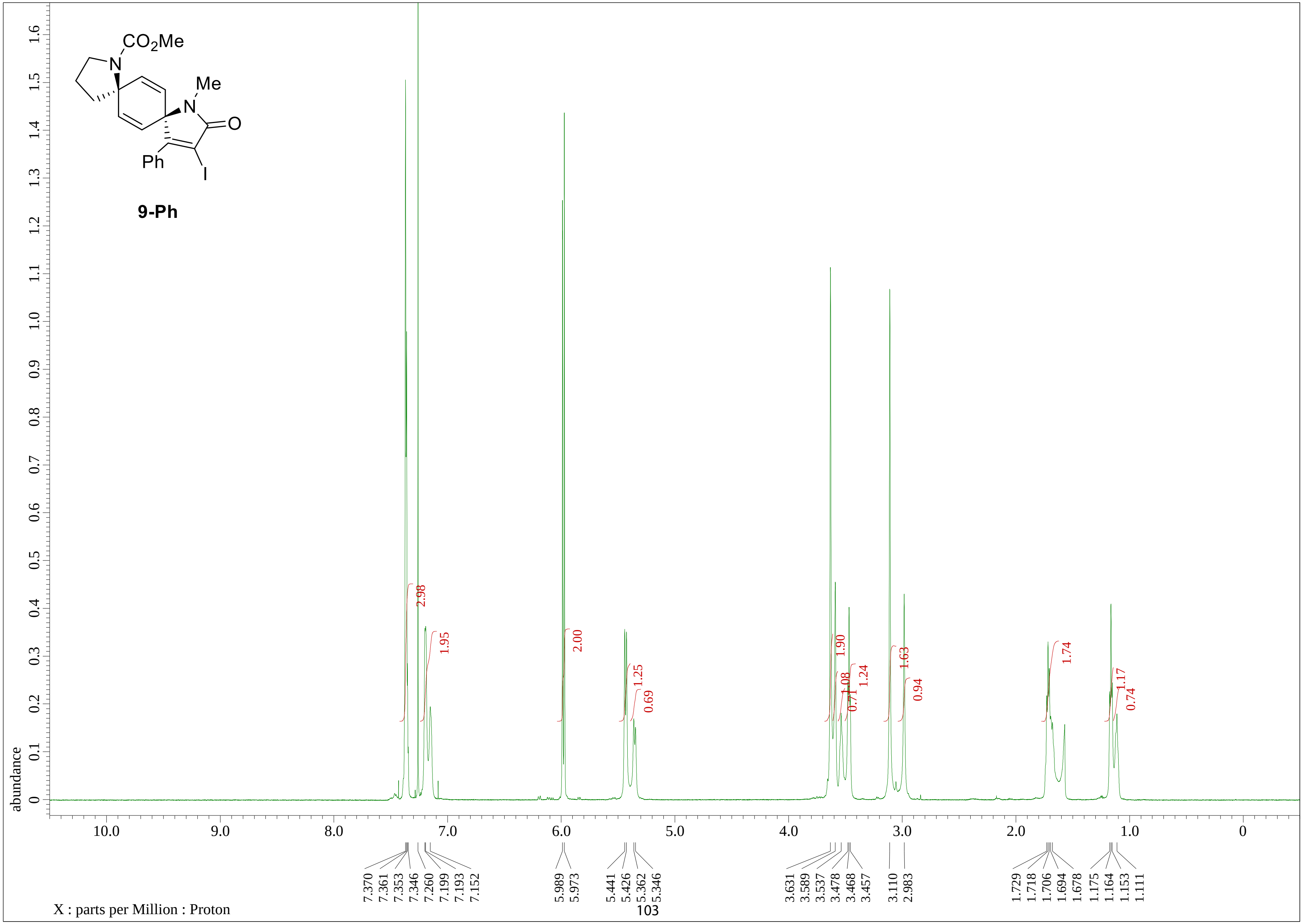

CO2Me
N
Me
N
O
Ph
I
9-Ph
abundance
X : parts per Million : Proton
10.0 9.0 8.0 7.0 6.0 5.0 4.0 3.0 2.0 1.0 0
2.98 1.95 2.00 1.25 0.69 1.90 1.08 0.71 1.24 1.63 0.94 1.74 1.17 0.74
7.370 7.361 7.353 7.346 7.260 7.199 7.193 7.152
5.989 5.973
5.441 5.426 5.362 5.346
3.631 3.589 3.537 3.478 3.468 3.457
3.110 2.983
1.729 1.718 1.706 1.694 1.678 1.175 1.164 1.153 1.111

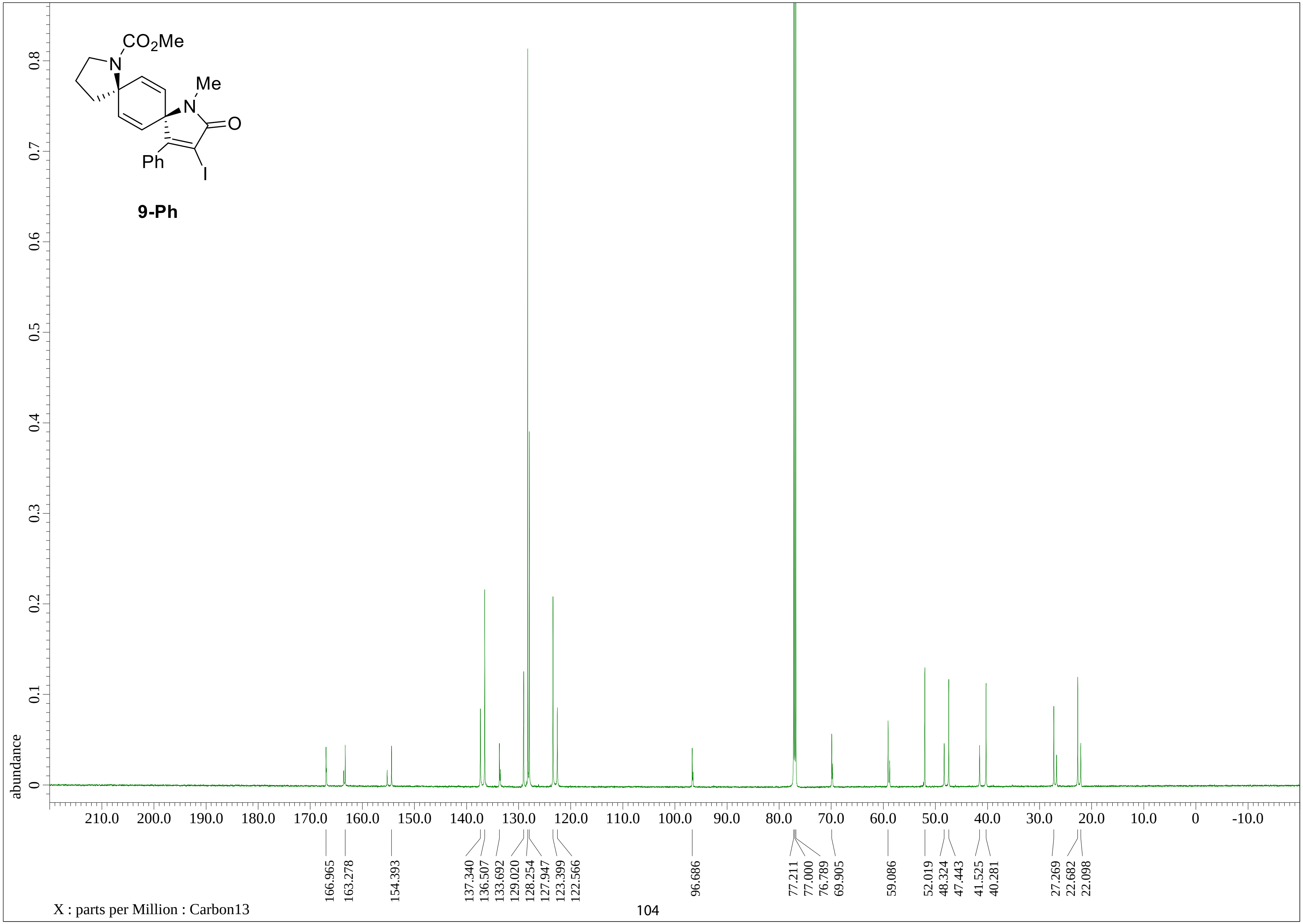
CO2Me
N
Me
N
O
Ph
I
9-Ph
abundance
0.8
0.7
0.6
0.5
0.4
0.3
0.2
0.1
0
210.0 200.0 190.0 180.0 170.0 160.0 150.0 140.0 130.0 120.0 110.0 100.0 90.0 80.0 70.0 60.0 50.0 40.0 30.0 20.0 10.0 0 -10.0
166.965
163.278
154.393
137.340
136.507
133.692
129.020
128.254
127.947
123.399
122.566
96.686
77.211
77.000
76.789
69.905
59.086
52.019
48.324
47.443
41.525
40.281
27.269
22.682
22.098
X : parts per Million : Carbon13

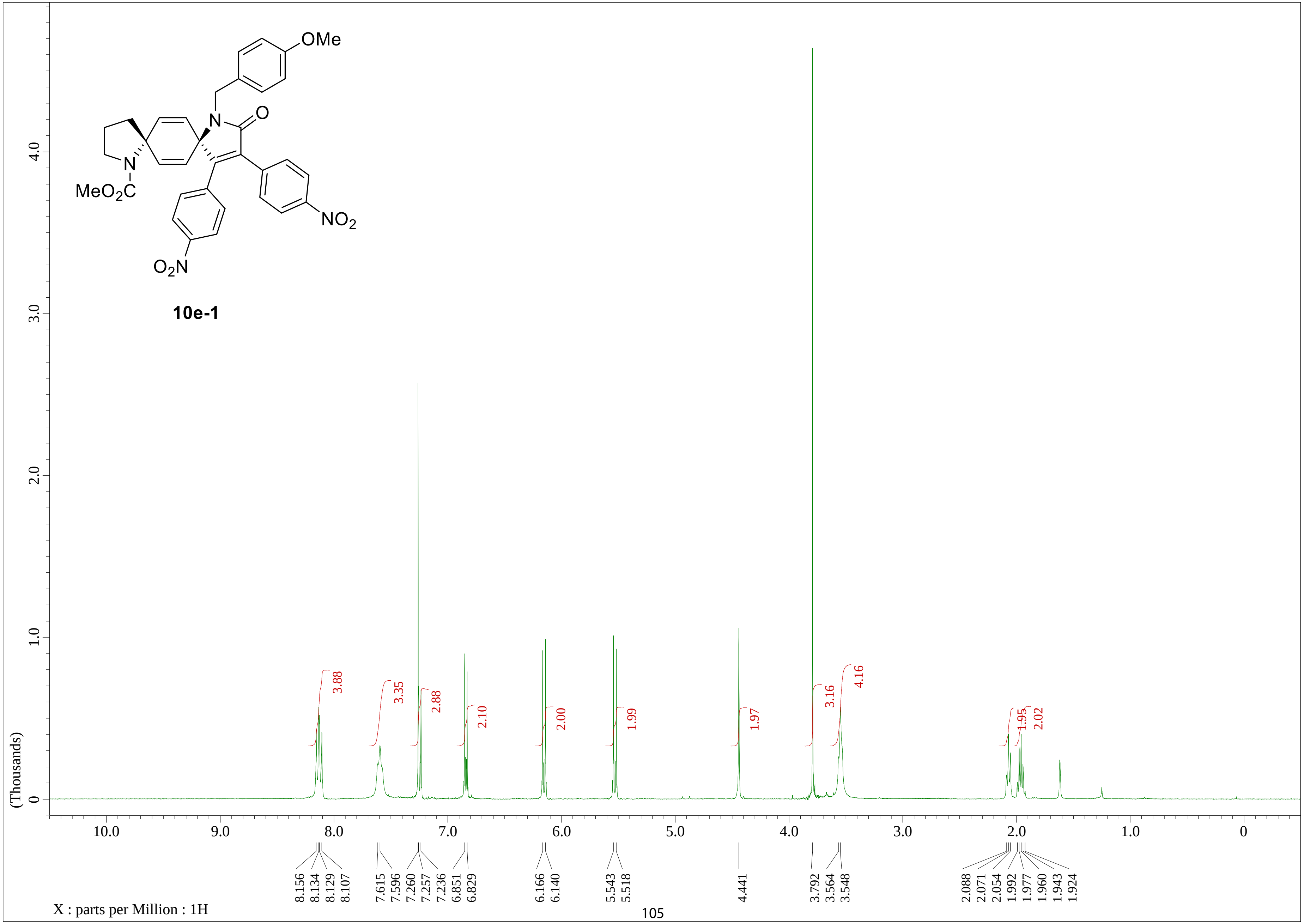
OMe
N
O
N
MeO2C
NO2
O2N
10e-1
(Thousands)
4.0
3.0
2.0
1.0
0
3.88
3.35
2.88
2.10
2.00
1.99
1.97
3.16
4.16
1.95
2.02
10.0
9.0
8.0
7.0
6.0
5.0
4.0
3.0
2.0
1.0
0
8.156
8.134
8.129
8.107
7.615
7.596
7.260
7.257
7.236
6.851
6.829
6.166
6.140
5.543
5.518
4.441
3.792
3.564
3.548
2.088
2.071
2.054
1.992
1.977
1.960
1.943
1.924
X : parts per Million : 1H

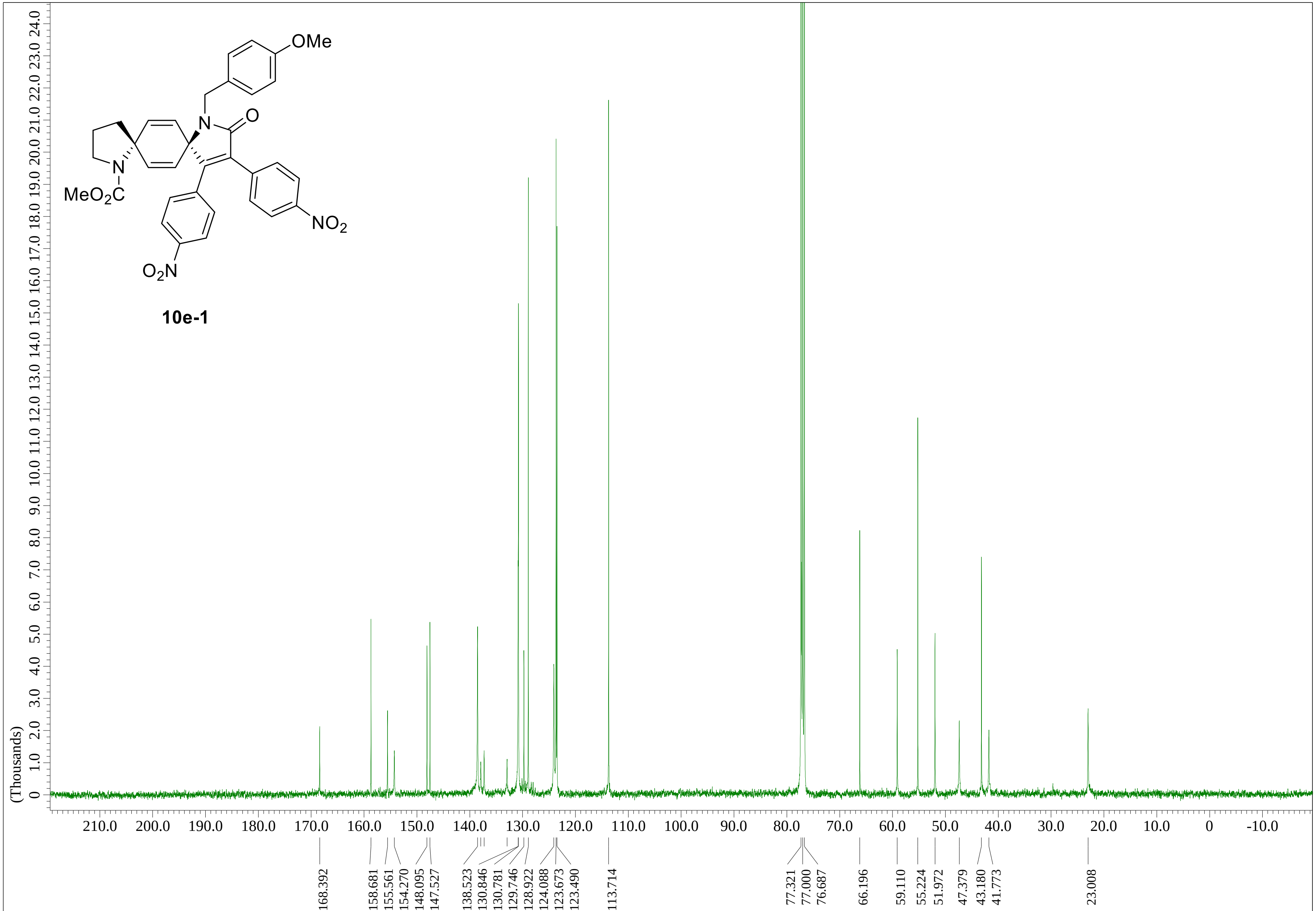

OMe
N
O
N
MeO2C
NO2
O2N
10e-1
(Thousands)
168.392
158.681
155.561
154.270
148.095
147.527
138.523
130.846
130.781
129.746
128.922
124.088
123.673
123.490
113.714
77.321
77.000
76.687
66.196
59.110
55.224
51.972
47.379
43.180
41.773
23.008
X : parts per Million : 13C : RANGED

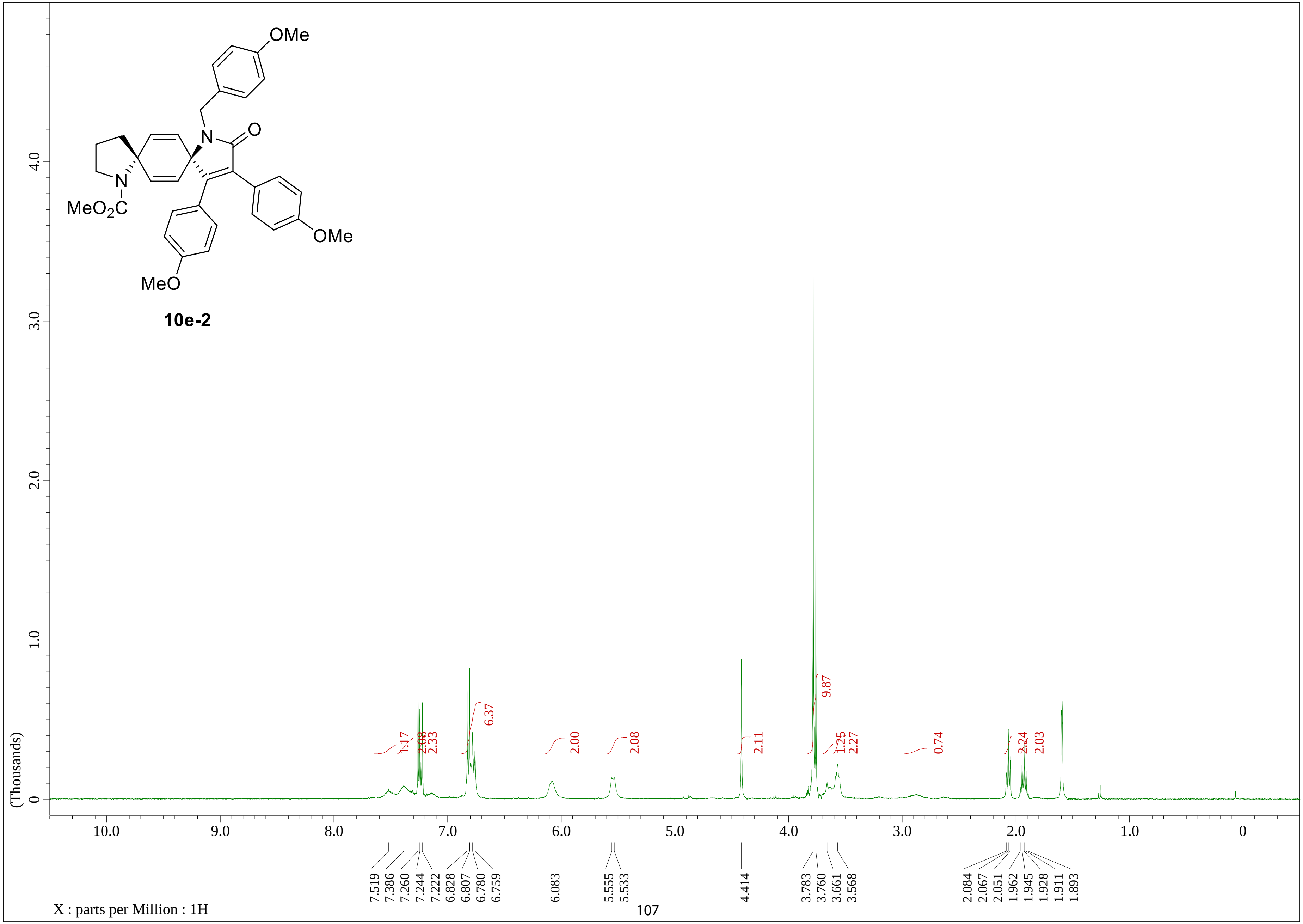

10e-2
(Thousands)
X : parts per Million : 1H
7.519 7.386 7.260 7.244 7.222 6.828 6.807 6.780 6.759
6.083
5.555 5.533
4.414
3.783 3.760 3.661 3.568
2.084 2.067 2.051 1.962 1.945 1.928 1.911 1.893
1.17 2.08 2.33 6.37 2.00 2.08 2.11 9.87 1.25 2.27 0.74 2.24 2.03

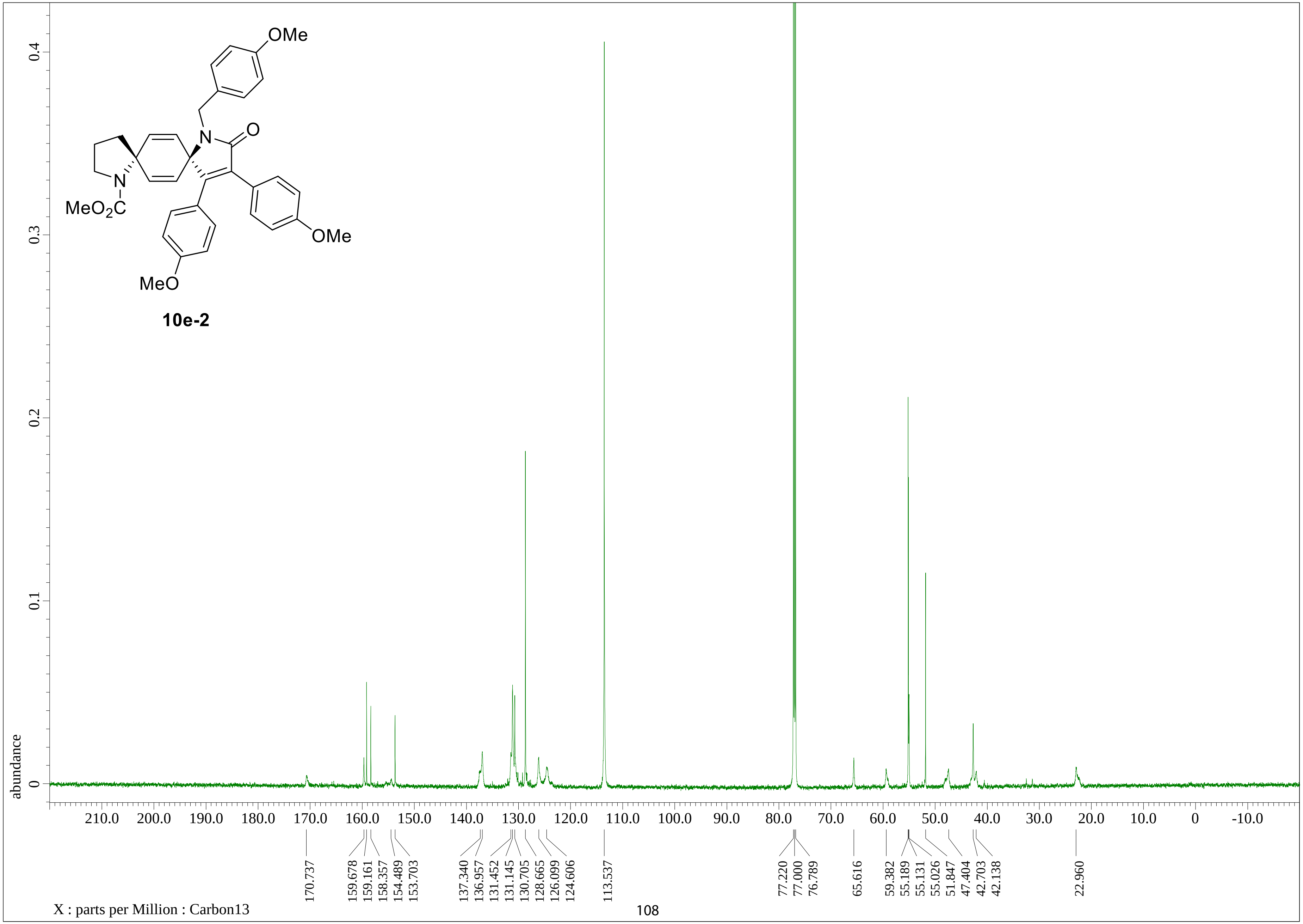
OMe
N
O
MeO2C
OMe
MeO
10e-2
abundance
0.4
0.3
0.2
0.1
0
210.0 200.0 190.0 180.0 170.0 160.0 150.0 140.0 130.0 120.0 110.0 100.0 90.0 80.0 70.0 60.0 50.0 40.0 30.0 20.0 10.0 0 -10.0
170.737
159.678
159.161
158.357
154.489
153.703
137.340
136.957
131.452
131.145
130.705
128.665
126.099
124.606
113.537
77.220
77.000
76.789
65.616
59.382
55.189
55.131
55.026
51.847
47.404
42.703
42.138
22.960
X : parts per Million : Carbon13

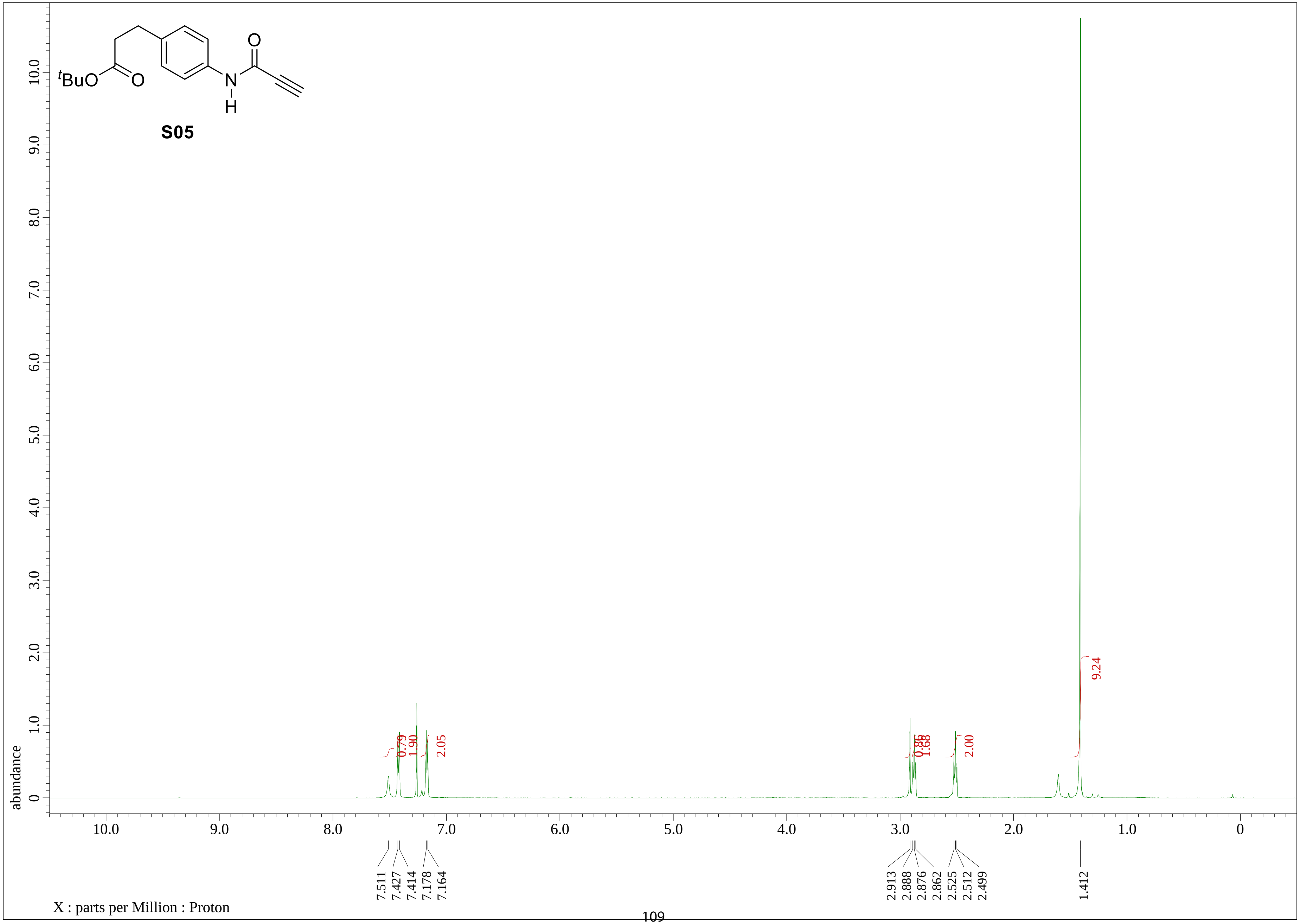

tBuO
O
N
H
O
S05
abundance
X : parts per Million : Proton
7.511
7.427
7.414
7.178
7.164
2.913
2.888
2.876
2.862
2.525
2.512
2.499
1.412
0.79
1.90
2.05
2.00
9.24

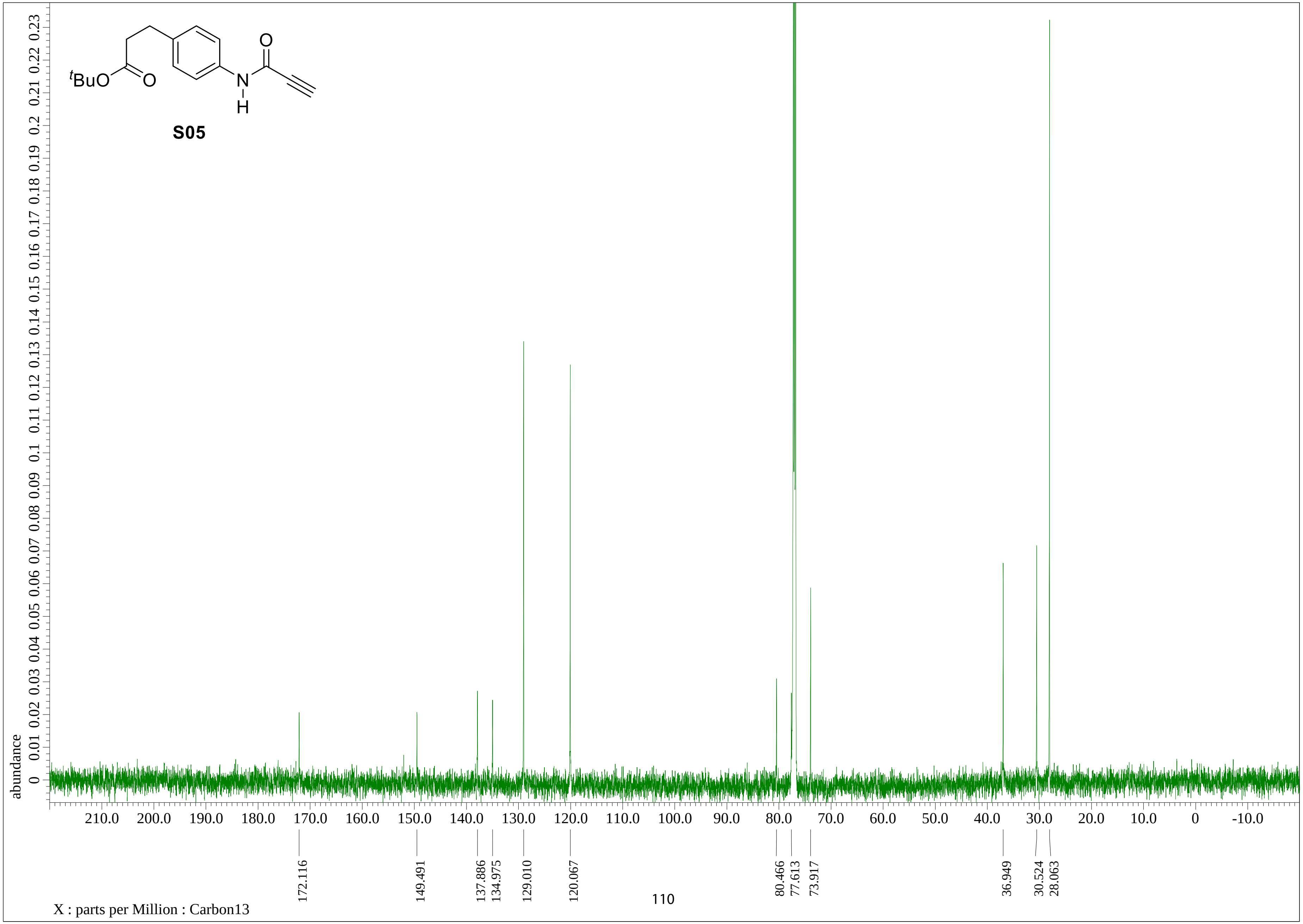

tBuO
O
N
H
O
S05
abundance
X : parts per Million : Carbon13
172.116
149.491
137.886
134.975
129.010
120.067
80.466
77.613
73.917
36.949
30.524
28.063

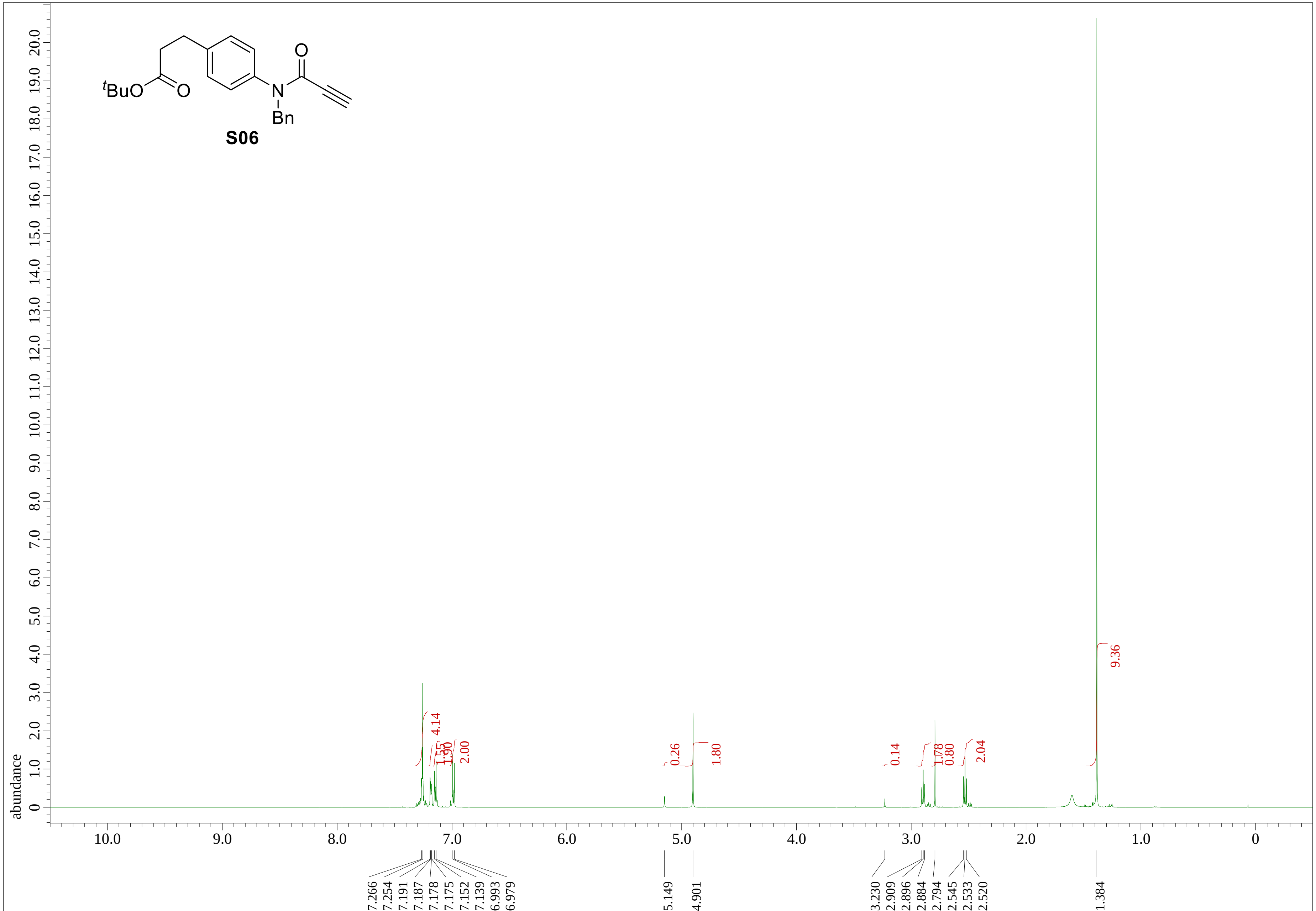

tBuO
O
N
Bn
O
S06
abundance
X : parts per Million : Proton
7.266
7.254
7.191
7.187
7.178
7.175
7.152
7.139
6.993
6.979
5.149
4.901
3.230
2.909
2.896
2.884
2.794
2.545
2.533
2.520
1.384
4.14
1.55
1.90
2.00
0.26
1.80
0.14
1.78
0.80
2.04
9.36

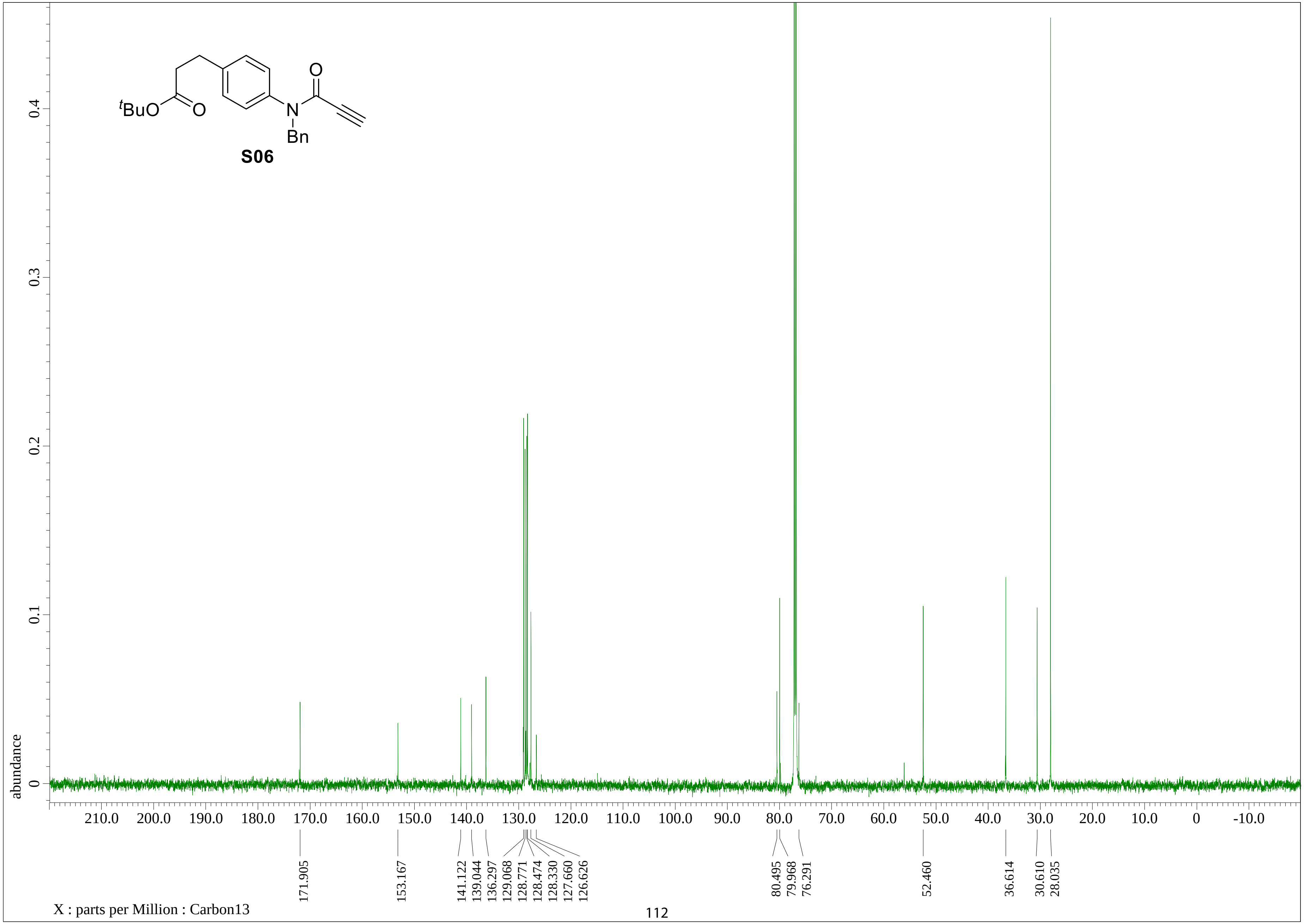

tBuO
O
N
O
Bn
S06
abundance
0.4
0.3
0.2
0.1
0
210.0 200.0 190.0 180.0 170.0 160.0 150.0 140.0 130.0 120.0 110.0 100.0 90.0 80.0 70.0 60.0 50.0 40.0 30.0 20.0 10.0 0 -10.0
171.905
153.167
141.122
139.044
136.297
129.068
128.771
128.474
128.330
127.660
126.626
80.495
79.968
76.291
52.460
36.614
30.610
28.035
X : parts per Million : Carbon13

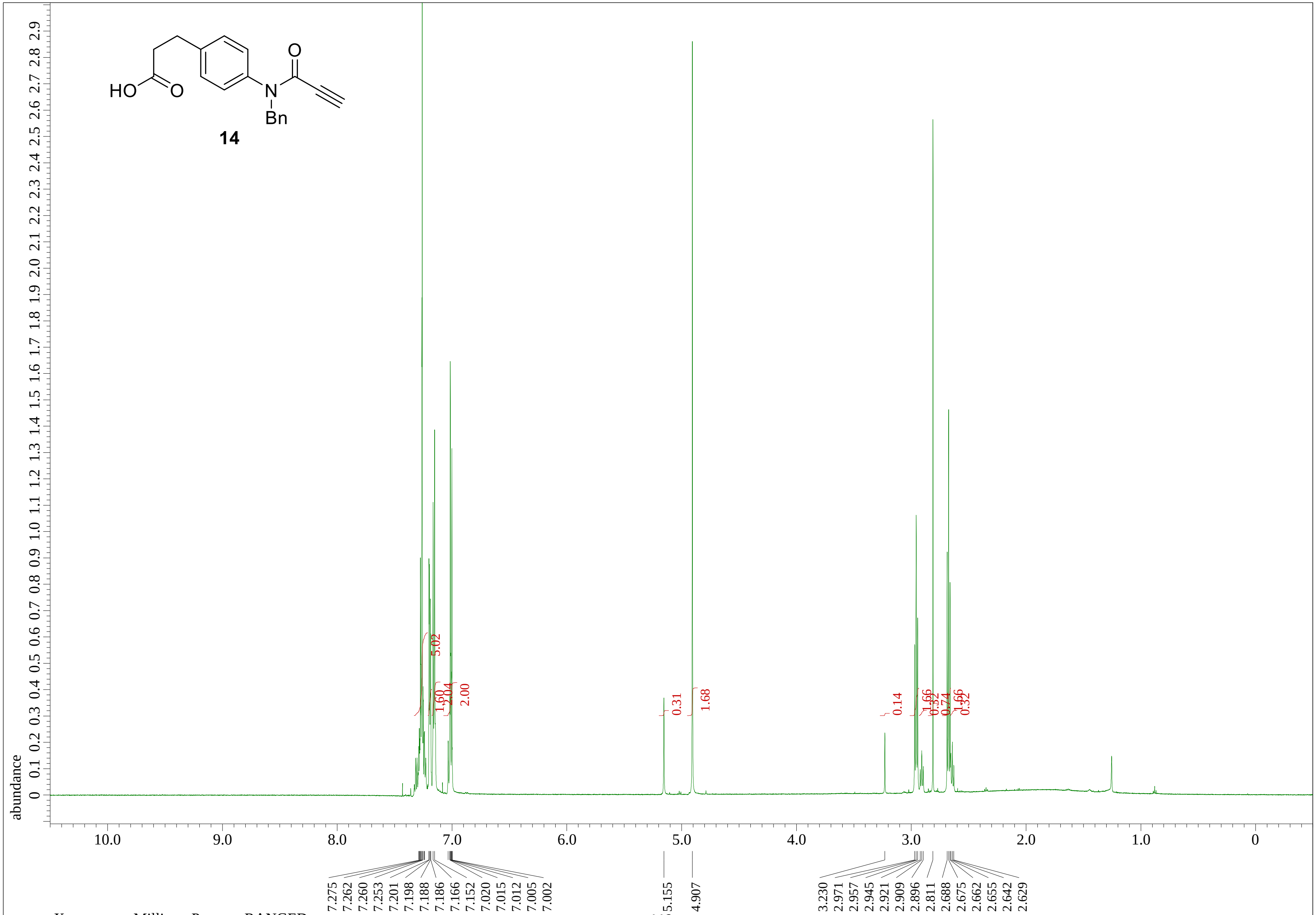
HO
O
N
O
Bn
14
abundance
X : parts per Million : Proton : RANGED
10.0 9.0 8.0 7.0 6.0 5.0 4.0 3.0 2.0 1.0 0
5.02
1.60
2.04
2.00
0.31
1.68
0.14
1.66
0.32
0.74
1.66
0.32
7.275 7.262 7.260 7.253 7.201 7.198 7.188 7.186 7.166 7.152 7.020 7.015 7.012 7.005 7.002
5.155
4.907
3.230 2.971 2.957 2.945 2.921 2.909 2.896 2.811 2.688 2.675 2.662 2.655 2.642 2.629

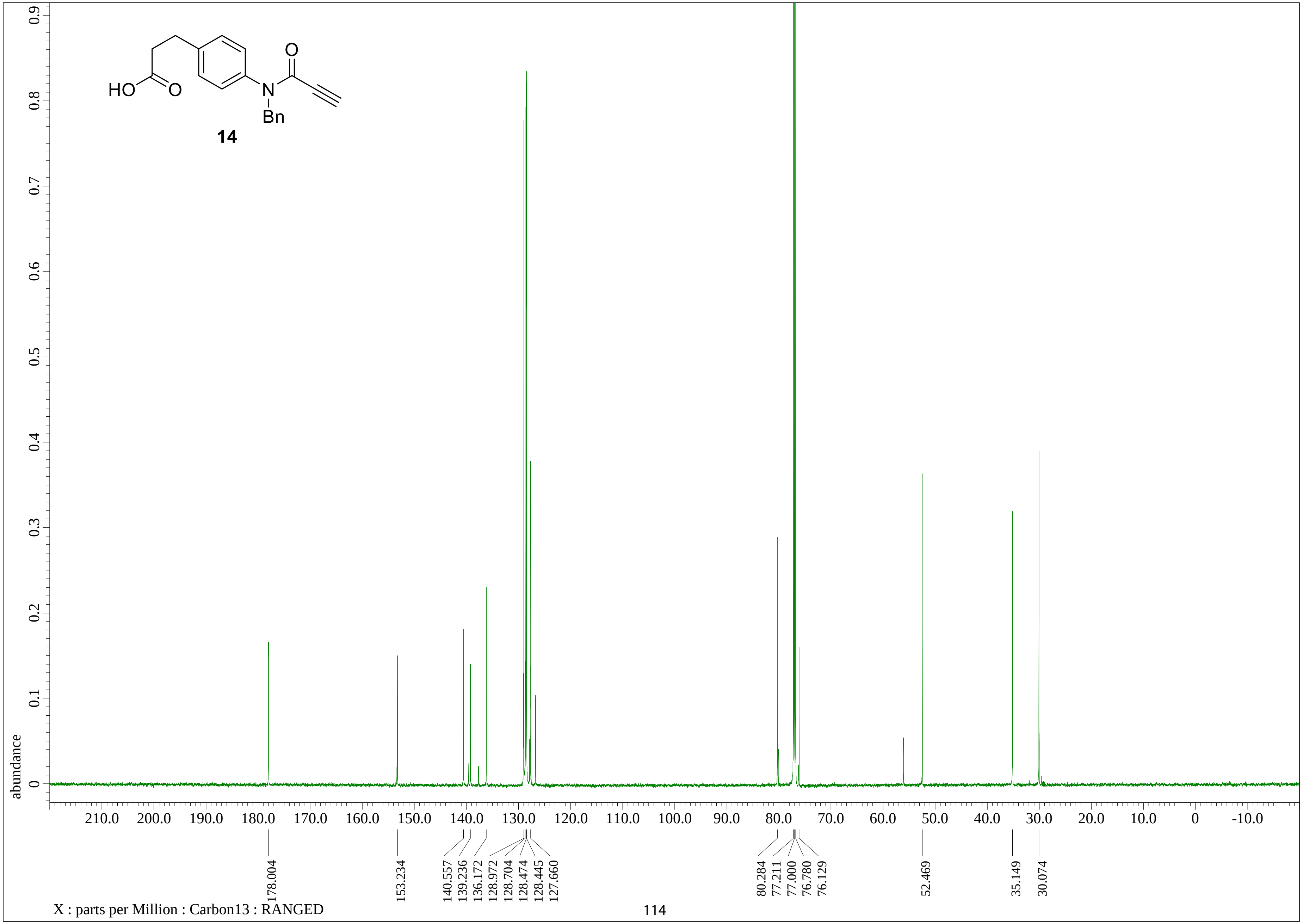
HO
O
O
N
Bn
14
abundance
0.9
0.8
0.7
0.6
0.5
0.4
0.3
0.2
0.1
0
210.0 200.0 190.0 180.0 170.0 160.0 150.0 140.0 130.0 120.0 110.0 100.0 90.0 80.0 70.0 60.0 50.0 40.0 30.0 20.0 10.0 0 -10.0
178.004
153.234
140.557
139.236
136.172
128.972
128.704
128.474
128.445
127.660
80.284
77.211
77.000
76.780
76.129
52.469
35.149
30.074
X : parts per Million : Carbon13 : RANGED

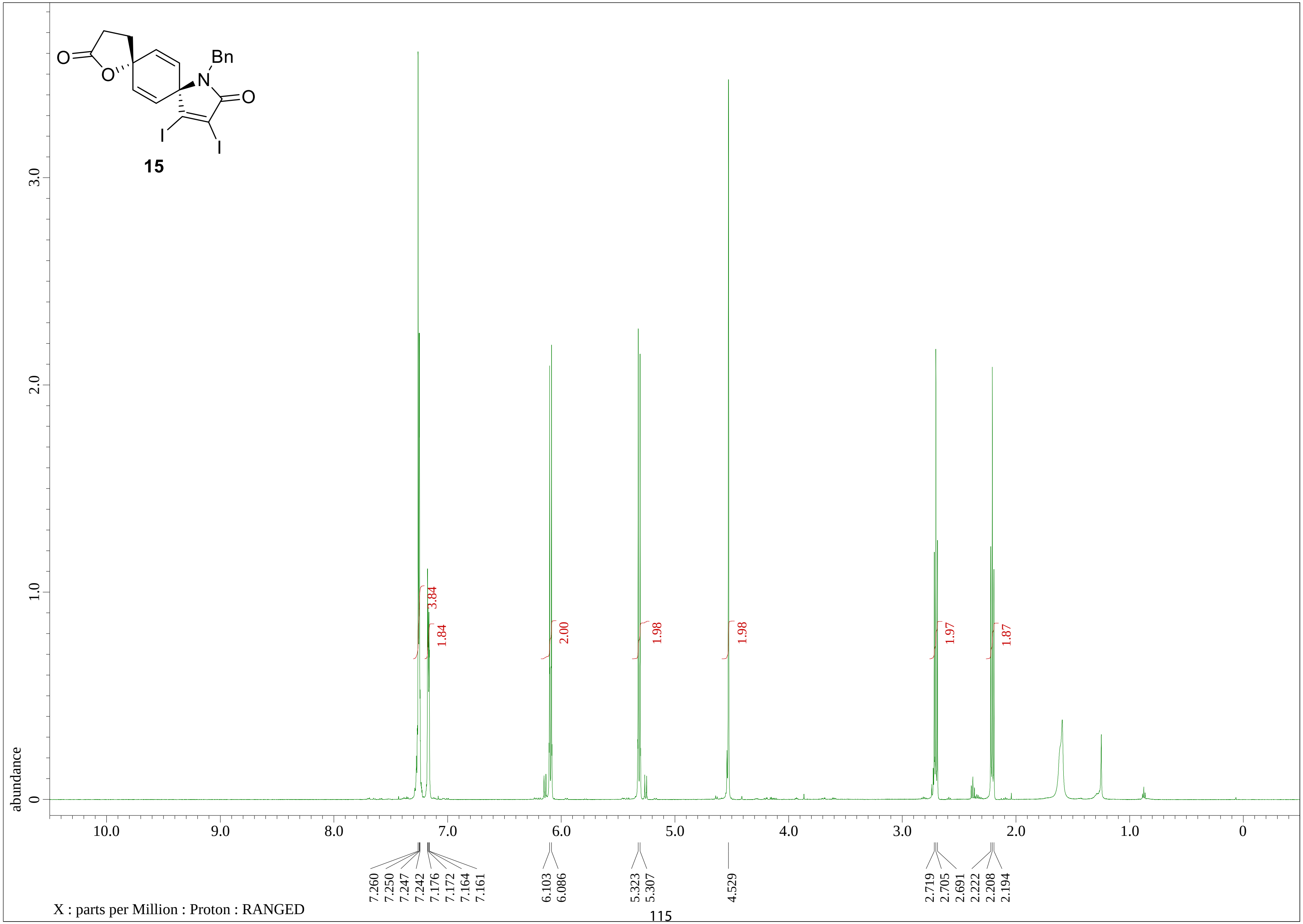

O
O
Bn
N
O
I
I
15
abundance
3.0
2.0
1.0
0
3.84
1.84
2.00
1.98
1.98
1.97
1.87
10.0
9.0
8.0
7.0
6.0
5.0
4.0
3.0
2.0
1.0
0
7.260
7.250
7.247
7.242
7.176
7.172
7.164
7.161
6.103
6.086
5.323
5.307
4.529
2.719
2.705
2.691
2.222
2.208
2.194
X : parts per Million : Proton : RANGED

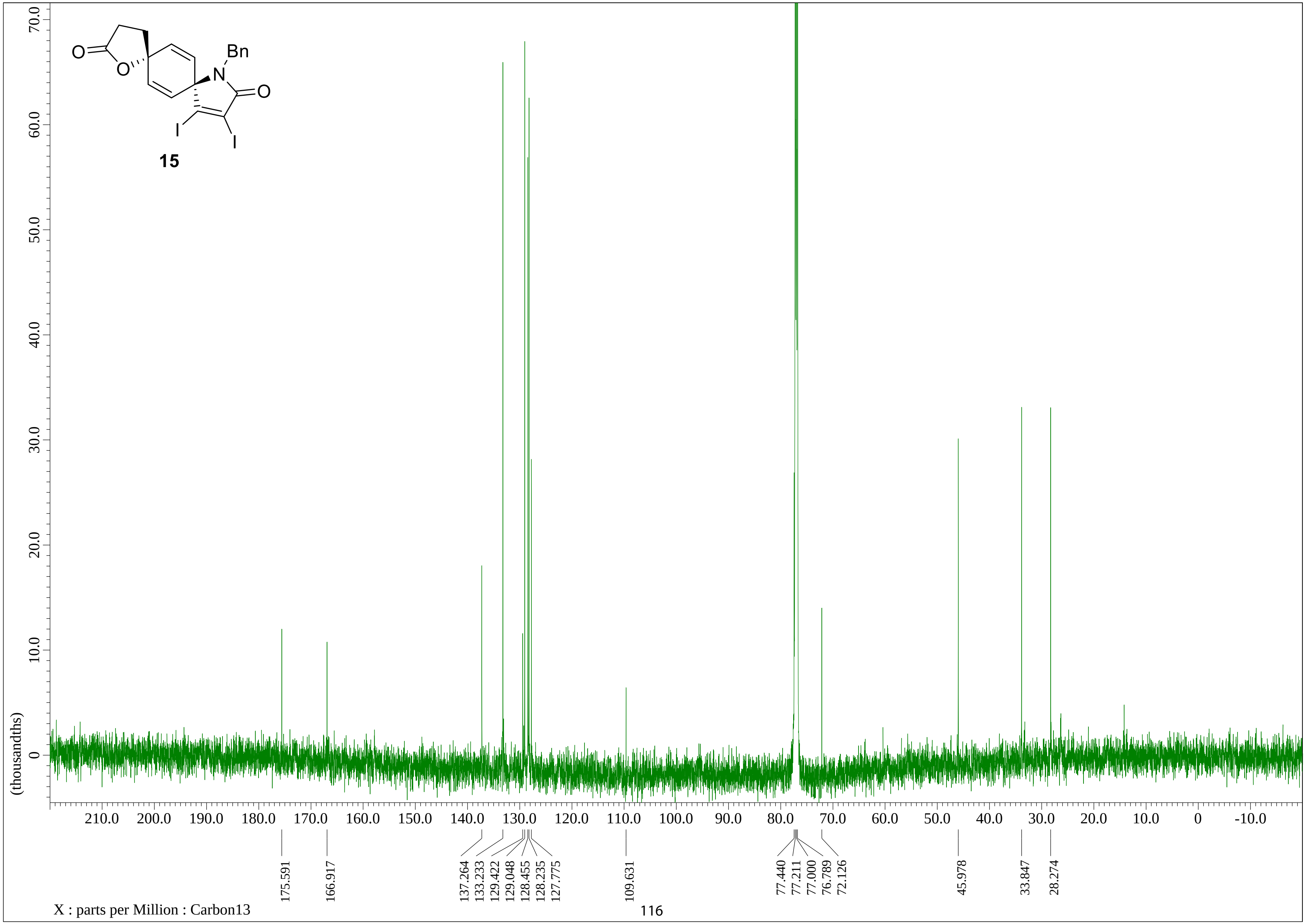
Bn
N
O
O
O
O
I
I
15
(thousandths)
70.0
60.0
50.0
40.0
30.0
20.0
10.0
0
210.0 200.0 190.0 180.0 170.0 160.0 150.0 140.0 130.0 120.0 110.0 100.0 90.0 80.0 70.0 60.0 50.0 40.0 30.0 20.0 10.0 0 -10.0
175.591
166.917
137.264
133.233
129.422
129.048
128.455
128.235
127.775
109.631
77.440
77.211
77.000
76.789
72.126
45.978
33.847
28.274
X : parts per Million : Carbon13

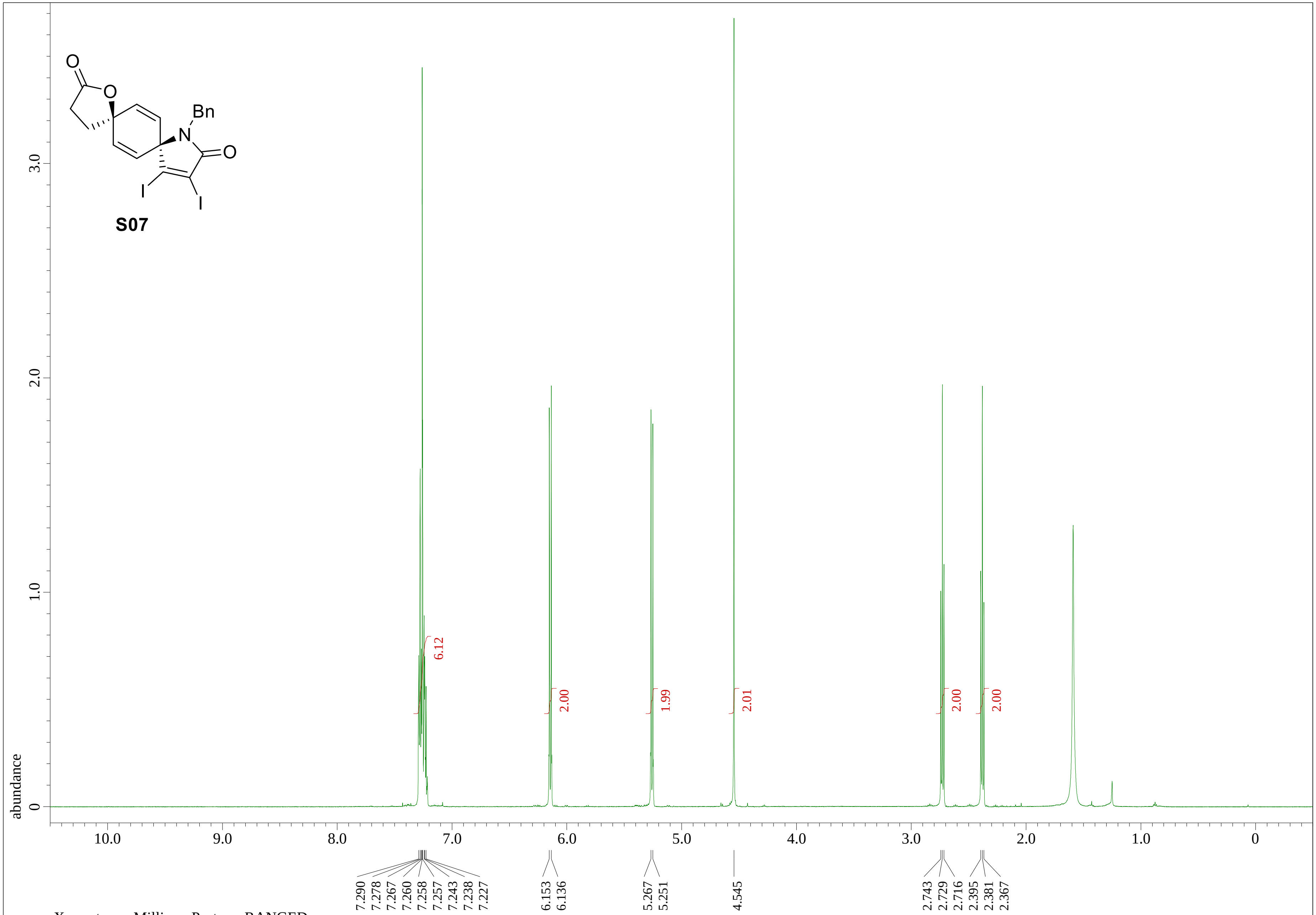
S07
Bn
abundance
3.0
2.0
1.0
0
6.12
2.00
1.99
2.01
2.00
2.00
10.0
9.0
8.0
7.0
6.0
5.0
4.0
3.0
2.0
1.0
0
7.290
7.278
7.267
7.260
7.258
7.257
7.243
7.238
7.227
6.153
6.136
5.267
5.251
4.545
2.743
2.729
2.716
2.395
2.381
2.367
X : parts per Million : Proton : RANGED

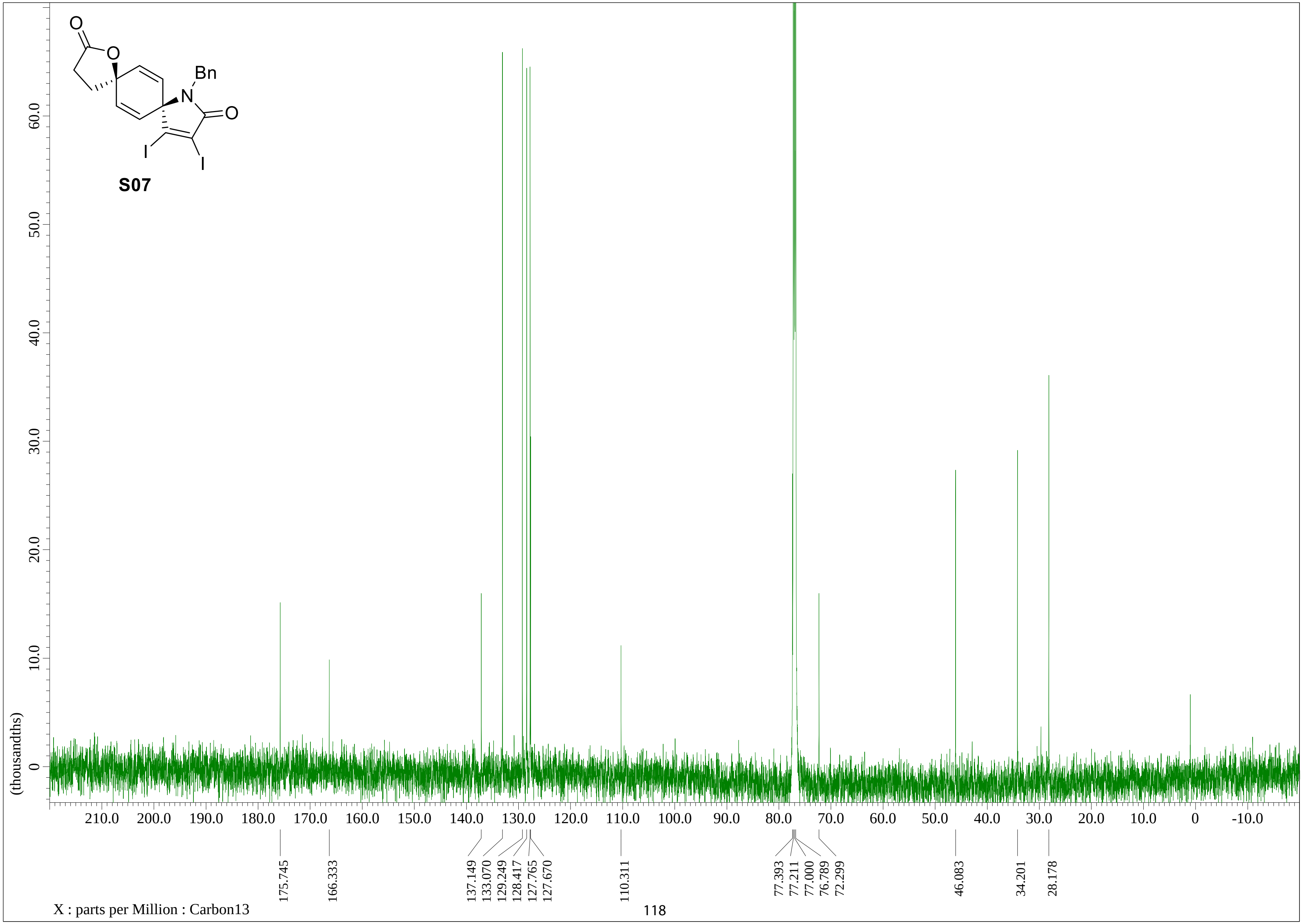
Bn
S07
(thousandths)
175.745
166.333
137.149
133.070
129.249
128.417
127.765
127.670
110.311
77.393
77.211
77.000
76.789
72.299
46.083
34.201
28.178
X : parts per Million : Carbon13

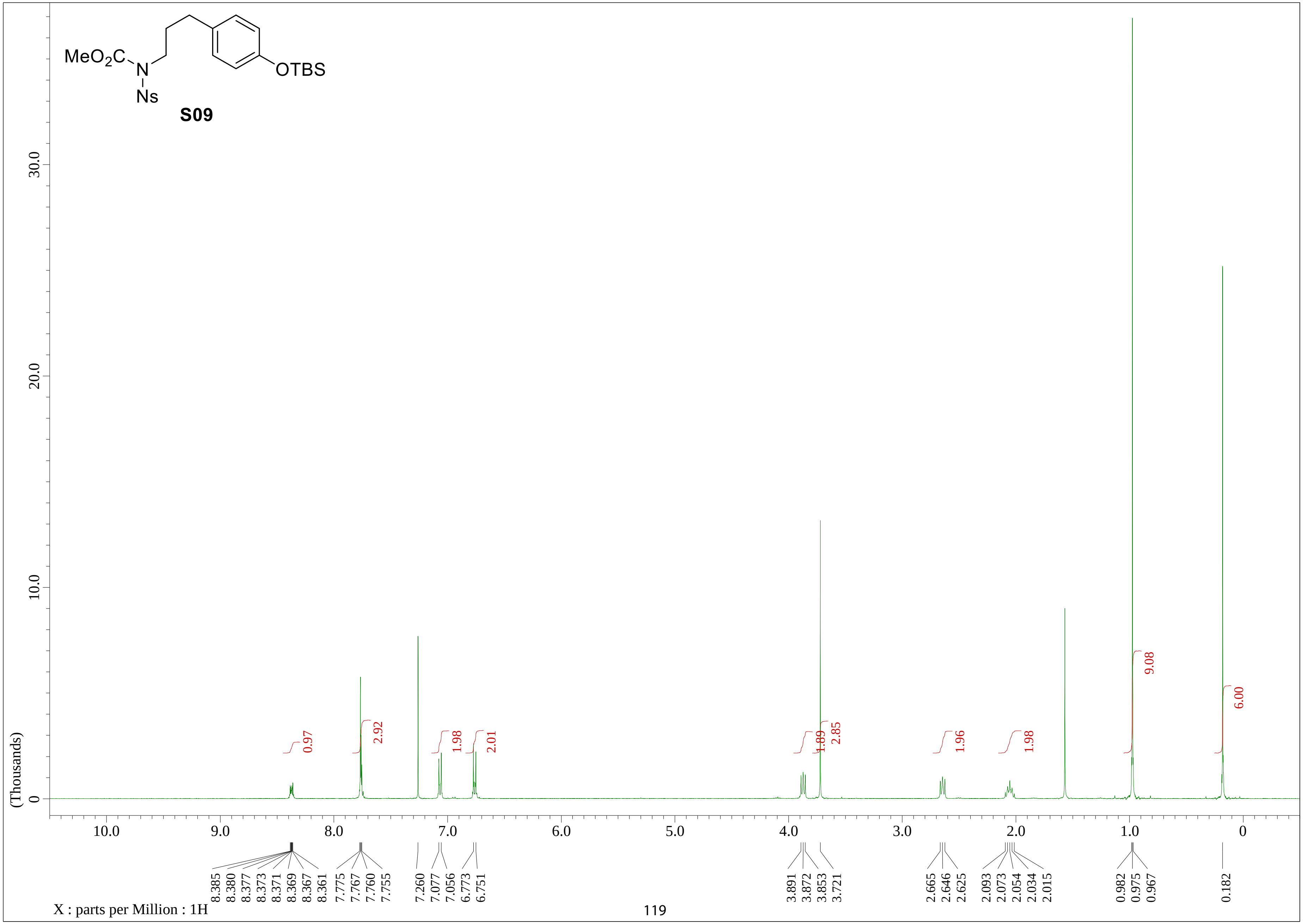
MeO2C
N
Ns
OTBS
S09
30.0
20.0
10.0
0
(Thousands)
0.97
2.92
1.98
2.01
1.89
2.85
1.96
1.98
9.08
6.00
10.0
9.0
8.0
7.0
6.0
5.0
4.0
3.0
2.0
1.0
0
8.385
8.380
8.377
8.373
8.371
8.369
8.367
8.361
7.775
7.767
7.760
7.755
7.260
7.077
7.056
6.773
6.751
3.891
3.872
3.853
3.721
2.665
2.646
2.625
2.093
2.073
2.054
2.034
2.015
0.982
0.975
0.967
0.182
X : parts per Million : 1H

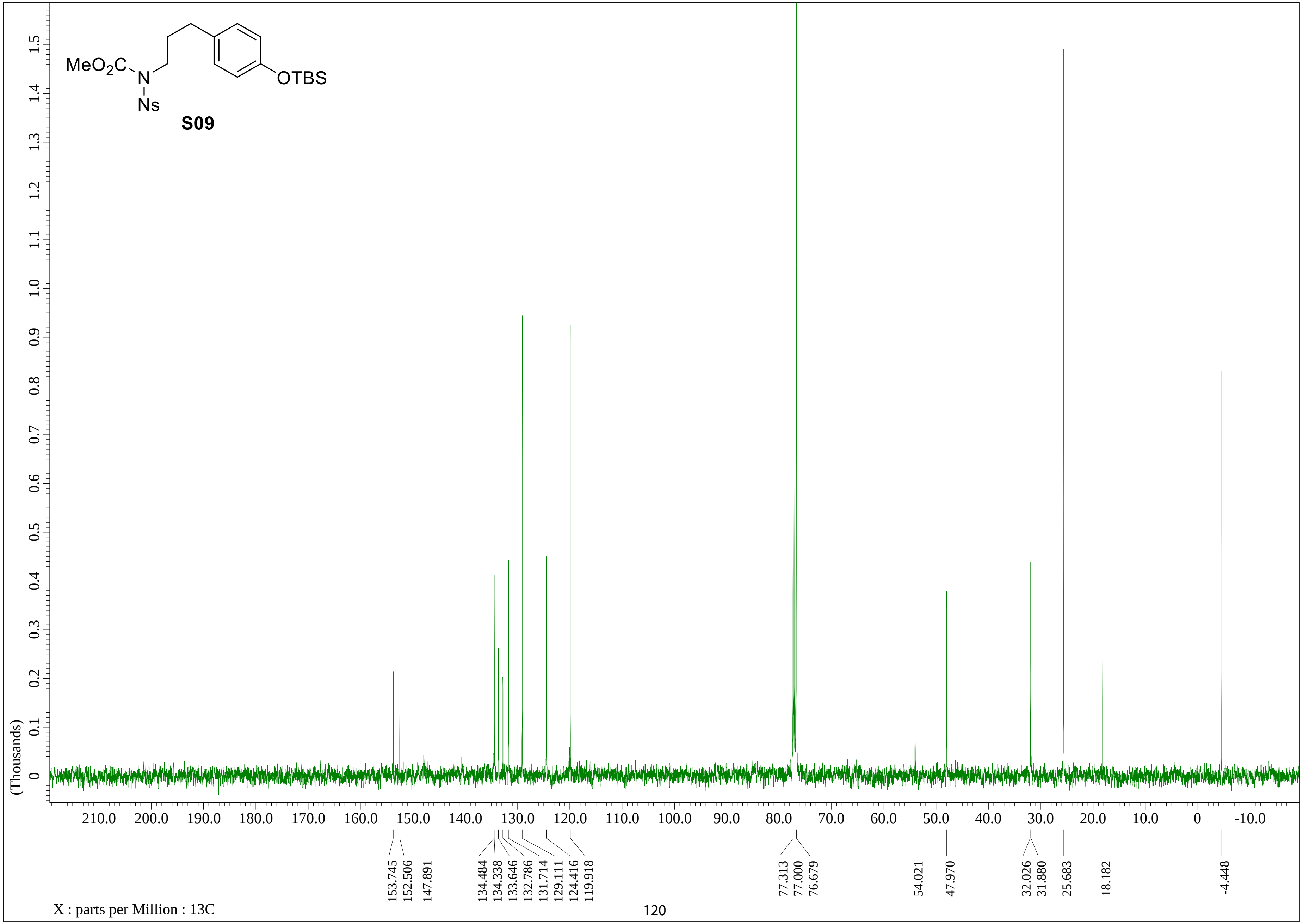
MeO2C
N
Ns
OTBS
S09
(Thousands)
0
0.1
0.2
0.3
0.4
0.5
0.6
0.7
0.8
0.9
1.0
1.1
1.2
1.3
1.4
1.5
210.0 200.0 190.0 180.0 170.0 160.0 150.0 140.0 130.0 120.0 110.0 100.0 90.0 80.0 70.0 60.0 50.0 40.0 30.0 20.0 10.0 0 -10.0
153.745
152.506
147.891
134.484
134.338
133.646
132.786
131.714
129.111
124.416
119.918
77.313
77.000
76.679
54.021
47.970
32.026
31.880
25.683
18.182
-4.448
X : parts per Million : 13C

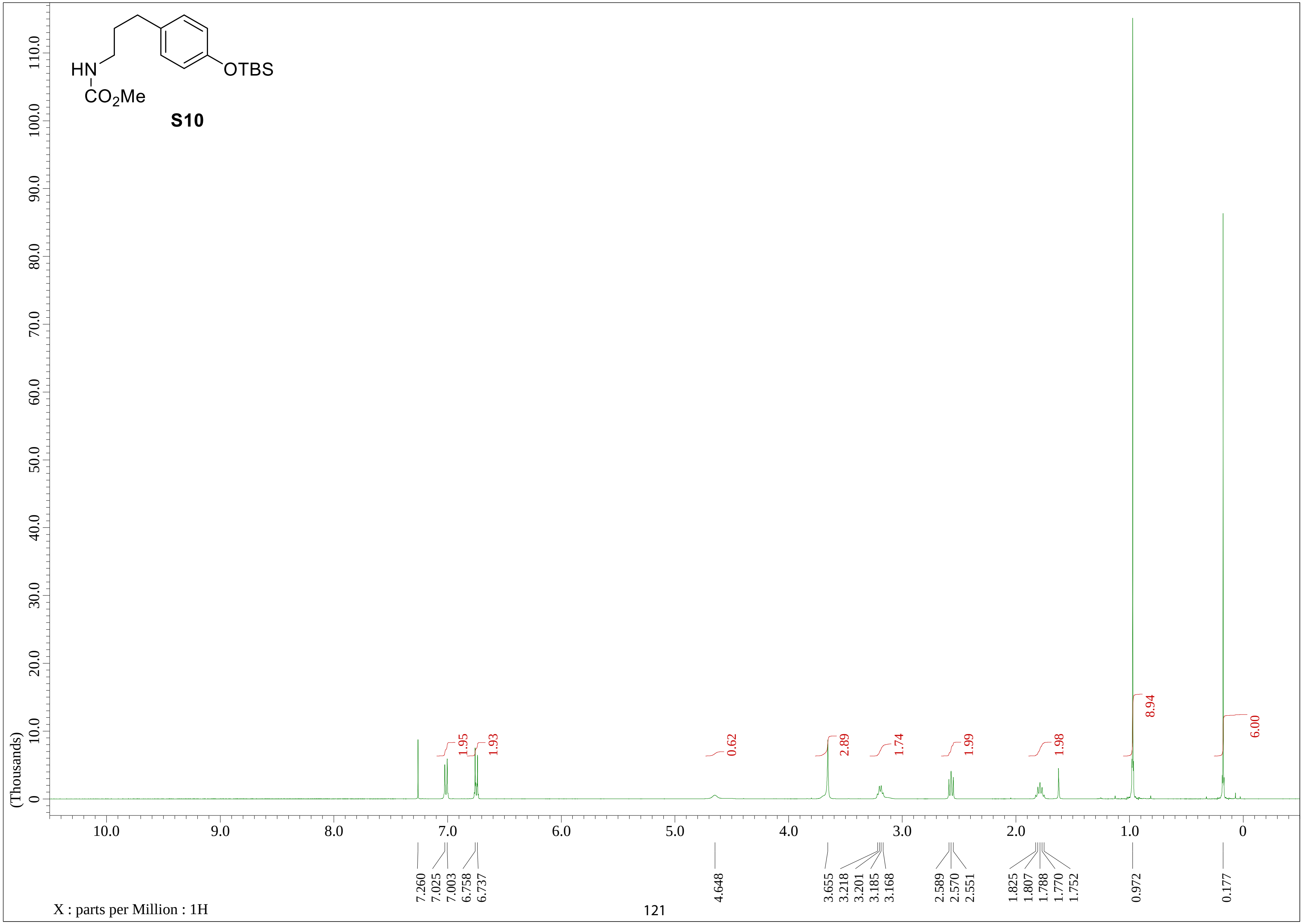
HN
CO2Me
OTBS
S10
(Thousands)
0
10.0
20.0
30.0
40.0
50.0
60.0
70.0
80.0
90.0
100.0
110.0
1.95
1.93
0.62
2.89
1.74
1.99
1.98
8.94
6.00
10.0
9.0
8.0
7.0
6.0
5.0
4.0
3.0
2.0
1.0
0
7.260
7.025
7.003
6.758
6.737
4.648
3.655
3.218
3.201
3.185
3.168
2.589
2.570
2.551
1.825
1.807
1.788
1.770
1.752
0.972
0.177
X : parts per Million : 1H

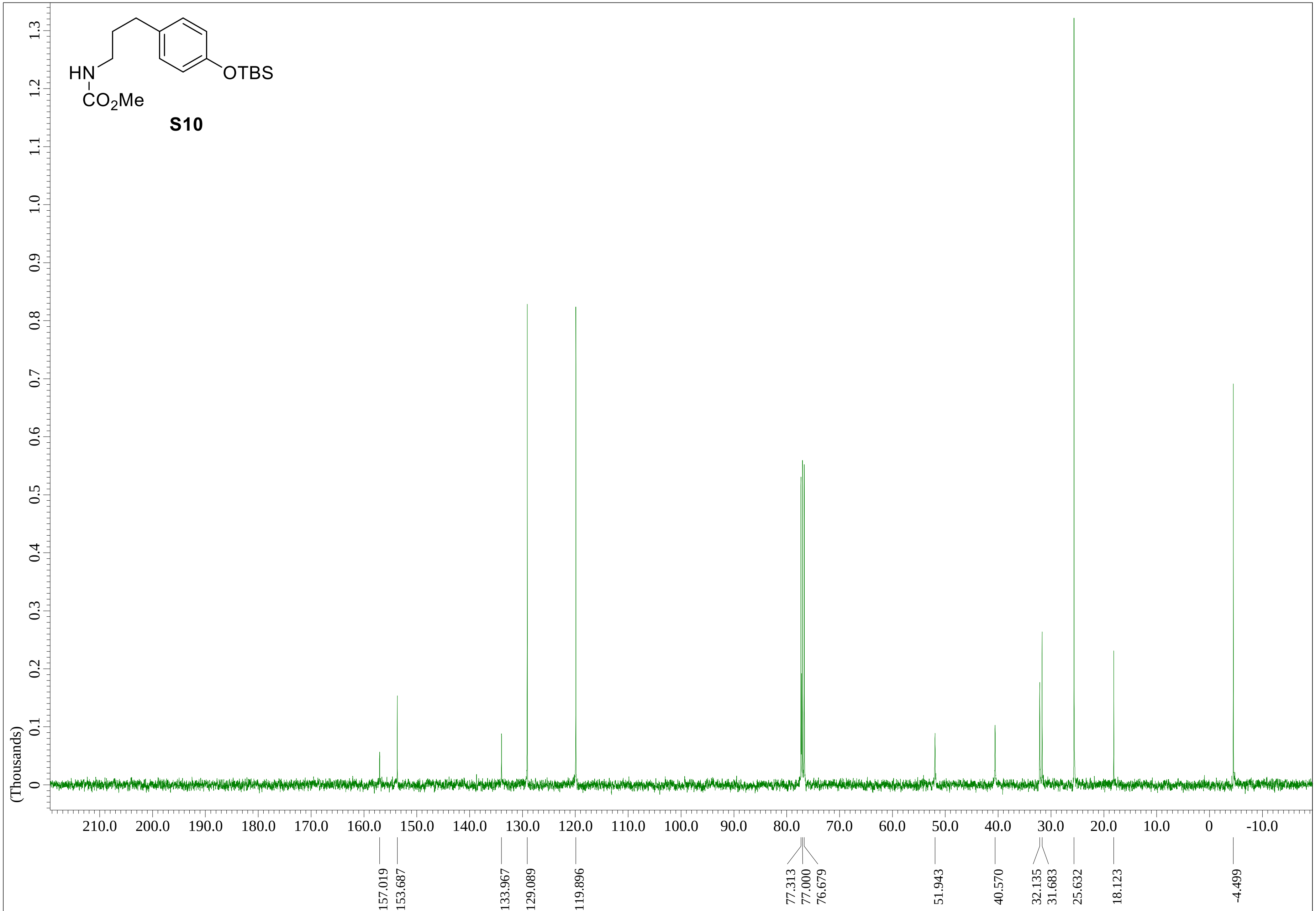

HN
CO2Me
OTBS
S10
(Thousands)
X : parts per Million : 13C
157.019
153.687
133.967
129.089
119.896
77.313
77.000
76.679
51.943
40.570
32.135
31.683
25.632
18.123
-4.499

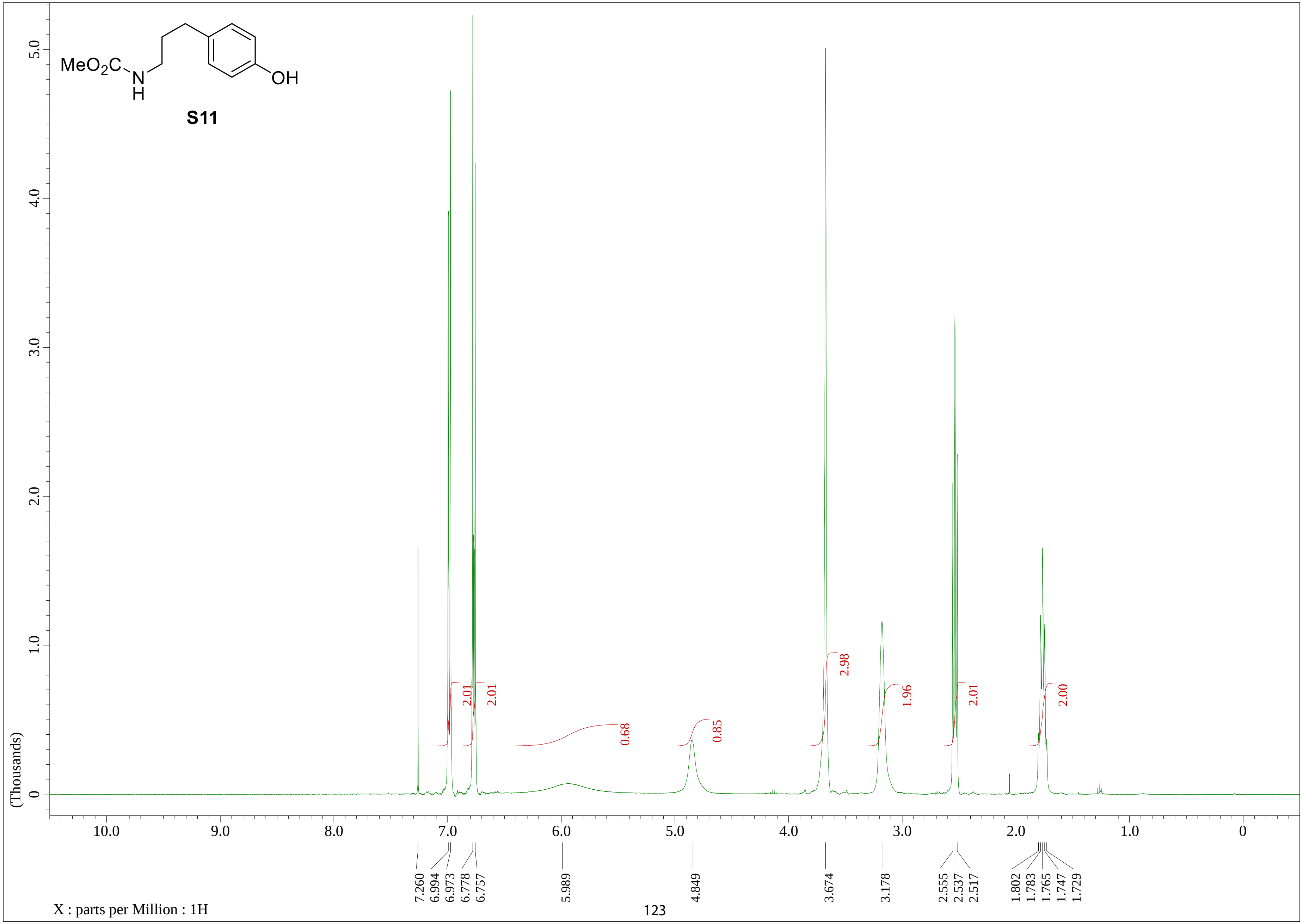
MeO2C
N
H
OH
S11
(Thousands)
X : parts per Million : 1H
7.260
6.994
6.973
6.778
6.757
5.989
4.849
3.674
3.178
2.555
2.537
2.517
1.802
1.783
1.765
1.747
1.729
2.01
2.01
0.68
0.85
2.98
1.96
2.01
2.00

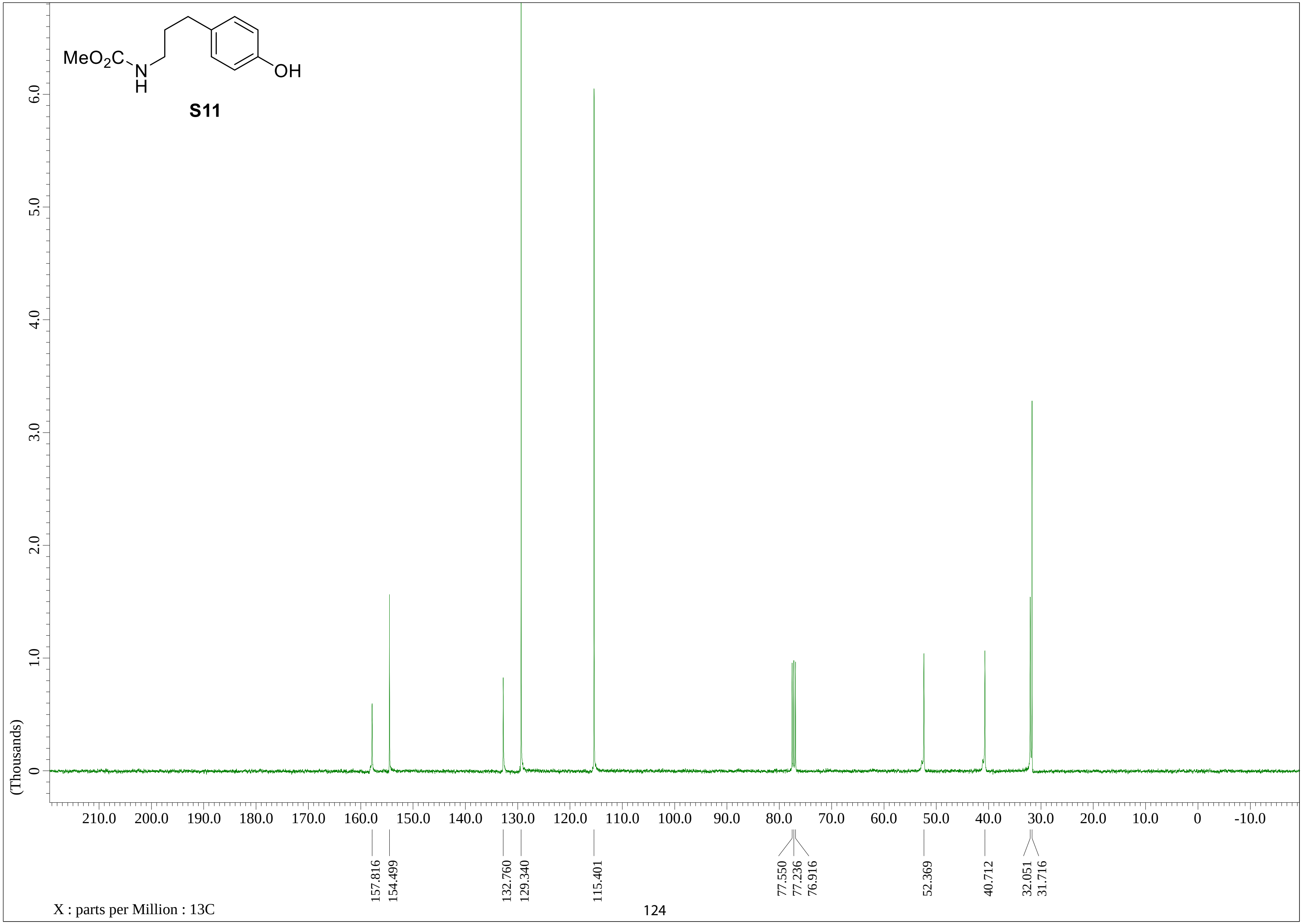

MeO2C
N
H
OH
S11
(Thousands)
0
1.0
2.0
3.0
4.0
5.0
6.0
210.0 200.0 190.0 180.0 170.0 160.0 150.0 140.0 130.0 120.0 110.0 100.0 90.0 80.0 70.0 60.0 50.0 40.0 30.0 20.0 10.0 0 -10.0
157.816
154.499
132.760
129.340
115.401
77.550
77.236
76.916
52.369
40.712
32.051
31.716
X : parts per Million : 13C

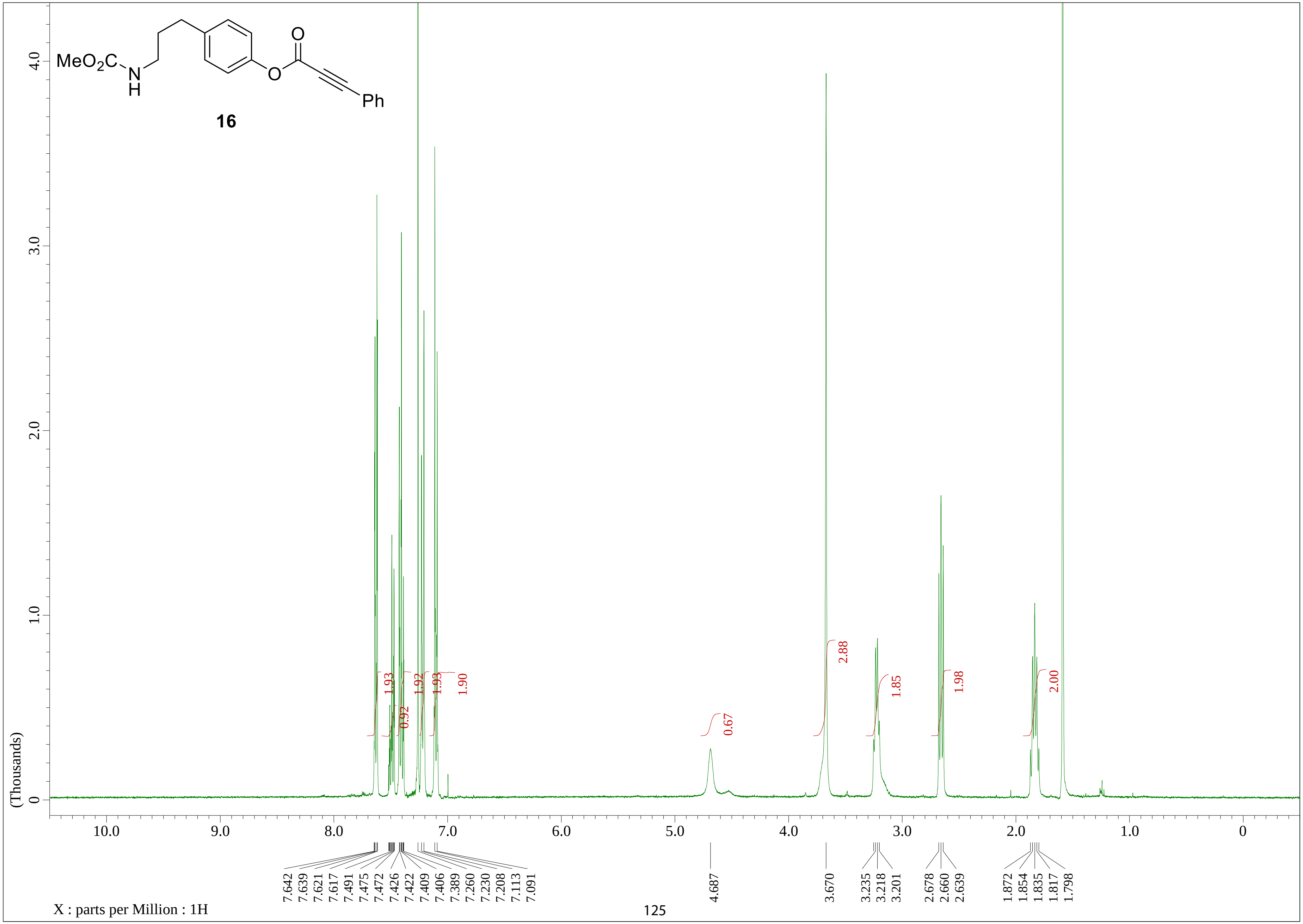
MeO2C
N
H
O
O
Ph
16
(Thousands)
X : parts per Million : 1H
7.642
7.639
7.621
7.617
7.491
7.475
7.472
7.426
7.422
7.409
7.406
7.389
7.260
7.230
7.208
7.113
7.091
4.687
3.670
3.235
3.218
3.201
2.678
2.660
2.639
1.872
1.854
1.835
1.817
1.798
1.93
0.92
1.92
1.93
1.90
0.67
2.88
1.85
1.98
2.00

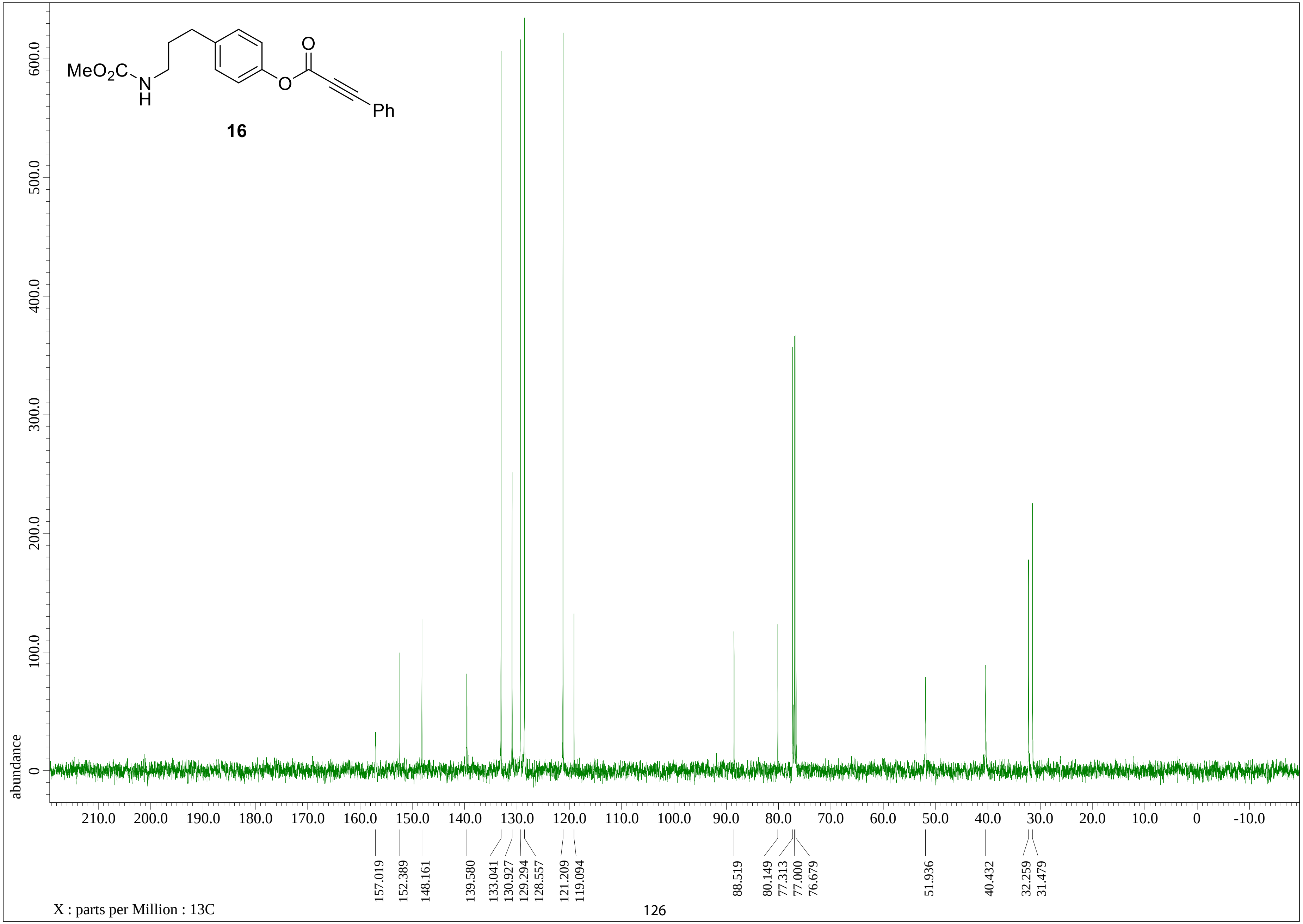

MeO2C
N
H
O
O
O
Ph
16
abundance
600.0
500.0
400.0
300.0
200.0
100.0
0
210.0 200.0 190.0 180.0 170.0 160.0 150.0 140.0 130.0 120.0 110.0 100.0 90.0 80.0 70.0 60.0 50.0 40.0 30.0 20.0 10.0 0 -10.0
157.019
152.389
148.161
139.580
133.041
130.927
129.294
128.557
121.209
119.094
88.519
80.149
77.313
77.000
76.679
51.936
40.432
32.259
31.479
X : parts per Million : 13C

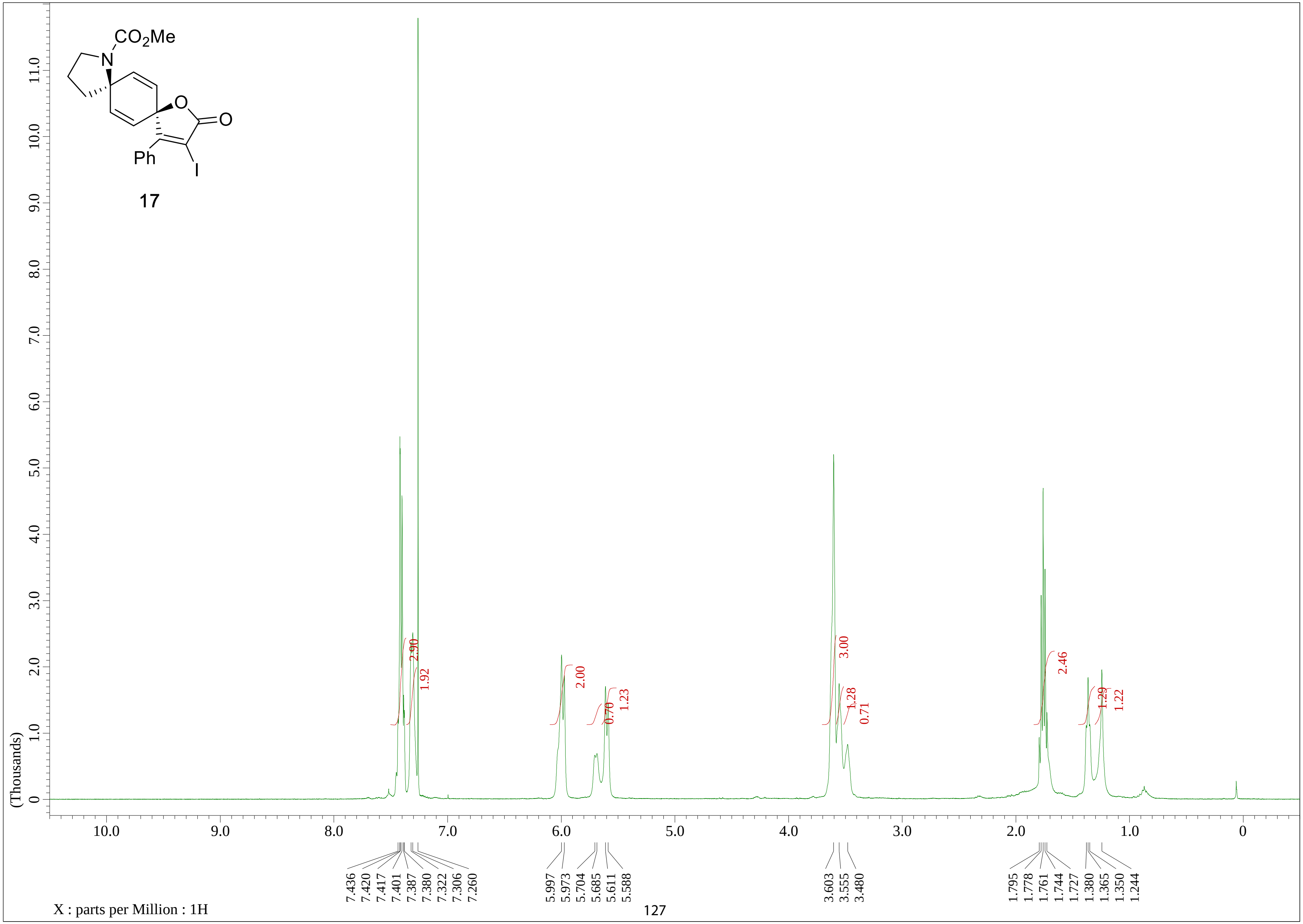
CO2Me
N
O
O
Ph
I
(Thousands)
X : parts per Million : 1H
7.436
7.420
7.417
7.401
7.387
7.380
7.322
7.306
7.260
5.997
5.973
5.704
5.685
5.611
5.588
3.603
3.555
3.480
1.795
1.778
1.761
1.744
1.727
1.380
1.365
1.350
1.244
2.90
1.92
2.00
0.70
1.23
3.00
1.28
0.71
2.46
1.29
1.22

17

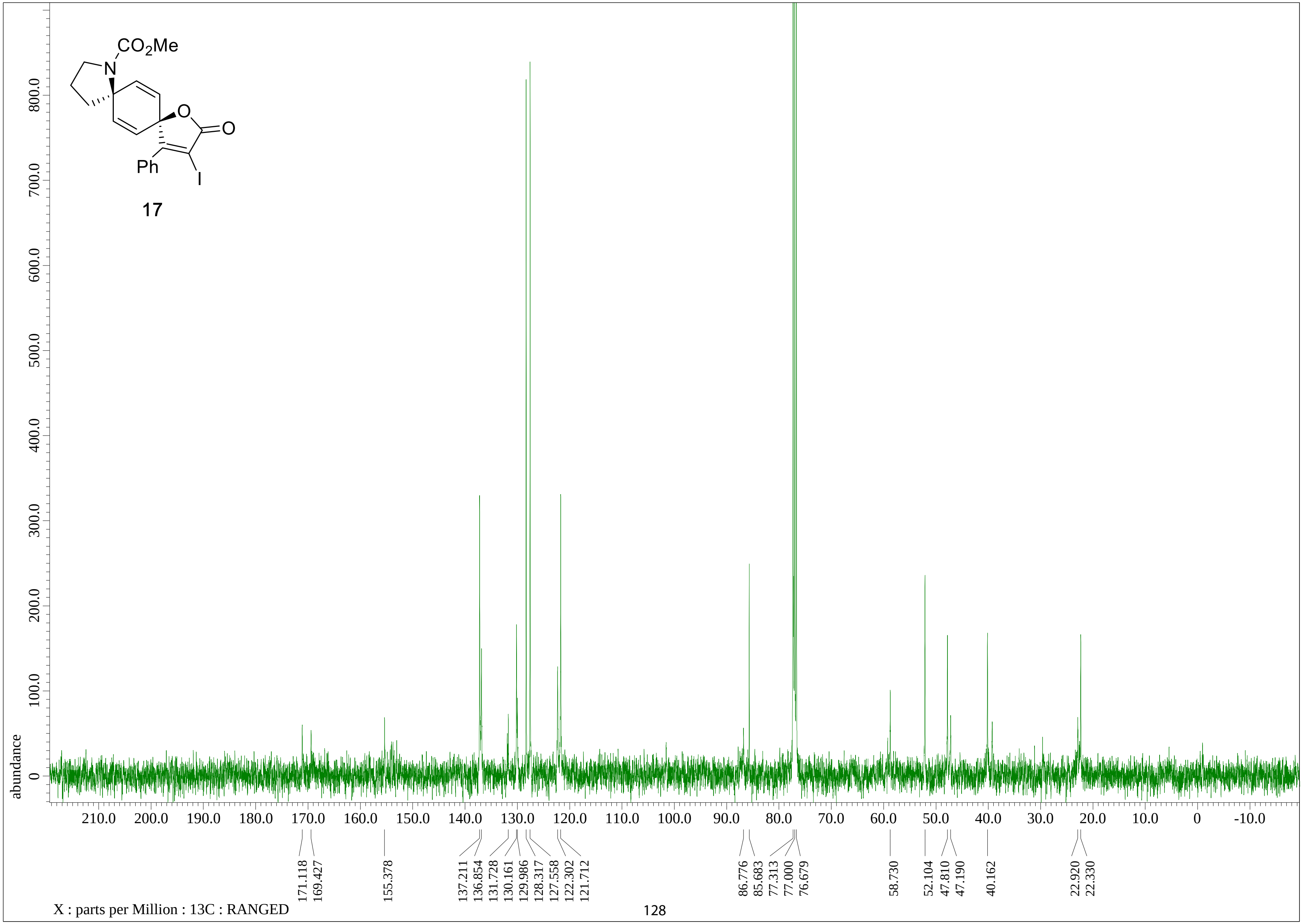
CO2Me
N
O
O
Ph
I
17
abundance
800.0
700.0
600.0
500.0
400.0
300.0
200.0
100.0
0
210.0 200.0 190.0 180.0 170.0 160.0 150.0 140.0 130.0 120.0 110.0 100.0 90.0 80.0 70.0 60.0 50.0 40.0 30.0 20.0 10.0 0 -10.0
171.118
169.427
155.378
137.211
136.854
131.728
130.161
129.986
128.317
127.558
122.302
121.712
86.776
85.683
77.313
77.000
76.679
58.730
52.104
47.810
47.190
40.162
22.920
22.330
X : parts per Million : 13C : RANGED

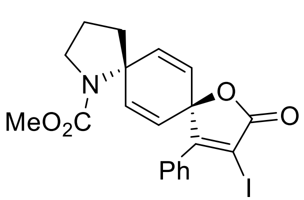
MeO2C
N
O
O
Ph
I

S12

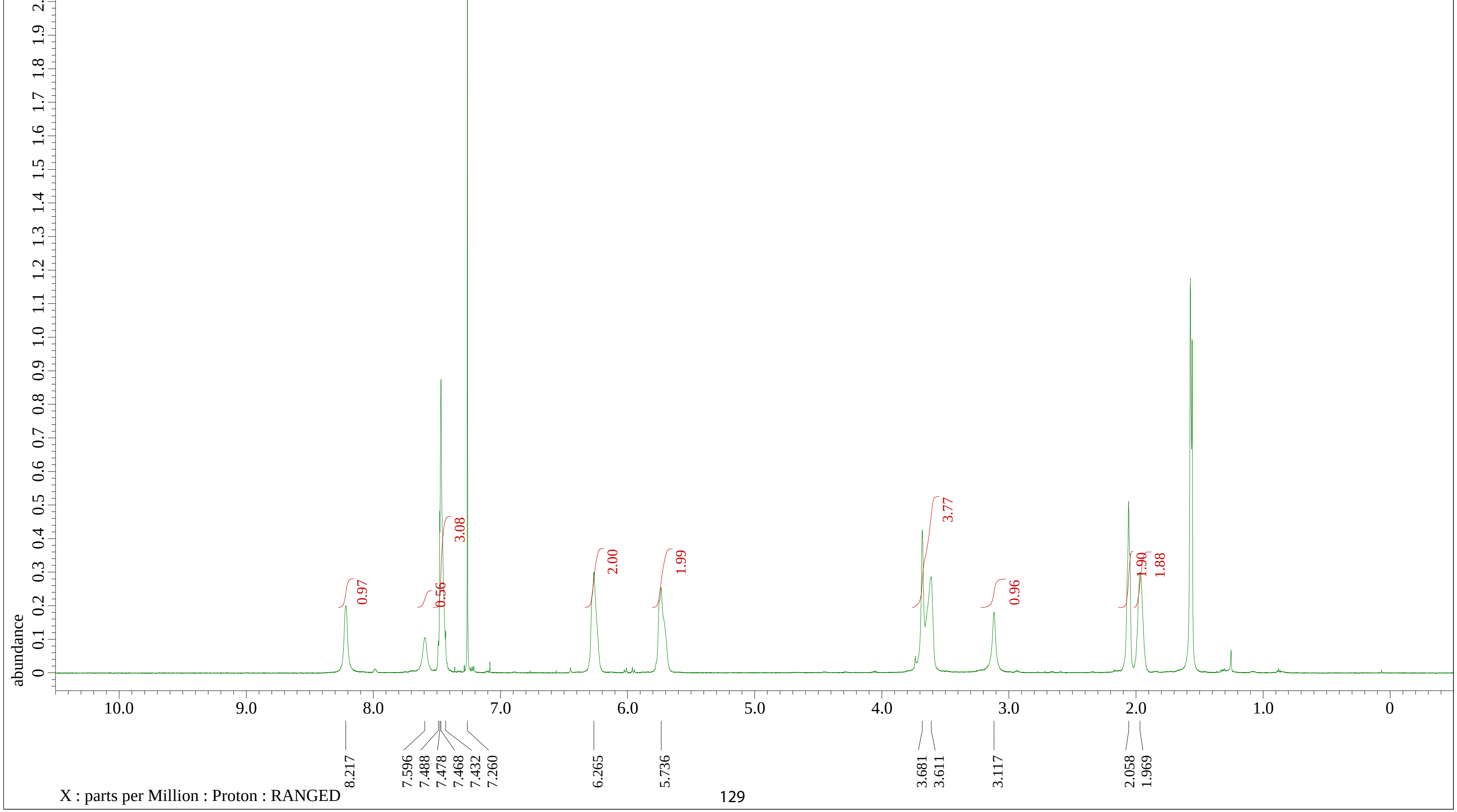
abundance
0.97
0.56
3.08
2.00
1.99
3.77
0.96
1.90
1.88
8.217
7.596
7.488
7.478
7.468
7.432
7.260
6.265
5.736
3.681
3.611
3.117
2.058
1.969
X : parts per Million : Proton : RANGED

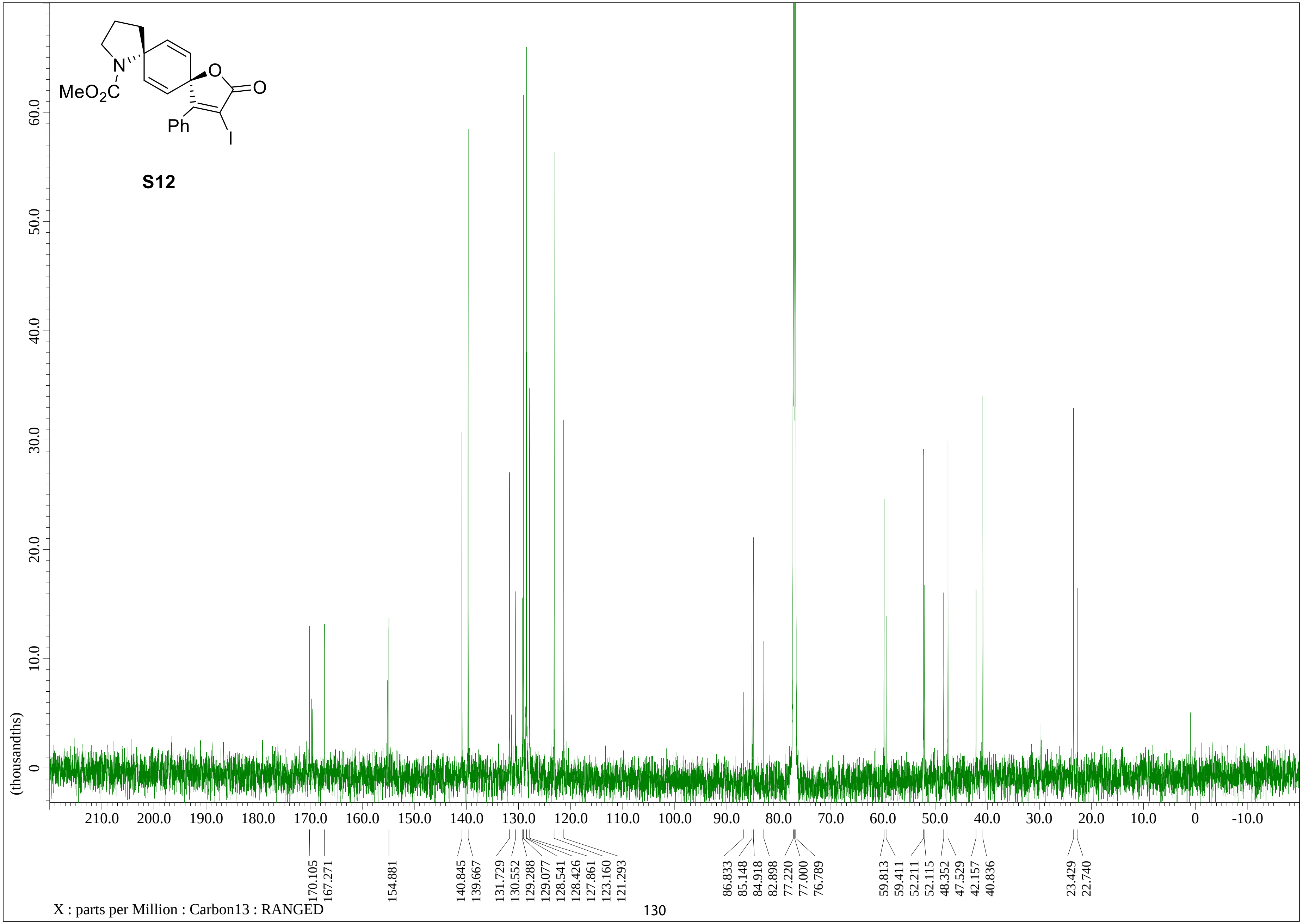
MeO2C
N
O
O
Ph
I
S12
(thousandths)
60.0
50.0
40.0
30.0
20.0
10.0
0
210.0 200.0 190.0 180.0 170.0 160.0 150.0 140.0 130.0 120.0 110.0 100.0 90.0 80.0 70.0 60.0 50.0 40.0 30.0 20.0 10.0 0 -10.0
170.105
167.271
154.881
140.845
139.667
131.729
130.552
129.288
129.077
128.541
128.426
127.861
123.160
121.293
86.833
85.148
84.918
82.898
77.220
77.000
76.789
59.813
59.411
52.211
52.115
48.352
47.529
42.157
40.836
23.429
22.740
X : parts per Million : Carbon13 : RANGED
